# Supplementary material for: Chromium-catalyzed para-selective formation of quaternary carbon centers by alkylation of benzamide derivatives
Source: Nat Commun. 2018 Nov 6;9:4637. doi: 10.1038/s41467-018-07069-1 (PMC6219510; doi:10.1038/s41467-018-07069-1)
Supplement: Supplementary file 1 — Description of Additional Supplementary Files [file 41467_2018_7069_MOESM1_ESM.pdf]

Description of Additional Supplementary Files

File Name: Supplementary Data 1
Description: Cif Document for Product 3
