# Supplementary material for: Chromium-catalyzed para-selective formation of quaternary carbon centers by alkylation of benzamide derivatives
Source: Nat Commun. 2018 Nov 6;9:4637. doi: 10.1038/s41467-018-07069-1 (PMC6219510; doi:10.1038/s41467-018-07069-1)
Supplement: Supplementary file 3 — Supplementary Information [file 41467_2018_7069_MOESM3_ESM.pdf]

Supplementary Information for

Chromium-Catalyzed *para*-Selective Formation of Quaternary Carbon Centers by Alkylation of Benzamide Derivatives

Liu et, al.

Supplementary Methods

General Information

All reactions dealing with air- or moisture-sensitive compounds were carried out in a flame-dried, sealed Schlenk reaction tube under an atmosphere of nitrogen. Analytical thin-layer chromatography was performed on glass plates coated with 0.25 mm 230–400 mesh silica gel containing a fluorescent indicator (Merck). Flash silica gel column chromatography was performed on silica gel 60N (spherical and neutral, 140–325 mesh) as described by Still.[1] NMR spectra were measured on a Bruker AV-400 spectrometer and reported in parts per million. ^{1}H NMR spectra were recorded at 400 MHz in $CDCl_3$ were referenced internally to tetramethylsilane as a standard, and ^{13}C NMR spectra were recorded at 100 MHz and referenced to the solvent resonance. Analytical gas chromatography (GC) was carried out on a Thermo Trace 1300 gas chromatograph or TECHCOMP GC-7900 (for analysis of hydrogen evolution), equipped with a flame ionization detector. Mass spectra (GC-MS) were taken at Thermo Trace 1300 gas chromatograph mass spectrometer or Pfeiffer Vacuum Omni Star GSD 320 for the analysis of hydrogen evolution. High resolution mass spectra (HRMS) were recorded on the Exactive Mass Spectrometer (Thermo Scientific, USA) equipped with ESI ionization source. Melting points were determined with a Hanon MP-300. Electron paramagnetic resonance (EPR) spectrum was recorded on an instrument of Bruker A300-9.5/12.

Unless otherwise noted, materials were purchased from Tokyo Chemical Industry Co., Aldrich Inc., Alfa Aesar, Adamas, and other commercial suppliers and used as received. Solvents were dried over sodium (for THF and ether) by refluxing for overnight and freshly distilled prior to use. Grignard reagents were purchased from commercial suppliers or prepared by the reaction between related organic halides and magnesium turnings in anhydrous THF, and titrated prior to use. The purities of metal salts: $CrCl_3$ (99.99%), $CrCl_2$ (99.99%), $Cr(acac)_3$ (97%), $FeCl_2$ (98%), $CoCl_2$ (99.9%).

Procedure for the Preparation of Substituted benzamides

General procedure A: Benzoyl chlorides was slowly dropwised into the solution of methylamine (1.5 equiv, aqueous), Et_3N (2 equiv) and DCM (2 M) at ice-water bath. The mixture was warmed to room temperature and stirred for 3 h. After removing the volatiles under vacuum, the crude product was then purified by flash chromatography on silica gel to give the corresponding benzamides (85–96% yield).

General procedure B: In a dried flask, substituted benzoic acid was dissolved in DCM and then a few drops of DMF were added. The resulting mixture was added slowly into the solution of oxalyl dichloride (3 equiv) in DCM. After stirring at room temperature for 6 h, the volatiles were removed under vacuum. The crude product was used directly for next-step synthesis.

The prepared benzoyl chloride was added to the solution of methylamine (1.5 equiv, aqueous), Et_3N (2 equiv) and DCM (2 M) at ice-water bath. The mixture was warmed to room temperature and stirred for 3 h. The crude product was then purified by flash chromatography on silica gel to give the corresponding benzamides (60–96% yield).

General procedure C: 2-Bromo-*N*-methylbenzamide (5 mmol), phenylboronic acids (1.5 equiv), $Pd(OAc)_2$ (0.1 equiv) and sodium carbonate (2 equiv) were putted into a dried flask, and 10 mL of DMF/H_2O (v/v = 2:1) was added under nitrogen atmosphere. The solution was then stirred at 80 ^{o}C for 20 h. After cooling to the room temperature, 30 mL of H_2O was added and the organic phase was extracted with EtOAc (3 x 10 mL). The combined organic phase was dried over anhydrous Na_2SO_4 and concentrated under vacuum. The crude product was purified by flash chromatography on silica gel to give the corresponding benzamides (60–85% yield).

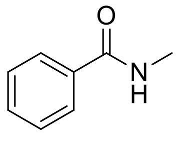

***N*-methylbenzamide (1a)**

The title compound was prepared according to the procedure A as a white solid (94% yield). Melting point: 77–79°C. ^{1}H NMR (400 MHz, $CDCl_3$): δ = 7.76 (d, J = 7.7 Hz, 2H), 7.47–7.44 (m, 1H), 7.40–7.37 (m, 2H), 6.61 (brs, 1H), 2.97 (d, J = 4.3 Hz, 3H);

^{13}C NMR (100 MHz, $CDCl_3$): δ = 168.3, 134.5, 131.2, 128.4, 126.8, 26.7. IR (neat): 3322, 1634, 1577, 1548, 1491, 1407, 1308, 1163, 1076, 935, 830, 708, 694 cm^{-1}. GC-MS (EI): calcd for C_8H_9NO [M^+] 135.07, found 135.10. Spectroscopic data are in accordance with those described in the literature.[2]

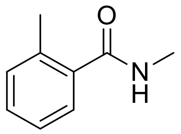

N, 2-dimethylbenzamide (1b)

The title compound was prepared according to the procedure A as a white solid (90% yield). Melting point: 79–81 °C. ^1H NMR (400 MHz, $CDCl_3$): δ = 7.28 (s, 2H), 7.23–7.10 (m, 2H), 6.03 (brs, 1H), 2.93 (s, 3H), 2.40 (s, 3H). ^{13}C NMR (100 MHz, $CDCl_3$): δ = 170.8, 136.5, 135.9, 130.8, 129.6, 126.6, 125.6, 26.5, 19.6. IR (neat): 3290, 1632, 1540, 1405, 1320, 1286, 1172, 784, 720, 692 cm^{-1}. GC-MS (EI): calcd for $C_9H_{11}NO$ [M^+] 149.08, found 149.11. Spectroscopic data are in accordance with those described in the literature.[3]

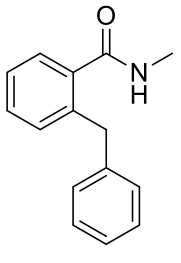

2-Benzyl-*N*-methylbenzamide (1c)

The title compound was prepared according to the procedure A as a white solid (88% yield). Melting point: 108–110 °C. ^1H NMR (400 MHz, $CDCl_3$): δ = 7.33 (t, J = 7.2 Hz, 2H), 7.29–7.20 (m, 4H), 7.17 (t, J = 6.3 Hz, 3H), 5.63 (brs, 1H), 4.16 (s, 2H), 2.83 (d, J = 4.8 Hz, 3H). ^{13}C NMR (100 MHz, $CDCl_3$): δ = 170.7, 140.8, 138.8, 136.8, 130.9, 129.9, 128.9, 128.4, 127.0, 126.3, 126.0, 38.9, 26.5. IR (neat): 3287, 1635, 1573, 1494, 1318, 1170, 738, 727, 664 cm^{-1}. GC-MS (EI): calcd for $C_{15}H_{15}NO$ [M^+] 225.12., found 225.10.

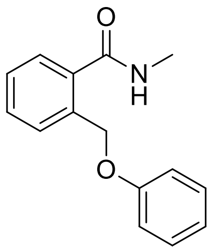

N-methyl-2-(phenoxymethyl)benzamide (1d)

The title compound was prepared according to the procedure A as a white solid (92% yield). Melting point: 101–103 °C. ^{1}H NMR (400 MHz, $CDCl_3$): δ = 7.54 (t, J = 7.5 Hz, 2H), 7.45–7.40 (m, 1H), 7.38–7.24 (m, 3H), 7.06–6.85 (m, 3H), 6.57 (brs, 1H), 5.18 (s, 2H), 2.89 (dd, J = 5.9, 3.5 Hz, 3H). ^{13}C NMR (100 MHz, $CDCl_3$): δ = 169.5, 158.3, 135.8, 134.6, 130.3, 129.5, 129.4, 128.2, 127.7, 121.3, 114.8, 68.2, 26.6. IR (neat): 3290, 1634, 1548, 1497, 1321, 1240, 1044, 749, 688 cm^{-1}. GC-MS (EI): calcd for $C_{15}H_{15}NO_2$ [M^+] 241.11., found 241.14.

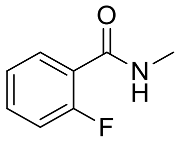

2-Fluoro-N-methylbenzamide (1e)

The title compound was prepared according to the procedure A as a white solid (88% yield). Melting point: 48–50 °C. ^{1}H NMR (400 MHz, $CDCl_3$): δ = 8.08–7.96 (m, 1H), 7.40 (d, J = 5.0 Hz, 1H), 7.21–7.17 (m, 1H), 7.08–7.03 (m, 1H), 6.82 (brs, 1H), 2.98 (s, 3H); ^{13}C NMR (100 MHz, $CDCl_3$): δ = 163.9 (d, $J_{C\text{-}F}$ = 2.9 Hz), 160.4 (d, $J_{C\text{-}F}$ = 245 Hz), 132.9 (d, $J_{C\text{-}F}$ = 9.2 Hz), 131.7 (d, $J_{C\text{-}F}$ = 2.4 Hz), 124.6 (d, $J_{C\text{-}F}$ = 3.0 Hz), 121.0 (d, $J_{C\text{-}F}$ = 11.8 Hz), 115.8 (d, $J_{C\text{-}F}$ = 25 Hz), 26.6; ^{19}F NMR (377 MHz, $CDCl_3$): δ = –114.0. IR (neat): 3355, 1646, 1538, 1308, 1308, 1216, 758 cm^{-1}. GC-MS (EI): calcd for C_8H_8FNO [M^+] 153.06, found 153.09. Spectroscopic data are in accordance with those described in the literature.[4]

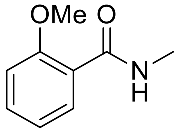

2-Methoxy-N-methylbenzamide (1f)

The title compound was prepared according to the procedure A as a colorless oil (85%

yield). ^{1}H NMR (400 MHz, $CDCl_3$): δ = 8.14–8.03 (m, 1H), 7.80 (brs, 1H), 7.34–7.29 (m, 1H), 6.99–6.90 (m, 1H), 6.88–6.82 (m, 1H), 3.83–3.82 (m, 3H), 2.93–2.86 (m, 3H); ^{13}C NMR (100 MHz, $CDCl_3$): δ = 165.7, 157.1, 132.3, 131.7, 121.2, 120.8, 111.0, 55.5, 26.2. IR (neat): 3411, 1643, 1532, 1483, 1407, 1296, 1238, 1161, 1104, 1019, 840, 754 cm^{-1}. GC-MS (EI): calcd for $C_9H_{11}NO_2$ [M$^+$] 165.08, found 165.09. Spectroscopic data are in accordance with those described in the literature.[2]

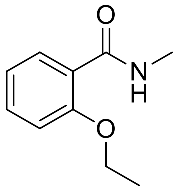

2-Ethoxy-*N*-methylbenzamide (1g)

The title compound was prepared according to the procedure A as a white solid (96% yield). Melting point: 78–80 °C. ^{1}H NMR (400 MHz, $CDCl_3$): δ = 8.21 (d, J = 7.8 Hz, 1H), 7.98 (brs, 1H), 7.38 (t, J = 7.8 Hz, 1H), 7.03 (t, J = 7.5 Hz, 1H), 6.92 (d, J = 8.3 Hz, 1H), 4.16 (q, J = 6.9 Hz, 2H), 2.98 (d, J = 4.8 Hz, 3H), 1.48 (t, J = 6.9 Hz, 3H). ^{13}C NMR (100 MHz, $CDCl_3$): δ = 166.0, 156.8, 132.5, 132.1, 132.1, 121.5, 121.1, 112.2, 64.6, 26.3, 14.7. IR (neat): 3342, 1636, 1531, 1487, 1263, 1162, 1037, 924, 799, 750 cm^{-1}. GC-MS (EI): calcd for $C_{10}H_{13}NO_2$ [M$^+$] 179.09., found 179.10.

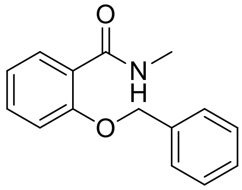

2-(Benzyloxy)-*N*-methylbenzamide (1h)

The title compound was prepared according to the procedure A as a white solid (95% yield). Melting point: 81–83 °C. ^{1}H NMR (400 MHz, $CDCl_3$): δ = 8.25 (dd, J = 7.8, 1.7 Hz, 1H), 7.90 (brs, 1H), 7.47–7.34 (m, 6H), 7.09 (t, J = 7.6 Hz, 1H), 7.03 (d, J = 8.3 Hz, 1H), 5.18 (s, 2H), 2.90 (d, J = 4.8 Hz, 3H). ^{13}C NMR (100 MHz, $CDCl_3$): δ = 165.8, 156.7, 135.8, 132.5, 132.2, 128.9, 128.6, 127.5, 122.0, 121.6, 112.8, 71.3, 26.3. IR (neat): 3395, 1645, 1498, 1301, 1253, 1001, 858, 760, 698 cm^{-1}. GC-MS (EI): calcd for $C_{15}H_{15}NO_2$ [M$^+$] 241.11., found 241.13.

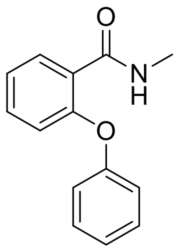

***N*-methyl-2-phenoxybenzamide (1i)**

The title compound was prepared according to the procedure A as a white solid (94% yield). Melting point: 113–115 °C. ^{1}H NMR (400 MHz, $CDCl_3$): δ = 8.25 (d, J = 7.9 Hz, 1H), 7.67 (brs, 1H), 7.41–7.32 (m, 3H), 7.24–7.14 (m, 2H), 7.06 (d, J = 7.8 Hz, 2H), 6.79 (d, J = 8.3 Hz, 1H), 2.98 (dd, J = 4.8, 0.8 Hz, 3H). ^{13}C NMR (100 MHz, $CDCl_3$): δ = 165.4, 155.6, 155.4, 132.4, 132.2, 132.1, 130.1, 124.7, 123.8, 123.4, 119.8, 118.0, 26.65. IR (neat): 3319, 1643, 1537, 1481, 1231, 1152, 879, 803, 749, 678 cm^{-1}. GC-MS (EI): calcd for $C_{14}H_{13}NO_2$ [M$^+$] 227.09, found 227.09.

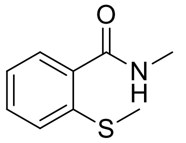

***N*-methyl-2-(methylthio)benzamide (1j)**

The title compound was prepared according to the procedure A as a white solid (93% yield). Melting point: 145–147 °C. ^{1}H NMR (400 MHz, $CDCl_3$): δ = 7.51–7.44 (m, 1H), 7.37–7.29 (m, 1H), 7.28–7.22 (m, 1H), 7.15–7.11 (m, 1H), 6.50 (brs, 1H), 2.98–2.90 (m, 3H), 2.44–2.38 (m, 3H). ^{13}C NMR (100 MHz, $CDCl_3$): δ = 168.7, 168.7, 136.8, 136.8, 135.1, 135.0, 130.4, 128.3, 128.3, 127.0, 126.9, 125.0, 125.0, 26.5, 16.5. IR (neat): 3275, 1626, 1543, 1463, 1326, 1257, 1171, 1069, 968, 756 cm^{-1}. GC-MS (EI): calcd for $C_9H_{11}NOS$ [M$^+$] 181.06., found 181.05.

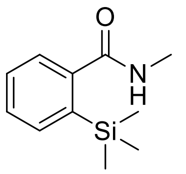

***N*-methyl-2-(trimethylsilyl)benzamide (1k)**

The title compound was prepared according to the procedure A as a white solid (93% yield). Melting point: 98–100 °C. ^{1}H NMR (400 MHz, $CDCl_3$): δ = 7.61 (d, J = 7.4 Hz, 1H), 7.38 (dd, J = 8.3, 7.4 Hz, 2H), 7.35–7.29 (m, 1H), 6.06 (brs, 1H), 2.95–2.89 (m,

3H), 0.30 (s, 9H). ^{13}C NMR (100 MHz, $CDCl_3$): δ = 171.8, 142.3, 139.4, 135.2, 129.2, 128.6, 125.9, 26.7, -0.1. IR (neat): 3064, 1625, 1548, 1317, 1258, 1071, 837, 731 cm^{-1}. GC-MS (EI): calcd for $C_{11}H_{17}NOSi$ [M^+] 207.11., found 207.18.

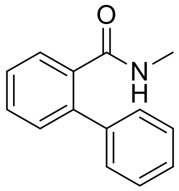

***N*-methyl-[1,1'-biphenyl]-2-carboxamide (1l)**

The title compound was prepared according to the procedure B as a white solid (96% yield). Melting point: 168–170°C. ^{1}H NMR (400 MHz, $CDCl_3$): δ = 7.67 (d, J = 7.3 Hz, 1H), 7.49–7.44 (m, 1H), 7.41–7.36 (m, 7H), 5.24 (brs, 1H), 2.66 (d, J = 4.9 Hz, 3H); ^{13}C NMR (100 MHz, $CDCl_3$): δ = 170.2, 140.0, 139.2, 135.7, 130.0, 129.9, 128.6, 128.5, 128.4, 127.6, 127.4, 26.5. IR (neat): 3308, 1625, 1548, 1473, 1407, 1309, 1155, 1008, 836, 780, 756, 745, 734, 699 cm^{-1}. GC-MS (EI): calcd for $C_{14}H_{13}NO$ [M^+] 211.10, found 211.11. Spectroscopic data are in accordance with those described in the literature.[2]

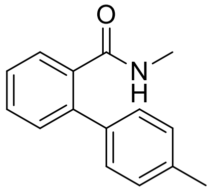

***N*,4'-dimethyl-[1,1'-biphenyl]-2-carboxamide (1m)**

The title compound was prepared according to the procedure A as a white solid (81% yield). Melting point: 138–140 °C. ^{1}H NMR (400 MHz, $CDCl_3$): δ = 7.63 (d, J = 7.5 Hz, 1H), 7.43 (t, J = 7.4 Hz, 1H), 7.34 (dd, J = 11.5, 7.6 Hz, 2H), 7.26 (t, J = 7.3 Hz, 2H), 7.19 (d, J = 7.8 Hz, 2H), 5.32 (brs, 1H), 2.65 (s, 3H), 2.37 (s, 3H). ^{13}C NMR (100 MHz, $CDCl_3$): δ = 170.2, 139.3, 137.4, 137.1, 135.5, 130.1, 130.0, 129.2, 128.7, 128.4, 127.3, 26.4, 21.1. IR (neat): 3311, 1626, 1547, 1475, 1308, 1005, 972, 818, 761 cm^{-1}. GC-MS (EI): calcd for $C_{15}H_{15}NO$ [M^+] 225.12., found 225.11.

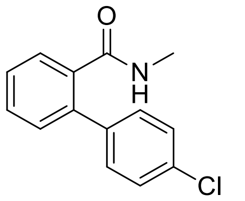

4'-Chloro-*N*-methyl-[1,1'-biphenyl]-2-carboxamide (1n)

The title compound was prepared according to the procedure C as a white solid (85% yield). Melting point: 149–151°C. ^{1}H NMR (400 MHz, CDCl$_3$): δ = 7.63 (d, J = 7.3 Hz, 1H), 7.49–7.46 (m, 1H), 7.43–7.33 (m, 6H), 5.30 (brs, 1H), 2.73 (d, J = 4.6 Hz, 3H); ^{13}C NMR (100 MHz, CDCl$_3$): δ = 170.1, 138.6, 138.1, 135.8, 133.9, 130.2, 130.0, 129.9, 128.7, 128.6, 127.9, 26.7. IR (neat): 3312, 1637, 1578, 1498, 1310, 1092, 833, 792, 762, 677 cm^{-1}. GC-MS (EI): calcd for $C_{14}H_{12}NOCl$ [M$^+$] 245.06, found 245.09.

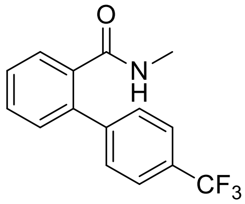

***N*-methyl-4'-(trifluoromethyl)-[1,1'-biphenyl]-2-carboxamide (1o)**

The title compound was prepared according to the procedure C as a white solid (78% yield). Melting point: 112–114 °C. ^{1}H NMR (400 MHz, CDCl$_3$): δ = 7.66 (d, J = 8.1 Hz, 2H), 7.61 (dd, J = 7.5, 1.1 Hz, 1H), 7.55–7.47 (m, 3H), 7.45–7.41 (m, 1H), 7.38–7.33 (m, 1H), 5.43 (brs, 1H), 2.72 (d, J = 4.9 Hz, 3H); ^{13}C NMR (100 MHz, CDCl$_3$): δ = 170.0, 143.8, 130.14, 130.08, 129.7 (q, $J_{C\text{-}F}$ = 32 Hz), 128.9, 128.4, 128.2, 125.3 (q, $J_{C\text{-}F}$ = 4.0Hz), 124.1 (q, $J_{C\text{-}F}$ = 270Hz), 26.6; ^{19}F NMR (377 MHz, CDCl$_3$): δ = -62.5. IR (neat): 3288, 1633, 1567, 1538, 1404, 1322, 1165, 1107, 1068, 959, 880, 840, 765, 738, 698 cm^{-1}. GC-MS (EI): calcd for $C_{15}H_{12}F_3NO$ [M$^+$] 279.09, found 279.11.

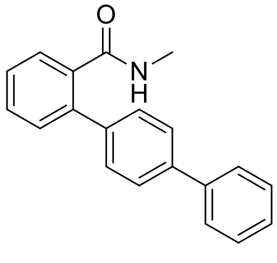

***N*-methyl-[1,1':4',1''-terphenyl]-2-carboxamide (1p)**

The title compound was prepared according to the procedure C as a white solid (60% yield). Melting point: 145–147°C. ^{1}H NMR (400 MHz, $CDCl_3$): δ = 7.62 (s, 5H), 7.50–7.33 (m, 8H), 5.50 (brs, 1H), 2.67 (d, J = 2.9 Hz, 3H); ^{13}C NMR (100 MHz, $CDCl_3$): δ = 170.3, 140.3, 140.2, 138.9, 138.8, 135.7, 130.00, 129.98, 128.9, 128.8, 128.6, 127.5, 127.4, 127.1, 126.9, 26.6. IR (neat): 3370, 1623, 1569, 1403, 1246, 1160, 1003, 845, 756, 699 cm^{-1}. GC-MS (EI): calcd for $C_{20}H_{17}NO$ [M^+] 287.13, found 287.15.

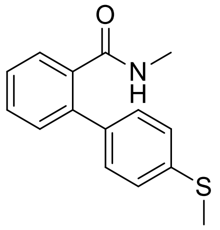

N-methyl-4'-(methylthio)-[1,1'-biphenyl]-2-carboxamide (1q)

The title compound was prepared according to the procedure C as a white solid (78% yield). Melting point: 150–152°C. ^{1}H NMR (400 MHz, $CDCl_3$): δ = 7.58–7.56 (m, 1H), 7.46–7.42 (m, 1H), 7.37–7.24 (m, 6H), 5.56 (brs, 1H), 2.68 (d, J = 4.9 Hz, 3H), 2.50 (s, 3H); ^{13}C NMR (100 MHz, $CDCl_3$): δ = 170.2, 138.5, 138.1, 136.5, 135.6, 129.91, 129.87, 128.8, 128.5, 127.3, 126.1, 26.5, 15.4. IR (neat): 3294, 1743, 1654, 1532, 1474, 1408, 1373, 1244, 1152, 1002, 836, 761, 679 cm^{-1}. GC-MS (EI): calcd for $C_{15}H_{15}NOS$ [M^+] 257.09, found 257.13.

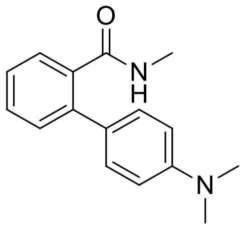

4'-(Dimethylamino)-N-methyl-[1,1'-biphenyl]-2-carboxamide (1r)

The title compound was prepared according to the procedure C as a white solid (77% yield). Melting point: 112–114°C. ^{1}H NMR (400 MHz, $CDCl_3$): δ = 7.74 (d, J = 7.2 Hz, 1H), 7.48–7.43 (m, 1H), 7.40 (t, J = 6.8 Hz, 2H), 7.30–7.25 (m, 2H), 6.74 (d, J = 7.4 Hz, 3H), 5.28 (brs, 1H), 2.97 (s, 6H), 2.67 (d, J = 4.8 Hz, 3H); ^{13}C NMR (100 MHz, $CDCl_3$): δ = 170.3, 150.6, 141.0, 140.3, 135.4, 130.02, 129.97, 129.3, 129.0, 127.4, 116.8, 112.7, 111.9, 40.6, 26.8. IR (neat): 3291, 1633, 1539, 1424, 1309, 1230,

988, 865, 777, 756, 707, 695 cm^{-1}. GC-MS (EI): calcd for $C_{16}H_{18}N_2O$ [M^+] 254.14, found 254.18.

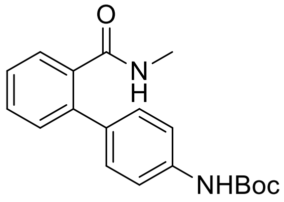

***Tert*-butyl (2'-(methylcarbamoyl)-[1,1'-biphenyl]-4-yl)carbamate (1s)**

The title compound was prepared according to the procedure C as a white solid (82% yield). Melting point: 173–175°C. ^{1}H NMR (400 MHz, DMSO): δ = 9.42 (brs, 1H), 8.01 (brs, 1H), 7.54–7.20 (m, 8H), 2.59 (s, 3H), 1.50 (s, 9H); ^{13}C NMR (100 MHz, DMSO): δ = 170.3, 153.3, 139.2, 139.0, 137.6, 134.3, 130.0, 129.7, 128.9, 128.1, 127.1, 118.3, 79.6, 28.6, 26.4. IR (neat): 3347, 1696, 1647, 1602, 1533, 1365, 1323, 1263, 1159, 1060, 842, 800, 769 cm^{-1}. GC-MS (EI): calcd for $C_{13}H_{18}N_2O_3$ [M^+] 250.13, found 250.15.

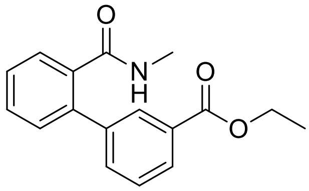

Ethyl 2'-(methylcarbamoyl)-[1,1'-biphenyl]-3-carboxylate (1t)

The title compound was prepared according to the procedure C as a white solid (68% yield). Melting point: 106–108°C. ^{1}H NMR (400 MHz, $CDCl_3$): δ = 8.10 (d, J = 1.6 Hz, 1H), 8.06–8.02 (m, 1H), 7.67–7.58 (m, 2H), 7.52–7.38 (m, 4H), 5.33 (brs, 1H), 4.39 (q, J = 7.1 Hz, 2H), 2.70 (d, J = 4.9 Hz, 3H), 1.40 (t, J = 7.1 Hz, 3H); ^{13}C NMR (100 MHz, $CDCl_3$): δ = 170.1, 166.3, 140.4, 138.4, 135.9, 133.1, 130.9, 130.2, 129.4, 128.8, 128.6, 128.5, 128.0, 61.1, 26.6, 14.3. IR (neat): 3297, 1710, 1634, 1538, 1441, 1305, 1250, 1144, 1081, 937, 778, 754, 690 cm^{-1}. GC-MS (EI): calcd for $C_{17}H_{17}NO_3$ [M^+] 283.12, found 283.14.

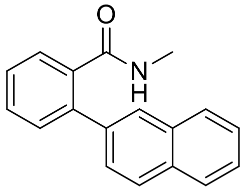

***N*-methyl-2-(naphthalen-2-yl)benzamide (1u)**

The title compound was prepared according to the procedure C as a white solid (74% yield). Melting point: 155–157°C. ^{1}H NMR (400 MHz, CDCl$_3$): δ = 7.92–7.86 (m, 4H), 7.74 (d, J = 7.4 Hz, 1H), 7.54–7.42 (m, 6H), 5.27 (brs, 1H), 2.62 (d, J = 4.9 Hz, 3H); ^{13}C NMR (100 MHz, CDCl$_3$): δ = 170.3, 139.2, 137.7, 135.8, 133.4, 132.6, 130.5, 130.2, 129.0, 128.1, 127.7, 127.4, 126.9, 126.5, 126.4, 26.7. IR (neat): 3273, 1636, 1595, 1321, 1171, 1021, 867, 766, 703 cm^{-1}. GC-MS (EI): calcd for $C_{18}H_{15}NO$ [M$^+$] 261.12, found 261.17

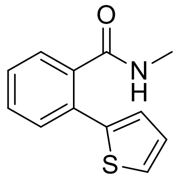

***N*-methyl-2-(thiophen-2-yl)benzamide (1v)**

The title compound was prepared according to the procedure C as a white solid (79% yield). Melting point: 133–135°C. ^{1}H NMR (400 MHz, CDCl$_3$): δ = 7.53 (d, J = 7.3 Hz, 1H), 7.46 (d, J = 7.3 Hz, 1H), 7.41 (t, J = 7.0 Hz, 1H), 7.34 (t, J = 7.2 Hz, 2H), 7.14 (d, J = 2.8 Hz, 1H), 7.05 (dd, J = 4.7, 3.9 Hz, 1H), 5.63 (brs, 1H), 2.77 (d, J = 4.9 Hz, 3H); ^{13}C NMR (100 MHz, CDCl$_3$): δ = 170.2, 141.2, 136.0, 131.6, 130.3, 129.8, 128.4, 127.9, 127.7, 126.6, 126.2, 26.7. IR (neat): 3262, 1628, 1568, 1402, 1268, 1168, 854, 762, 693 cm^{-1}. GC-MS (EI): calcd for $C_{12}H_{11}NOS$ [M$^+$] 217.06, found 217.09. Spectroscopic data are in accordance with those described in the literature.[6]

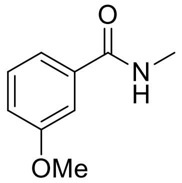

3-Methoxy-*N*-methylbenzamide (1w)

The title compound was prepared according to the procedure B as a white solid (60% yield). Melting point: 68–70°C. ^{1}H NMR (400 MHz, CDCl$_3$): δ = 7.36 (s, 1H), 7.33–7.26 (m, 2H), 7.05–6.97 (m, 1H), 6.47 (brs, 1H), 3.82 (s, 3H), 2.99 (d, J = 4.7 Hz, 3H); ^{13}C NMR (100 MHz, CDCl$_3$): δ = 168.1, 159.7, 136.0, 129.4, 118.6, 117.5, 112.2, 55.3, 26.8. IR (neat): 3289, 1632, 1552, 1284, 1238, 1045, 788, 690 cm^{-1}. GC-MS (EI): calcd for $C_9H_{11}NO_2$ [M$^+$] 165.08, found 165.10. Spectroscopic data are

in accordance with those described in the literature.[3]

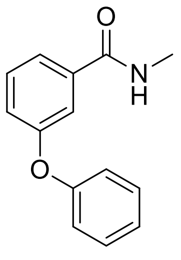

***N*-methyl-3-phenoxybenzamide (1x)**

The title compound was prepared according to the procedure A as a white solid (89% yield). Melting point: 100–102 °C. ^{1}H NMR (400 MHz, CDCl$_3$): δ = 7.47 (d, J = 7.7 Hz, 1H), 7.42 (brs, 1H), 7.32 (t, J = 7.9 Hz, 3H), 7.10 (t, J = 7.7 Hz, 2H), 6.97 (d, J = 8.1 Hz, 2H), 6.70 (s, 1H), 2.92 (d, J = 4.5 Hz, 3H). ^{13}C NMR (100 MHz, CDCl$_3$): δ = 167.6, 157.5, 156.6, 136.4, 129.8, 123.6, 121.4, 119.0, 117.1, 26.7. IR (neat): 3348, 1640, 1541, 1478, 1271, 1233, 1075, 919, 780, 685 cm^{-1}. GC-MS (EI): calcd for $C_{14}H_{13}NO_2$ [M$^+$] 227.09., found 227.10.

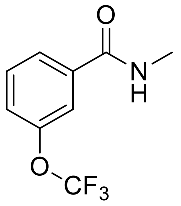

***N*-methyl-3-(trifluoromethoxy)benzamide (1y)**

The title compound was prepared according to the procedure A as a white solid (91% yield). Melting point: 84–86 °C. ^{1}H NMR (400 MHz, CDCl$_3$): δ = 7.71–7.61 (m, 2H), 7.43–7.38 (m, 1H), 7.30 (d, J = 7.4 Hz, 1H), 6.85 (brs, 1H), 2.97 (dd, J = 4.6, 2.0 Hz, 3H). ^{13}C NMR (100 MHz, CDCl$_3$): δ = 166.9 (d, $J_{C\text{-}F}$ = 8 Hz), 149.3, 136.7, 130.0, 125.1 (d, $J_{C\text{-}F}$ = 8 Hz), 123.6, 119.8, 26.8. ^{19}F NMR (377 MHz, CDCl$_3$): δ = -54.6. IR (neat): 3301, 1634, 1557, 1248, 1151, 1001, 903, 812, 757, 700 cm^{-1}. GC-MS (EI): calcd for $C_9H_8NO_2F_3$ [M$^+$] 219.05., found 219.07.

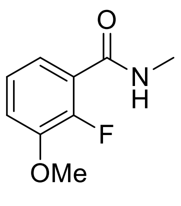

2-Fluoro-3-methoxy-*N*-methylbenzamide (1z)

The title compound was prepared according to the procedure A as a white solid (92% yield). Melting point: 81–83 °C. ^{1}H NMR (400 MHz, $CDCl_3$): δ = 7.62–7.58 (m, 1H), 7.17–7.13 (m, 1H), 7.10–7.05 (m, 1H), 6.71 (brs, 1H), 3.90 (s, 3H), 3.03 (dd, J = 4.8, 0.9 Hz, 3H); ^{13}C NMR (100 MHz, $CDCl_3$): δ = 164.0 (d, $J_{C\text{-}F}$ = 2.3 Hz), 150.7 (d, $J_{C\text{-}F}$ = 246 Hz), 147.8 (d, $J_{C\text{-}F}$ = 12.5 Hz), 124.2 (d, $J_{C\text{-}F}$ = 4.4 Hz), 122.4 (d, $J_{C\text{-}F}$ = 1.2 Hz), 122.1 (d, $J_{C\text{-}F}$ = 9.4 Hz), 116.0 (d, $J_{C\text{-}F}$ = 2.5 Hz), 56.5, 26.8; ^{19}F NMR (377 MHz, $CDCl_3$): δ = –111.3. IR (neat): 3292, 1640, 1480, 1270, 1072, 715 cm^{-1}. GC-MS (EI): calcd for $C_9H_{10}FNO$ [M^+] 183.07, found 183.10. Spectroscopic data are in accordance with those described in the literature.[5]

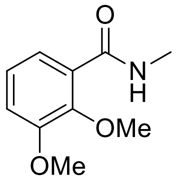

2,3-Dimethoxy-*N*-methylbenzamide (1aa)

The title compound was prepared according to the procedure A as a white solid (87% yield). Melting point: 87–89 °C. ^{1}H NMR (400 MHz, $CDCl_3$): δ = 7.87 (brs, 1H), 7.70–7.46 (m, 1H), 7.15–6.81 (m, 2H), 3.87–3.72 (m, 6H), 3.00–2.83 (m, 3H). ^{13}C NMR (100 MHz, $CDCl_3$): δ = 165.7, 152.3, 147.2, 126.6, 124.1, 122.5, 115.0, 61.0, 55.8, 26.3. IR (neat): 3329, 1651, 1520, 1427, 1260, 1068, 976, 813, 771 cm^{-1}. GC-MS (EI): calcd for $C_{10}H_{13}NO_3$ [M^+] 195.09., found 195.08.

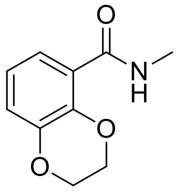

***N*-methyl-2,3-dihydrobenzo[*b*][1,4]dioxine-5-carboxamide (1ab)**

The title compound was prepared according to the procedure A as a white solid (78% yield). Melting point: 101–103 °C. ^{1}H NMR (400 MHz, $CDCl_3$): δ = 7.72 (dd, J = 7.7, 1.6 Hz, 1H), 7.56 (brs, 1H), 7.00–6.93 (m, 1H), 6.90 (t, J = 7.9 Hz, 1H), 4.39 (dd, J = 4.8, 3.1 Hz, 2H), 4.28 (dd, J = 5.2, 2.7 Hz, 2H), 2.98 (d, J = 4.8 Hz, 3H). ^{13}C NMR (101 MHz, $CDCl_3$): δ = 165.4, 143.4, 141.8, 123.9, 123.8, 122.1, 120.4, 64.80, 63.5, 26.4. IR (neat): 3268, 1620, 1470, 1319, 1300, 1105, 917, 821, 770 cm^{-1}. GC-MS (EI):

calcd for $C_{10}H_{11}NO_3$ [M^+] 193.07., found 193.08.

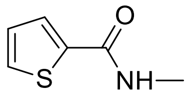

***N*-methylthiophene-2-carboxamide (1ac)**

The title compound was prepared according to the procedure A as a white solid (86% yield). Melting point: 115–117°C. ^{1}H NMR (400 MHz, $CDCl_3$): δ = 7.57–7.51 (m, 1H), 7.43–7.42 (m, 1H), 7.04–7.02 (m, 1H), 6.66 (brs, 1H), 2.96 (d, J = 4.8 Hz, 3H); ^{13}C NMR (100 MHz, $CDCl_3$): δ = 162.8, 139.0, 129.6, 127.9, 127.5, 26.6. IR (neat): 3282, 1609, 1557, 1402, 1309, 1230, 1248, 1149, 1057, 859, 755, 710, 674 cm^{-1}. GC-MS (EI): calcd for C_6H_7NOS [M^+] 141.02, found 141.05. Spectroscopic data are in accordance with those described in the literature.[3]

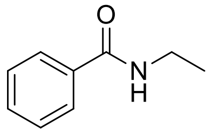

***N*-ethylbenzamide (1ad)**

The title compound was prepared according to the procedure A as a white solid (93% yield). Melting point: 105–107°C. ^{1}H NMR (400 MHz, $CDCl_3$): δ = 7.76 (d, J = 7.2 Hz, 2H), 7.47 (t, J = 7.3 Hz, 1H), 7.40 (t, J = 7.4 Hz, 2H), 6.33 (brs, 1H), 3.51–3.42 (m, 2H), 1.23 (t, J = 7.3 Hz, 3H); ^{13}C NMR (100 MHz, $CDCl_3$): δ = 167.4, 134.7, 131.2, 128.4, 126.8, 34.9, 14.8. IR (neat): 3380, 1629, 1537, 1452, 1349, 1280, 1258, 1189, 1037, 889, 765, 760, 684 cm^{-1}. GC-MS (EI): calcd for $C_9H_{11}NO$ [M^+] 149.08, found 149.10.

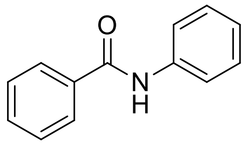

***N*-phenylbenzamide (1ae)**

The title compound was prepared according to the procedure A as a white solid (95% yield). Melting point: 155–157°C. ^{1}H NMR (400 MHz, $CDCl_3$): δ = 7.97 (brs, 1H), 7.86 (d, J = 7.5 Hz, 2H), 7.65 (d, J = 7.9 Hz, 2H), 7.54 (t, J = 7.2 Hz, 1H), 7.46 (t, J = 7.5 Hz, 2H), 7.36 (t, J = 7.8 Hz, 2H), 7.15 (t, J = 7.3 Hz, 1H); ^{13}C NMR (100 MHz,

$CDCl_3$): δ = 165.8, 137.9, 134.9, 131.8, 129.1, 128.7, 127.0, 124.5, 120.2. IR (neat): 3392, 1786, 1723, 1653, 1547, 1432, 1329, 1270, 1268, 1199, 1027, 879, 745, 664 cm^{-1}. GC-MS (EI): calcd for $C_{13}H_{11}NO$ [M^+] 197.08, found 197.10.

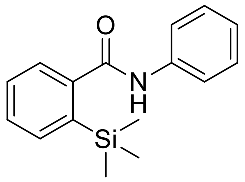

N-phenyl-2-(trimethylsilyl)benzamide (1af)

The title compound was prepared according to the procedure A as a white solid (93% yield). Melting point: 141–143 °C. ^{1}H NMR (400 MHz, $CDCl_3$): δ = 7.71–7.66 (m, 1H), 7.59 (dd, J = 18.9, 7.5 Hz, 3H), 7.53 (brs, 1H), 7.43 (ddd, J = 21.7, 15.1, 7.2 Hz, 4H), 7.16 (t, J = 7.4 Hz, 1H), 0.34 (s, 9H). ^{13}C NMR (100 MHz, $CDCl_3$): δ = 169.2, 139.8, 138.0, 135.6, 129.7, 129.2, 128.9, 126.0, 124.6, 119.9, 0.3. IR (neat): 3179, 1674, 1519, 1439, 1322, 1251, 1135, 912, 840, 753 cm^{-1}. GC-MS (EI): calcd for $C_{16}H_{19}NOSi$ [M^+] 269.12., found 269.13.

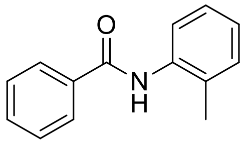

N-(o-tolyl)benzamide (1ag)

The title compound was prepared according to the procedure A as a white solid (81% yield). Melting point: 143–145 °C. ^{1}H NMR (400 MHz, $CDCl_3$): δ = 7.86 (d, J = 7.6 Hz, 3H), 7.80 (brs, 1H), 7.54 (t, J = 7.3 Hz, 1H), 7.46 (t, J = 7.5 Hz, 2H), 7.25–7.19 (m, 2H), 7.11 (t, J = 7.4 Hz, 1H), 2.30 (s, 3H). ^{13}C NMR (100 MHz, $CDCl_3$): δ = 165.7, 135.7, 134.9, 131.7, 130.5, 129.6, 128.7, 127.0, 126.8, 125.4, 123.3, 17.8. IR (neat): 3228, 1646, 1519, 1308, 1287, 1076, 1041, 908, 759, 689 cm^{-1}. GC-MS (EI): calcd for $C_{14}H_{13}NO$ [M^+] 211.10., found 211.10.

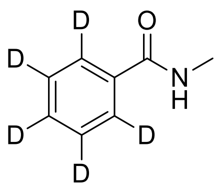

1a–d_5

The title compound was prepared according to the procedure A using aryl-deuteriated benzoyl chlorides (>99% D) as starting material (84% yield). Melting point: 83–85°C. ^{1}H NMR (400 MHz, $CDCl_3$): δ = 6.49 (brs, 1H), 2.98 (d, J = 4.8 Hz, 3H); ^{13}C NMR (100 MHz, $CDCl_3$): δ = 168.3, 134.4, 131.0, 130.8, 130.5, 128.2, 128.0, 127.7, 126.6, 126.4, 126.2, 26.8. IR (neat): 3323, 1632, 1567, 1539, 1407, 1295, 1281, 1156, 842, 670 cm^{-1}. GC-MS (EI): calcd for $C_8H_4D_5NO$ [M^+] 140.10, found 140.12.

Supplementary Table 1. Studying the Effect of Additives on the Cr-Catalyzed *para*-Selective Alkylation of Benzamides with Tertiary Alkylmagnesium Bromide[a]

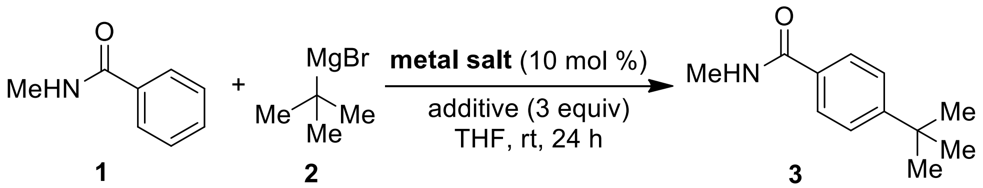

Entry	Metal salt	Additive	Yield (3a)[b]
1	$CrCl_3$	–	nd
2	$CrCl_3$	DCB	nd
3	$CrCl_3$	$AlCl_3$	nd
4	$CrCl_3$	$AlMe_3$	nd
5	$CrCl_3$	Et_3SiCl	trace
6	$CrCl_3$	TMSCl	49%
7	$CrCl_3$	TMSBr	77% (72%)[c]
8	$CrCl_3$	$PhMeSiCl_2$	nd
9	$CrCl_3$	Me_2HSiCl	24%
10	$CrCl_3$	$PhMe_2SiCl$	44%
11	$CrCl_3$	*t*$BuMe_2SiCl$	nd
12	$CrCl_3$	Me_2SiCl_2	17%
13	$CrCl_3$	$Me_2ClSiCH_2Br$	nd
14	$CrCl_3$	Me_3SiH	nd
15	$CrCl_3$	$PhI(OAc)_2$	nd
16	$CrCl_3$	Ag_2O	nd
17	$CrCl_3$	$NaIO_4$	nd
18	$CrCl_3$	CuO	nd
19	$CrCl_3$	TBHP	nd
20	$CrCl_3$	DCP	nd
21	$CrCl_3$	DDQ	nd
22	$CrCl_3$	DTBP	nd

23	$CrCl_3$	AIBN	nd

[a]Conditions: **1** (0.2 mmol), **2** (0.8 mmol), $CrCl_3$ (10 mol %), Additive (3 equiv), THF (0.5 mL), rt, 24 h.

[b]The yield was determined by GC analysis using *n*-tridecane as internal standard. [c]Isolated yield in the parenthesis.

Supplementary Table 2. Investigation of the Effect of the Amount of TMSBr on Cr-Catalyzed *para*-Alkylation of Benzamides with Tertiary Alkylmagnesium Bromides[a]

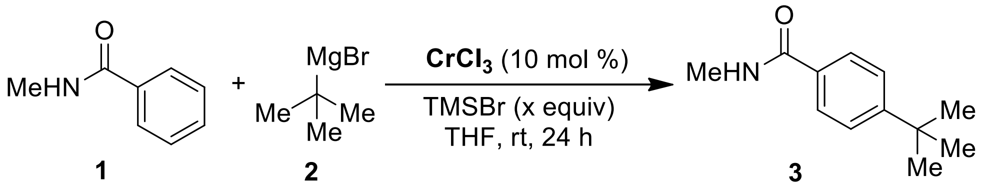

Entry	Metal salt	TMSBr (X equiv)	Yield (3a)[b]
1	$CrCl_3$	0.5 equiv	trace
2	$CrCl_3$	1.0 equiv	8%
3	$CrCl_3$	1.5 equiv	13%
4	$CrCl_3$	2.0 equiv	23%
5	$CrCl_3$	2.5 equiv	41%
2	$CrCl_3$	2.7 equiv	52%
3	$CrCl_3$	3.0 equiv	77%
4	$CrCl_3$	3.2 equiv	73%
5	$CrCl_3$	3.5 equiv	58%
6	$CrCl_3$	4.0 equiv	54%

[a]Conditions: **1** (0.2 mmol), **2** (0.8 mmol), $CrCl_3$ (10 mol %), TMSBr (0.5–4 equiv), THF (0.5 mL), rt, 24 h.

[b]The yield was determined by GC analysis.

Supplementary Table 3. Investigation of the Effect of the Amount of ^{t}BuMgBr on Cr-Catalyzed Coupling of Benzamides with Tertiary Alkylmagnesium Bromides[a]

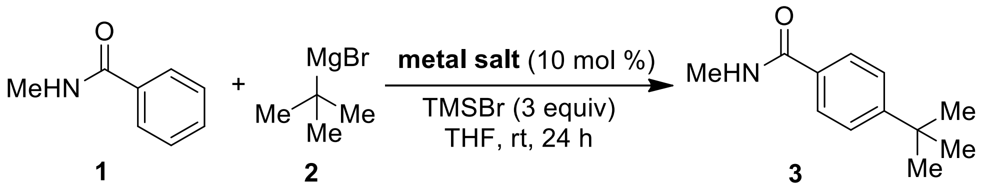

Entry	Metal salt	2a	Yield (3a)[b]
1	$CrCl_3$	2 equiv	4%
2	$CrCl_3$	3 equiv	48%
3	$CrCl_3$	3.5 equiv	62%
4	$CrCl_3$	4 equiv	77%
5	$CrCl_3$	5 equiv	68%

[a]Conditions: **1** (0.2 mmol), **2** (2–5 equiv), $CrCl_3$ (10 mol %), TMSBr (3 equiv), THF (0.5 mL), rt, 24 h.

[b]The yield was determined by GC analysis.

Cr-Catalyzed *para*-Selective Alkylative Reaction of Benzamide Derivatives with Tertiary Organometallic Reagents

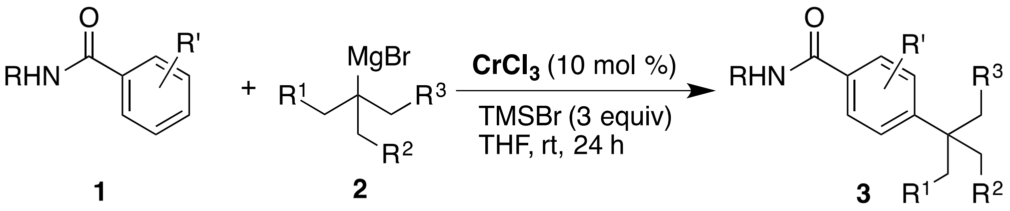

A dried Schlenk tube were placed *N*-methylbenzamide **1** (0.2 mmol), $CrCl_3$ (3 mg, 0.02 mmol) and freshly distilled THF (0.5 mL). Tertiary alkylmagnesium bromide **2** (0.8–1 mmol) was dropwise added by syringe at room temperature. After stirring the mixture for 30 min, trimethylbromosilane (92 mg, 0.6 mmol) was added by syringe and the mixture was stirred at room temperature for 24 h. The resulting mixture was then quenched by an aqueous solution of NH_4Cl and extraction with ethyl acetate (3 x 10 mL). The combined organic phase was dried over anhydrous Na_2SO_4 and concentrated under vacuum. The crude product was purified by silica gel

chromatography to give the *para*-selective alkylated product.

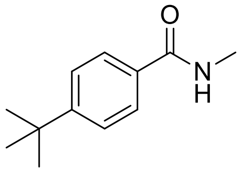

4-(*Tert*-butyl)-*N*-methylbenzamide (3)

The general procedure was applied to *N*-methylbenzamide (27 mg, 0.2 mmol), *tert*-butylmagnesium bromide (1.2 mL, 0.7 M in THF, 0.8 mmol), $CrCl_3$ (3 mg, 0.02 mmol), and trimethylbromosilane (92 mg, 0.6 mmol) at room temperature for 24 h. The crude product was purified by column chromatography on silica gel (EtOAc/PE = 1/5) to afford the title compound as a pale yellow oil (28 mg, 72% yield). ^{1}H NMR (400 MHz, $CDCl_3$): δ = 7.70 (d, J = 8.4 Hz, 2H), 7.41 (d, J = 8.5 Hz, 2H), 6.47 (brs, 1H), 2.98 (d, J = 4.8 Hz, 3H), 1.31 (s, 9H); ^{13}C NMR (100 MHz, $CDCl_3$): δ = 168.2, 154.7, 131.7, 126.7, 125.4, 34.8, 31.1, 26.7. IR (neat): 3324, 2963, 1639, 1551, 1503, 1412, 1311, 1154, 852, 774, 706 cm^{-1}. HRMS (ESI$^+$): calcd for $C_{12}H_{18}NO$ $[M+H]^+$ 192.1388, found 192.1386. Spectroscopic data are in accordance with those described in the literature.[8]

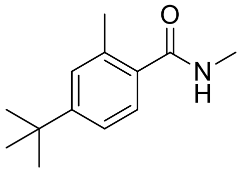

4-(*Tert*-butyl)-*N*,2-dimethylbenzamide (4)

The general procedure was applied to *N*,2-dimethylbenzamide (30 mg, 0.2 mmol), *tert*-butylmagnesium bromide (1.2 mL, 0.7 M in THF, 0.8 mmol), $CrCl_3$ (3 mg, 0.02 mmol), and trimethylbromosilane (92 mg, 0.6 mmol) at room temperature for 24 h. The crude product was purified by column chromatography on silica gel (EtOAc/PE = 1/5) to afford the title compound as a pale yellow oil (23 mg, 56% yield). ^{1}H NMR (400 MHz, $CDCl_3$): δ = 7.28 (d, J = 8.1 Hz, 1H), 7.20–7.18 (m, 2H), 5.87 (brs, 1H), 2.96 (d, J = 4.9 Hz, 3H), 2.44 (s, 3H), 1.30 (s, 9H); ^{13}C NMR (100 MHz, $CDCl_3$): δ = 170.9, 153.0, 135.7, 133.5, 128.0, 126.6, 122.6, 34.6, 31.1, 26.6, 20.1. IR (neat): 3295,

2961, 1539, 1408, 1313, 1295, 1155, 914, 833, 736, 698 cm^{-1}. HRMS (ESI^{+}): calcd for $C_{13}H_{20}NO$ [M+H]$^{+}$ 206.1545, found 206.1538.

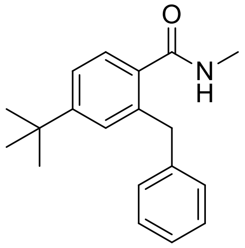

2-Benzyl-4-(*tert*-butyl)-*N*-methylbenzamide (5)

The general procedure was applied to 2-benzyl-*N*-methylbenzamide (46 mg, 0.2 mmol), *tert*-butylmagnesium bromide (1.2 mL, 0.7 M in THF, 0.8 mmol), $CrCl_3$ (3 mg, 0.02 mmol), and trimethylbromosilane (92 mg, 0.6 mmol) at room temperature for 24 h. The crude product was purified by column chromatography on silica gel (EtOAc/PE = 1/4) to afford the title compound as a pale yellow oil (30 mg, 53% yield). ^{1}H NMR (400 MHz, $CDCl_3$): δ = 7.31–7.23 (m, 5H), 7.17 (t, J = 6.3 Hz, 3H), 5.50 (brs, 1H), 4.18 (s, 2H), 2.82 (d, J = 4.9 Hz, 3H), 1.29 (s, 9H). ^{13}C NMR (100 MHz, $CDCl_3$): δ = 170.8, 153.2, 141.1, 138.3, 134.0, 128.8, 128.3, 128.2, 127.0, 126.0, 123.3, 39.3, 34.7, 31.2, 26.5. IR (neat): 3302, 2961, 1636, 1494, 1363, 1181, 1074, 923, 738, 703 cm^{-1}. HRMS (ESI^{+}): calcd for $C_{19}H_{24}NO$ [M+H]$^{+}$ 282.1858, found 282.1851.

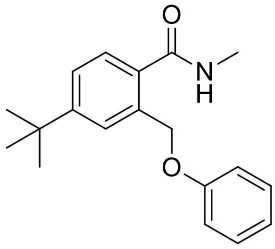

4-(*Tert*-butyl)-*N*-methyl-2-(phenoxymethyl)benzamide (6)

The general procedure was applied to *N*-methyl-2-(phenoxymethyl)benzamide (49 mg, 0.2 mmol), *tert*-butylmagnesium bromide (1.2 mL, 0.7 M in THF, 0.8 mmol), $CrCl_3$ (3 mg, 0.02 mmol), and trimethylbromosilane (92 mg, 0.6 mmol) at room temperature for 24 h. The crude product was purified by column chromatography on silica gel (EtOAc/PE = 1/5) to afford the title compound as a pale yellow oil (41 mg, 68% yield). ^{1}H NMR (400 MHz, $CDCl_3$): δ = 7.57–7.51 (m, 2H), 7.41 (dd, J = 8.1, 1.9 Hz,

1H), 7.31 (dd, J = 8.6, 7.4 Hz, 2H), 7.00 (t, J = 7.9 Hz, 3H), 6.48 (brs, 1H), 5.18 (s, 2H), 2.90 (d, J = 4.9 Hz, 3H), 1.32 (s, 9H). ^{13}C NMR (100 MHz, $CDCl_3$): δ = 169.6, 158.4, 153.8, 133.9, 133.4, 129.6, 128.0, 127.2, 125.5, 121.5, 115.0, 34.9, 31.1, 26. 8. IR (neat): 3306, 2962, 1636, 1598, 1495, 1241, 1172, 1035, 843, 783 cm^{-1}. HRMS (ESI$^+$): calcd for $C_{19}H_{24}NO_2$ [M+H]$^+$ 298.1807, found 298.1795.

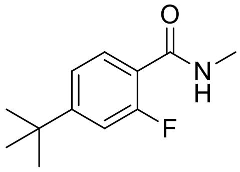

4-(*Tert*-butyl)-2-fluoro-N-methylbenzamide (7)

The general procedure was applied to 2-fluoro-*N*-methylbenzamide (31 mg, 0.2 mmol), *tert*-butylmagnesium bromide (1.2 mL, 0.7 M in THF, 0.8 mmol), $CrCl_3$ (3.2 mg, 0.02 mmol), and trimethylbromosilane (92 mg, 0.6 mmol) at room temperature for 24 h. The crude product was purified by column chromatography on silica gel (EtOAc/PE = 1/5) to afford the title compound as a pale yellow oil (20 mg, 48% yield). ^{1}H NMR (400 MHz, $CDCl_3$): δ = 8.03 (t, J = 8.5 Hz, 1H), 7.26 (dd, J = 7.4, 2.4 Hz, 2H), 7.09 (dd, J = 14.5, 1.5 Hz, 1H), 6.73 (brs,, 1H), 3.02 (d, J = 4.8 Hz, 3H), 1.32 (s, 9H); ^{13}C NMR (100 MHz, $CDCl_3$): δ = 164.0 (d, $J_{C\text{-}F}$ = 3.0 Hz), 160.6 (d, $J_{C\text{-}F}$ = 244 Hz), 157.9 (d, $J_{C\text{-}F}$ = 8 Hz), 131.6 (d, $J_{C\text{-}F}$ = 3 Hz), 121.8 (d, $J_{C\text{-}F}$ = 3 Hz), 117.9 (d, $J_{C\text{-}F}$ = 12 Hz), 112.9 (d, $J_{C\text{-}F}$ = 25 Hz), 35.0 (d, $J_{C\text{-}F}$ = 1 Hz), 31.0, 26.7; ^{19}F NMR (377 MHz, $CDCl_3$): δ = –114.1. IR (neat): 3325, 2963, 1651, 1538, 1410, 1365, 1302, 1196, 1150, 928, 840, 739, 694, 679 cm^{-1}. HRMS (ESI$^+$): calcd for $C_{12}H_{17}FNO$ [M+H]$^+$ 210.1294, found 210.1287.

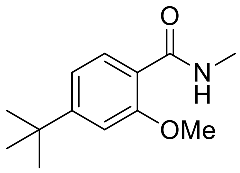

4-(*Tert*-butyl)-2-methoxy-*N*-methylbenzamide (8)

The general procedure was applied to 2-methoxy-*N*-methylbenzamide (33 mg, 0.2 mmol), *tert*-butylmagnesium bromide (1.2 mL, 0.7 M in THF, 0.8 mmol), $CrCl_3$ (3 mg, 0.02 mmol), and trimethylbromosilane (92 mg, 0.6 mmol) at room temperature

for 24 h. The crude product was purified by column chromatography on silica gel (EtOAc/PE = 1/5) to afford the title compound as a pale yellow oil (30 mg, 67% yield). ^{1}H NMR (400 MHz, $CDCl_3$): δ = δ 8.14 (d, J = 8.2 Hz, 1H), 7.81 (brs,, 1H), 7.10 (dd, J = 8.3, 1.6 Hz, 1H), 6.96 (d, J = 1.4 Hz, 1H), 3.97 (s, 3H), 3.00 (d, J = 4.8 Hz, 3H), 1.33 (s, 9H); ^{13}C NMR (100 MHz, $CDCl_3$): δ = 166.0, 157.2, 156.6, 131.9, 118.8, 118.4, 108.3, 55.7, 35.1, 31.1, 26.4. IR (neat): 3416, 2960, 1651, 1610, 1538, 1407, 1292, 1227, 1032, 855, 778, 694 cm^{-1}. HRMS (ESI$^+$): calcd for $C_{13}H_{20}NO_2$ $[M+H]^+$ 222.1494, found 222.1486.

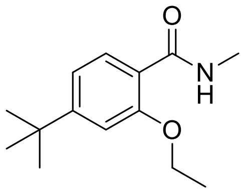

4-(*Tert*-butyl)-2-ethoxy-*N*-methylbenzamide (9)

The general procedure was applied to 2-ethoxy-*N*-methylbenzamide (36 mg, 0.2 mmol), *tert*-butylmagnesium bromide (1.2 mL, 0.7 M in THF, 0.8 mmol), $CrCl_3$ (3 mg, 0.02 mmol), and trimethylbromosilane (92 mg, 0.6 mmol) at room temperature for 24 h. The crude product was purified by column chromatography on silica gel (EtOAc/PE = 1/5) to afford the title compound as a pale yellow oil (27 mg, 58% yield). ^{1}H NMR (400 MHz, $CDCl_3$): δ = 8.13 (d, J = 8.3 Hz, 1H), 7.96 (brs, 1H), 7.08 (d, J = 8.2 Hz, 1H), 6.94 (s, 1H), 4.21 (q, J = 6.9 Hz, 2H), 2.99 (d, J = 4.8 Hz, 3H), 1.50 (t, J = 6.9 Hz, 3H), 1.31 (s, 9H). ^{13}C NMR (100 MHz, $CDCl_3$): δ = 166.1, 156.6, 156.5, 131.8, 118.4, 109.6, 64.6, 35.1, 31.1, 26.3, 14.9. IR (neat): 3418, 2961, 1655, 1543, 1410, 1221, 1040, 859, 778, 738 cm^{-1}. HRMS (ESI$^+$): calcd for $C_{10}H_{14}NO_2$ $[M+H]^+$ 180.1025, found 180.1019.

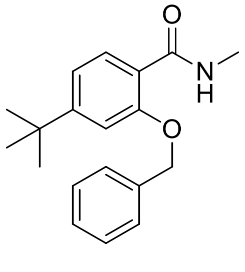

2-(Benzyloxy)-4-(*tert*-butyl)-*N*-methylbenzamide (10)

The general procedure was applied to 2-(benzyloxy)-*N*-methylbenzamide (49 mg, 0.2 mmol), *tert*-butylmagnesium bromide (1.2 mL, 0.7 M in THF, 0.8 mmol), $CrCl_3$ (3 mg, 0.02 mmol), and trimethylbromosilane (92 mg, 0.6 mmol) at room temperature for 24 h. The crude product was purified by column chromatography on silica gel (EtOAc/PE = 1/5) to afford the title compound as a pale yellow oil (30 mg, 49% yield). ^{1}H NMR (400 MHz, $CDCl_3$): δ = 8.15 (d, J = 8.2 Hz, 1H), 7.88 (brs, 1H), 7.46–7.36 (m, 5H), 7.12 (d, J = 8.3 Hz, 1H), 7.02 (s, 1H), 5.20 (s, 2H), 2.89 (d, J = 4.8 Hz, 3H), 1.31 (s, 9H). ^{13}C NMR (100 MHz, $CDCl_3$): δ = 165.9, 156.6, 156.5, 136.0, 132.0, 128.9, 128.6, 127.7, 119.4, 118.9, 110.4, 71.5, 35.1, 31.1, 26.3. IR (neat): 3422, 2922, 2370, 1693, 1659, 1292, 1123, 1014, 934, 844 cm^{-1}. HRMS (ESI$^+$): calcd for $C_{19}H_{23}NO_2Na$ $[M+Na]^+$ 320.1626, found 320.1634.

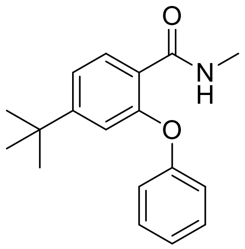

4-(*Tert*-butyl)-*N*-methyl-2-phenoxybenzamide (11)

The general procedure was applied to *N*-methyl-2-phenoxybenzamide (46 mg, 0.2 mmol), *tert*-butylmagnesium bromide (1.2 mL, 0.7 M in THF, 0.8 mmol), $CrCl_3$ (3 mg, 0.02 mmol), and trimethylbromosilane (92 mg, 0.6 mmol) at room temperature for 24 h. The crude product was purified by column chromatography on silica gel (EtOAc/PE = 1/5) to afford the title compound as a pale yellow oil (35 mg, 61% yield). ^{1}H NMR (400 MHz, $CDCl_3$): δ = 8.17 (d, J = 8.3 Hz, 1H), 7.59 (brs, 1H), 7.39 (t, J = 7.9 Hz, 2H), 7.24–7.16 (m, 2H), 7.03 (d, J = 7.9 Hz, 2H), 6.83 (d, J = 1.7 Hz, 1H), 2.96 (d, J = 4.8 Hz, 3H), 1.21 (s, 9H). ^{13}C NMR (100 MHz, $CDCl_3$): δ = 165.5, 156.7, 155.9, 154.9, 131.8, 130.1, 124.3, 121.0, 119.1, 115.8, 35.0, 30.9, 26.6. IR (neat): 3435, 2962, 1695, 1591, 1489, 1409, 1219, 1126, 1090, 942, 755 cm^{-1}. HRMS (ESI$^+$): calcd for $C_{18}H_{22}NO_2$ $[M+H]^+$ 284.1651, found 284.1643.

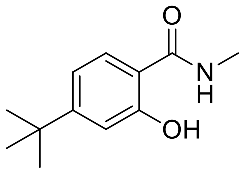

4-(*Tert*-butyl)-2-hydroxy-*N*-methylbenzamide (12)

The general procedure was applied to 2-hydroxy-*N*-methylbenzamide (31 mg, 0.2 mmol), *tert*-butylmagnesium bromide (1.3 mL, 0.7 M in THF, 0.9 mmol), $CrCl_3$ (3 mg, 0.02 mmol), and trimethylbromosilane (92 mg, 0.6 mmol) at room temperature for 24 h. The crude product was purified by column chromatography on silica gel (EtOAc/PE = 1/3) to afford the title compound as a pale yellow oil (12 mg, 28% yield). ^{1}H NMR (400 MHz, $CDCl_3$): δ = 7.28 (s, 2H), 7.03 (d, J = 1.8 Hz, 1H), 6.89 (dd, J = 8.4, 1.9 Hz, 1H), 6.29 (brs, 1H), 3.04 (d, J = 4.8 Hz, 3H), 1.32 (s, 9H). ^{13}C NMR (100 MHz, $CDCl_3$): δ = 170.5, 161.3, 158.3, 124.8, 116.2, 115.4, 111.5, 35.0, 30.9, 26.3. IR (neat): 3374, 2961, 1644, 1602, 1552, 1503, 1366, 1312, 1227, 1023, 938, 814, 742, 703 cm^{-1}. HRMS (ESI^{+}): calcd for $C_{12}H_{18}NO_2$ $[M+H]^{+}$ 208.1338, found 208.1335.

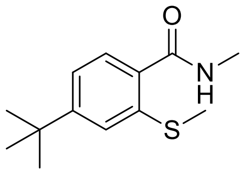

4-(*Tert*-butyl)-*N*-methyl-2-(methylthio)benzamide (13)

The general procedure was applied to *N*-methyl-2-(methylthio)benzamide (37 mg, 0.2 mmol), *tert*-butylmagnesium bromide (1.2 mL, 0.7 M in THF, 0.8 mmol), $CrCl_3$ (3 mg, 0.02 mmol), and trimethylbromosilane (92 mg, 0.6 mmol) at room temperature for 24 h. The crude product was purified by column chromatography on silica gel (EtOAc/PE = 1/5) to afford the title compound as a pale yellow oil (29 mg, 61% yield). ^{1}H NMR (400 MHz, $CDCl_3$): δ = 7.56 (d, J = 8.1 Hz, 1H), 7.36 (d, J = 1.7 Hz, 1H), 7.24 (dd, J = 8.1, 1.8 Hz, 1H), 6.58 (brs, 1H), 3.01 (d, J = 4.9 Hz, 3H), 2.47 (s, 3H), 1.32 (s, 9H). ^{13}C NMR (100 MHz, $CDCl_3$): δ = 168.6, 154.0, 132.7, 130.9, 128.8, 125.6, 123.1, 35.0, 31.1, 26.7, 17.4. IR (neat): 3310, 2960, 1635, 1410, 1260, 1120,

949, 788, 720 cm^{-1}. HRMS (ESI$^+$): calcd for $C_{13}H_{19}NOSNa$ [M+H]$^+$ 260.1085, found 260.1089.

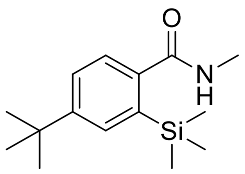

4-(*Tert*-butyl)-*N*-methyl-2-(trimethylsilyl)benzamide (14)

The general procedure was applied to *N*-methyl-2-(trimethylsilyl)benzamide (42 mg, 0.2 mmol), *tert*-butylmagnesium bromide (1.2 mL, 0.7 M in THF, 0.8 mmol), $CrCl_3$ (3 mg, 0.02 mmol), and trimethylbromosilane (92 mg, 0.6 mmol) at room temperature for 24 h. The crude product was purified by column chromatography on silica gel (EtOAc/PE = 1/5) to afford the title compound as a pale yellow oil (28 mg, 52% yield). ^{1}H NMR (400 MHz, $CDCl_3$): δ = 7.67 (s, 1H), 7.36 (s, 2H), 5.90 (brs, 1H), 2.97 (d, J = 4.9 Hz, 3H), 1.32 (s, 9H), 0.32 (s, 9H). ^{13}C NMR (100 MHz, $CDCl_3$): δ = 171.8, 152.1, 139.4, 139.2, 132.3, 125.8, 125.6, 34.8, 31.2, 26.8, 0.1. IR (neat): 3295, 2954, 1640, 1478, 1262, 1118, 878, 841, 765 cm^{-1}. HRMS (ESI$^+$): calcd for $C_{15}H_{26}NOSi$ [M+H]$^+$ 264.1784, found 264.1779.

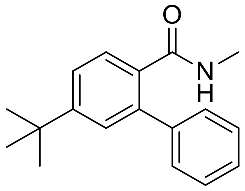

5-(*Tert*-butyl)-*N*-methyl-[1,1'-biphenyl]-2-carboxamide (15)

The general procedure was applied to *N*-methyl-[1,1'-biphenyl]-2-carboxamide (43 mg, 0.2 mmol), *tert*-butylmagnesium bromide (1.2 mL, 0.7 M in THF, 0.8 mmol), $CrCl_3$ (3 mg, 0.02 mmol), and trimethylbromosilane (92 mg, 0.6 mmol) at room temperature for 24 h. The crude product was purified by column chromatography on silica gel (EtOAc/PE = 1/5) to afford the title compound as a pale yellow oil (37 mg, 69% yield). ^{1}H NMR (400 MHz, $CDCl_3$): δ = 7.65 (d, J = 8.1 Hz, 1H), 7.45–7.40 (m, 5H), 7.39–7.34 (m, 2H), 5.16 (brs, 1H), 2.66 (d, J = 4.9 Hz, 3H), 1.35 (s, 9H); ^{13}C NMR (100 MHz, $CDCl_3$): δ = 170.2, 153.4, 140.8, 139.1, 132.8, 128.8, 128.7, 128.6, 127.6, 127.2, 124.7, 34.9, 31.2, 26.6. IR (neat): 3306, 2927, 1624, 1552, 1485, 1407,

1315, 1265, 844, 772, 741, 704 cm^{-1}. HRMS (ESI^{+}): calcd for $C_{18}H_{21}NONa$ [M+Na]$^{+}$ 290.1521, found 290.1511. Spectroscopic data are in accordance with those described in the literature.[7]

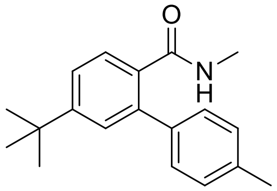

5-(*Tert*-butyl)-*N*,4'-dimethyl-[1,1'-biphenyl]-2-carboxamide (16)

The general procedure was applied to *N*,4'-dimethyl-[1,1'-biphenyl]-2-carboxamide (46 mg, 0.2 mmol), *tert*-butylmagnesium bromide (1.2 mL, 0.7 M in THF, 0.8 mmol), $CrCl_3$ (3 mg, 0.02 mmol), and trimethylbromosilane (92 mg, 0.6 mmol) at room temperature for 24 h. The crude product was purified by column chromatography on silica gel (EtOAc/PE = 1/5) to afford the title compound as a pale yellow oil (18 mg, 31% yield). ^{1}H NMR (400 MHz, $CDCl_3$): δ = 7.65 (d, J = 8.1 Hz, 1H), 7.42 (dd, J = 8.1, 1.9 Hz, 1H), 7.32 (dd, J = 10.7, 4.9 Hz, 3H), 7.23 (d, J = 7.9 Hz, 2H), 5.19 (brs, 1H), 2.69 (d, J = 4.9 Hz, 3H), 2.40 (s, 3H), 1.34 (s, 9H). ^{13}C NMR (100 MHz, $CDCl_3$): δ = 170.3, 153.4, 139.1, 137.9, 137.4, 132.7, 129.3, 128.8, 128.6, 127.2, 124.5, 34.8, 31.2, 26.6, 21.2. IR (neat): 3425, 2954, 1656, 1537, 1409, 1291, 819, 701, 692 cm^{-1}. HRMS (ESI^{+}): calcd for $C_{19}H_{24}NO$ [M+H]$^{+}$ 282.1858, found 282.1862.

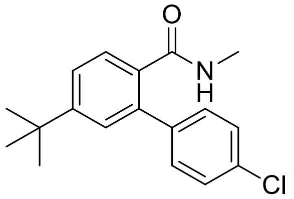

5-(*Tert*-butyl)-4'-chloro-*N*-methyl-[1,1'-biphenyl]-2-carboxamide (17)

The general procedure was applied to 4'-chloro-*N*-methyl-[1,1'-biphenyl]-2-carboxamide (49 mg, 0.2 mmol), *tert*-butylmagnesium bromide (1.2 mL, 0.7 M in THF, 0.8 mmol), $CrCl_3$ (3 mg, 0.02 mmol), and trimethylbromosilane (92 mg, 0.6 mmol) at room temperature for 24 h. The crude product was purified by column chromatography on silica gel (EtOAc/PE = 1/5) to afford the title compound as a pale yellow oil (26 mg, 43% yield). ^{1}H NMR (400 MHz, $CDCl_3$): δ = 7.59 (d, J = 8.1 Hz, 1H), 7.43 (dd, J = 8.1, 1.9 Hz, 1H), 7.37

(td, J = 8.5, 2.0 Hz, 4H), 7.31 (d, J = 1.8 Hz, 1H), 5.27 (brs, 1H), 2.72 (d, J = 4.9 Hz, 3H), 1.34 (s, 9H); ^{13}C NMR (100 MHz, $CDCl_3$): δ = 153.6, 139.2, 137.9, 133.8, 133.0, 130.0, 128.7, 128.6, 127.1, 125.0, 34.9, 31.2, 26.7. IR (neat): 3291, 2962, 1639, 1540, 1485, 1408, 1315, 1248, 1090, 1013, 873, 835, 738, 715 cm^{-1}. HRMS (ESI$^+$): calcd for $C_{18}H_{21}NOCl$ [M+H]$^+$ 302.1312, found 302.1303.

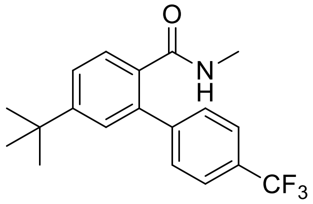

5-(*Tert*-butyl)-*N*-methyl-4'-(trifluoromethyl)-[1,1'-biphenyl]-2-carboxamide (18)

The general procedure was applied to *N*-methyl-4'-(trifluoromethyl)-[1,1'-biphenyl]-2-carboxamide (56 mg, 0.2 mmol), *tert*-butylmagnesium bromide (1.2 mL, 0.7 M in THF, 0.8 mmol), $CrCl_3$ (3 mg, 0.02 mmol), and trimethylbromosilane (92 mg, 0.6 mmol) at room temperature for 24 h. The crude product was purified by column chromatography on silica gel (EtOAc/PE = 1/5) to afford the title compound as a pale yellow oil (44 mg, 65% yield). ^{1}H NMR (400 MHz, $CDCl_3$): δ = 7.67 (d, J = 8.0 Hz, 2H), 7.56 (dd, J = 17.9, 8.1 Hz, 3H), 7.47 (d, J = 8.1 Hz, 1H), 7.35 (s, 1H), 2.73 (d, J = 4.9 Hz, 3H), 1.35 (s, 9H); ^{13}C NMR (100 MHz, $CDCl_3$): δ = 170.0, 153.7, 144.5, 137.8, 133.2, 129.7 (d, $J_{C\text{-}F}$ = 32 Hz), 129.0, 128.4, 127.2, 125.4 (q, $J_{C\text{-}F}$ = 4 Hz), 124.1 (q, $J_{C\text{-}F}$ = 271 Hz), 34.9, 31.2, 26.7; ^{19}F NMR (377 MHz, $CDCl_3$): δ = –62.5. IR (neat): 3302, 2966, 1634, 1548, 1411, 1324, 1264, 1109, 1068, 1017, 848, 735, 703 cm^{-1}. HRMS (ESI$^+$): calcd for $C_{19}H_{20}F_3NONa$ [M+Na]$^+$ 358.1395, found 358.1381.

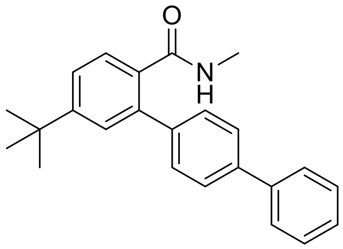

5-(*Tert*-butyl)-*N*-methyl-[1,1':4',1''-terphenyl]-2-carboxamide (19)

The general procedure was applied to *N*-methyl-[1,1':4',1''-terphenyl]-2-carboxamide (57 mg, 0.2 mmol), *tert*-butylmagnesium bromide (1.2 mL, 0.7 M in THF, 0.8 mmol),

$CrCl_3$ (3 mg, 0.02 mmol), and trimethylbromosilane (92 mg, 0.6 mmol) at room temperature for 24 h. The crude product was purified by column chromatography on silica gel (EtOAc/PE = 1/5) to afford the title compound as a pale yellow oil (49 mg, 71% yield). ^{1}H NMR (400 MHz, $CDCl_3$): δ = 7.68–7.64 (m, 5H), 7.51–7.44 (m, 5H), 7.43–7.35 (m, 2H), 5.31 (brs, 1H), 2.71 (d, J = 4.9 Hz, 3H), 1.37 (s, 9H); ^{13}C NMR (100 MHz, $CDCl_3$): δ = 170.4, 153.4, 140.4, 140.3, 139.6, 138.6, 132.8, 129.1, 128.8, 128.7, 127.5, 127.2, 127.0, 124.8, 34.9, 31.2, 26.7. IR (neat): 3291, 2961, 1647, 1537, 1484, 1408, 1312, 1267, 1156, 1007, 843, 768, 740, 698 cm^{-1}. HRMS (ESI$^+$): calcd for $C_{24}H_{26}NO$ [M+H]$^+$ 344.2014, found 344.2007.

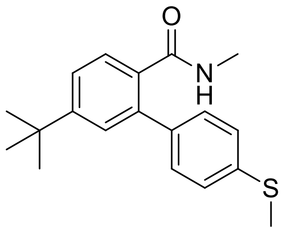

5-(*Tert*-butyl)-*N*-methyl-4'-(methylthio)-[1,1'-biphenyl]-2-carboxamide (20)

The general procedure was applied to *N*-methyl-4'-(methylthio)-[1,1'-biphenyl]-2-carboxamide (52 mg, 0.2 mmol), *tert*-butylmagnesium bromide (1.2 mL, 0.7 M in THF, 0.8 mmol), $CrCl_3$ (3 mg, 0.02 mmol), and trimethylbromosilane (92 mg, 0.6 mmol) at room temperature for 24 h. The crude product was purified by column chromatography on silica gel (EtOAc/PE = 1/5) to afford the title compound as a pale yellow oil (43 mg, 69% yield). ^{1}H NMR (400 MHz, $CDCl_3$): δ = 7.61 (d, J = 8.1 Hz, 1H), 7.42 (dd, J = 8.1, 1.8 Hz, 1H), 7.34 (dd, J = 7.2, 5.2 Hz, 3H), 7.31–7.26 (m, 2H), 5.28 (brs, 1H), 2.71 (d, J = 4.9 Hz, 3H), 2.52 (s, 3H), 1.34 (s, 9H); ^{13}C NMR (100 MHz, $CDCl_3$): δ = 170.3, 153.4, 138.4, 138.2, 137.3, 132.7, 129.1, 128.7, 127.1, 126.3, 124.7, 34.8, 31.2, 26.7, 15.6. IR (neat): 3290, 2961, 1640, 1538, 1484, 1407, 1314, 1092, 1013, 873, 834, 736, 702 cm^{-1}. HRMS (ESI$^+$): calcd for $C_{19}H_{24}NOS$ [M+H]$^+$ 314.1579, found 314.1567.

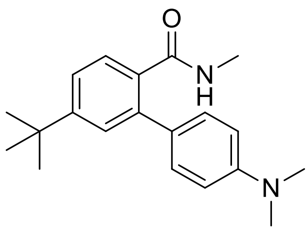

5-(*Tert*-butyl)-4'-(dimethylamino)-*N*-methyl-[1,1'-biphenyl]-2-carboxamide (21)

The general procedure was applied to 3'-(dimethylamino)-*N*-methyl-[1,1'-biphenyl]-2-carboxamide (51 mg, 0.2 mmol), *tert*-butylmagnesium bromide (1.2 mL, 0.7 M in THF, 0.8 mmol), $CrCl_3$ (3 mg, 0.02 mmol), and trimethylbromosilane (92 mg, 0.6 mmol) at room temperature for 24 h. The crude product was purified by column chromatography on silica gel (EtOAc/PE = 1/5) to afford the title compound as a pale yellow oil (30 mg, 48% yield). ^{1}H NMR (400 MHz, $CDCl_3$): δ = 7.71 (d, J = 8.1 Hz, 1H), 7.43 (dd, J = 8.2, 1.7 Hz, 1H), 7.37 (d, J = 1.6 Hz, 1H), 7.28 (dd, J = 13.4, 5.7 Hz, 1H), 6.79–6.69 (m, 3H), 5.30 (brs, 1H), 2.98 (s, 6H), 2.67 (d, J = 4.9 Hz, 3H), 1.34 (s, 9H); ^{13}C NMR (100 MHz, $CDCl_3$): δ = 170.2, 153.3, 150.6, 141.7, 140.0, 132.5, 129.3, 128.9, 127.1, 124.6, 116.9, 112.8, 111.8, 40.56, 34.8, 31.2, 26.7. IR (neat): 3294, 2961, 1651, 1599, 1575, 1487, 1352, 1178, 993, 886, 779, 739, 700 cm^{-1}. HRMS (ESI$^+$): calcd for $C_{20}H_{27}N_2O$ [M+H]$^+$ 311.2123, found 311.2110.

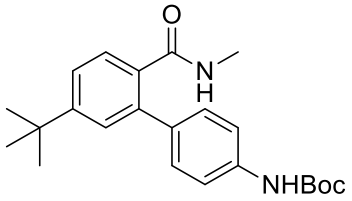

***Tert*-butyl (5'-(tert-butyl)-2'-(methylcarbamoyl)-[1,1'-biphenyl]-4-yl)carbamate (22)**

The general procedure was applied to *tert*-butyl (2'-(methylcarbamoyl)-[1,1'-biphenyl]-4-yl)carbamate (65 mg, 0.2 mmol), *tert*-butylmagnesium bromide (1.2 mL, 0.7 M in THF, 0.8 mmol), $CrCl_3$ (3 mg, 0.02 mmol), and trimethylbromosilane (92 mg, 0.6 mmol) at room temperature for 24 h. The crude product was purified by column chromatography on silica gel (EtOAc/PE = 1/5) to afford the title compound as a pale yellow oil (41 mg, 53% yield). ^{1}H NMR

(400 MHz, DMSO): δ = 9.41 (brs, 1H), 7.96 (q, J = 4.4 Hz, 1H), 7.45 (d, J = 8.5 Hz, 2H), 7.38 (dd, J = 8.0, 1.9 Hz, 1H), 7.32–7.25 (m, 4H), 2.56 (d, J = 4.6 Hz, 3H), 1.49 (s, 9H), 1.31 (s, 9H); ^{13}C NMR (100 MHz, DMSO): δ = 170.4, 153.3, 152.2, 139.1, 138.8, 135.0, 134.8, 129.0, 128.0, 126.8, 124.0, 118.3, 79.5, 34.9, 31.5, 28.6, 26.4. IR (neat): 3416, 2968, 1660, 1543, 1410, 1208, 1178, 938, 824, 761 cm^{-1}. HRMS (ESI$^+$): calcd for $C_{23}H_{30}N_2O_3Na$ [M+Na]$^+$ 405.2154, found 405.2148.

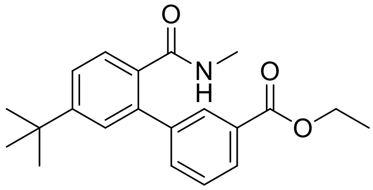

Ethyl 5'-(*tert*-butyl)-2'-(methylcarbamoyl)-[1,1'-biphenyl]-3-carboxylate (23)

The general procedure was applied to ethyl 2'-(methylcarbamoyl)-[1,1'-biphenyl]-3-carboxylate (57 mg, 0.2 mmol), *tert*-butylmagnesium bromide (1.2 mL, 0.7 M in THF, 0.8 mmol), $CrCl_3$ (3 mg, 0.02 mmol), and trimethylbromosilane (92 mg, 0.6 mmol) at room temperature for 24 h. The crude product was purified by column chromatography on silica gel (EtOAc/PE = 1/5) to afford the title compound as a pale yellow oil (31 mg, 45% yield). ^{1}H NMR (400 MHz, $CDCl_3$): δ = 8.10 (s, 1H), 8.04 (d, J = 7.8 Hz, 1H), 7.60 (dd, J = 7.8, 3.7 Hz, 2H), 7.50–7.43 (m, 2H), 7.35 (d, J = 1.6 Hz, 1H), 5.28 (brs, 1H), 4.40 (q, J = 7.1 Hz, 2H), 2.69 (d, J = 4.9 Hz, 3H), 1.40 (t, J = 7.1 Hz, 3H), 1.35 (s, 9H); ^{13}C NMR (100 MHz, $CDCl_3$): δ = 170.1, 166.4, 153.6, 141.1, 138.2, 133.3, 133.1, 130.9, 129.4, 128.7, 128.5, 128.5, 127.2, 125.1, 61.1, 34.9, 31.2, 26.6, 14.3. IR (neat): 3294, 2962, 1717, 1640, 1540, 1409, 1278, 1232, 1110, 1022, 838, 760, 737, 697 cm^{-1}. HRMS (ESI$^+$): calcd for $C_{21}H_{25}NO_3Na$ [M+Na]$^+$ 362.1732, found 362.1716.

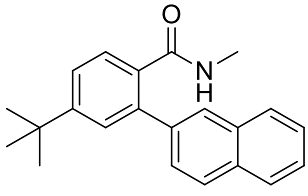

4-(*Tert*-butyl)-*N*-methyl-2-(naphthalen-2-yl)benzamide (24)

The general procedure was applied to *N*-methyl-2-(naphthalen-2-yl)benzamide (52 mg, 0.2 mmol), *tert*-butylmagnesium bromide (1.2 mL, 0.7 M in THF, 0.8 mmol),

$CrCl_3$ (3 mg, 0.02 mmol), and trimethylbromosilane (92 mg, 0.6 mmol) at room temperature for 24 h. The crude product was purified by column chromatography on silica gel (EtOAc/PE = 1/5) to afford the title compound as a pale yellow oil (30 mg, 47% yield). ^{1}H NMR (400 MHz, $CDCl_3$): δ = 7.89 (dd, J = 9.4, 4.8 Hz, 4H), 7.71 (d, J = 8.0 Hz, 1H), 7.54–7.46 (m, 5H), 5.22 (brs, 1H), 2.62 (d, J = 4.9 Hz, 3H), 1.37 (s, 9H); ^{13}C NMR (100 MHz, $CDCl_3$): δ = 170.2, 153.6, 139.0, 138.3, 133.4, 132.9, 132.6, 128.9, 128.1, 128.0, 127.7, 127.5, 127.3, 127.1, 126.5, 126.3, 124.9, 34.9, 31.2, 26.7. IR (neat): 3292, 2962, 1644, 1537, 1505, 1408, 1363, 1314, 1263, 891, 819, 741, 702 cm^{-1}. HRMS (ESI$^+$): calcd for $C_{22}H_{23}NONa$ $[M+Na]^+$ 340.1677, found 340.1665.

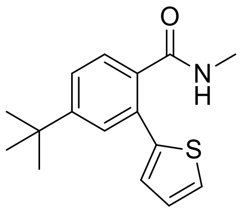

4-(*Tert*-butyl)-*N*-methyl-2-(thiophen-2-yl)benzamide (25)

The general procedure was applied to *N*-methyl-2-(thiophen-2-yl)benzamide (43 mg, 0.2 mmol), *tert*-butylmagnesium bromide (1.2 mL, 0.7 M in THF, 0.8 mmol), $CrCl_3$ (3 mg, 0.02 mmol), and trimethylbromosilane (92 mg, 0.6 mmol) at room temperature for 24 h. The crude product was purified by column chromatography on silica gel (EtOAc/PE = 1/5) to afford the title compound as a pale yellow oil (34 mg, 63% yield). ^{1}H NMR (400 MHz, $CDCl_3$): δ = 7.55 (d, J = 8.1 Hz, 1H), 7.45 (d, J = 1.8 Hz, 1H), 7.41 (dd, J = 8.1, 1.9 Hz, 1H), 7.35 (dd, J = 5.1, 1.0 Hz, 1H), 7.14 (dd, J = 3.5, 1.0 Hz, 1H), 7.07 (dd, J = 5.1, 3.6 Hz, 1H), 5.48 (brs, 1H), 2.79 (d, J = 4.9 Hz, 3H), 1.34 (s, 9H); ^{13}C NMR (100 MHz, $CDCl_3$): δ = 170.2, 153.3, 141.9, 133.3, 131.3, 128.4, 127.7, 127.5, 126.6, 126.1, 125.2, 34.8, 31.1, 26.8. IR (neat):3285, 2958, 1625, 1567, 1409, 1321, 1264, 841, 756, 731, 706 cm^{-1}. HRMS (ESI$^+$): calcd for $C_{16}H_{20}NOS$ $[M+H]^+$ 274.1266, found 274.1260.

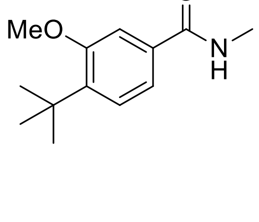

4-(*Tert*-butyl)-3-methoxy-*N*-methylbenzamide (26)

The general procedure was applied to 3-methoxy-*N*-methylbenzamide (33 mg, 0.2 mmol), *tert*-butylmagnesium bromide (1.2 mL, 0.7 M in THF, 0.8 mmol), $CrCl_3$ (3 mg, 0.02 mmol), and trimethylbromosilane (92 mg, 0.6 mmol) at room temperature for 24 h. The crude product was purified by column chromatography on silica gel (EtOAc/PE = 1/5) to afford the title compound as a pale yellow oil (29 mg, 65% yield). ^{1}H NMR (400 MHz, $CDCl_3$): δ = 7.35 (d, J = 1.6 Hz, 1H), 7.24 (d, J = 3.2 Hz, 1H), 7.12 (dd, J = 8.0, 1.7 Hz, 1H), 6.18 (brs, 1H), 3.85 (s, 3H), 2.97 (d, J = 4.9 Hz, 3H), 1.34 (s, 9H); ^{13}C NMR (100 MHz, $CDCl_3$): δ = 168.1, 158.7, 141.9, 133.5, 126.4, 117.7, 110.5, 55.1, 35.0, 29.5, 26.8. IR (neat): 3319, 2961, 1637, 1565, 1497, 1411, 1320, 1239, 1160, 1087, 1024, 871, 790, 764, 742 cm^{-1}. HRMS (ESI$^+$): calcd for $C_{13}H_{20}NO_2$ [M+H]$^+$ 222.1494, found 222.1487.

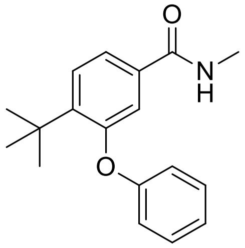

4-(*Tert*-butyl)-*N*-methyl-3-phenoxybenzamide (27)

The general procedure was applied to *N*-methyl-3-phenoxybenzamide (46 mg, 0.2 mmol), *tert*-butylmagnesium bromide (1.2 mL, 0.7 M in THF, 0.8 mmol), $CrCl_3$ (3 mg, 0.02 mmol), and trimethylbromosilane (92 mg, 0.6 mmol) at room temperature for 24 h. The crude product was purified by column chromatography on silica gel (EtOAc/PE = 1/5) to afford the title compound as a pale yellow oil (39 mg, 68% yield). ^{1}H NMR (400 MHz, $CDCl_3$): δ = 7.42 (q, J = 8.2 Hz, 2H), 7.33 (t, J = 7.6 Hz, 2H), 7.21 (s, 1H), 7.09 (t, J = 7.2 Hz, 1H), 6.97 (d, J = 8.0 Hz, 2H), 6.04 (brs, 1H), 2.92 (d, J = 4.7 Hz, 3H), 1.42 (s, 9H). ^{13}C NMR (100 MHz, $CDCl_3$): δ = 167.4, 157.2, 156.1, 144.5, 133.7, 130.0, 127.5, 123.1, 121.3, 118.8, 118.3, 35.0, 29.9, 26.7. IR (neat): 3319, 2957, 1643, 1557, 1489, 1361, 1229, 1079, 931, 838 cm^{-1}. HRMS (ESI$^+$): calcd for $C_{14}H_{13}NO_2$ [M+H]$^+$ 227.0946, found 227.0941.

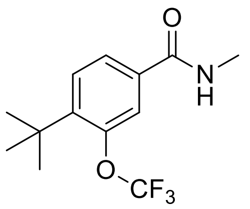

4-(*Tert*-butyl)-*N*-methyl-3-(trifluoromethoxy)benzamide (28)

The general procedure was applied to *N*-methyl-3-(trifluoromethoxy)benzamide (44 mg, 0.2 mmol), *tert*-butylmagnesium bromide (1.2 mL, 0.7 M in THF, 0.8 mmol), $CrCl_3$ (3 mg, 0.02 mmol), and trimethylbromosilane (92 mg, 0.6 mmol) at room temperature for 24 h. The crude product was purified by column chromatography on silica gel (EtOAc/PE = 1/5) to afford the title compound as a pale yellow oil (21 mg, 38% yield). ^{1}H NMR (400 MHz, $CDCl_3$): δ = 7.66 (s, 1H), 7.52 (d, J = 8.2 Hz, 1H), 7.45 (d, J = 8.2 Hz, 1H), 6.17 (s, 1H), 3.01 (d, J = 4.8 Hz, 3H), 1.39 (s, 9H). ^{13}C NMR (100 MHz, $CDCl_3$): δ = 166.7, 148.8, 144.3, 134.1, 128.0, 123.8, 120.6 (q, J_{C-F} = 257 Hz), 117.8 (q, J_{C-F} = 2 Hz), 35.1, 30.0, 26.9. ^{19}F NMR (377 MHz, $CDCl_3$): δ = -54.6. IR (neat): 3312, 2963, 1643, 1577, 1498, 1414, 1289, 1245, 1218, 839, 765 cm^{-1}. HRMS (APCI$^+$): calcd for $C_{13}H_{17}NO_2F_3$ [M+H]$^+$ 276.1211, found 276.1206.

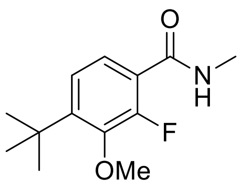

4-(*Tert*-butyl)-2-fluoro-3-methoxy-*N*-methylbenzamide (29)

The general procedure was applied to 2-fluoro-3-methoxy-*N*-methylbenzamide (37 mg, 0.2 mmol), *tert*-butylmagnesium bromide (1.2 mL, 0.7 M in THF, 0.8 mmol), $CrCl_3$ (3 mg, 0.02 mmol), and trimethylbromosilane (92 mg, 0.6 mmol) at room temperature for 24 h. The crude product was purified by column chromatography on silica gel (EtOAc/PE = 1/5) to afford the title compound as a pale yellow oil (17 mg, 35% yield). ^{1}H NMR (400 MHz, $CDCl_3$): δ = 7.66 (t, J = 8.1 Hz, 1H), 7.14 (dd, J = 8.5, 1.0 Hz, 1H), 6.64 (brs, 1H), 3.94 (d, J = 2.2 Hz, 3H), 3.03 (d, J = 4.6 Hz, 3H), 1.37 (s, 9H); ^{13}C NMR (100 MHz, $CDCl_3$): δ = 164.0, 154.9 (d, J_{C-F} = 246 Hz), 148.1 (d, J_{C-F} = 1.9 Hz), 147.0 (d, J_{C-F} = 12.9 Hz), 124.6 (d, J_{C-F} = 2.8 Hz), 121.9 (d, J_{C-F} = 3.1 Hz), 120.4 (d, J_{C-F} = 11.0 Hz), 61.3 (d, J_{C-F} = 8.8 Hz), 35.5 (d, J_{C-F} = 2.2 Hz), 30.1,

26.8; ^{19}F NMR (377 MHz, $CDCl_3$): δ = –131.75. IR (neat): 3304, 2958, 1652, 1537, 1417, 1301, 1243, 1058, 943, 821, 743, 702 cm^{-1}. HRMS (ESI$^+$): calcd for $C_{13}H_{18}FNO_2Na$ [M+Na]$^+$ 262.1219, found 262.1210.

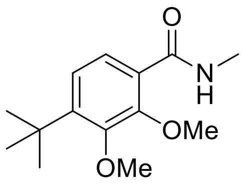

4-(*Tert*-butyl)-2,3-dimethoxy-*N*-methylbenzamide (30)

The general procedure was applied to 2,3-dimethoxy-*N*-methylbenzamide (39 mg, 0.2 mmol), *tert*-butylmagnesium bromide (1.2 mL, 0.7 M in THF, 0.8 mmol), $CrCl_3$ (3 mg, 0.02 mmol), and trimethylbromosilane (92 mg, 0.6 mmol) at room temperature for 24 h. The crude product was purified by column chromatography on silica gel (EtOAc/PE = 1/5) to afford the title compound as a pale yellow oil (20 mg, 40% yield). ^{1}H NMR (400 MHz, $CDCl_3$): δ = 7.90 (brs, 1H), 7.76 (d, J = 8.5 Hz, 1H), 7.13 (d, J = 8.5 Hz, 1H), 3.89 (s, 3H), 3.87 (s, 3H), 3.01 (d, J = 4.8 Hz, 3H), 1.37 (s, 9H). ^{13}C NMR (100 MHz, $CDCl_3$): δ = 165.7, 152.6, 151.9, 147.8, 125.1, 125.0, 122.2, 60.6, 60.2, 35.3, 30.3, 26.5. IR (neat): 3340, 2928, 1659, 1533, 1399, 1284, 1057, 1007, 839, 740 cm^{-1}. HRMS (ESI$^+$): calcd for $C_{14}H_{22}NO_3$ [M+H]$^+$ 252.1600, found 252.1605.

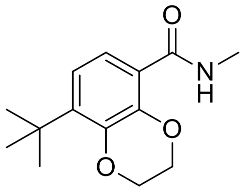

8-(*Tert*-butyl)-*N*-methyl-2,3-dihydrobenzo[b][1,4]dioxine-5-carboxamide (31)

The general procedure was applied to *N*-methyl-2,3-dihydrobenzo[b][1,4]dioxine-5-carboxamide (39 mg, 0.2 mmol), *tert*-butylmagnesium bromide (1.2 mL, 0.7 M in THF, 0.8 mmol), $CrCl_3$ (3 mg, 0.02 mmol), and trimethylbromosilane (92 mg, 0.6 mmol) at room temperature for 24 h. The crude product was purified by column chromatography on silica gel (EtOAc/PE = 1/5) to afford the title compound as a pale yellow oil (24 mg, 48% yield). ^{1}H NMR (400 MHz, $CDCl_3$): δ = 7.67 (d, J = 8.4 Hz, 1H), 7.56 (brs, 1H), 6.95 (d, J = 8.4 Hz,

1H), 4.41 (d, J = 3.5 Hz, 2H), 4.31 (d, J = 2.5 Hz, 2H), 2.99 (d, J = 4.7 Hz, 3H), 1.36 (s, 9H). ^{13}C NMR (100 MHz, $CDCl_3$): δ = 165.7, 142.4, 142.3, 142.1, 123.1, 120.1, 118.7, 64.6, 62.7, 35.1, 29.4, 26.5. IR (neat): 3291, 2923, 1643, 1548, 1426, 1318, 1234, 1090, 954, 740 cm^{-1}. HRMS (ESI$^+$): calcd for $C_{14}H_{20}NO_3$ [M+H]$^+$ 250.1443, found 250.1444.

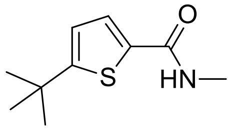

5-(*Tert*-butyl)-*N*-methylthiophene-2-carboxamide (32)

The general procedure was applied to *N*-methylthiophene-2-carboxamide (28 mg, 0.2 mmol), *tert*-butylmagnesium bromide (1.2 mL, 0.7 M in THF, 0.8 mmol), $CrCl_3$ (3 mg, 0.02 mmol), and trimethylbromosilane (92 mg, 0.6 mmol) at room temperature for 24 h. The crude product was purified by column chromatography on silica gel (EtOAc/PE = 1/5) to afford the title compound as a pale yellow oil (17 mg, 42% yield). ^{1}H NMR (400 MHz, $CDCl_3$): δ = 7.31 (d, J = 3.8 Hz, 1H), 6.79 (d, J = 3.8 Hz, 1H), 5.88 (brs, 1H), 2.97 (d, J = 4.9 Hz, 3H), 1.38 (s, 9H). ^{13}C NMR (100 MHz, $CDCl_3$): δ = 162.5, 131.4, 129.9, 127.9, 122.1, 34.9, 32.3, 27.6. IR (neat): 3316, 2961, 1632, 1557, 1524, 1479, 1411, 1334, 1254, 1156, 814, 780, 744, 684 cm^{-1}. HRMS (ESI$^+$): calcd for $C_{10}H_{16}NOS$ [M+H]$^+$ 198.0953, found 198.0945.

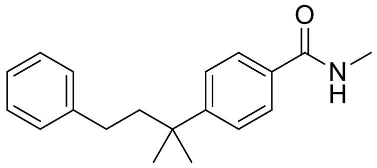

***N*-methyl-4-(2-methyl-4-phenylbutan-2-yl)benzamide (33)**

The general procedure was applied to *N*-methylbenzamide (27 mg, 0.2 mmol), (2-methyl-4-phenylbutan-2-yl)magnesium bromide (5.3 mL, 0.15 M in THF, 0.8 mmol), $CrCl_3$ (3 mg, 0.02 mmol), and trimethylbromosilane (92 mg, 0.6 mmol) at room temperature for 24 h. The crude product was purified by column chromatography on silica gel (EtOAc/PE = 1/5) to afford the title compound as a pale yellow oil (35 mg, 63% yield). ^{1}H NMR (400 MHz, $CDCl_3$): δ = 7.74 (d, J = 8.3 Hz, 2H), 7.43 (d, J = 8.4 Hz, 2H), 7.24 (dd, J = 11.9, 4.6 Hz, 2H), 7.14 (t, J = 7.3 Hz, 1H),

7.06 (d, *J* = 7.1 Hz, 2H), 6.31 (brs, 1H), 3.01 (d, *J* = 4.8 Hz, 3H), 2.35–2.27 (m, 2H), 1.96–1.90 (m, 2H), 1.38 (s, 6H); ^{13}C NMR (100 MHz, $CDCl_3$): δ = 168.2, 152.8, 142.7, 131.9, 128.3, 128.1, 126.7, 126.0, 125.6, 46.53, 38.1, 31.2, 28.8, 26.8. IR (neat): 3324, 2964, 2877, 1640, 1550, 1503, 1462, 1412, 1312, 1164, 852, 770, 739 cm^{-1}. HRMS (ESI$^+$): calcd for $C_{19}H_{23}NONa$ [M+Na]$^+$ 304.1677, found 304.1669.

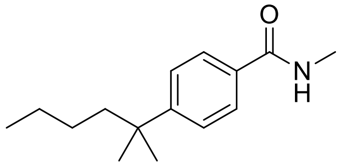

N-methyl-4-(2-methylhexan-2-yl)benzamide (34)

The general procedure was applied to *N*-methylbenzamide (27 mg, 0.2 mmol), (2-methylhexan-2-yl)magnesium bromide (4.0 mL, 0.2 M in THF, 0.8 mmol), $CrCl_3$ (3 mg, 0.02 mmol), and trimethylbromosilane (92 mg, 0.6 mmol) at room temperature for 24 h. The crude product was purified by column chromatography on silica gel (EtOAc/PE = 1/6) to afford the title compound as a pale yellow oil (25 mg, 53% yield). ^{1}H NMR (400 MHz, $CDCl_3$): δ = 7.70 (d, *J* = 8.3 Hz, 2H), 7.34 (d, *J* = 8.3 Hz, 2H), 6.48 (brs, 1H), 2.97 (d, *J* = 4.8 Hz, 3H), 1.58 (dd, *J* = 10.3, 6.4 Hz, 2H), 1.28 (s, 6H), 1.18 (dd, *J* = 14.7, 7.4 Hz, 2H), 1.02–0.93 (m, 2H), 0.79 (t, *J* = 7.3 Hz, 3H). ^{13}C NMR (100 MHz, $CDCl_3$): δ = 168.3, 153.5, 131.6, 126.6, 126.0, 44.1, 37.8, 28.8, 26.8, 26.7, 23.2, 13.9. IR (neat): 3320, 2958, 1639, 1552, 1412, 1311, 1163, 852, 742 cm^{-1}. HRMS (ESI$^+$): calcd for $C_{15}H_{24}NO$ [M+H]$^+$ 234.1858, found 234.1850.

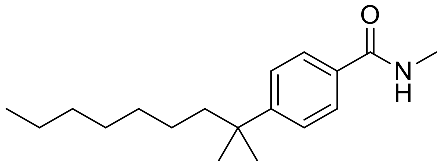

N-methyl-4-(2-methylnonan-2-yl)benzamide (35)

The general procedure was applied to *N*-methylbenzamide (27 mg, 0.2 mmol), (2-methylnonan-2-yl)magnesium bromide (4.0 mL, 0.2 M in THF, 0.8 mmol), $CrCl_3$ (3 mg, 0.02 mmol), and trimethylbromosilane (92 mg, 0.6 mmol) at room temperature for 24 h. The crude product was purified by column chromatography on silica gel (EtOAc/PE = 1/6) to afford the title compound as a pale yellow oil (38 mg, 68%

yield). ^{1}H NMR (400 MHz, CDCl$_3$): δ = 7.69 (d, J = 8.1 Hz, 2H), 7.36 (d, J = 8.1 Hz, 2H), 6.24 (brs, 1H), 2.99 (d, J = 4.8 Hz, 3H), 1.62–1.54 (m, 2H), 1.29 (s, 9H), 1.17 (s, 6H), 1.01–0.97 (m, 1H), 0.84 (t, J = 6.9 Hz, 3H). ^{13}C NMR (100 MHz, CDCl$_3$): δ = 168.3, 153.6, 131.7, 126.6, 126.0, 44.5, 37.9, 31.8, 30.2, 29.2, 28.8, 26.7, 24.7, 22.6, 14.0. IR (neat): 3329, 2927, 1635, 1548, 1412, 1310, 1154, 1022, 851, 738 cm^{-1}. HRMS (ESI$^+$): calcd for $C_{18}H_{30}NO$ [M+H]$^+$ 276.2327, found 276.2321.

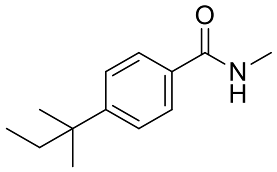

***N*-methyl-4-(*tert*-pentyl)benzamide (36)**

The general procedure was applied to *N*-methylbenzamide (27 mg, 0.2 mmol), *tert*-pentylmagnesium bromide (2.7 mL, 0.3 M in THF, 0.8 mmol), CrCl$_3$ (3 mg, 0.02 mmol), and trimethylbromosilane (92 mg, 0.6 mmol) at room temperature for 24 h. The crude product was purified by column chromatography on silica gel (EtOAc/PE = 1/5) to afford the title compound as a pale yellow oil (23 mg, 57% yield). ^{1}H NMR (400 MHz, CDCl$_3$): δ = 7.69 (d, J = 8.5 Hz, 2H), 7.36 (d, J = 8.5 Hz, 2H), 6.28 (brs, 1H), 2.99 (d, J = 4.9 Hz, 3H), 1.64 (q, J = 7.4 Hz, 2H), 1.28 (s, 6H), 0.65 (t, J = 7.4 Hz, 3H); ^{13}C NMR (100 MHz, CDCl$_3$): δ = 168.2, 153.2, 131.6, 126.5, 126.1, 38.1, 36.7, 28.3, 26.8, 9.0. IR (neat): 3324, 2964, 2877, 1640, 1550, 1503, 1412, 1363, 1312, 1164, 852, 770, 739 cm^{-1}. HRMS (ESI$^+$): calcd for $C_{13}H_{20}NO$ [M+H]$^+$ 206.1545, found 206.1538.

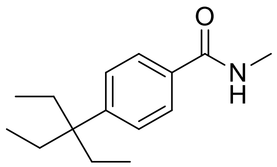

4-(3-Ethylpentan-3-yl)-*N*-methylbenzamide (37)

The general procedure was applied to *N*-methylbenzamide (27 mg, 0.2 mmol), (3-ethylpentan-3-yl)magnesium bromide (4.0 mL, 0.2 M in THF, 0.8 mmol), CrCl$_3$ (3 mg, 0.02 mmol), and trimethylbromosilane (92 mg, 0.6 mmol) at room temperature for 24 h. The crude product was purified by column chromatography on silica gel

(EtOAc/PE = 1/6) to afford the title compound as a pale yellow oil (15 mg, 32% yield). ^{1}H NMR (400 MHz, $CDCl_3$): δ = 7.68 (d, J = 8.5 Hz, 2H), 7.35 (d, J = 8.5 Hz, 2H), 6.14 (brs, 1H), 3.01 (d, J = 4.9 Hz, 3H), 1.68 (q, J = 7.4 Hz, 6H), 0.63 (t, J = 7.4 Hz, 9H). ^{13}C NMR (100 MHz, $CDCl_3$): δ = 168.4, 151.4, 131.5, 127.1, 126.4, 44.0, 28.6, 26.8, 7.9. IR (neat): 3324, 2964, 2877, 1640, 1550, 1503, 1412, 1312, 1164, 852, 770, 739 cm^{-1}. HRMS (ESI^{+}): calcd for $C_{15}H_{24}NO$ [M+H]$^{+}$ 234.1858, found 234.1853.

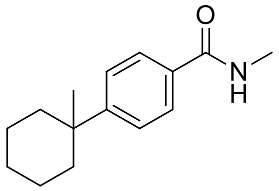

_N_-methyl-4-(1-methylcyclohexyl)benzamide (38)

The general procedure was applied to *N*-methylbenzamide (27 mg, 0.2 mmol), (1-methylcyclohexyl)magnesium chloride (2.7 mL, 0.3 M in THF, 0.8 mmol), $CrCl_3$ (3 mg, 0.02 mmol), and trimethylbromosilane (92 mg, 0.6 mmol) at room temperature for 24 h. The crude product was purified by column chromatography on silica gel (EtOAc/PE = 1/5) to afford the title compound as a pale yellow oil (27 mg, 58% yield). ^{1}H NMR (400 MHz, $CDCl_3$): δ = 7.71 (d, J = 8.4 Hz, 2H), 7.40 (d, J = 8.4 Hz, 2H), 6.42 (brs, 1H), 2.98 (d, J = 4.8 Hz, 3H), 2.03–1.96 (m, 2H), 1.60–1.51 (m, 4H), 1.44–1.34 (m, 4H), 1.16 (s, 3H); ^{13}C NMR (100 MHz, $CDCl_3$): δ = 168.3, 153.6, 131.5, 126.8, 126.1, 38.2, 37.7, 26.7, 26.2, 22.5. IR (neat): 3324, 2964, 2877, 1640, 1550, 1503, 1412, 1312, 1164, 852, 770, 739 cm^{-1}. HRMS (ESI^{+}): calcd for $C_{15}H_{22}NO$ [M+H]$^{+}$ 232.1701, found 232.1693.

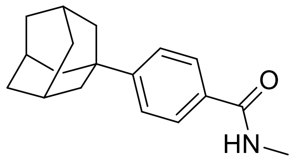

4-(Adamantan-1-yl)-_N_-methylbenzamide (39)

The general procedure was applied to *N*-methylbenzamide (27 mg, 0.2 mmol), adamantan-1-ylmagnesium chloride (5.3 mL, 0.15 M in THF, 0.8 mmol), $CrCl_3$ (3 mg, 0.02 mmol), and trimethylbromosilane (92 mg, 0.6 mmol) at room temperature for 24 h. The crude product was purified by column chromatography on silica gel

(EtOAc/PE = 1/5) to afford the title compound as a pale yellow oil (5 mg, 10% yield). ^{1}H NMR (400 MHz, $CDCl_3$): δ = 7.70 (d, J = 8.5 Hz, 2H), 7.41 (d, J = 8.5 Hz, 2H), 6.16 (brs, 1H), 3.00 (d, J = 4.9 Hz, 3H), 1.91 (d, J = 2.4 Hz, 6H), 1.77 (q, J = 12.2 Hz, 7H), 1.65–1.55 (m, 2H); ^{13}C NMR (100 MHz, $CDCl_3$): δ = 168.2, 154.9, 131.7, 126.6, 125.1, 42.9, 36.6, 36.4, 28.8, 26.8. IR (neat): 3328, 2929, 2858, 1636, 1551, 1505, 1468, 1411, 1317, 851, 772, 711 cm^{-1}. HRMS (ESI^{+}): calcd for $C_{18}H_{24}NO$ [M+H]$^{+}$ 270.1858, found 270.1851.

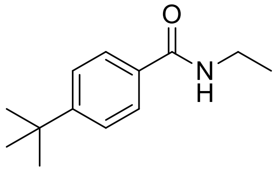

4-(*Tert*-butyl)-N-ethylbenzamide (40)

The general procedure was applied to *N*-ethylbenzamide (30 mg, 0.2 mmol), *tert*-butylmagnesium bromide (1.2 mL, 0.7 M in THF, 0.8 mmol), $CrCl_3$ (3 mg, 0.02 mmol), and trimethylbromosilane (92 mg, 0.6 mmol) at room temperature for 24 h. The crude product was purified by column chromatography on silica gel (EtOAc/PE = 1/5) to afford the title compound as a pale yellow oil (19 mg, 47% yield). ^{1}H NMR (400 MHz, $CDCl_3$): δ = 7.70 (d, J = 8.4 Hz, 2H), 7.43 (d, J = 8.4 Hz, 2H), 6.13 (s, 1H), 3.53–3.44 (m, 2H), 1.32 (s, 9H), 1.24 (t, J = 7.3 Hz, 3H). ^{13}C NMR (100 MHz, $CDCl_3$): δ = 167.3, 154.7, 131.9, 126.6, 125.4, 34.9, 34.8, 31.2, 14.9. IR (neat): 3358, 2939, 2868, 1686, 1541, 1478, 1431, 871, 762, 731 cm^{-1}. HRMS (ESI^{+}): calcd for $C_{13}H_{20}NO$ [M+H]$^{+}$ 206.1545, found 206.1539.

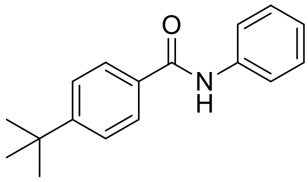

4-(*Tert*-butyl)-N-phenylbenzamide (41)

The general procedure was applied to *N*-phenylbenzamide (39 mg, 0.2 mmol), *tert*-butylmagnesium bromide (1.2 mL, 0.7 M in THF, 0.8 mmol), $CrCl_3$ (3 mg, 0.02 mmol), and trimethylbromosilane (92 mg, 0.6 mmol) at room temperature for 24 h. The crude product was purified by column chromatography on silica gel (EtOAc/PE =

1/5) to afford the title compound as a pale yellow oil (27 mg, 53% yield). ^{1}H NMR (400 MHz, $CDCl_3$): δ = 8.18 (brs, 1H), 7.81 (d, J = 8.3 Hz, 2H), 7.66 (d, J = 7.9 Hz, 2H), 7.44 (d, J = 7.9 Hz, 2H), 7.33 (t, J = 7.5 Hz, 2H), 7.13 (t, J = 7.3 Hz, 1H), 1.34 (s, 9H); ^{13}C NMR (100 MHz, $CDCl_3$): δ = 165.9, 155.2, 138.1, 132.0, 128.9, 126.9, 125.6, 124.3, 120.3, 34.9, 31.1. IR (neat): 3458, 2959, 2878, 1666, 1531, 1478, 1458, 1421, 1356, 1258, 851, 772, 721 cm^{-1}. HRMS (ESI$^+$): calcd for $C_{17}H_{20}NO$ [M+H]$^+$ 254.1545, found 254.1539.

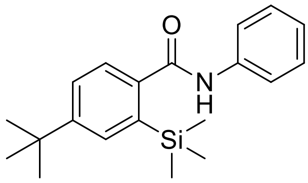

4-(*Tert*-butyl)-*N*-phenyl-2-(trimethylsilyl)benzamide (42)

The general procedure was applied to *N*-phenyl-2-(trimethylsilyl)benzamide (54 mg, 0.2 mmol), *tert*-butylmagnesium bromide (1.2 mL, 0.7 M in THF, 0.8 mmol), $CrCl_3$ (3 mg, 0.02 mmol), and trimethylbromosilane (92 mg, 0.6 mmol) at room temperature for 24 h. The crude product was purified by column chromatography on silica gel (EtOAc/PE = 1/5) to afford the title compound as a pale yellow oil (33 mg, 50% yield). ^{1}H NMR (400 MHz, $CDCl_3$): δ = 7.72 (d, J = 1.8 Hz, 1H), 7.60 (d, J = 8.0 Hz, 2H), 7.56 (brs, 1H), 7.52 (d, J = 8.0 Hz, 1H), 7.45 (dd, J = 8.0, 1.7 Hz, 1H), 7.37 (t, J = 7.8 Hz, 2H), 7.15 (t, J = 7.4 Hz, 1H), 1.36 (s, 9H), 0.35 (s, 9H). ^{13}C NMR (100 MHz, $CDCl_3$): δ = 169.2, 152.6, 139.8, 139.5, 138.1, 132.5, 129.2, 125.9, 125.8, 124.4, 120.0, 34.9, 31.2, 0.2. IR (neat): 3315, 2958, 1673, 1538, 1478, 1360, 1259, 1132, 893, 748 cm^{-1}. HRMS (ESI$^+$): calcd for $C_{20}H_{28}NOSi$ [M+H]$^+$ 326.1940, found 326.1934.

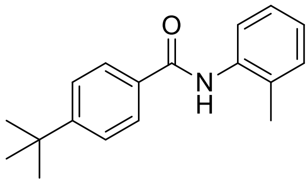

4-(*Tert*-butyl)-*N*-(*o*-tolyl)benzamide (43)

The general procedure was applied to *N*-(*o*-tolyl)benzamide (43 mg, 0.2 mmol), *tert*-butylmagnesium bromide (1.2 mL, 0.7 M in THF, 0.8 mmol), $CrCl_3$ (3 mg, 0.02

mmol), and trimethylbromosilane (92 mg, 0.6 mmol) at room temperature for 24 h. The crude product was purified by column chromatography on silica gel (EtOAc/PE = 1/5) to afford the title compound as a pale yellow oil (32 mg, 59% yield). ^{1}H NMR (400 MHz, $CDCl_3$): δ = 7.94 (d, J = 8.0 Hz, 1H), 7.82 (d, J = 8.3 Hz, 2H), 7.69 (brs, 1H), 7.50 (d, J = 8.3 Hz, 2H), 7.25–7.19 (m, 2H), 7.10 (t, J = 7.4 Hz, 1H), 2.31 (s, 3H), 1.35 (s, 9H). ^{13}C NMR (100 MHz, $CDCl_3$): δ = 165.5, 155.4, 135.9, 132.2, 130.5, 129.1, 126.9, 126.9, 125.8, 125.2, 123.1, 35.0, 31.2, 17.8. IR (neat): 3280, 2962, 1645, 1566, 1505, 1306, 1270, 1127, 854, 747 cm^{-1}. HRMS (ESI$^+$): calcd for $C_{18}H_{21}NONa$ $[M+Na]^+$ 290.1521, found 290.1525.

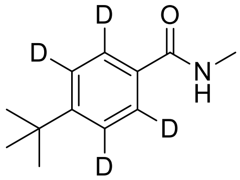

3–*d*4

The general procedure was applied to **1–*d*5** (28 mg, 0.2 mmol), *tert*-butylmagnesium bromide (1.2 mL, 0.7 M in THF, 0.8 mmol), $CrCl_3$ (3 mg, 0.02 mmol), and trimethylbromosilane (92 mg, 0.6 mmol) at room temperature for 24 h. The crude product was purified by column chromatography on silica gel (EtOAc/PE = 1/5) to afford the title compound as a pale yellow oil (20 mg, 53% yield). ^{1}H NMR (400 MHz, $CDCl_3$): δ = 6.38 (brs, 1H), 2.98 (d, J = 4.8 Hz, 3H), 1.31 (s, 9H); ^{13}C NMR (100 MHz, $CDCl_3$): δ = 168.2, 154.6, 131.5, 126.5, 126.2, 126.0, 125.2, 125.0, 124.8, 34.8, 31.1, 27.6. IR (neat): 3330, 2942, 2864, 1542, 1478, 1432, 1325, 888, 792, 758 cm^{-1}. HRMS (ESI$^+$): calcd for $C_{12}H_{14}D_4NO$ $[M+H]^+$ 196.1639, found 196.1637.

The Cr-catalyzed alkylation of benzamides using primary *iso*-butyl and secondary *iso*-propyl Grignard reagents did not furnish the desired *para*-alkylated products, but forming the corresponding *para*-acylated and silylated compounds was observed in these cases, respectively. However, the reaction with other alkylmagnesium bromides such as methyl-, allyl- and benzyl-substituted Grignard reagents did not give the alkylated products in the recovery of starting benzamide compounds. Meanwhile, the

use of non-substituted benzamide(**1ah**), aromatic ketone (**1ai**), benzoic acid (**1aj**), amide derivatives that contains other aromatic rings such as naphthalene, pyridine, pyrrole and furan (**1ak**–**1am**) in the reaction did not form the alkylated products.

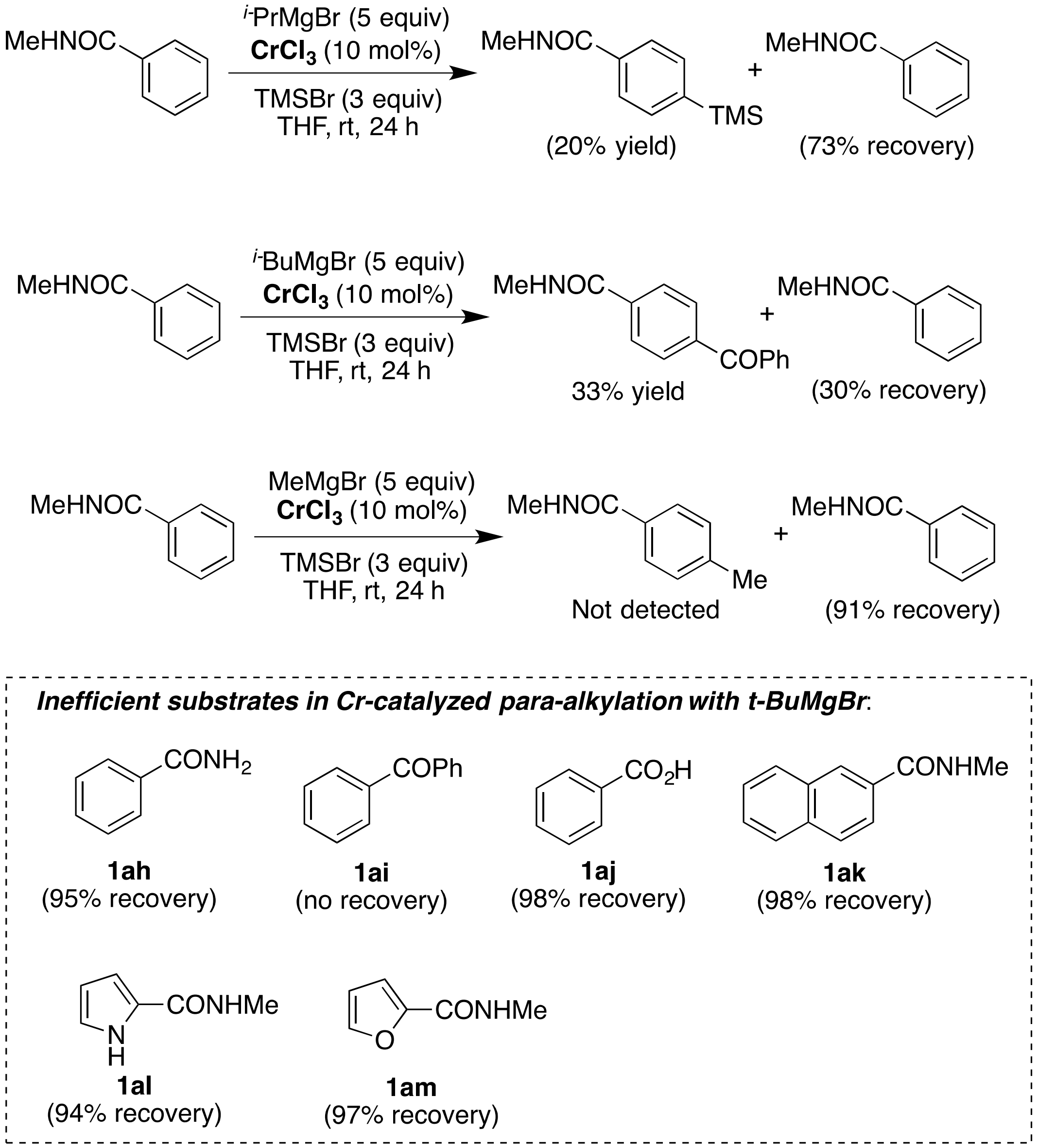

Supplementary Figure 1. Inefficient Substrates in the Cr-Catalyzed Alkylation.

Kinetic Studies

Experiments of Kinetic Isotope Effect

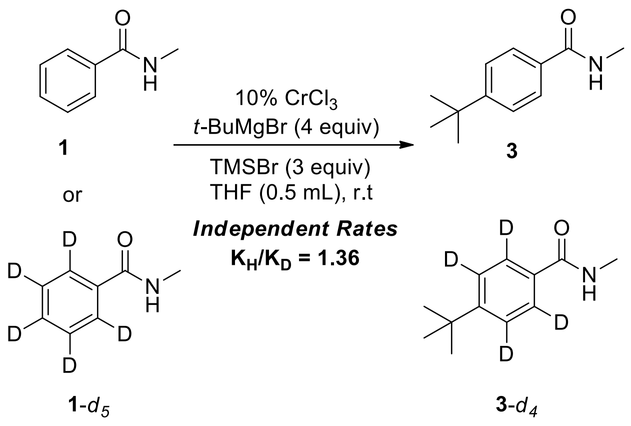

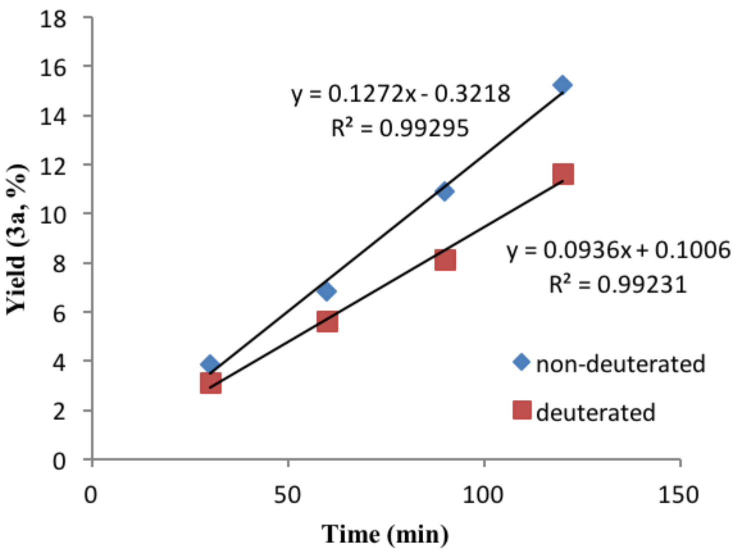

Supplementary Figure 2. KIE experiments.

A dried Schlenk tube were placed *N*-methylbenzamide **1** (27 mg, 0.2 mmol) or **1-*d*$_5$** (28 mg, 0.2 mmol) and $CrCl_3$ (3 mg, 0.02 mmol) and freshly distilled THF (0.5 mL). *Tert*-butylmagnesium bromide **2** (1.2 mL, 0.8 mmol) was dropwise added by syringe at room temperature. After stirring the mixture for 30 min, trimethylbromosilane (92 mg, 0.6 mmol) was added by syringe and reacted then reacted for the designated time (30 min, 60 min, 90 min, 120 min) at room temperature. Then, the reaction mixture quenched with aqueous solution of NH_4Cl. The yield was determined by GC analysis. A value of K_H/K_D = 1.36 was obtained.

Procedure for determining the order in $CrCl_3$:

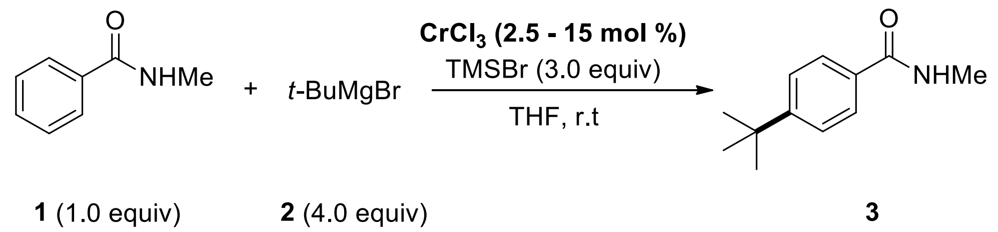

N-methylbenzamide **1** (0.500 mmol) and different amounts of $CrCl_3$ (0.0125, 0.0250, 0.0375, 0.050, 0.0625, 0.0750 mmol) were placed in a dried Schlenk tube, followed by the addition of *n*-tridecane (0.25 mmol, an internal standard for GC analysis) and freshly distilled THF (1.4 mL). Then, *tert*-butylmagnesium bromide **2** (2.8 mL, 2.0 mmol, 0.7 M in THF) was dropwise added by syringe at room temperature. After stirring the mixture for 30 min, trimethylbromosilane (230 mg, 1.5 mmol) was added by syringe. Periodic aliquots (30 μL) were removed by a syringe and quenched by an aqueous solution of NH_4Cl. The resulting mixture was extracted with ethyl acetate (1.0 mL) and the organic phase was analyzed by GC using *n*-tridecane as internal standard. The concentrations of the products were plotted to yield the initial rates for the formation of **3** (with a maximum of 25% conversion).

Supplementary Table 4. Initial Rate Data Obtained by Variation of the Concentration of $CrCl_3$

Entry	mol %	$CrCl_3$ [M]	Initial rate [M/min]
1	2.5	**0.002907**	1.06×10^{-4}
2	5	**0.005814**	2.96×10^{-4}
3	7.5	**0.008721**	4.24×10^{-4}
4	10	**0.011628**	4.78×10^{-4}
5	12.5	**0.014535**	6.84×10^{-4}
6	15	**0.017442**	8.11×10^{-4}

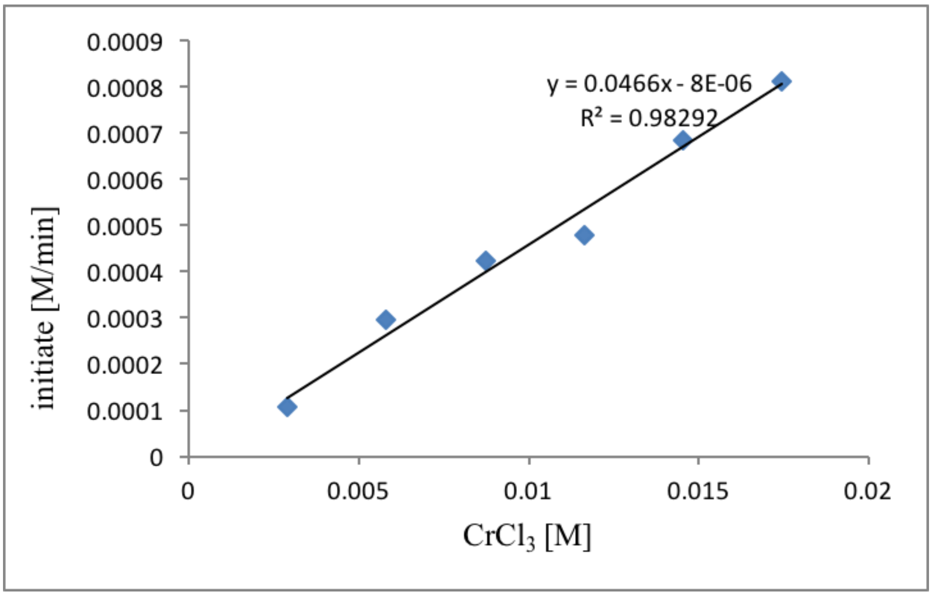

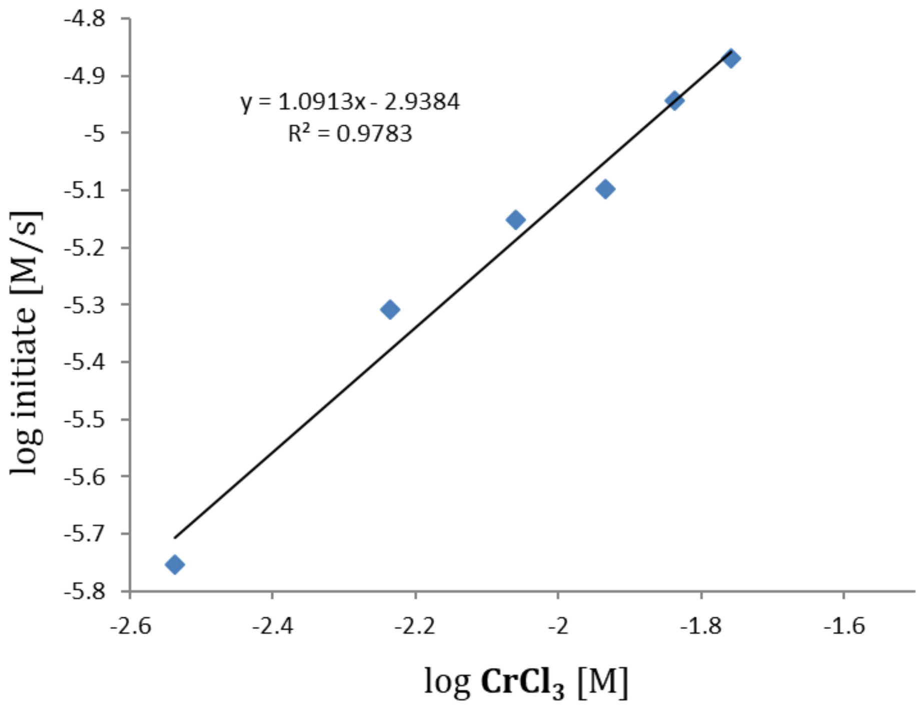

Supplementary Figure 3. Plot of initial rates versus the concentration of $CrCl_3$.

Procedure for determining the order in N-methylbenzamide 1:

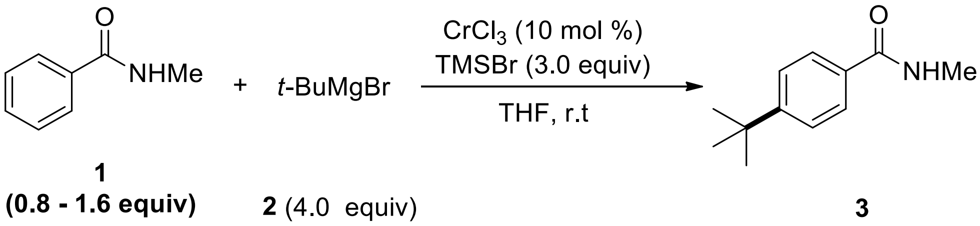

Different amounts of *N*-methylbenzamide **1** (0.40, 0.50, 0.60, 0.70, 0.80 mmol) and $CrCl_3$ (0.05 mmol) were placed in a dried Schlenk tube, followed by the addition of *n*-tridecane (0.25 mmol, an internal standard for GC analysis) and freshly distilled THF (1.4 mL). Then, *tert*-butylmagnesium bromide **2** (2.8 mL, 0.7 M in THF) was dropwise added by syringe at room temperature. After stirring the mixture for 30 min, trimethylbromosilane (230 mg, 1.5 mmol) was added by syringe. Periodic aliquots (30 μL) were removed by a syringe and quenched by an aqueous solution of NH_4Cl. The resulting mixture was extracted with ethyl acetate (1.0 mL) and the organic phase was analyzed by GC using *n*-tridecane as internal standard. The concentrations of the products were plotted to yield the initial rates for the formation of **3** (with a maximum of 25% conversion).

Supplementary Table 5. Initial Rate Data Obtained by Variation of the Concentration of 1

Entry	amide **1** [M]	^{t}BuMgBr **2** [M]	Initial rate [M/min]
1	**0.0930**	0.4761	4.41×10^{-4}
2	**0.1163**	0.4761	4.78×10^{-4}
3	**0.1395**	0.4761	5.81×10^{-4}
4	**0.1628**	0.4761	6.31×10^{-4}
5	**0.1860**	0.4761	6.83×10^{-4}

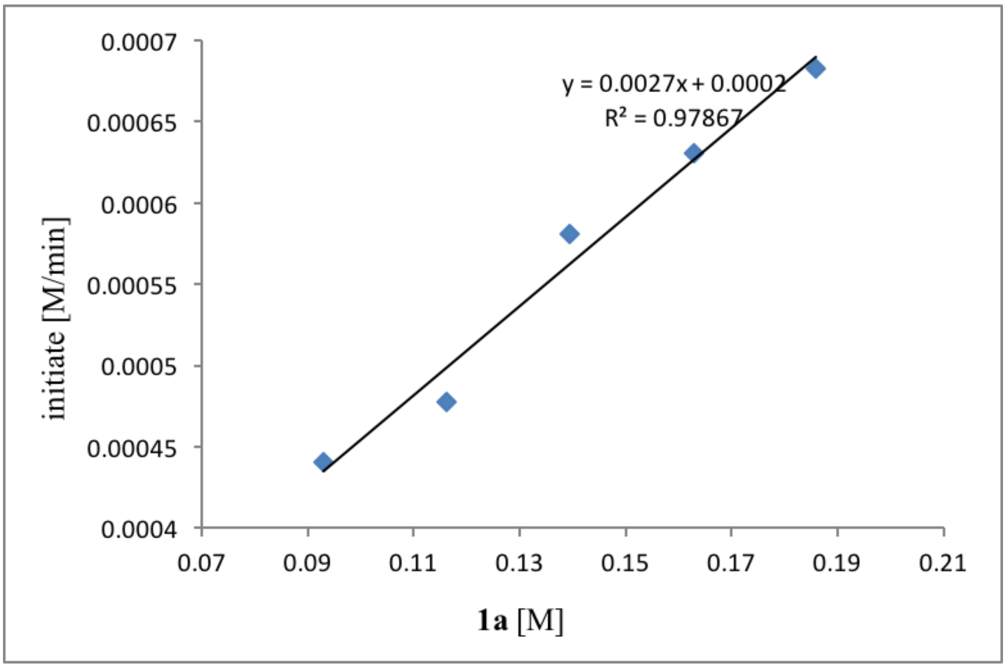

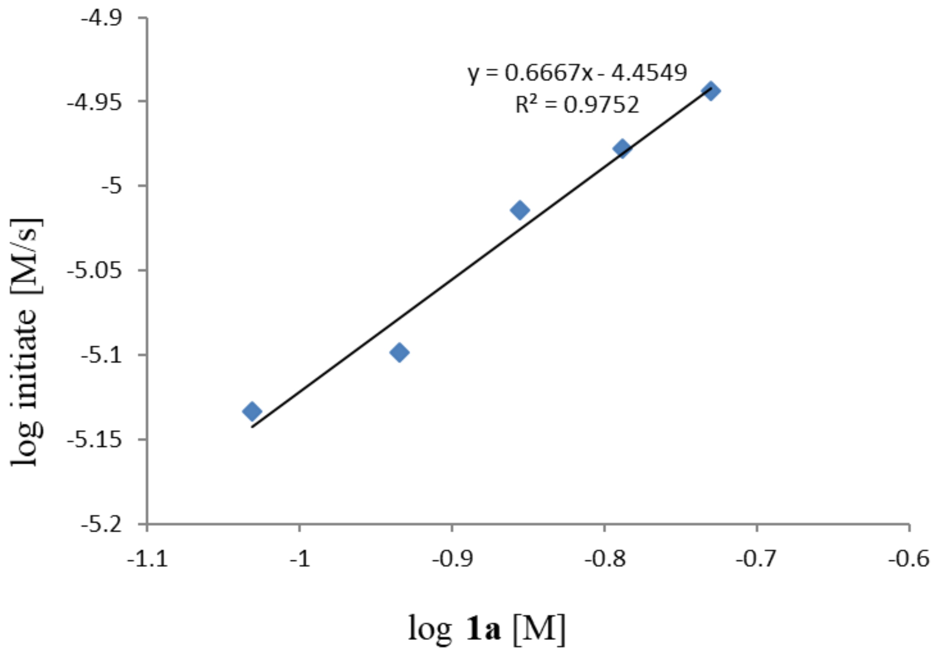

Supplementary Figure 4. Plot of initial rates versus the concentration of benzamide (1).

Stoichiometric reaction of $CrCl_3$ with tert-butylmagnesium bromide

A dried Schlenk tube (25 mL) containing $CrCl_3$ (16 mg, 0.1 mmol) was added freshly distilled THF (2 mL) under nitrogen atmosphere. *Tert*-butylmagnesium bromide (5.7 mL, 4 mmol) was added by syringe at room temperature. The gas of hydrogen was

determined by GC-MS, and the evolution amounts of hydrogen was analyzed by GC at related reaction time of 1, 8, 15, 24, 40, 79, 109, 139, 199, 229 and 259 min. The corresponding yield of hydrogen was 0.02834, 0.07842, 0.08598, 0.08725, 0.08859, 0.09008, 0.09201, 0.09582, 0.10001, 0.10002, 0.09892 mmol, respectively. Please see the following reaction profile for details.

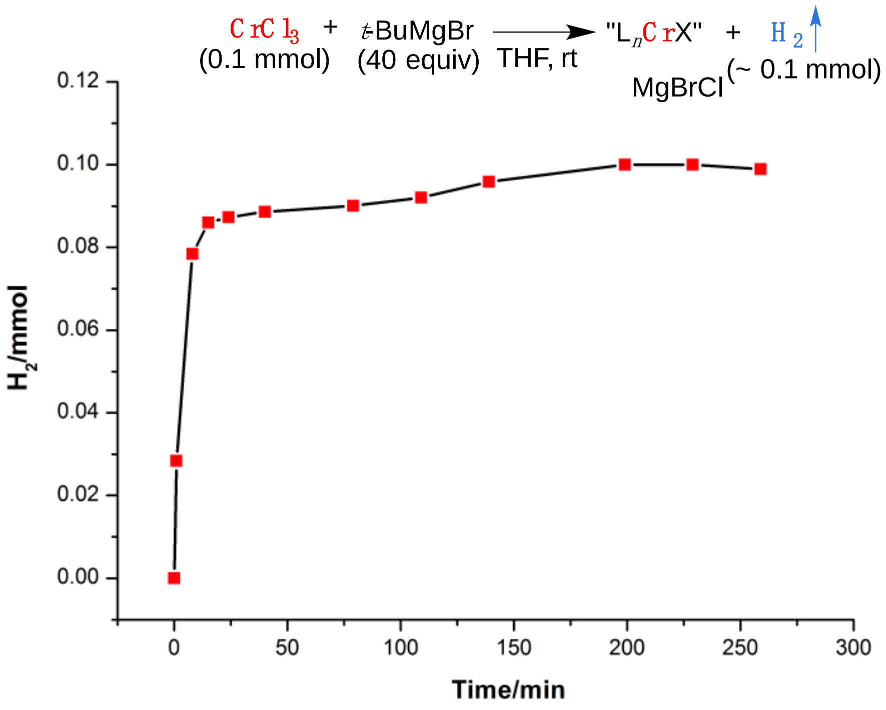

Supplementary Figure 5. Reaction profile for stoichiometric reaction of $CrCl_3$ with *tert*-butylmagnesium bromide.

Experiments for other mechanistic studies

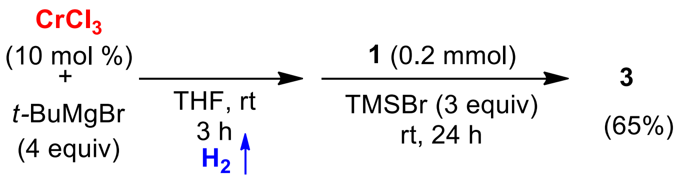

A dried Schlenk tube were placed $CrCl_3$ (3 mg, 0.02 mmol), *tert*-butylmagnesium bromide (1.2 mL, 0.8 mmol) and freshly distilled THF (0.5 mL). After stirring the mixture for 3 h at room temperature, *N*-methylbenzamide **1** (27 mg, 0.2 mmol) and trimethylbromosilane (92 mg, 0.6 mmol) was added by syringe and reacted at room

temperature for 24 h. The resulting mixture was quenched by an aqueous solution of NH_4Cl and extraction with ethyl acetate (3 x 10 mL). The combined organic phase was dried over anhydrous Na_2SO_4 and concentrated under vacuum. The crude product was purified by silica gel chromatography to give the desired coupling product **3** in 65% yield

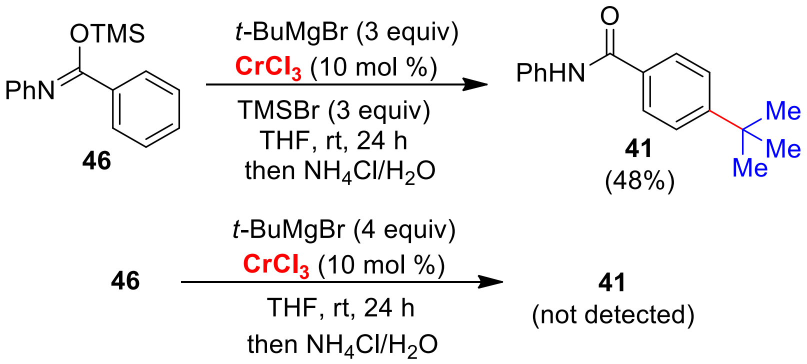

Supplementary Figure 6. Cr-Catalyzed *para*-Alkylation with or without TMSBr

A dried Schlenk tube were placed trimethylsilyl *N*-phenylbenzimidate 4**6** (54 mg, 0.2 mmol), $CrCl_3$ (3 mg, 0.02 mmol) and freshly distilled THF (0.5 mL). *Tert*-butylmagnesium bromide (1.2 mL, 0.8 mmol) was dropwise added by syringe at room temperature. After stirring the mixture for 30 min, trimethylbromosilane (92 mg, 0.6 mmol) was added by syringe and reacted at room temperature for 24 h. The resulting mixture was quenched by an aqueous solution of NH_4Cl and extraction with ethyl acetate (3 x 10 mL). The combined organic phase was dried over anhydrous Na_2SO_4 and concentrated under vacuum. The crude product was purified by silica gel chromatography to give the desired coupling product **41** in 48% yield.

It should be noted that the reaction of **6** with 1 equivalent of *t*-BuMgBr cannot give the para-alkylated product. When increasing the amount of alkyl Grignard reagent to 2 equivalents, the formation of the desired product **41** in 13% yield was observed. In the absence of $MgBr_2$, the *para*-alkylation with *t*-BuMgBr also proceeded effectively.

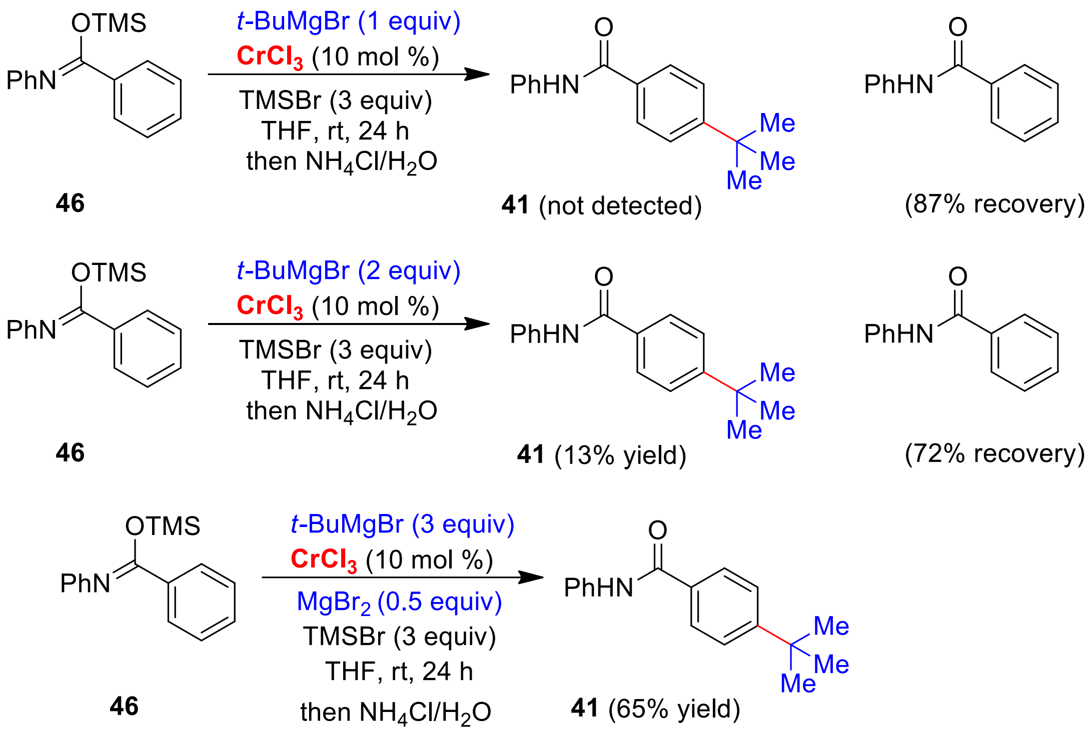

Supplementary Figure 7. The Effect of *tert*-Butyl Grignard on the Cr-Catalyzed *para*-Alkylation.

A dried Schlenk tube were placed *N*-methylbenzamide **1** (27 mg, 0.2 mmol), $CrCl_3$ (3 mg, 0.02 mmol), TEMPO (94 mg, 3 equiv) and freshly distilled THF (0.5 mL). *Tert*-butylmagnesium bromide (1.2 mL, 0.8 mmol) was dropwise added by syringe at room temperature. After stirring the mixture for 30 min, trimethylbromosilane (92 mg, 0.6 mmol) was added by syringe and reacted at room temperature for 24 h. The resulting mixture was quenched by an aqueous solution of NH_4Cl and extraction with ethyl acetate (3 x 10 mL). The desired product **3** was not detected by GC/MS and TLC analysis, and nearly 92% of benzamide **1** and 54% of TEMPO were recovered by GC/MS analyses. The attempt to isolated the compound formed by trapping *t*-butyl radical with TEMPO was failed.

In addition, the Cr-catalyzed *para*-alkylation with other radical scavengers such as SmI_2, Bu_3SnH and DHA cannot form the desired *para*-alkylated products.

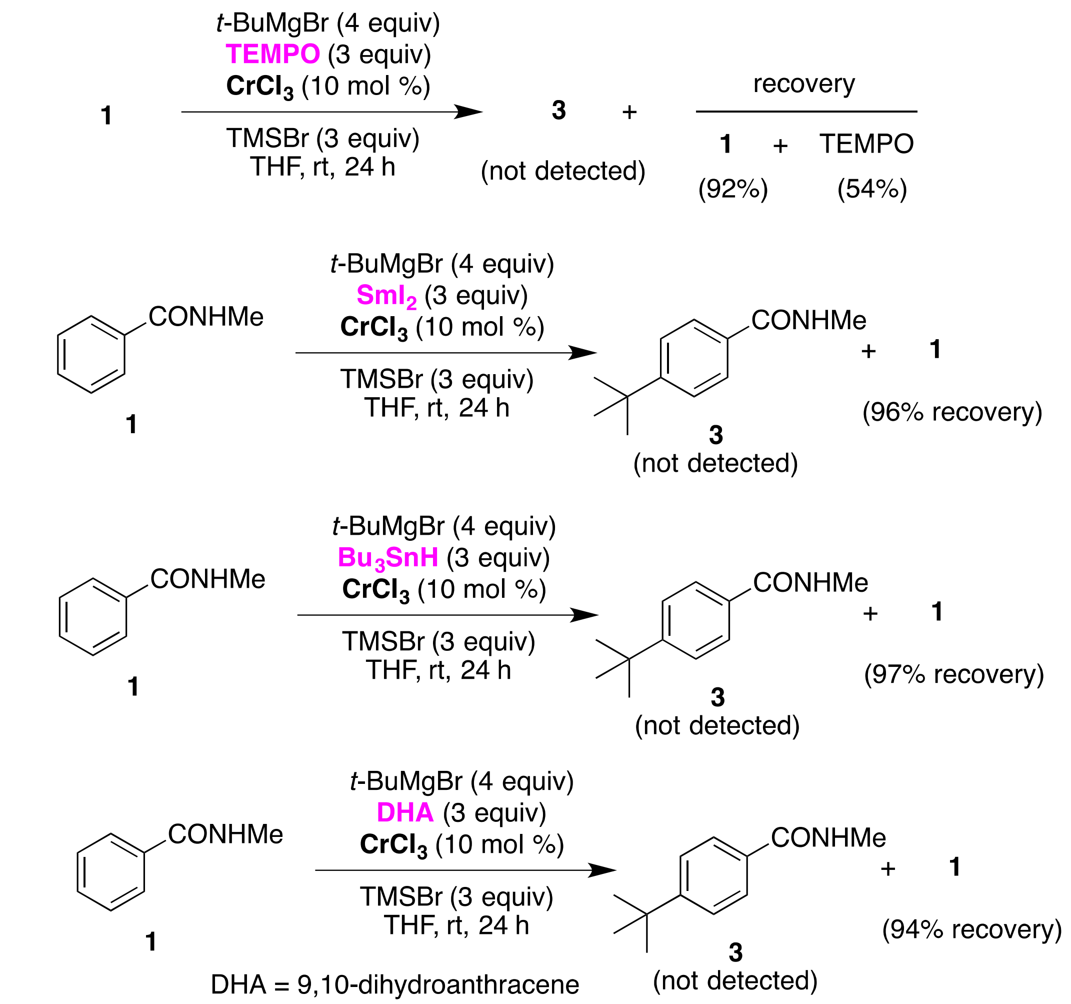

Supplementary Figure 8. Radical Scavenger Experiments.

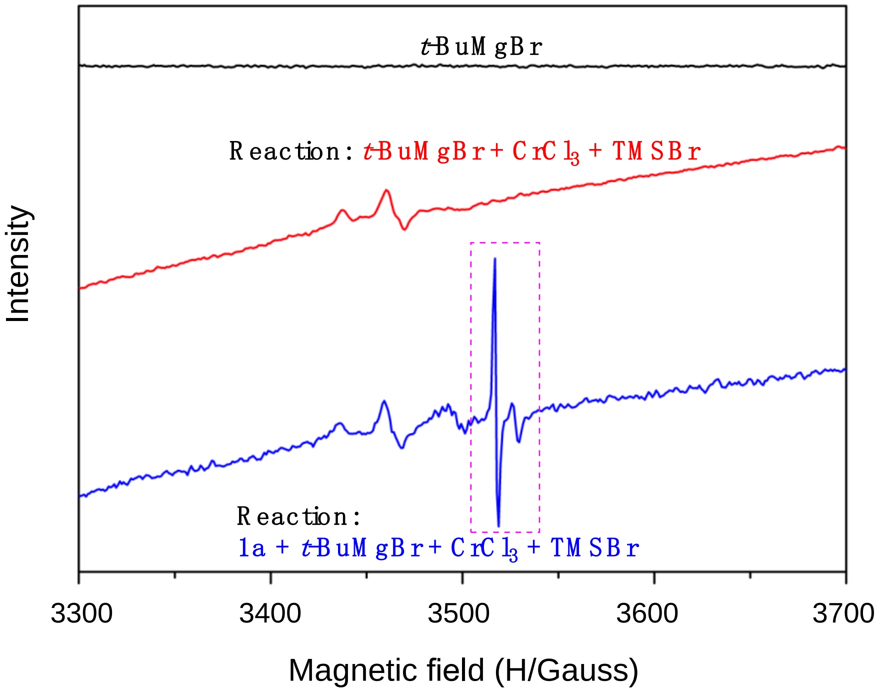

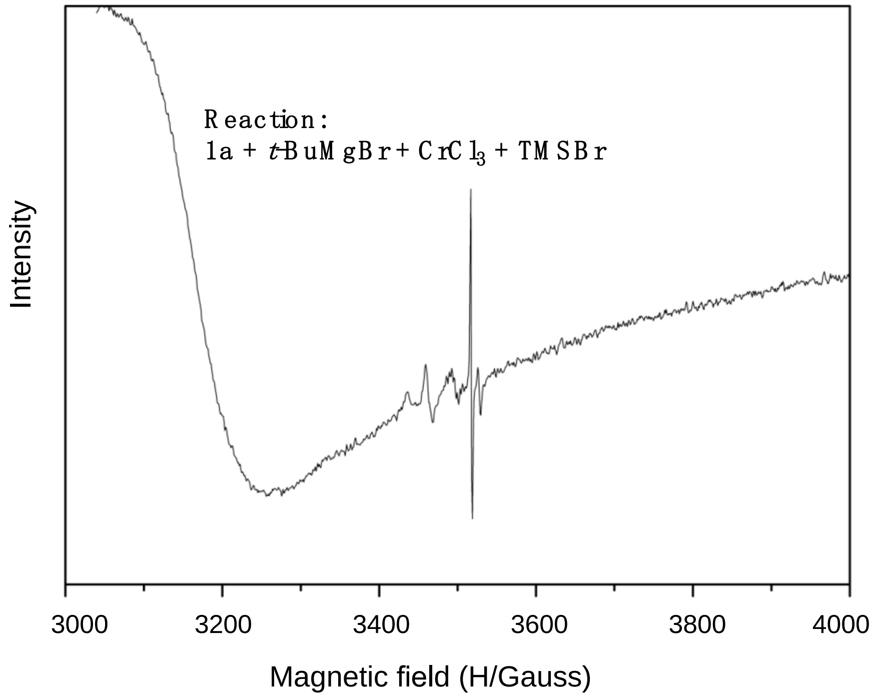

Supplementary Figure 9. EPR studies of stoichiometric reactions.

X-band EPR spectrum of the standard alkylation after 4 hours was recorded at room temperature. The sharp peak at about 3500 Oe is typical for radicals with the *g* value around 2.0, indicating that radical species are involved in the alkylation. Analysis of

tert-butylmagnesium bromide in THF by EPR spectroscopy suggested that no radical species was existed in the solution.

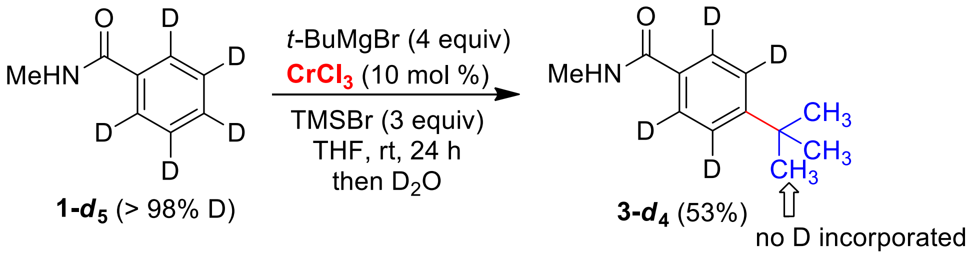

A dried Schlenk tube were placed **1-*d*$_5$** (28 mg, 0.2 mmol), $CrCl_3$ (3 mg, 0.02 mmol), and freshly distilled THF (0.5 mL). *Tert*-butylmagnesium bromide (1.2 mL, 0.8 mmol) was dropwise added by syringe at room temperature. After stirring the mixture for 30 min, trimethylbromosilane (92 mg, 0.6 mmol) was added by syringe and reacted at room temperature for 24 h. The resulting mixture was quenched by D_2O and extraction with ethyl acetate (3 x 10 mL). The combined organic phase was dried over anhydrous Na_2SO_4 and concentrated under vacuum. The crude product was purified by silica gel chromatography to give the desired coupling product **3-*d*$_4$** in 53% yield. The ^{1}H NMR analysis of the product show that no deuterium was introduced into the *tert*-butyl group.

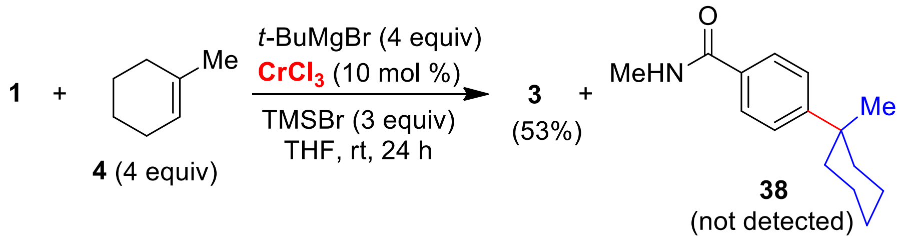

A dried Schlenk tube were placed *N*-methylbenzamide **1** (27 mg, 0.2 mmol), $CrCl_3$ (3 mg, 0.02 mmol), 1-methylcyclohex-1-ene **4** (77 mg, 4 equiv) and freshly distilled THF (0.5 mL). *Tert*-butylmagnesium bromide (1.2 mL, 0.8 mmol) was dropwise added by syringe at room temperature. After stirring the mixture for 30 min, trimethylbromosilane (92 mg, 0.6 mmol) was added by syringe and reacted at room temperature for 24 h. The resulting mixture was quenched by an aqueous solution of NH_4Cl. Analysis of the organic phase by GC/MS and TLC technique suggested that

the related hydroarylation compound **38** was not formed. The crude product was then extracted by ethyl acetate (3 x 10 mL), and the combined organic phase was dried over anhydrous Na_2SO_4 and concentrated under vacuum, which was purified by silica gel chromatography to give the product **3** in 53% yield.

^1H, ^{13}C and ^{19}F NMR Spectra

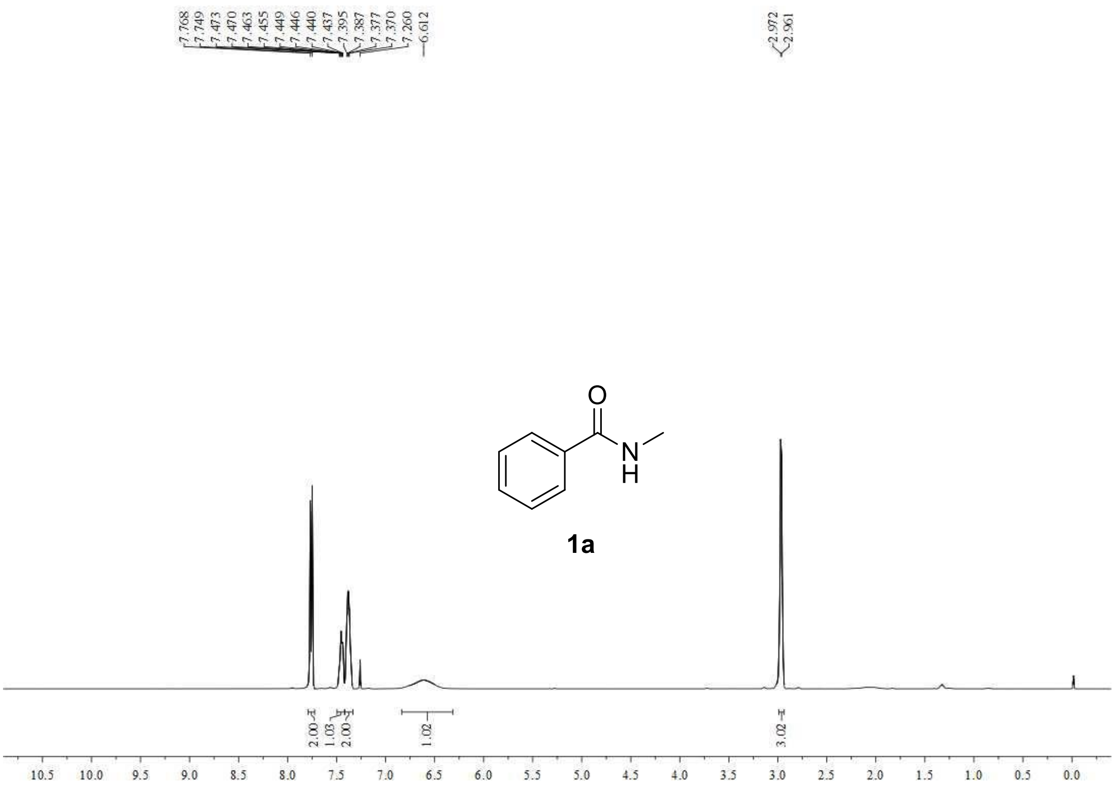

Supplementary Figure 10. ^1H NMR Spectrum of substrate 1a

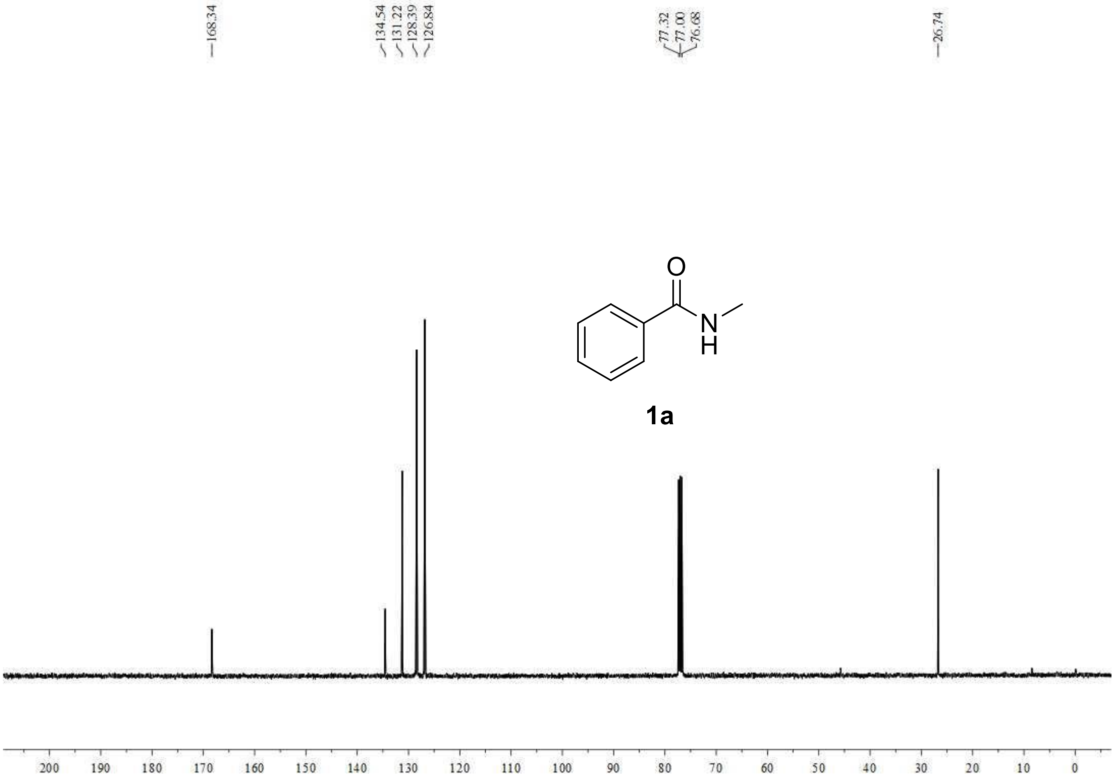

Supplementary Figure 11. ^{13}C NMR Spectrum of substrate 1a

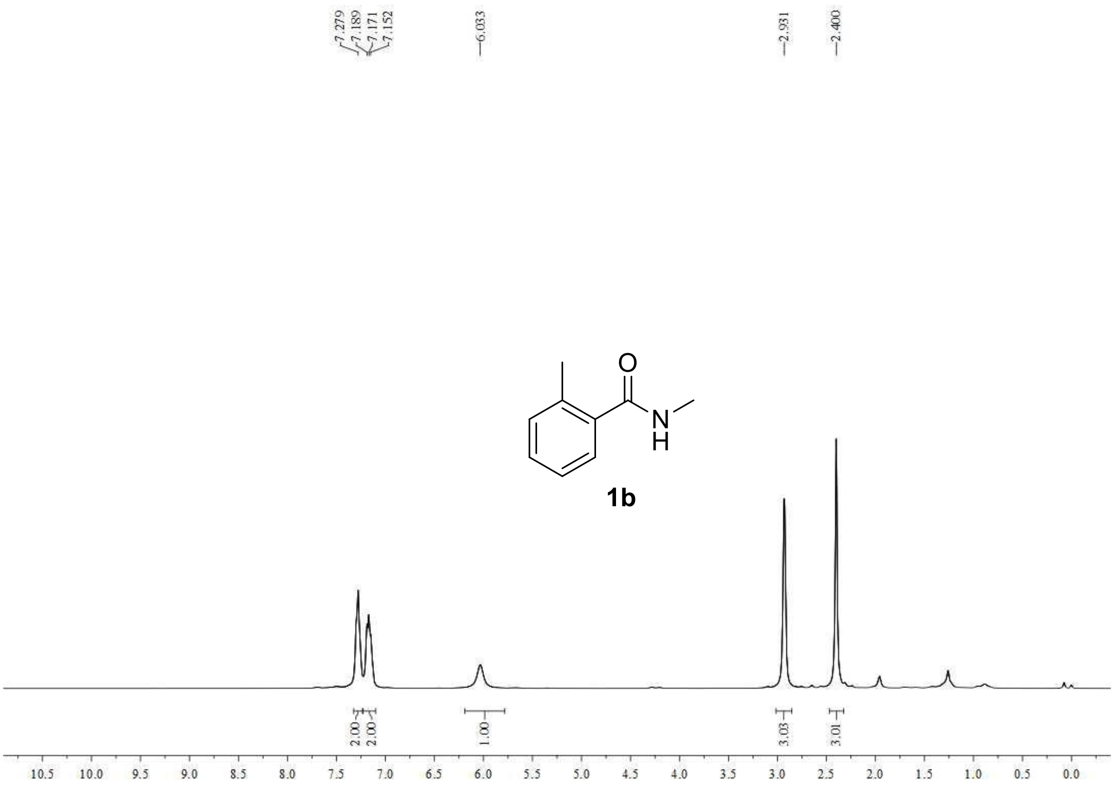

Supplementary Figure 12. ^{1}H NMR Spectrum of substrate 1b

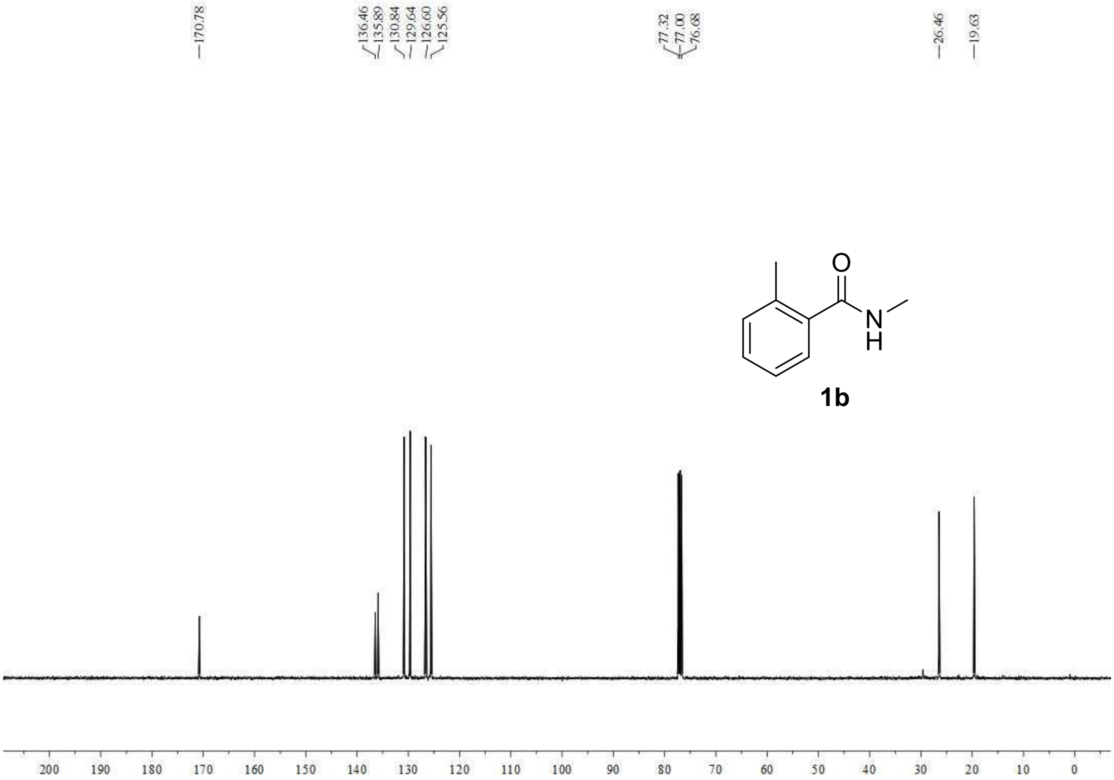

Supplementary Figure 13. ^{13}C NMR Spectrum of substrate 1b

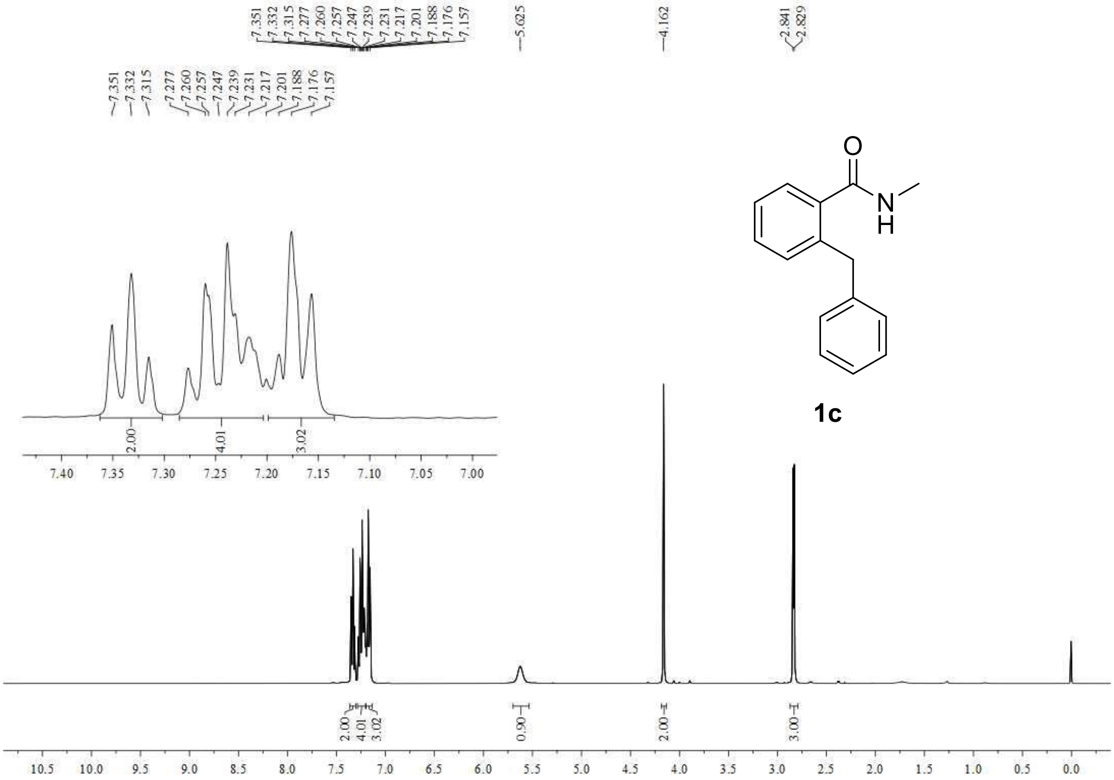

Supplementary Figure 14. ^{1}H NMR Spectrum of substrate 1c

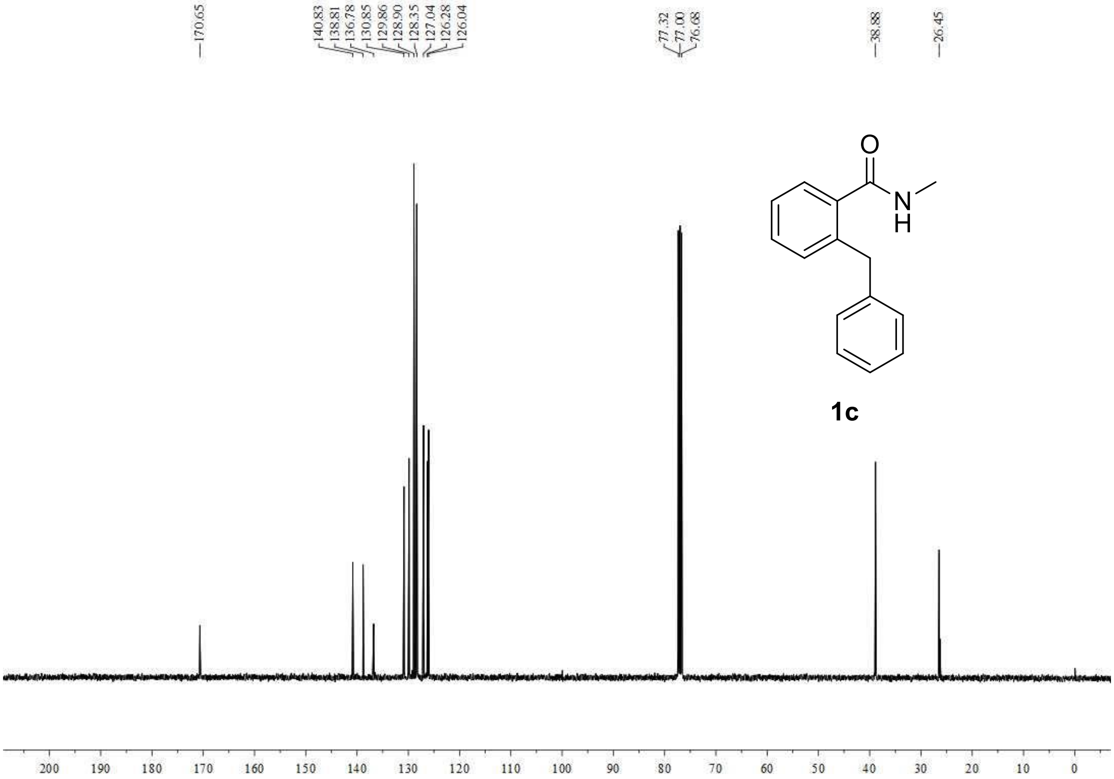

Supplementary Figure 15. ^{13}C NMR Spectrum of substrate 1c

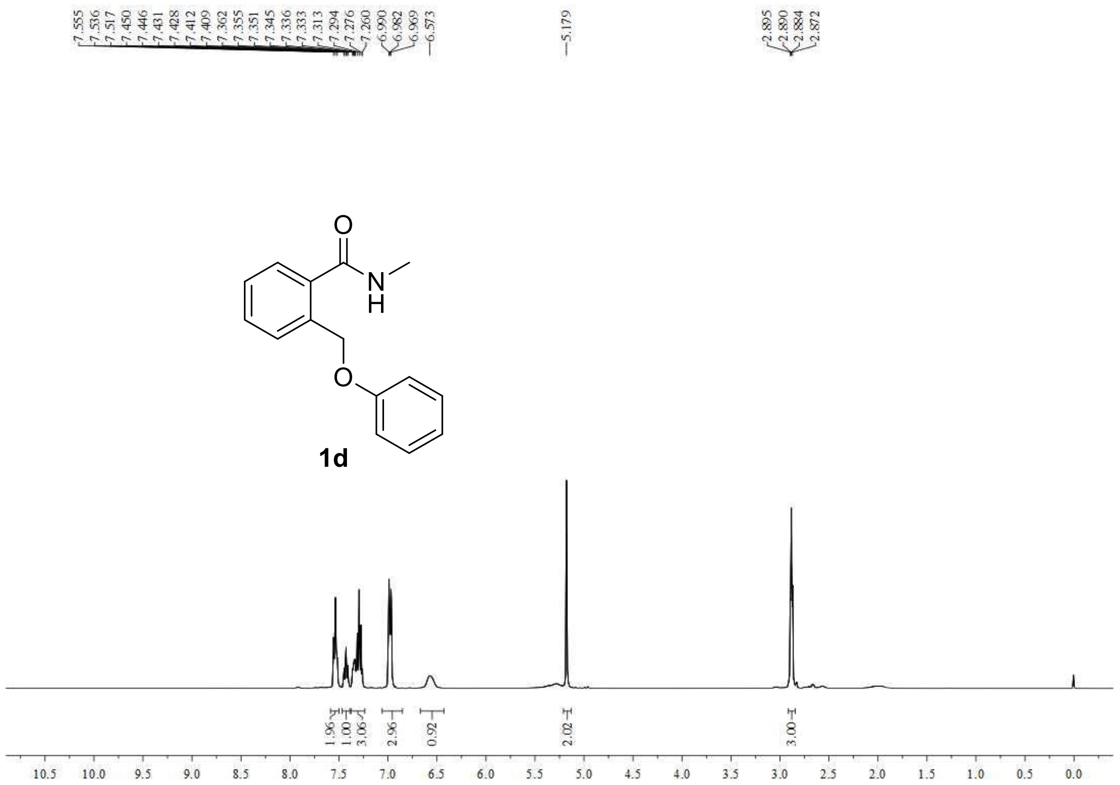

Supplementary Figure 16. ^{1}H NMR Spectrum of substrate 1d

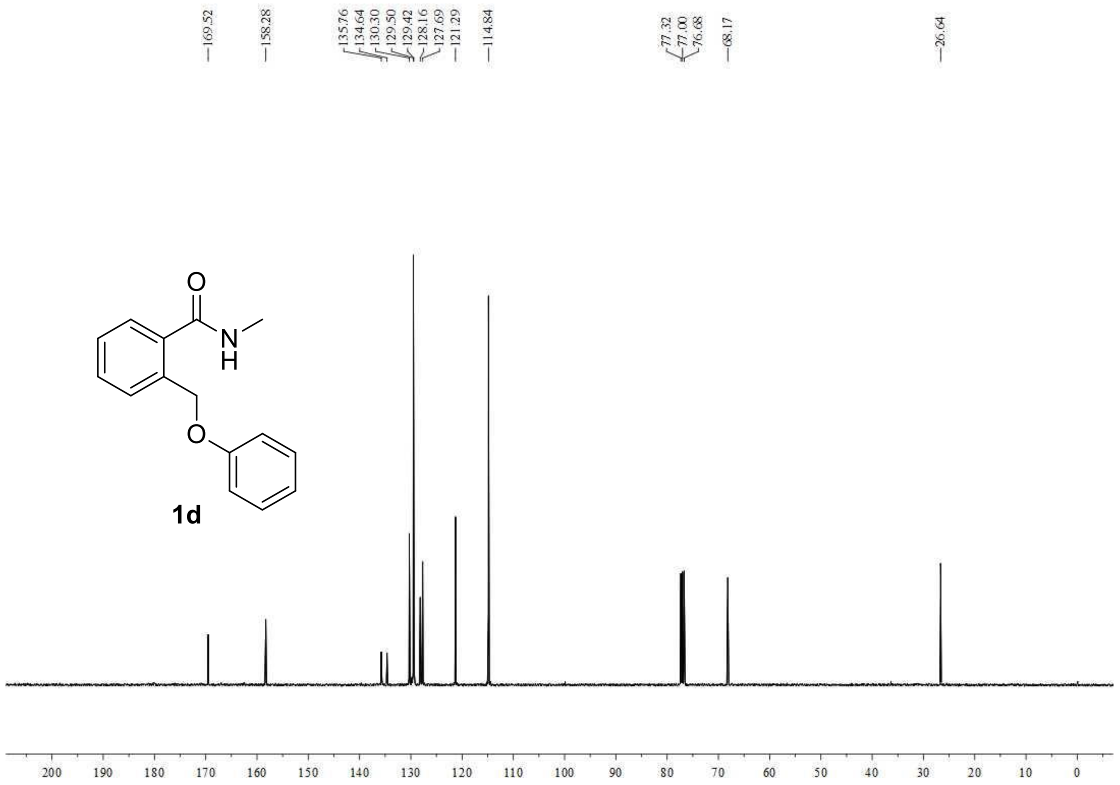

Supplementary Figure 17. ^{13}C NMR Spectrum of substrate 1d

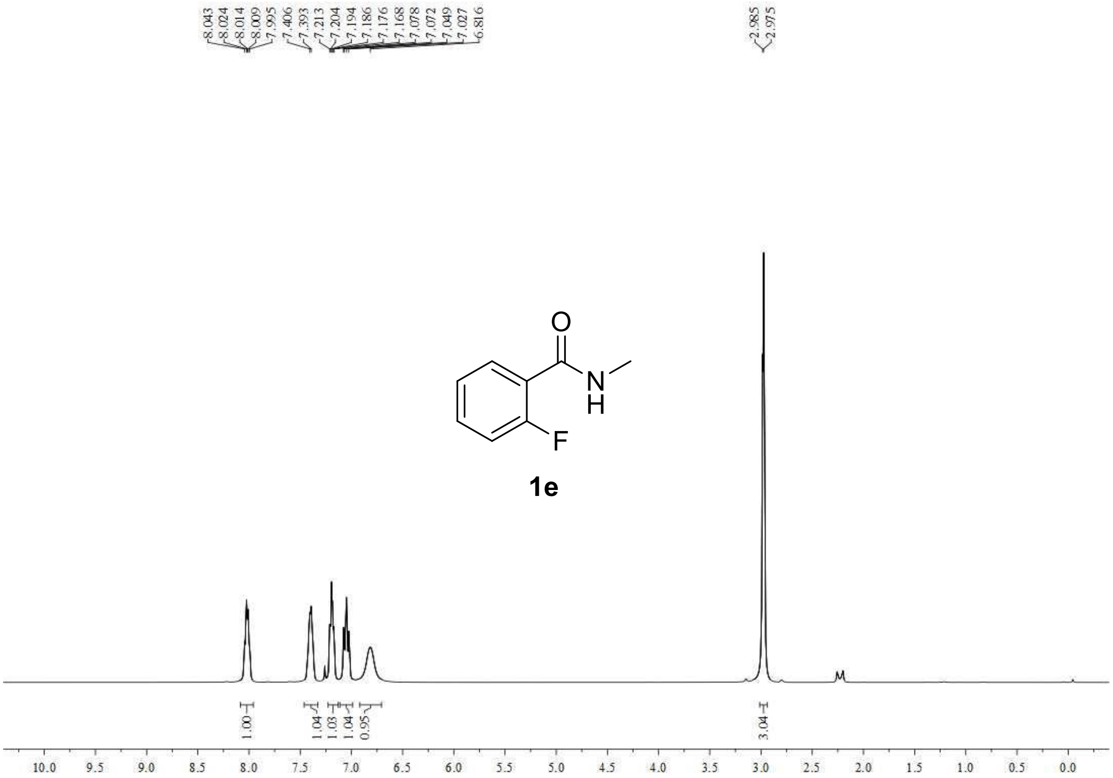

Supplementary Figure 18. ^{1}H NMR Spectrum of substrate 1e

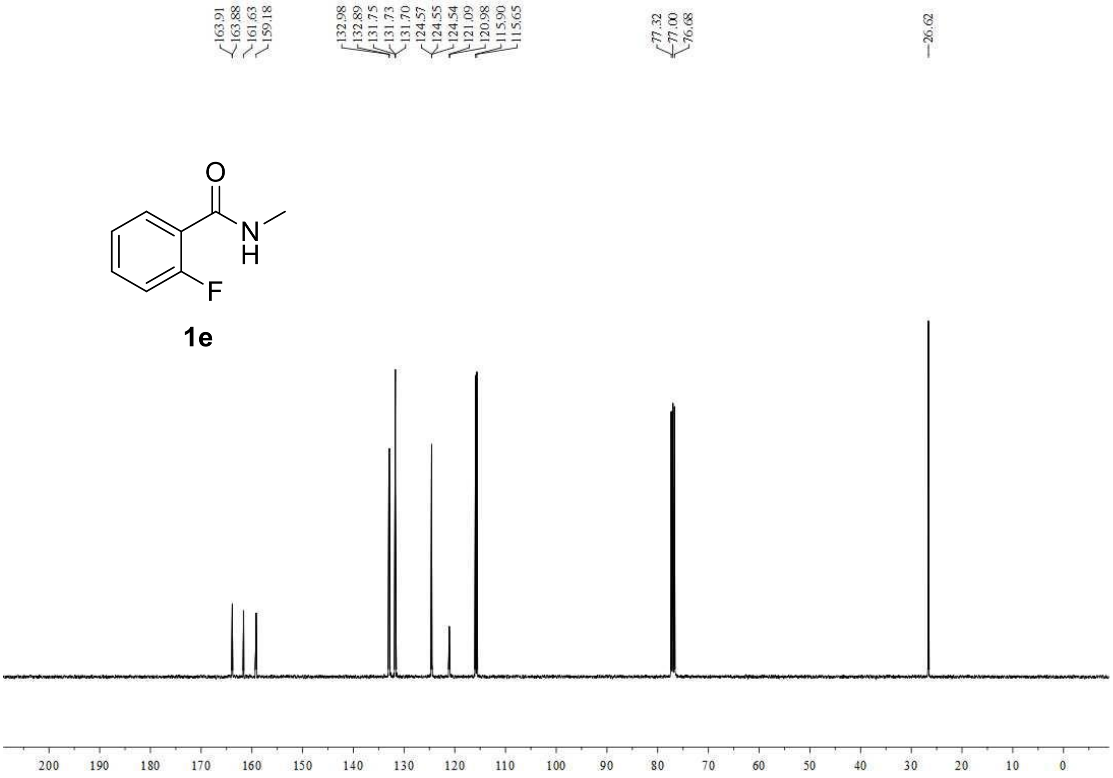

Supplementary Figure 19. ^{13}C NMR Spectrum of substrate 1e

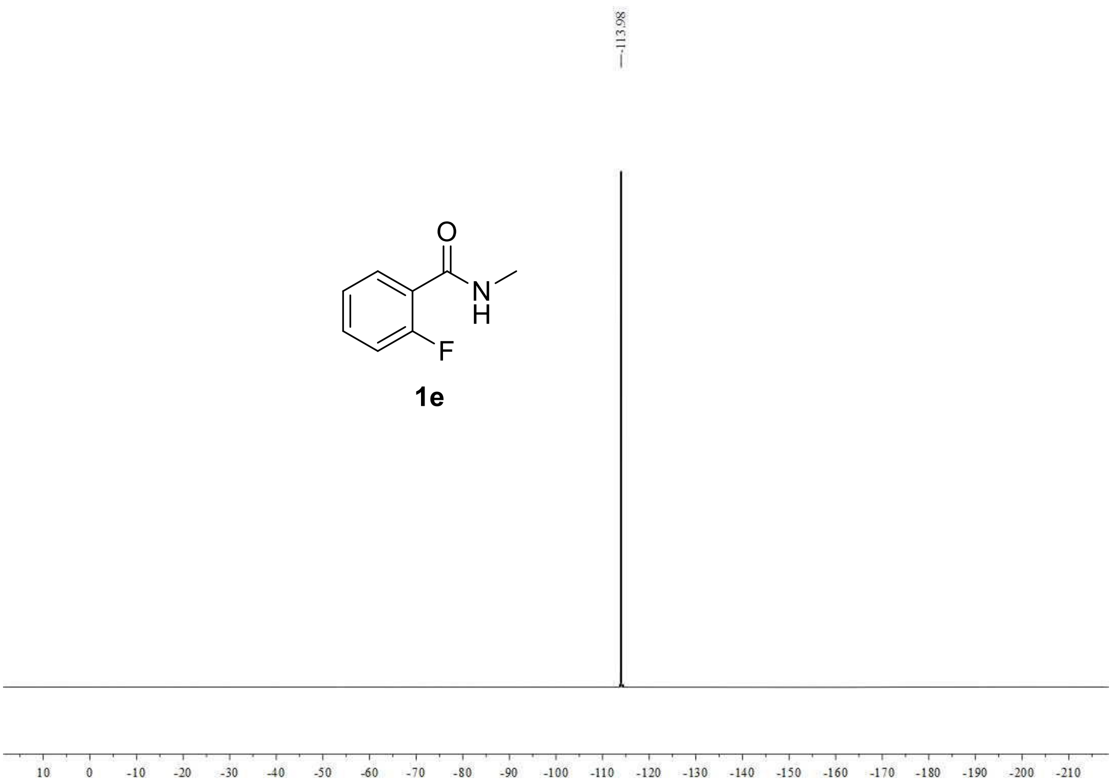

Supplementary Figure 20. ^{19}F NMR Spectrum of substrate 1e

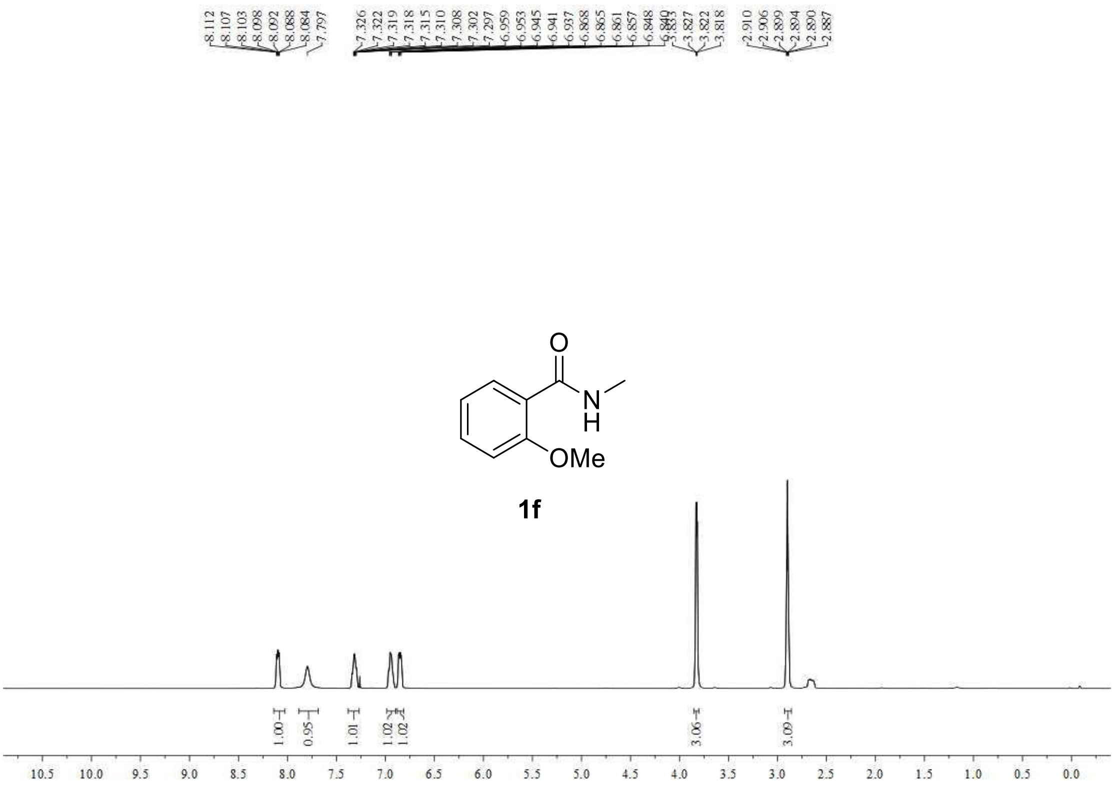

Supplementary Figure 21. ^{1}H NMR Spectrum of substrate 1f

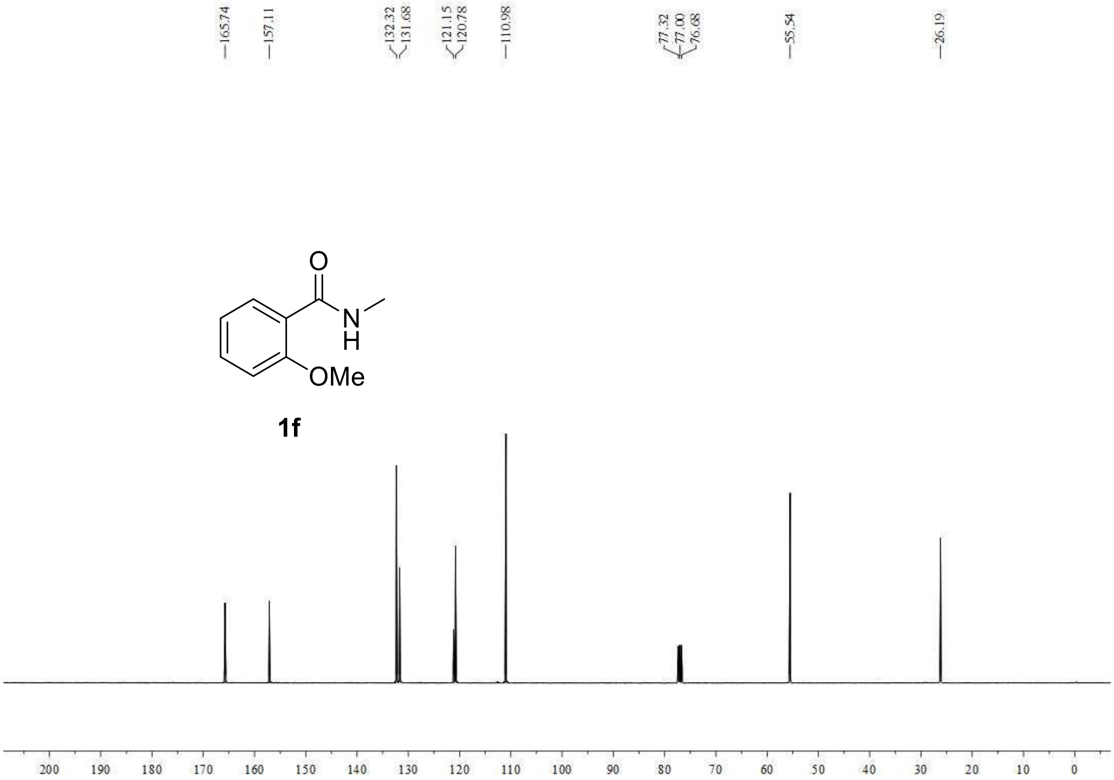

Supplementary Figure 22. ^{13}C NMR Spectrum of substrate 1f

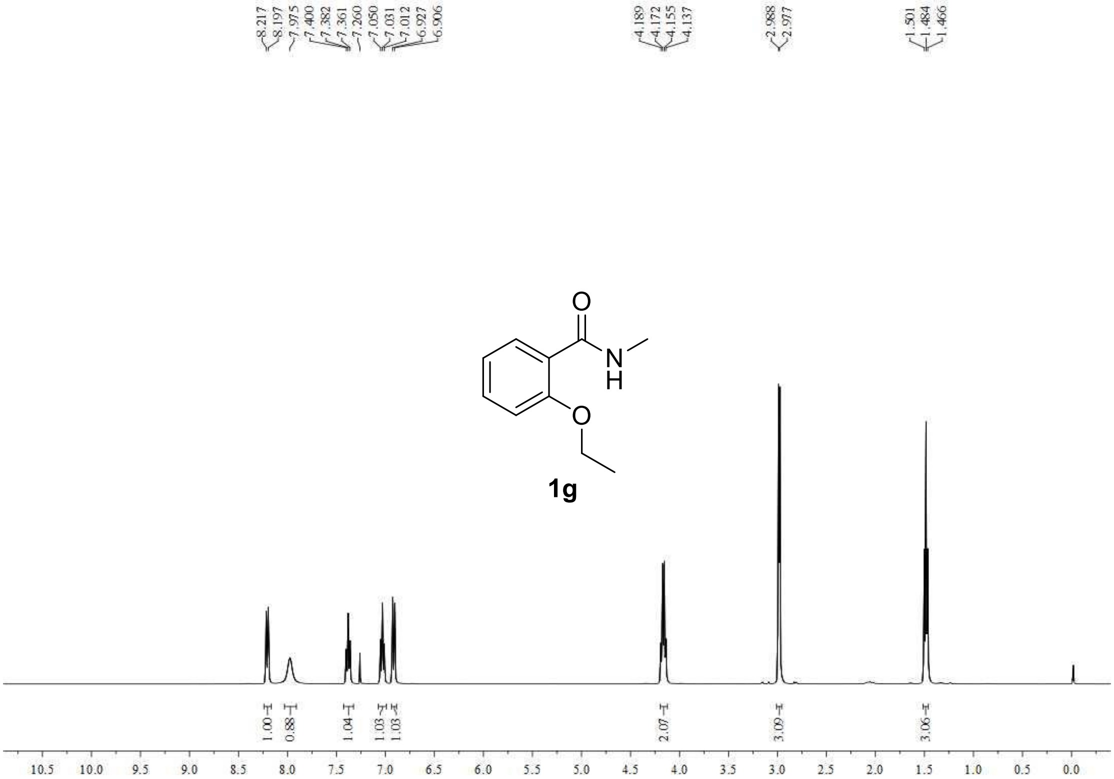

Supplementary Figure 23. ^{1}H NMR Spectrum of substrate 1g

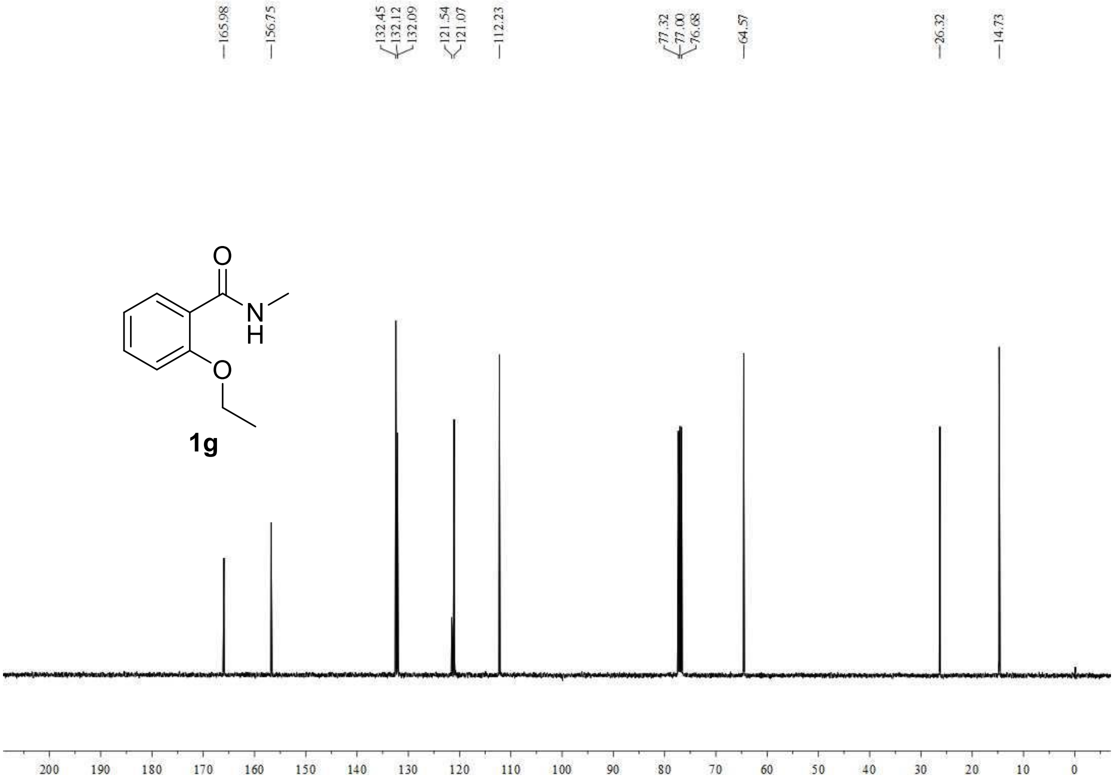

Supplementary Figure 24. ^{13}C NMR Spectrum of substrate 1g

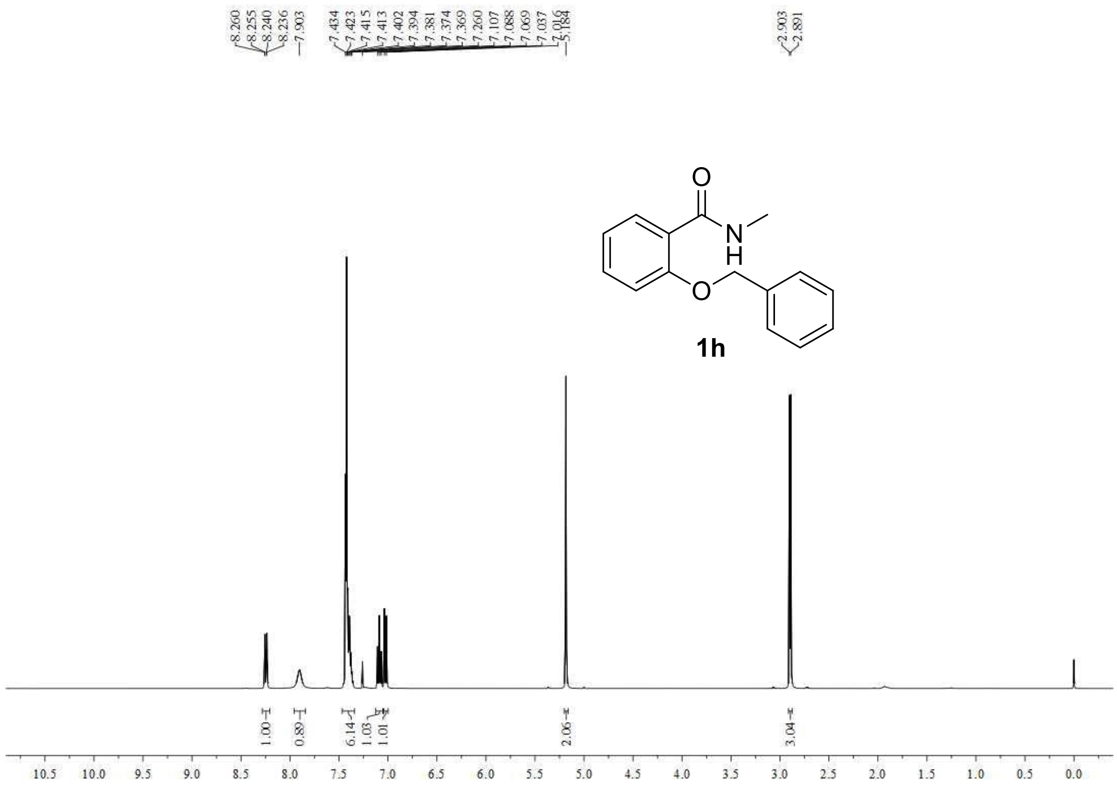

Supplementary Figure 25. ^{1}H NMR Spectrum of substrate 1h

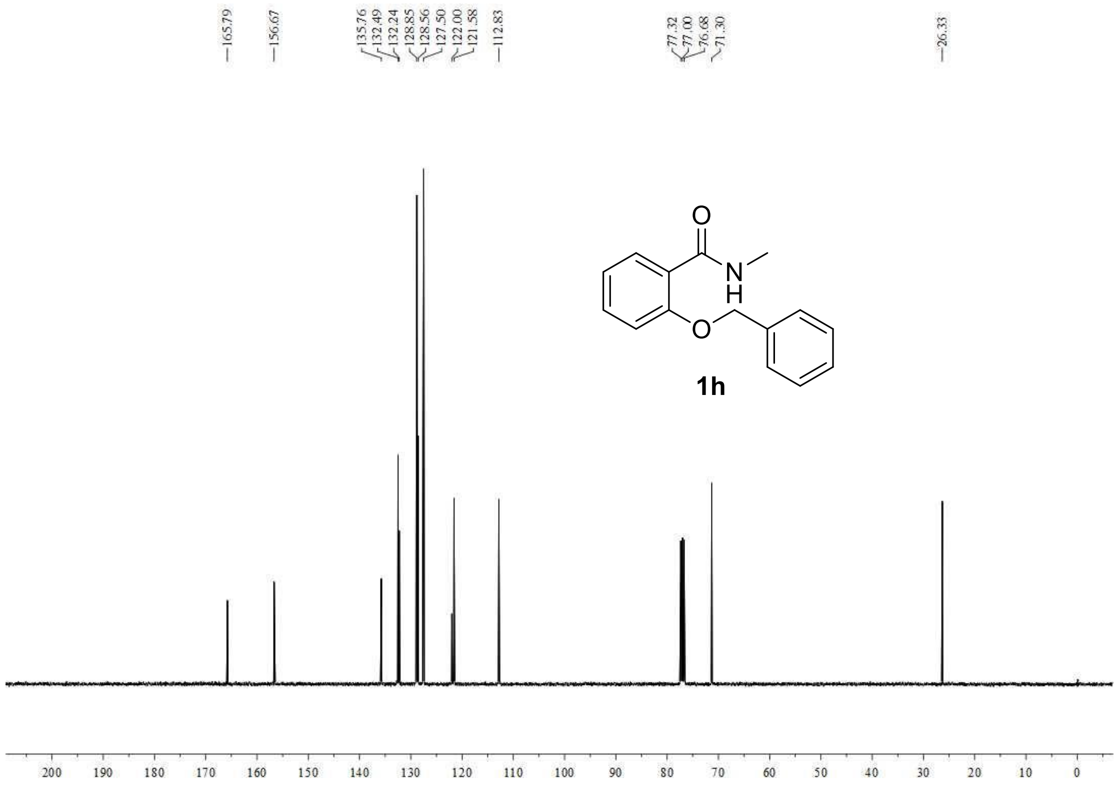

Supplementary Figure 26. ^{13}C NMR Spectrum of substrate 1h

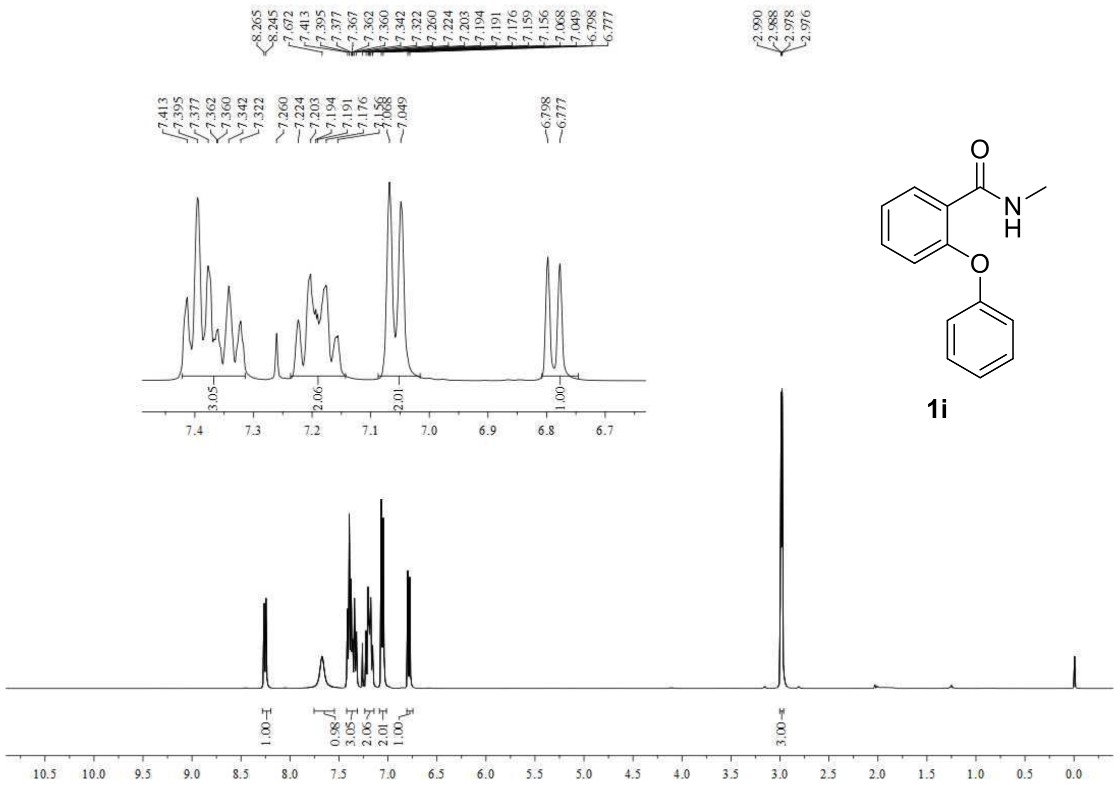

Supplementary Figure 27. ^{1}H NMR Spectrum of substrate 1i

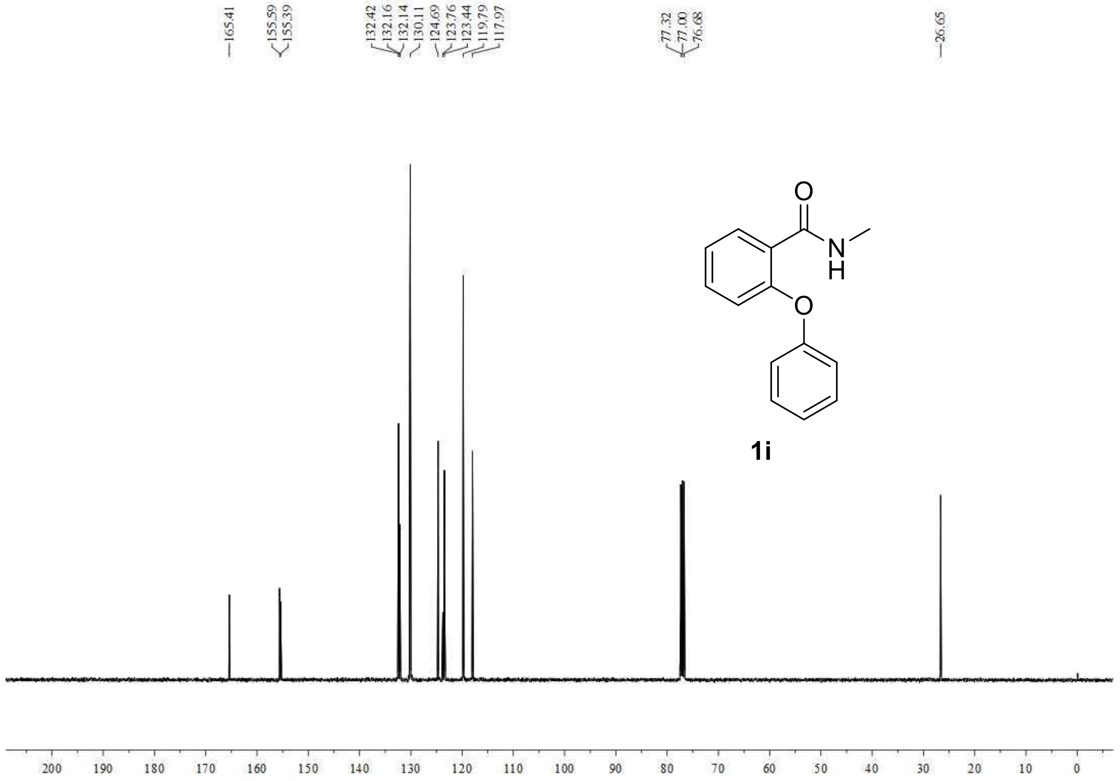

Supplementary Figure 28. ^{13}C NMR Spectrum of substrate 1i

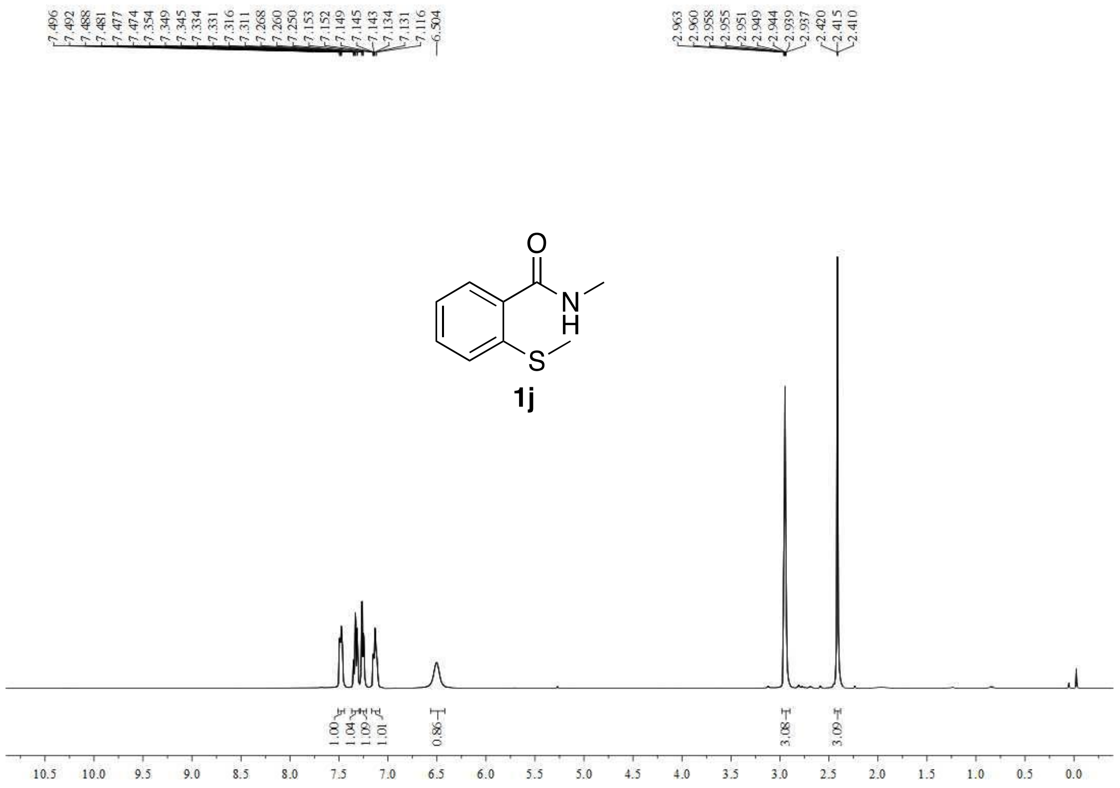

Supplementary Figure 29. ^{1}H NMR Spectrum of substrate 1j

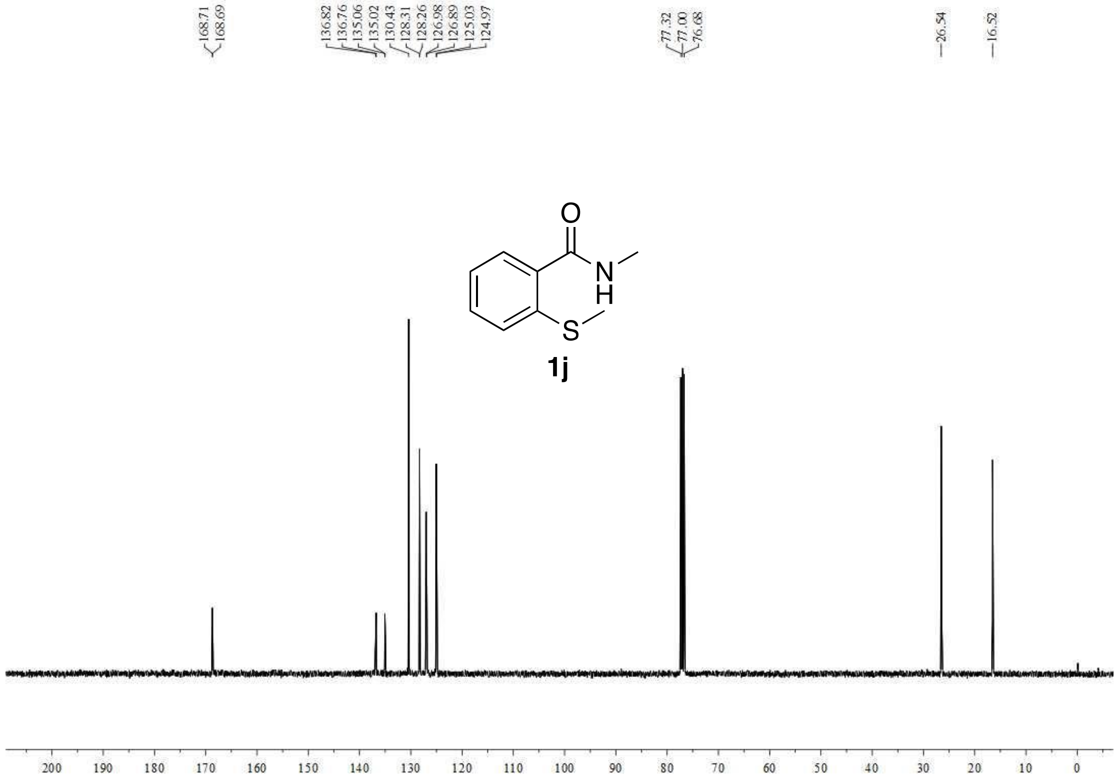

Supplementary Figure 30. ^{13}C NMR Spectrum of substrate 1j

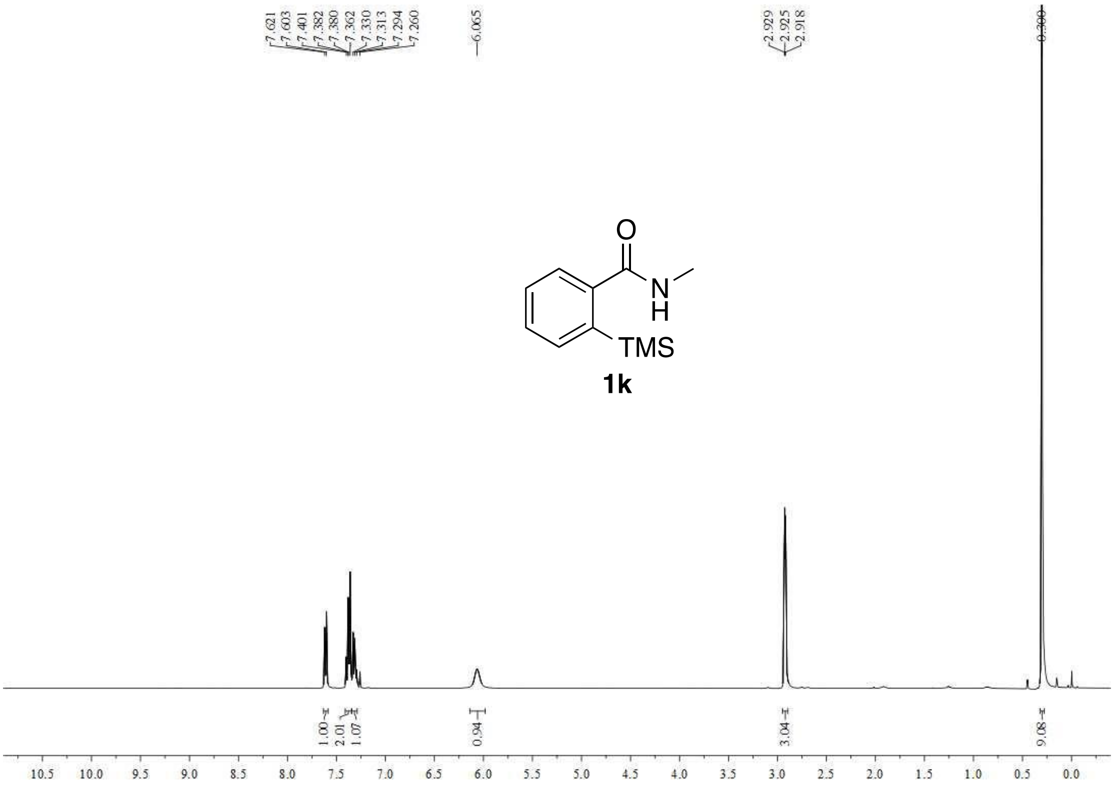

Supplementary Figure 31. ^{1}H NMR Spectrum of substrate 1k

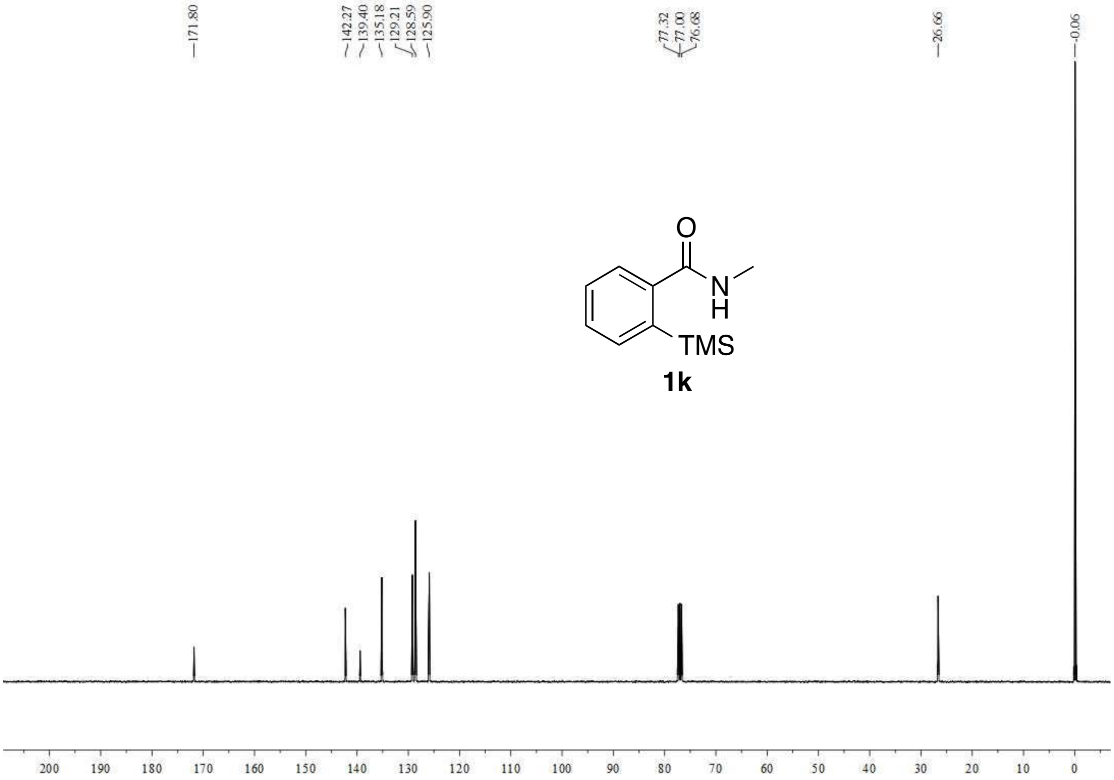

Supplementary Figure 32. ^{13}C NMR Spectrum of substrate 1k

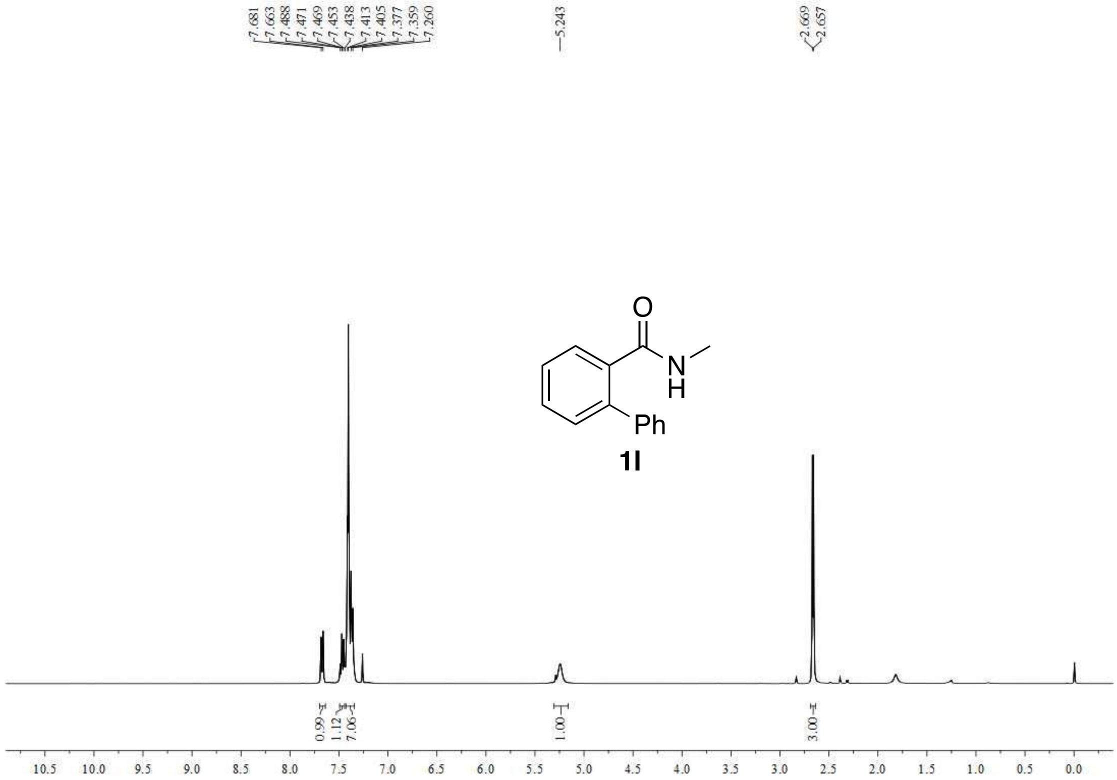

Supplementary Figure 33. ^{1}H NMR Spectrum of substrate 1l

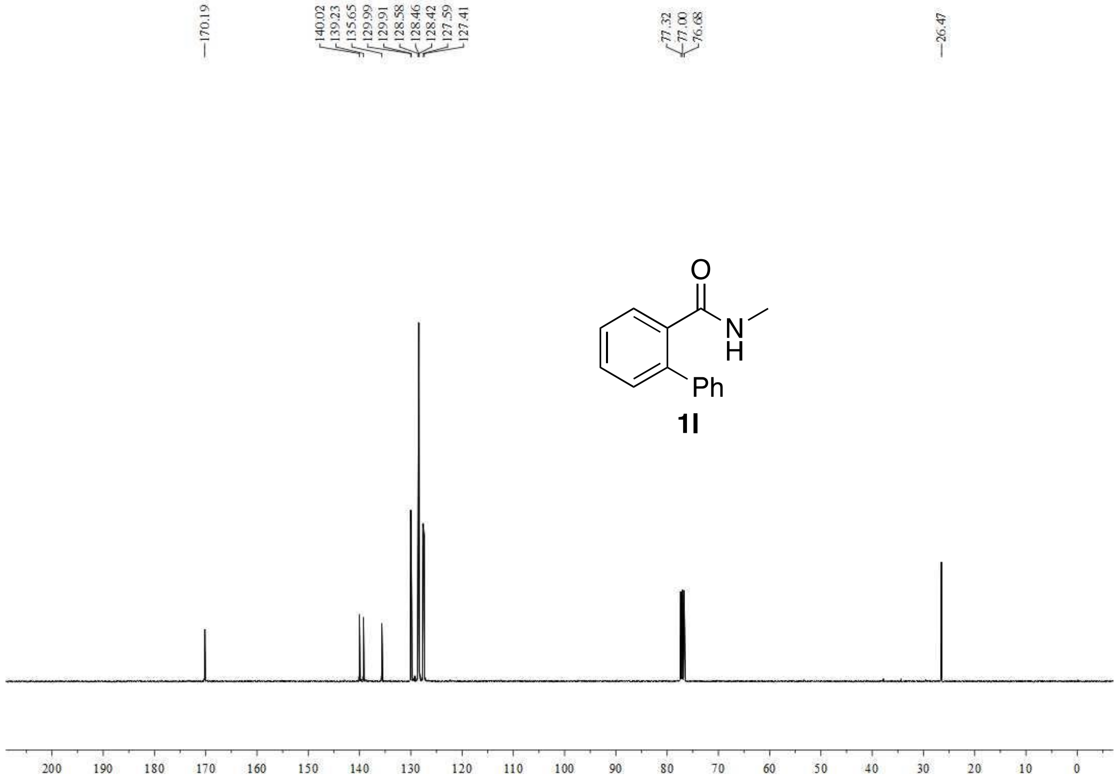

Supplementary Figure 34. ^{13}C NMR Spectrum of substrate 1l

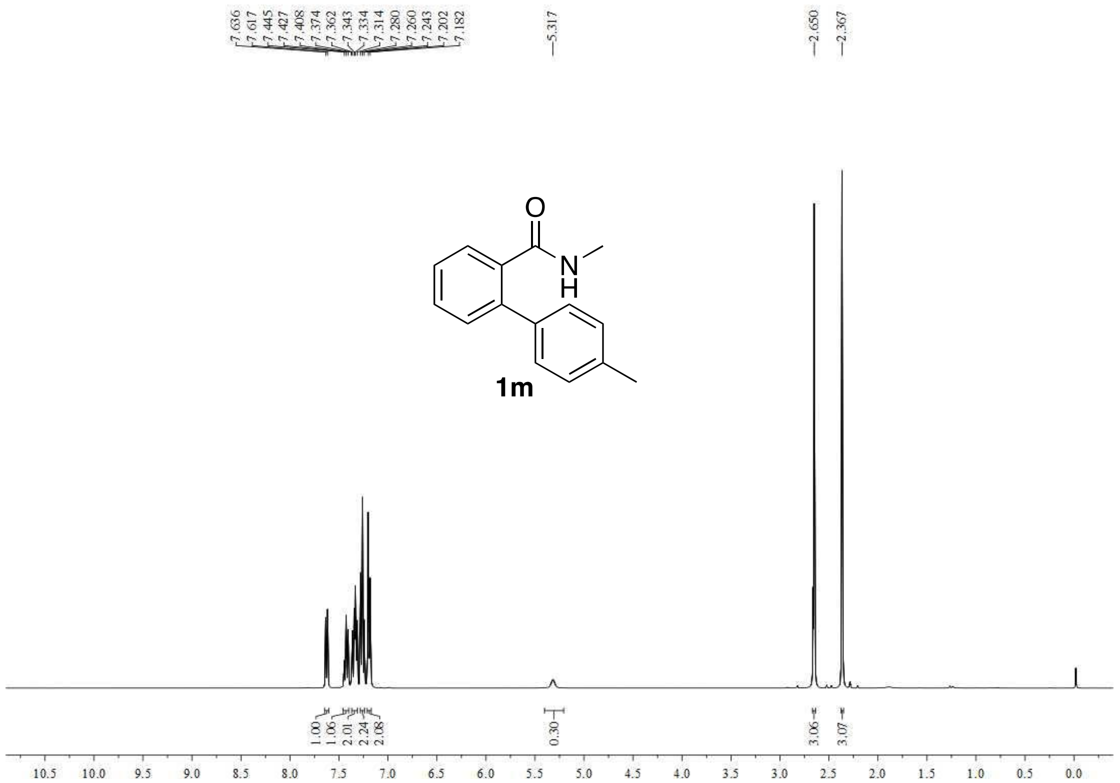

Supplementary Figure 35. ^{1}H NMR Spectrum of substrate 1m

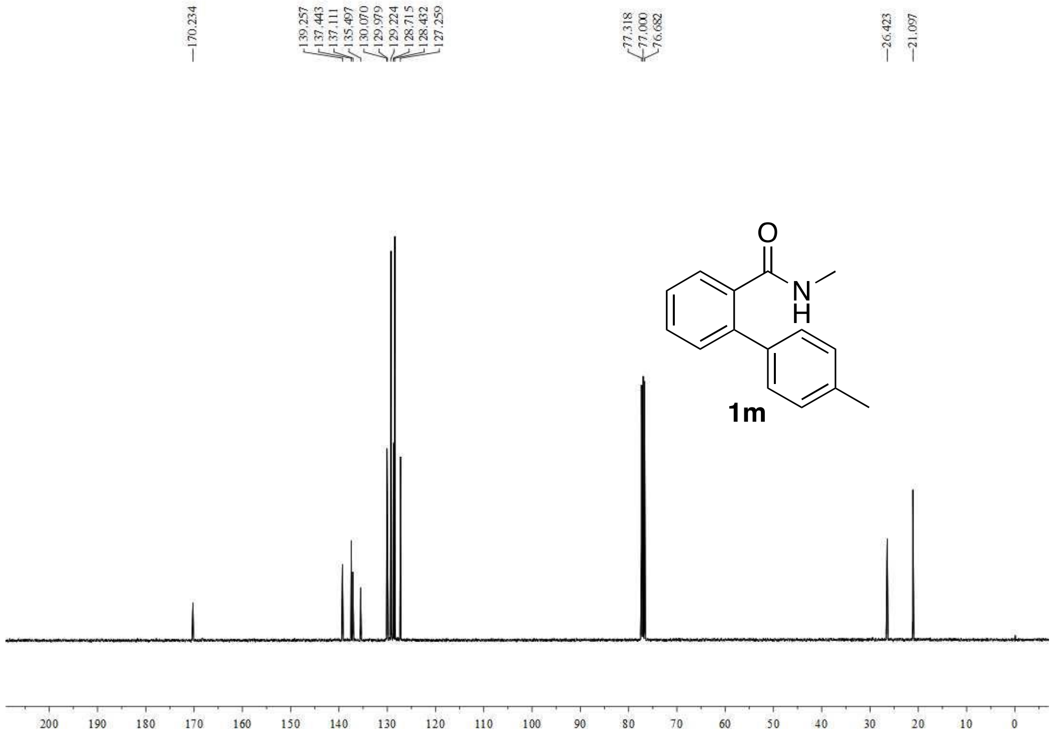

Supplementary Figure 36. ^{13}C NMR Spectrum of substrate 1m

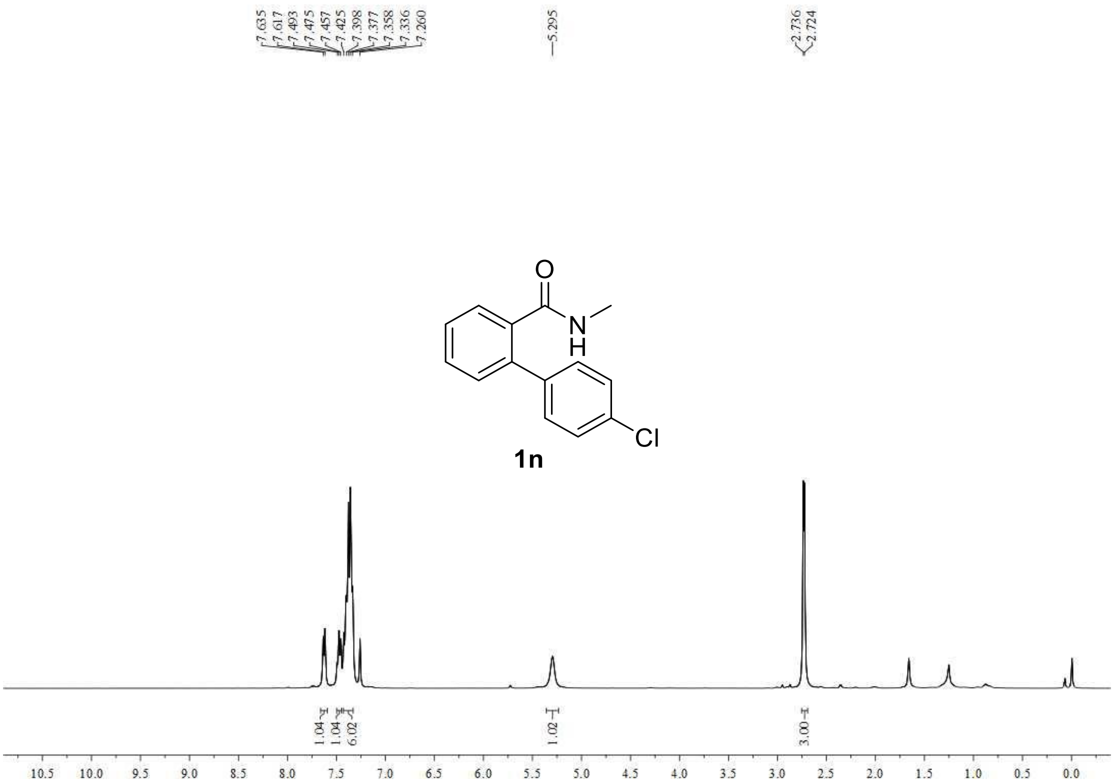

Supplementary Figure 37. ^{1}H NMR Spectrum of substrate 1n

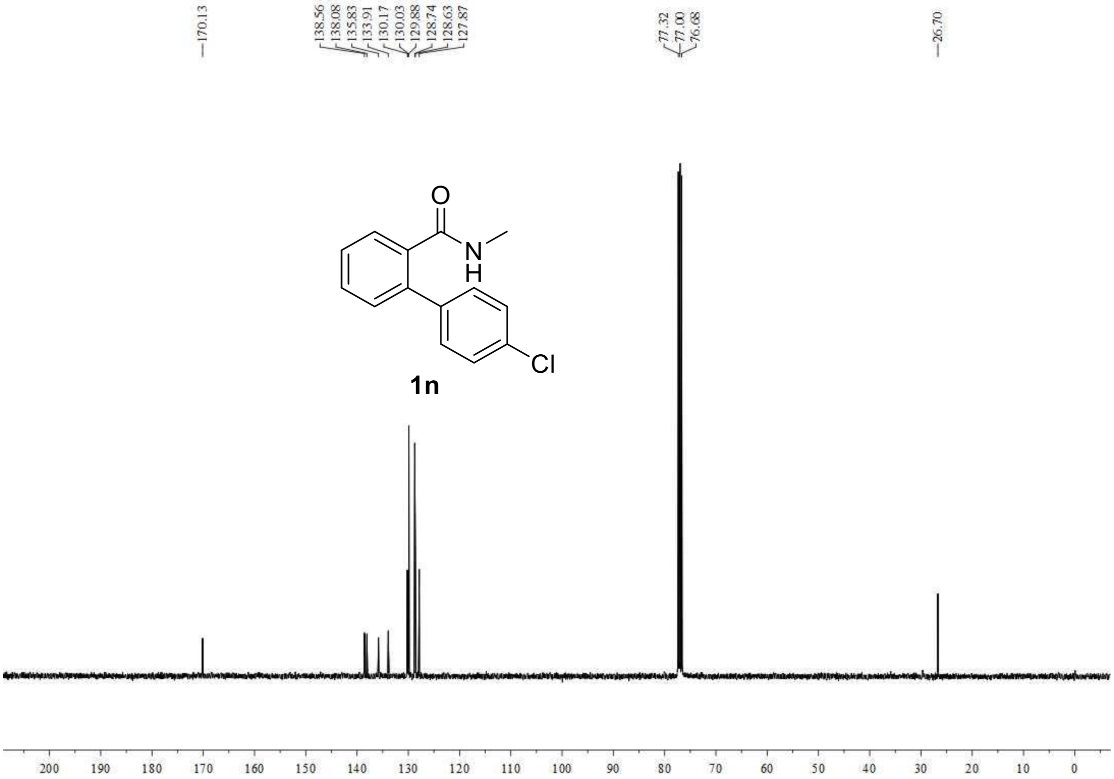

Supplementary Figure 38. ^{13}C NMR Spectrum of substrate 1n

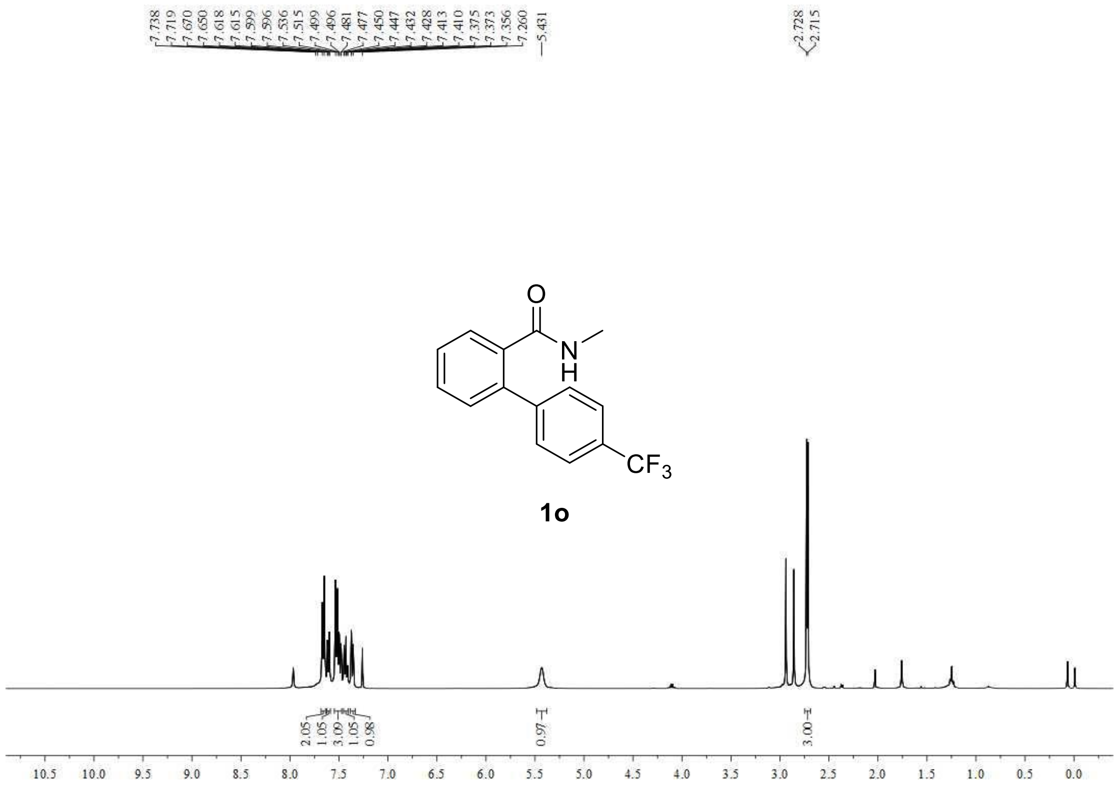

Supplementary Figure 39. ^{1}H NMR Spectrum of substrate 1o

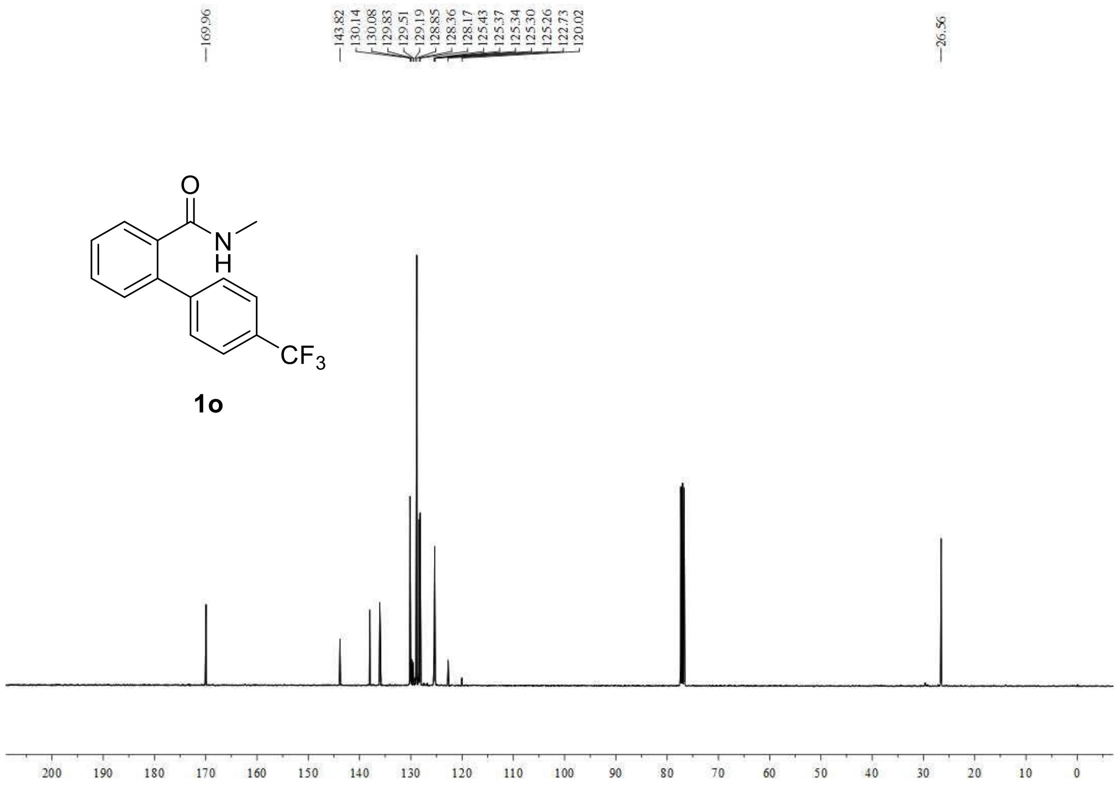

Supplementary Figure 40. ^{13}C NMR Spectrum of substrate 1o

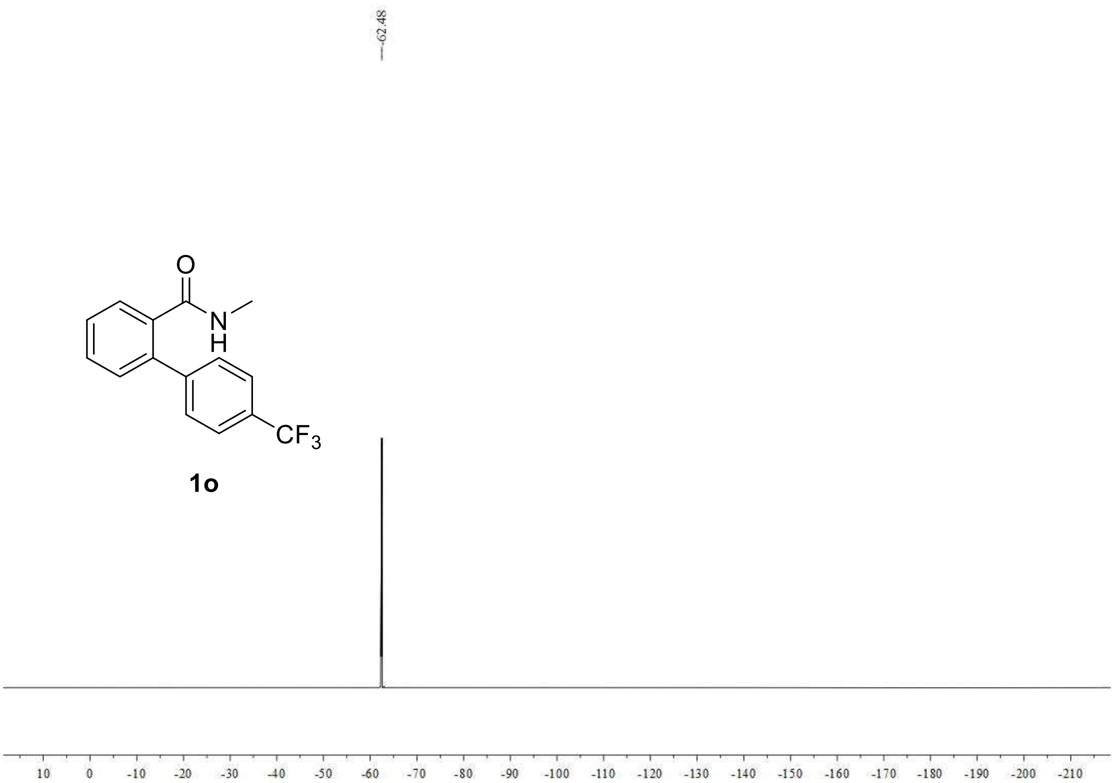

Supplementary Figure 41. ^{19}F NMR Spectrum of substrate 1o

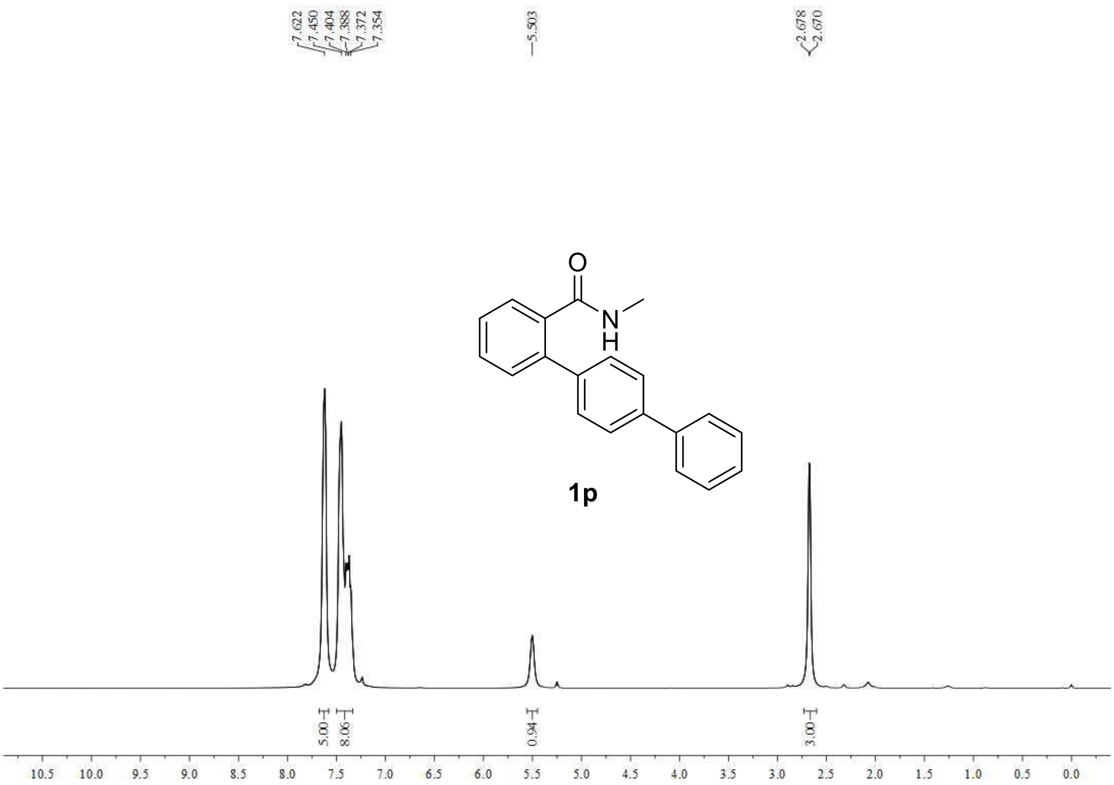

Supplementary Figure 42. ^{1}H NMR Spectrum of substrate 1p

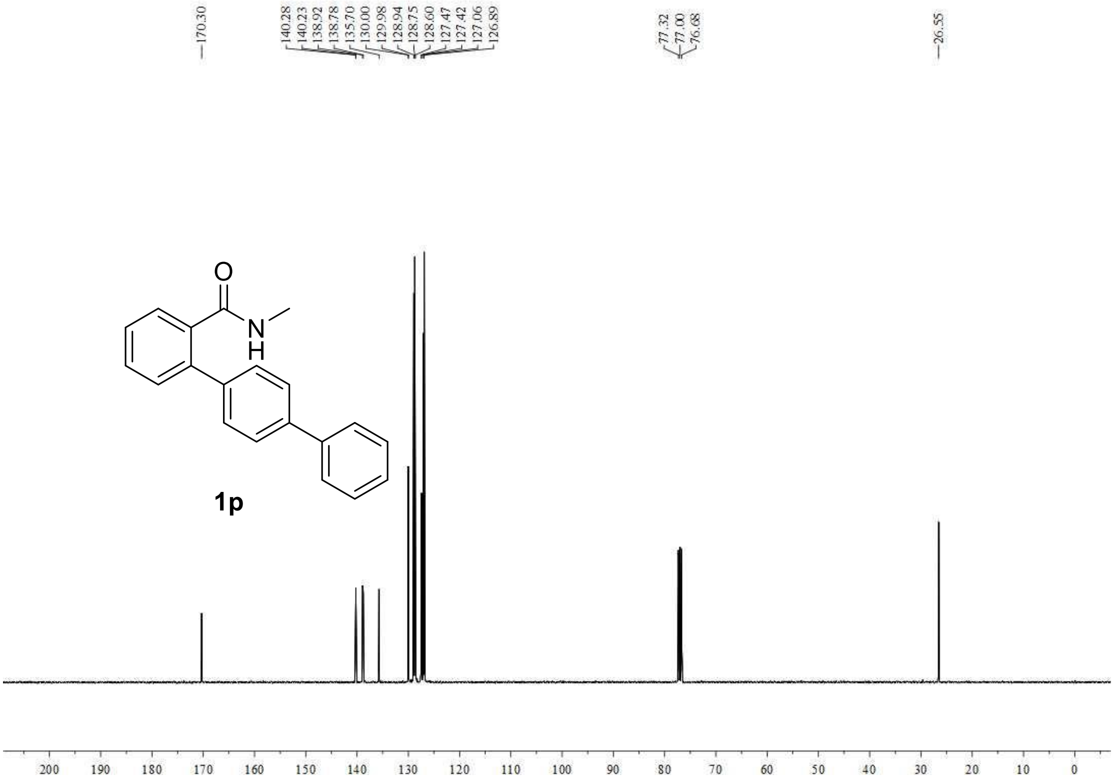

Supplementary Figure 43. ^{13}C NMR Spectrum of substrate 1p

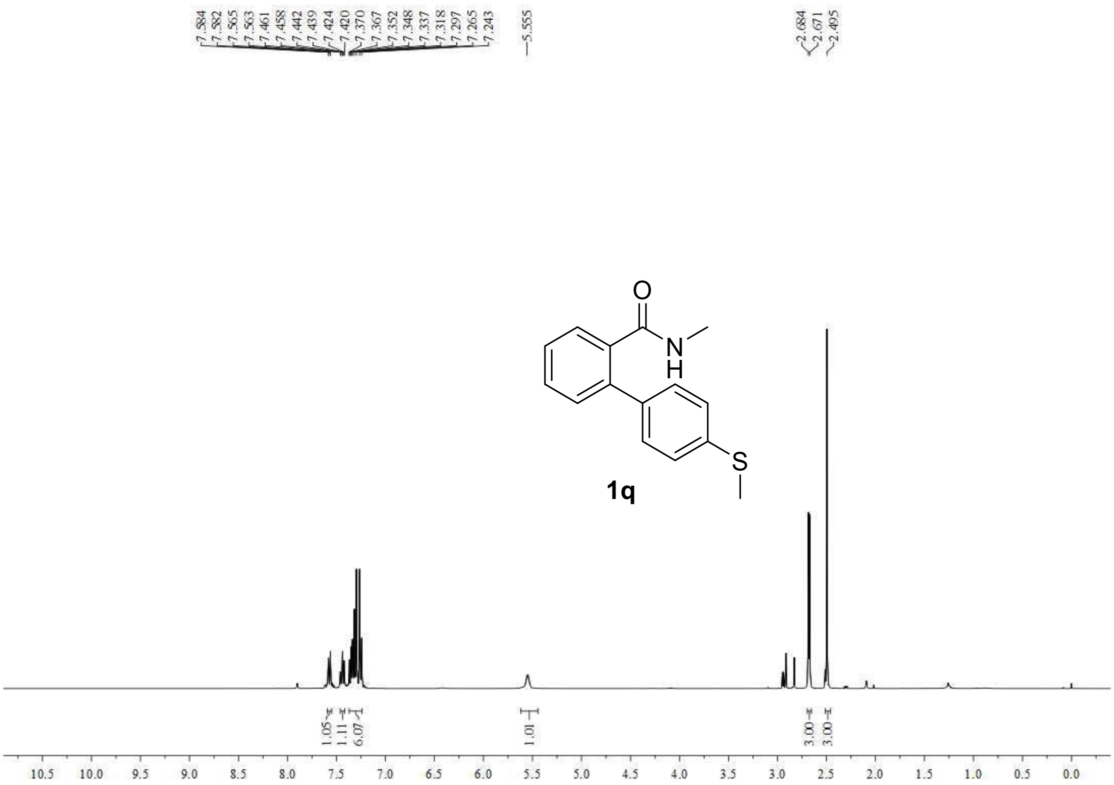

Supplementary Figure 44. ^{1}H NMR Spectrum of substrate 1q

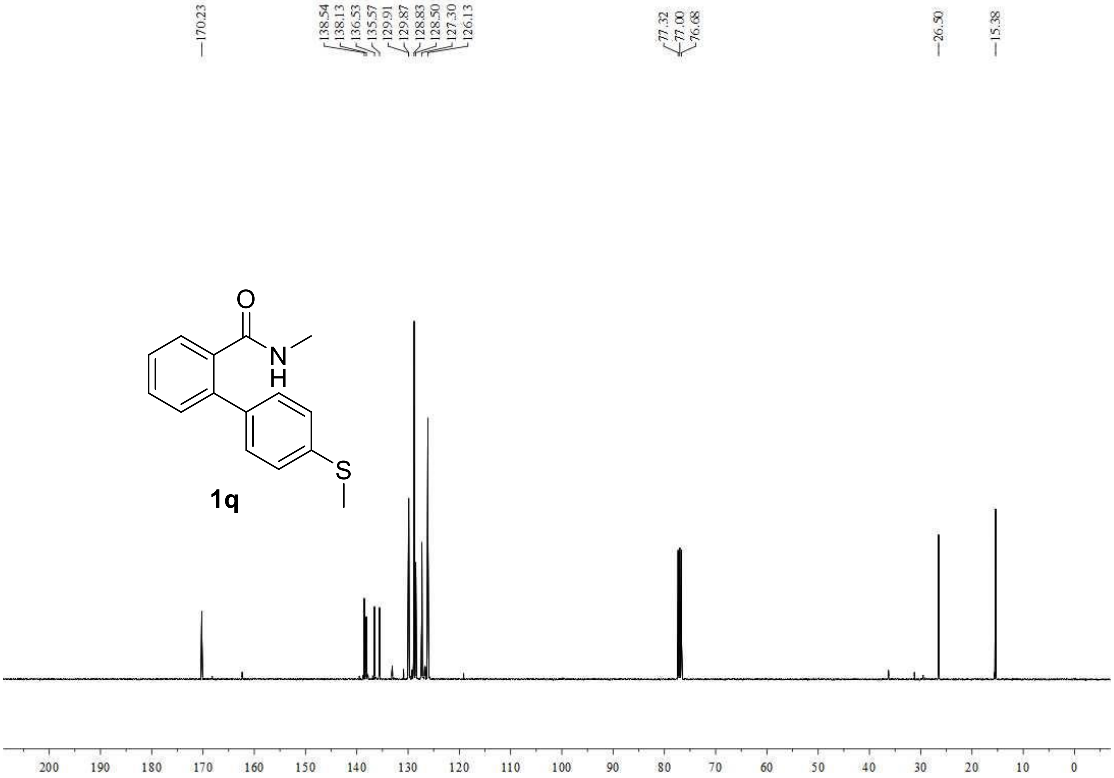

Supplementary Figure 45. ^{13}C NMR Spectrum of substrate 1q

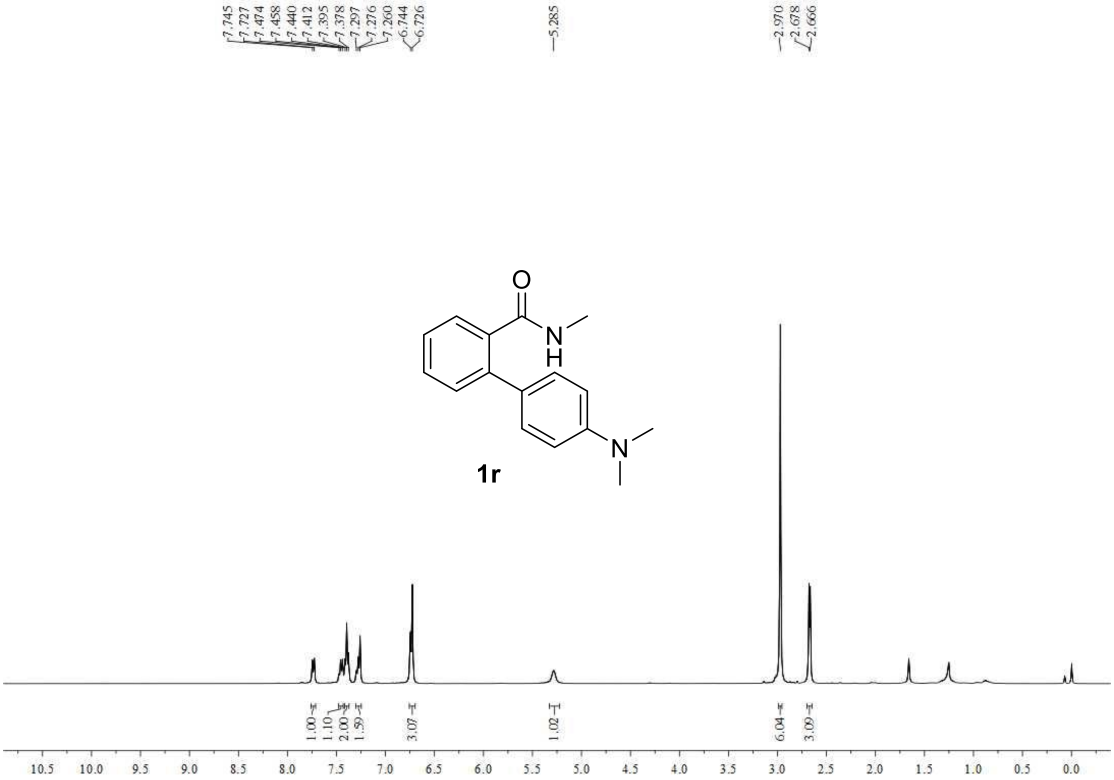

Supplementary Figure 46. ^{1}H NMR Spectrum of substrate 1r

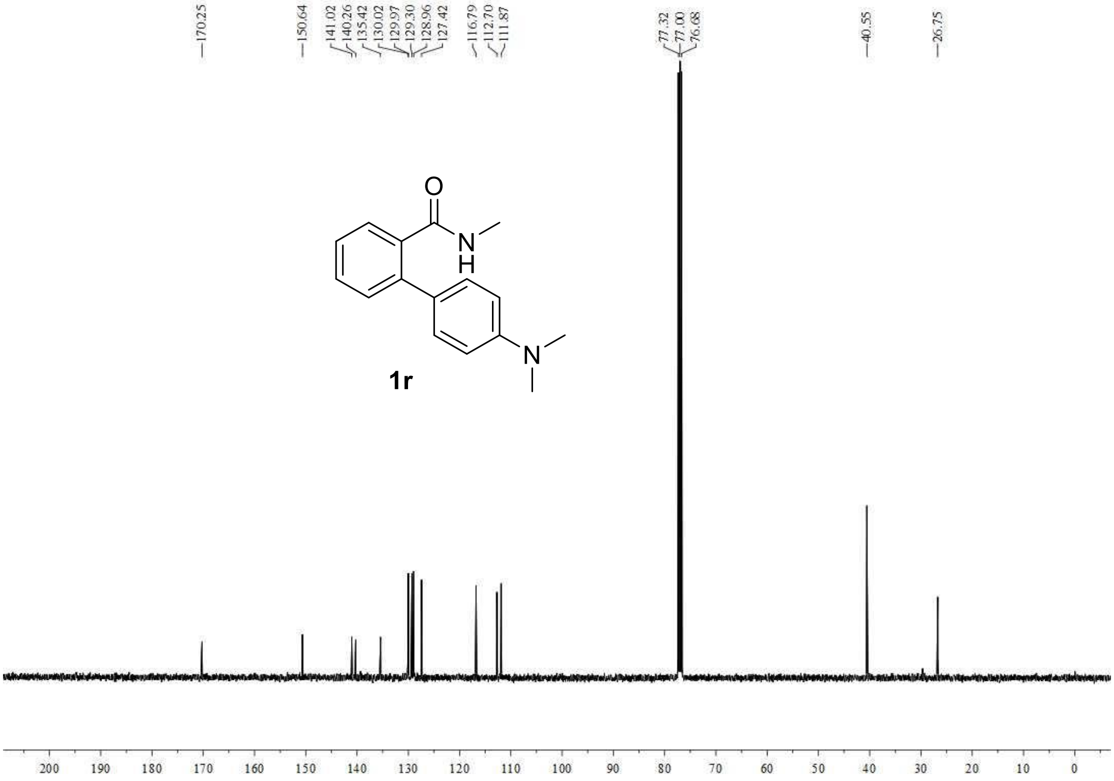

Supplementary Figure 47. ^{13}C NMR Spectrum of substrate 1r

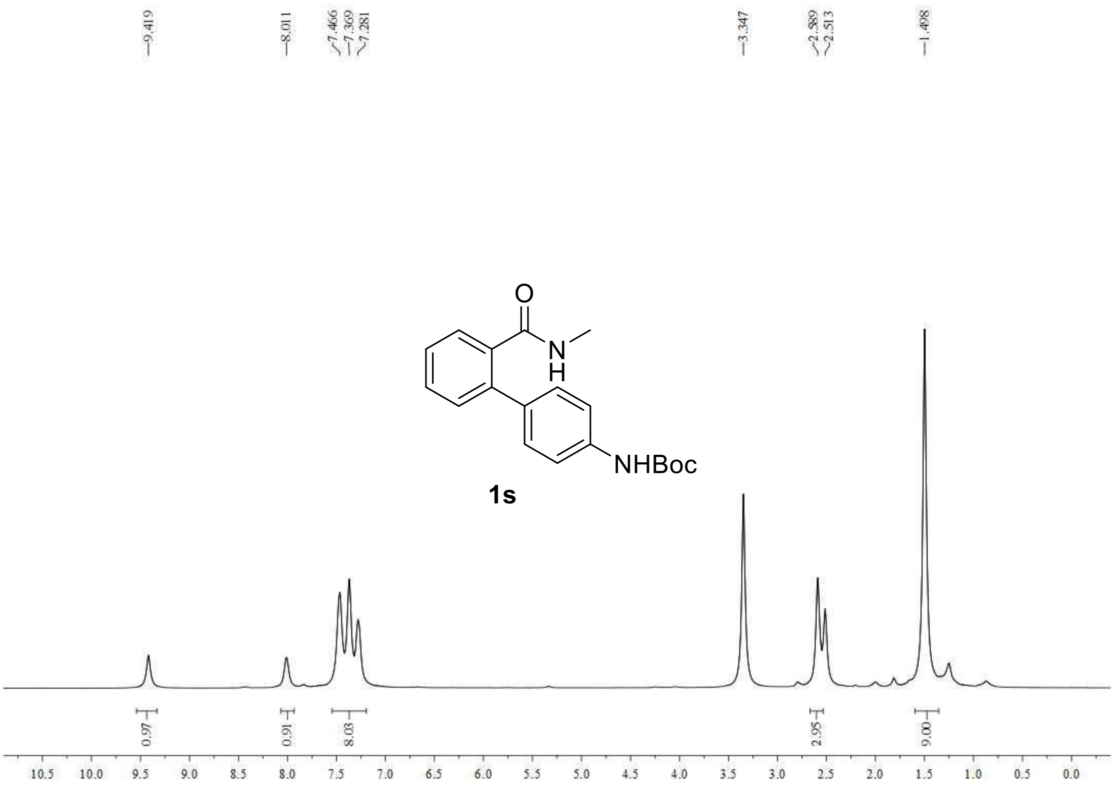

Supplementary Figure 48. ^{1}H NMR Spectrum of substrate 1s

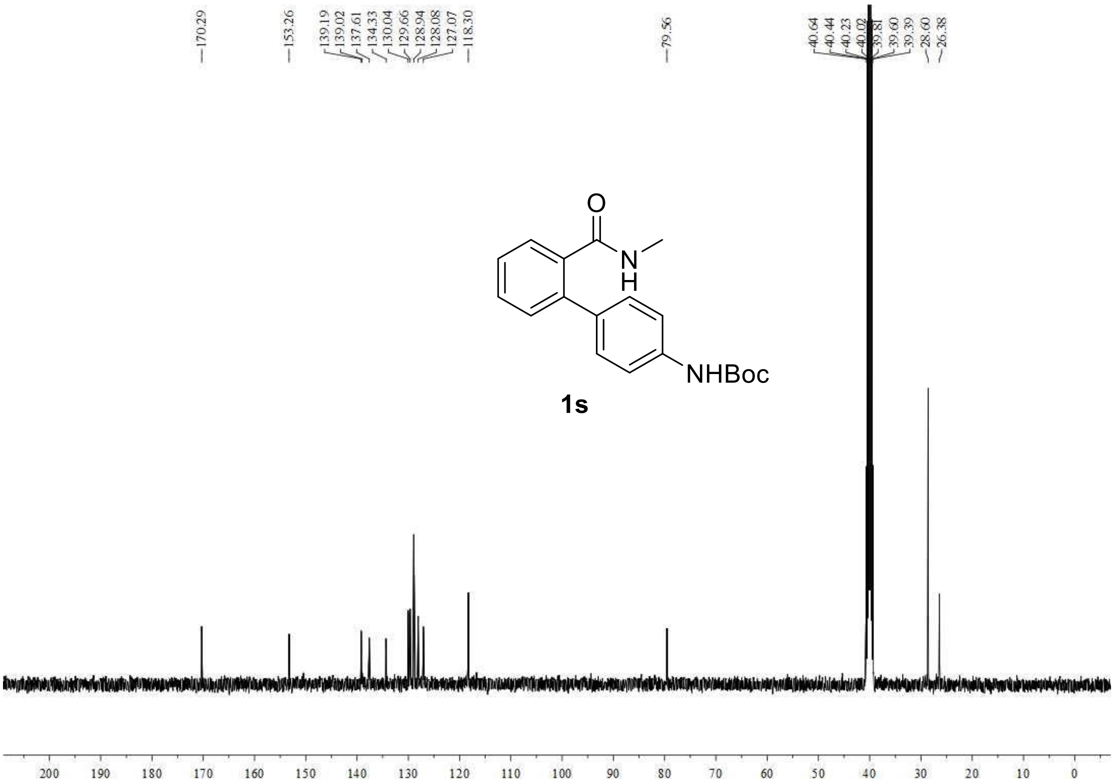

Supplementary Figure 49. ^{13}C NMR Spectrum of substrate 1s

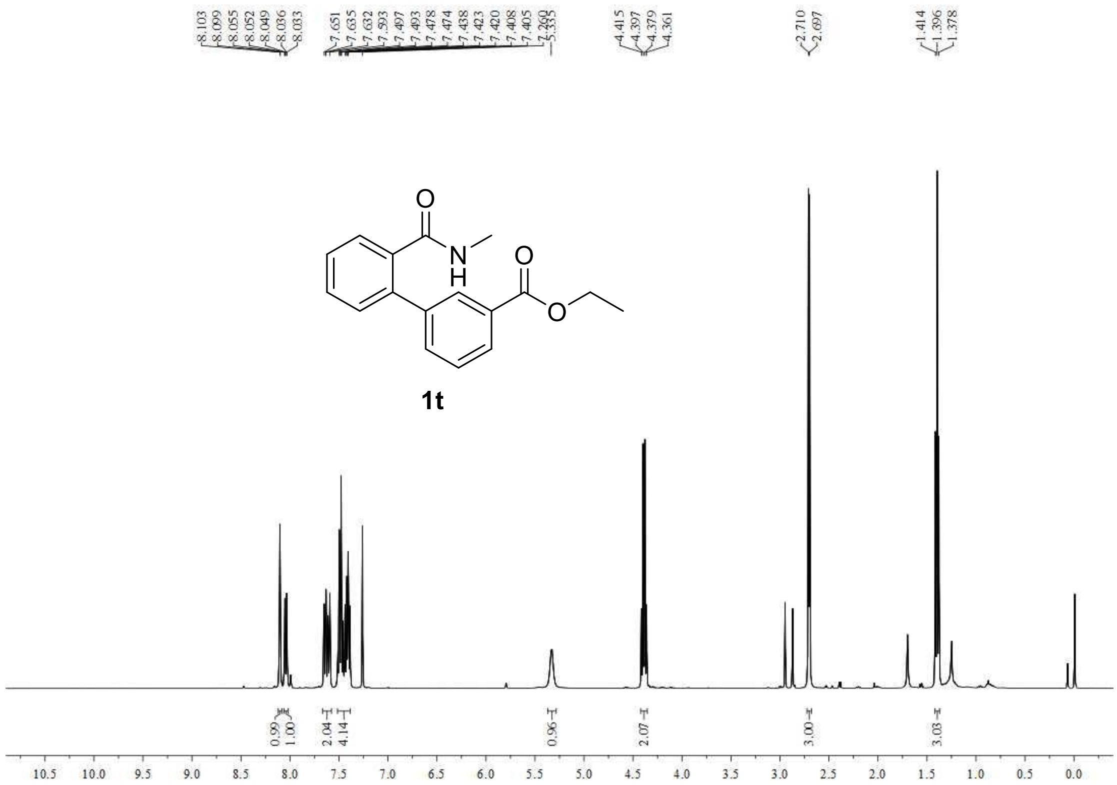

Supplementary Figure 50. ^{1}H NMR Spectrum of substrate 1t

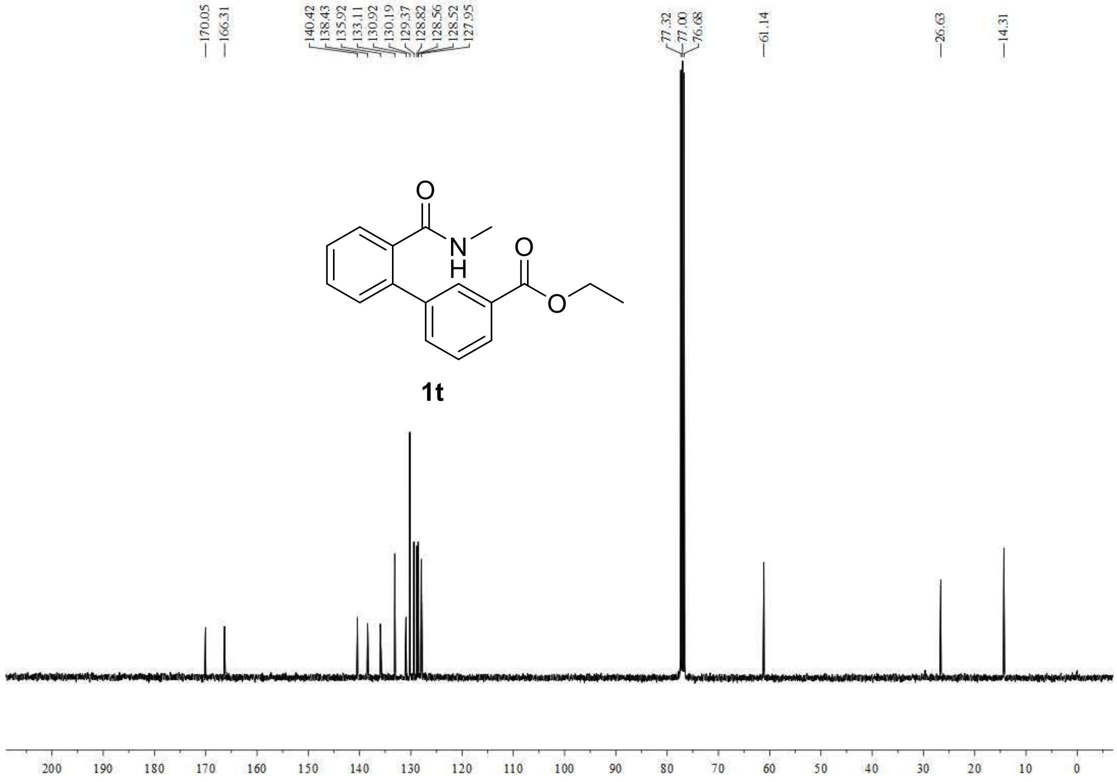

Supplementary Figure 51. ^{13}C NMR Spectrum of substrate 1t

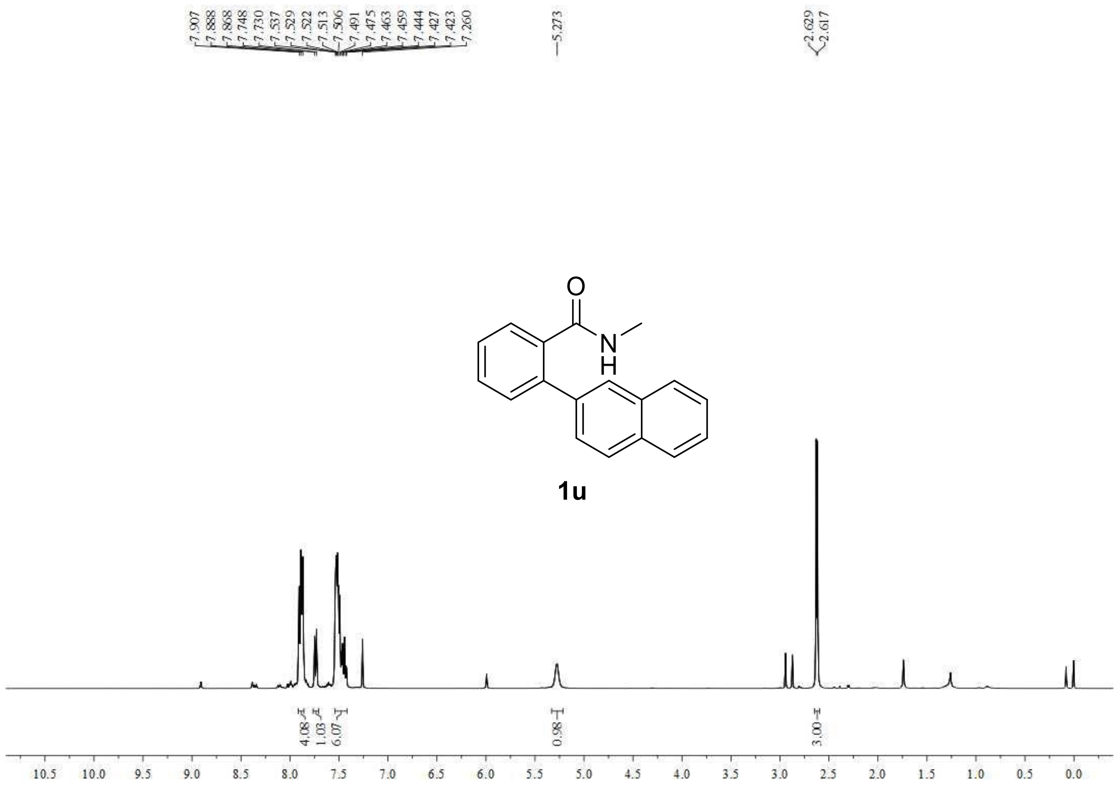

Supplementary Figure 52. ^{1}H NMR Spectrum of substrate 1u

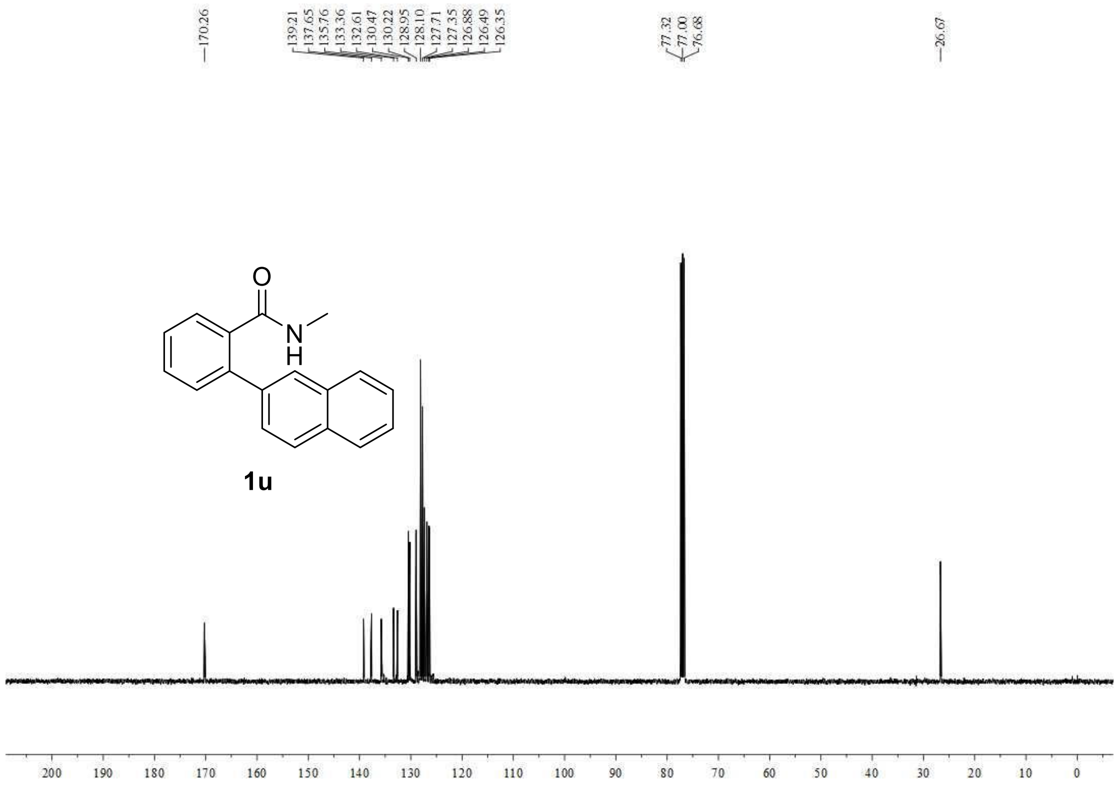

Supplementary Figure 53. ^{13}C NMR Spectrum of substrate 1u

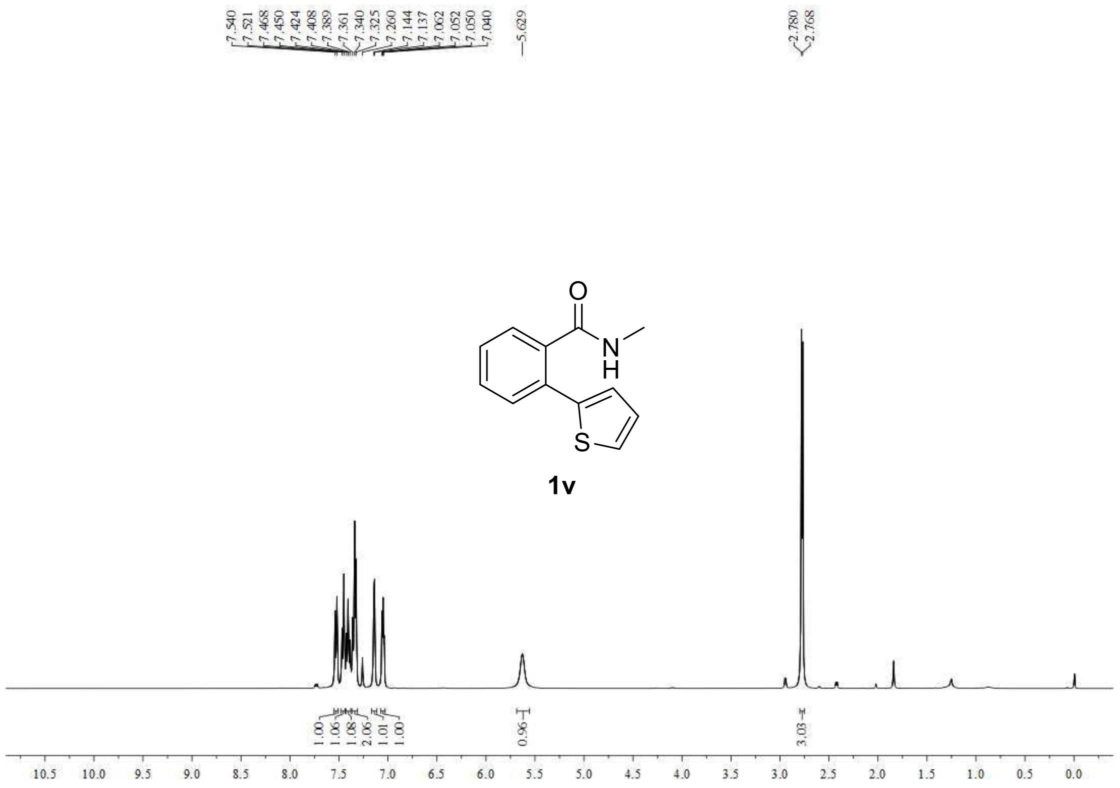

Supplementary Figure 54. ^{1}H NMR Spectrum of substrate 1v

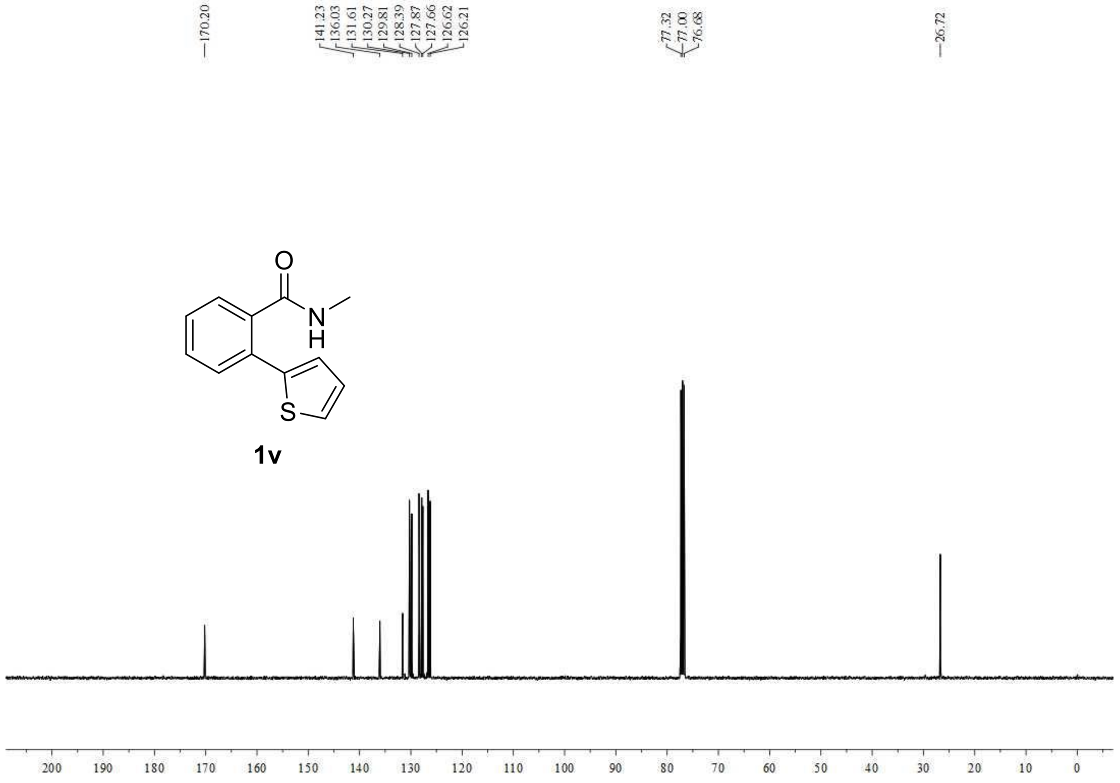

Supplementary Figure 55. ^{13}C NMR Spectrum of substrate 1v

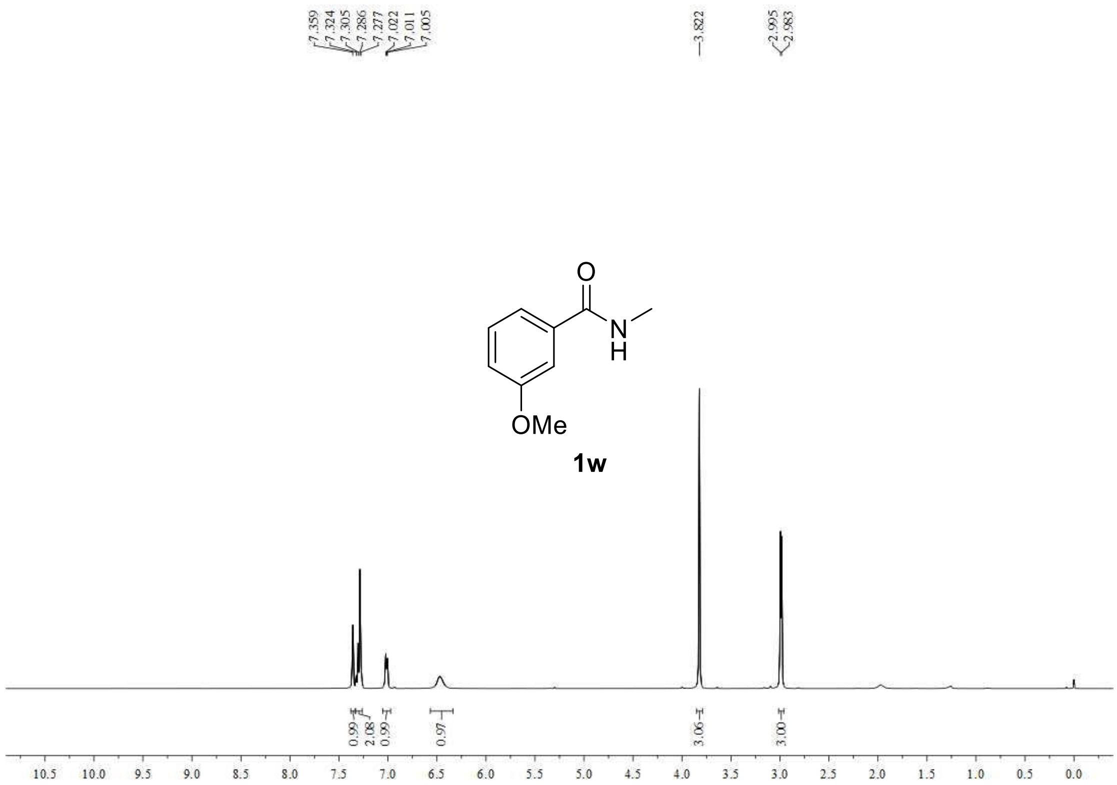

Supplementary Figure 56. ^{1}H NMR Spectrum of substrate 1w

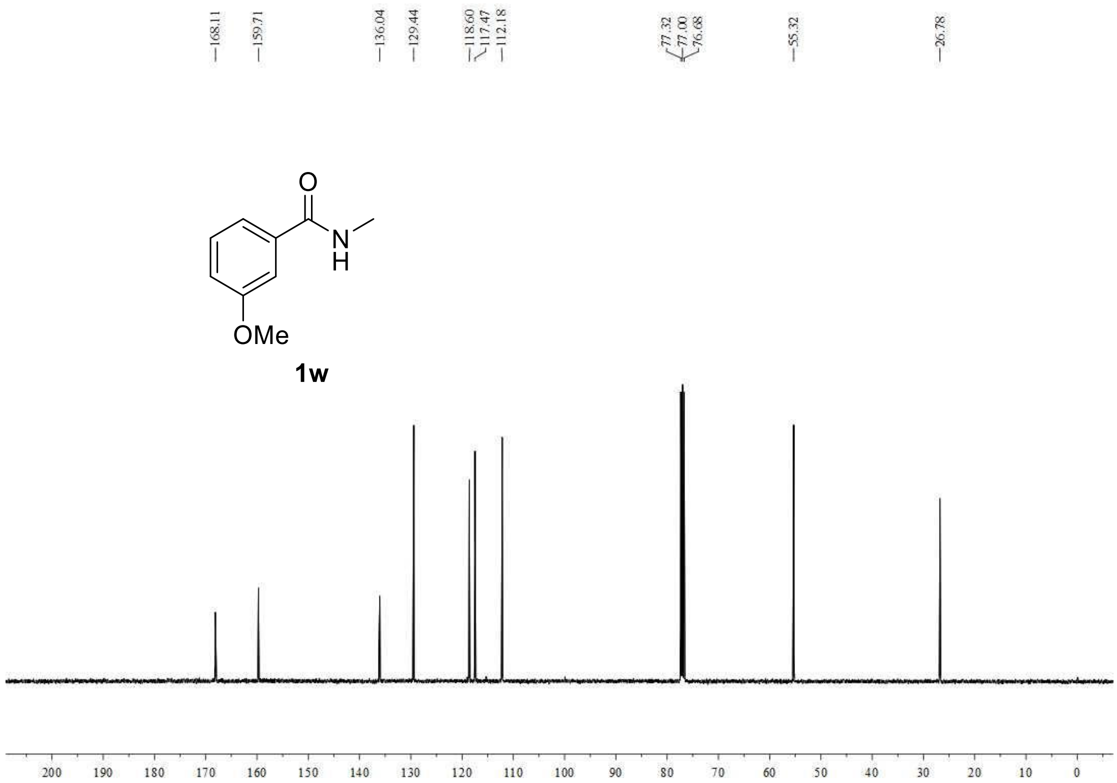

Supplementary Figure 57. ^{13}C NMR Spectrum of substrate 1w

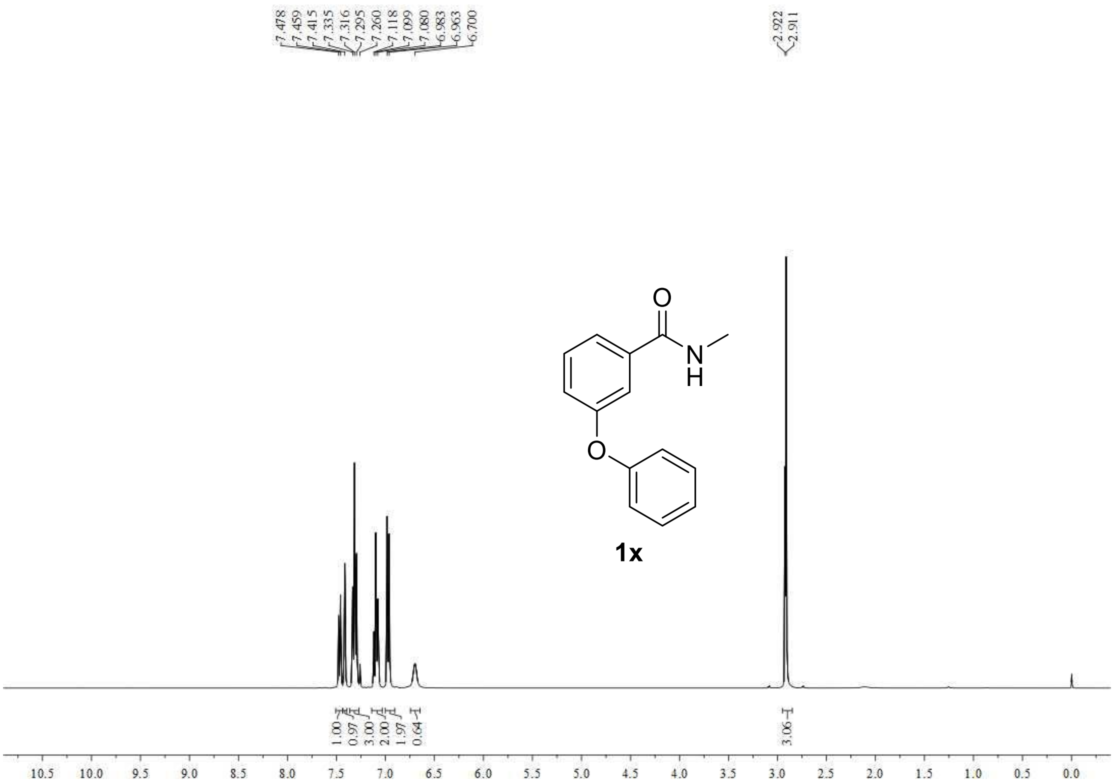

Supplementary Figure 58. ^{1}H NMR Spectrum of substrate 1x

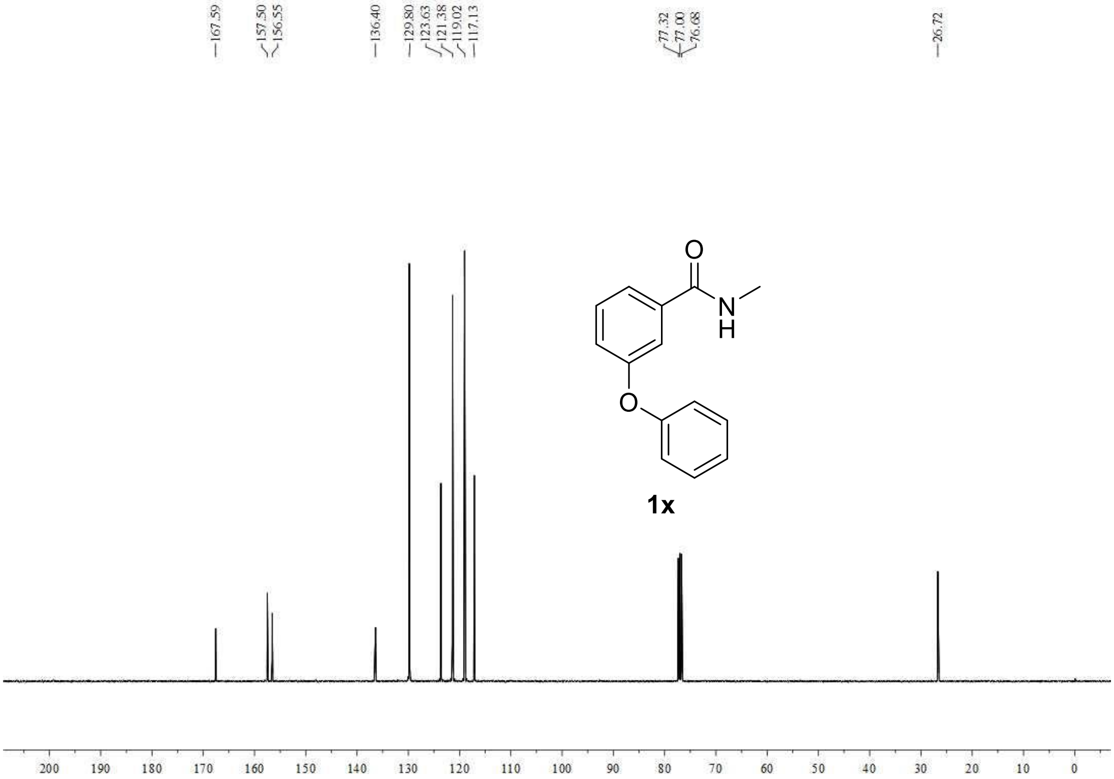

Supplementary Figure 59. ^{13}C NMR Spectrum of substrate 1x

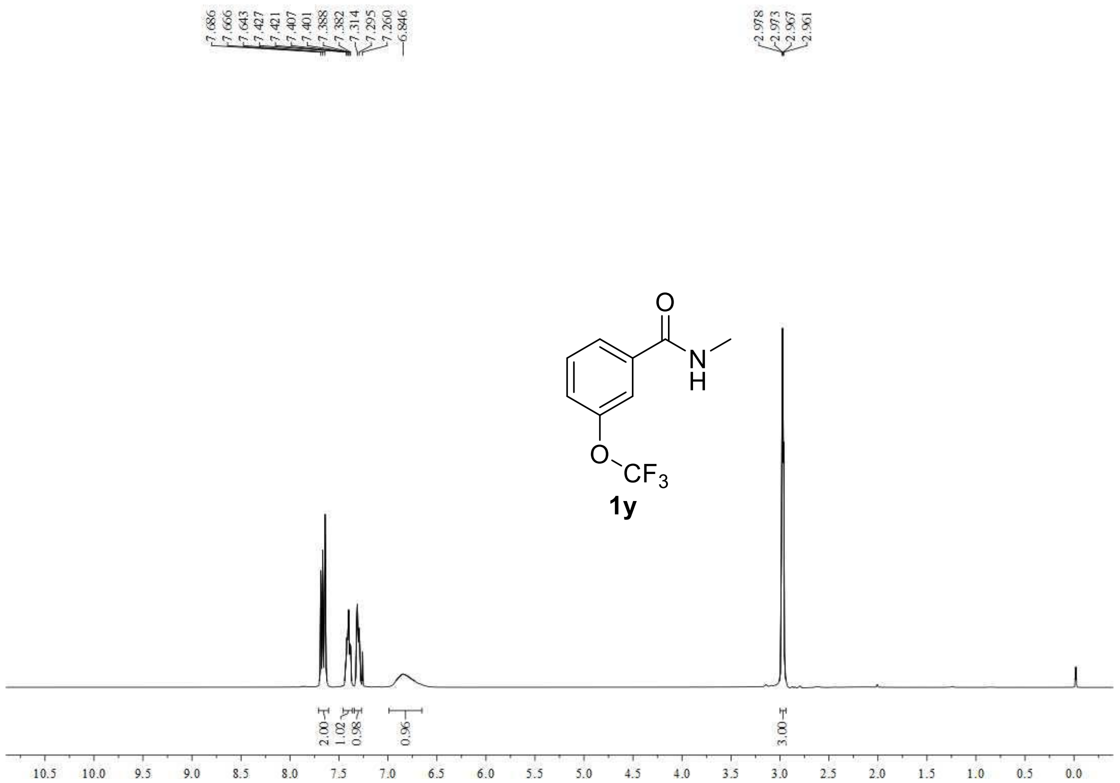

Supplementary Figure 60. ^{1}H NMR Spectrum of substrate 1y

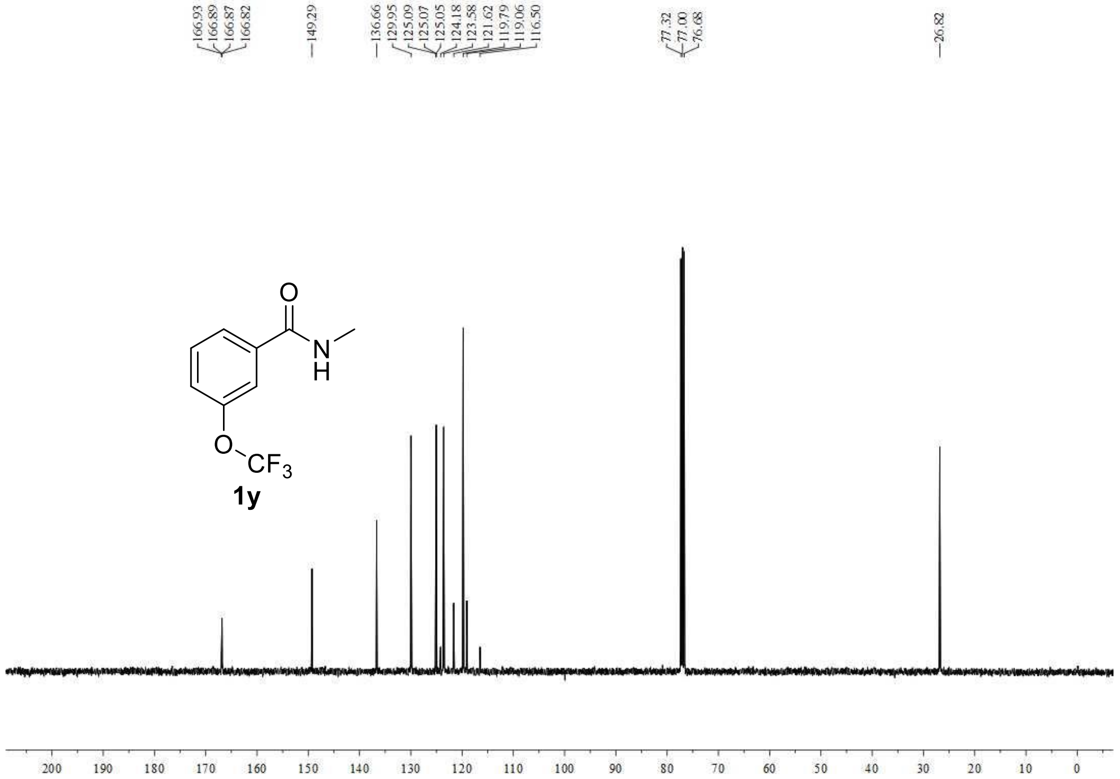

Supplementary Figure 61. ^{13}C NMR Spectrum of substrate 1y

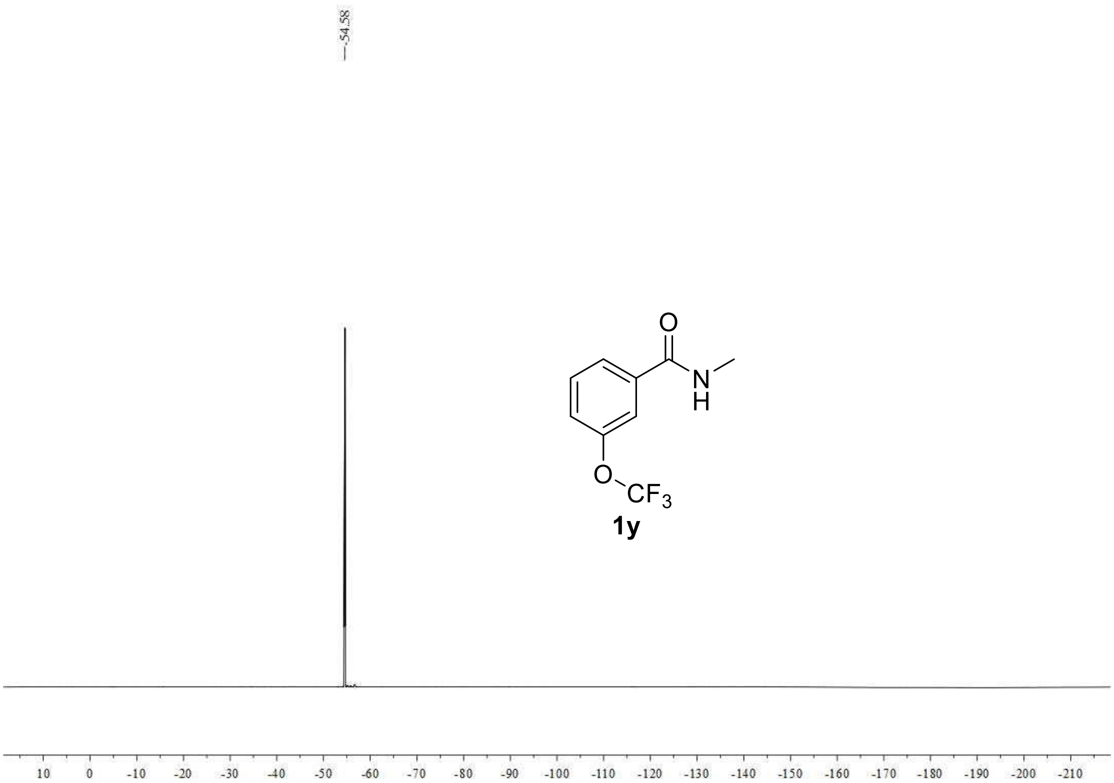

Supplementary Figure 62. ^{19}F NMR Spectrum of substrate 1y

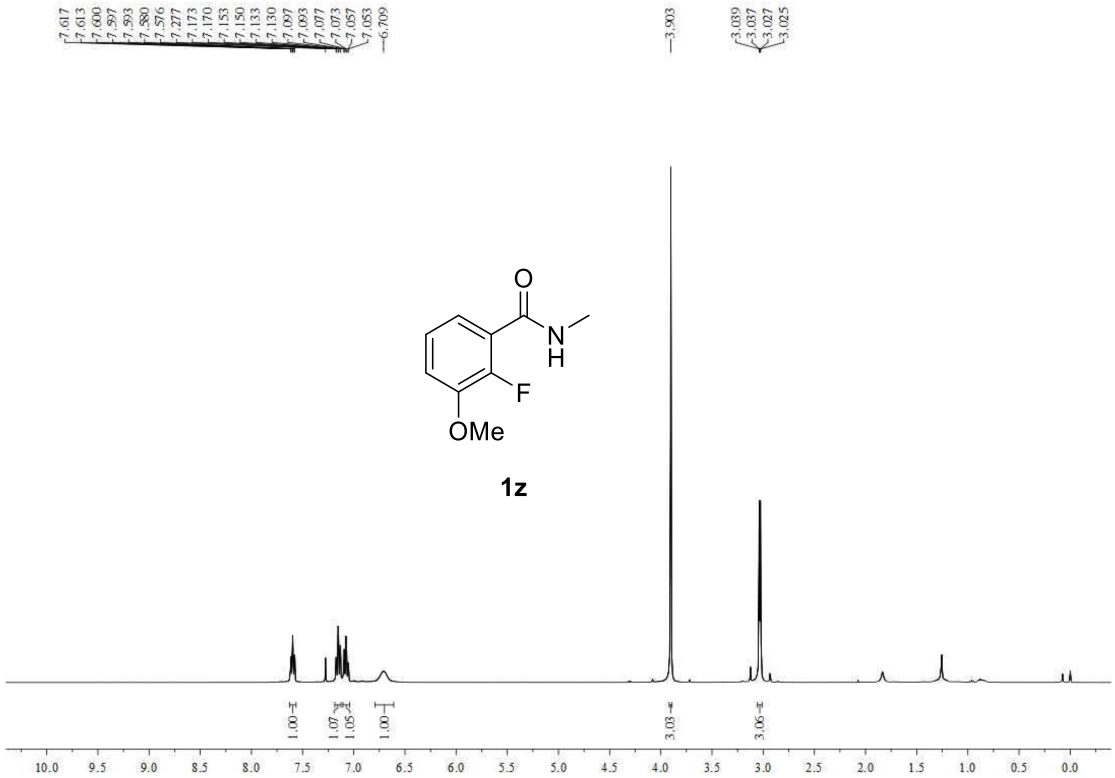

Supplementary Figure 63. ^{1}H NMR Spectrum of substrate 1z

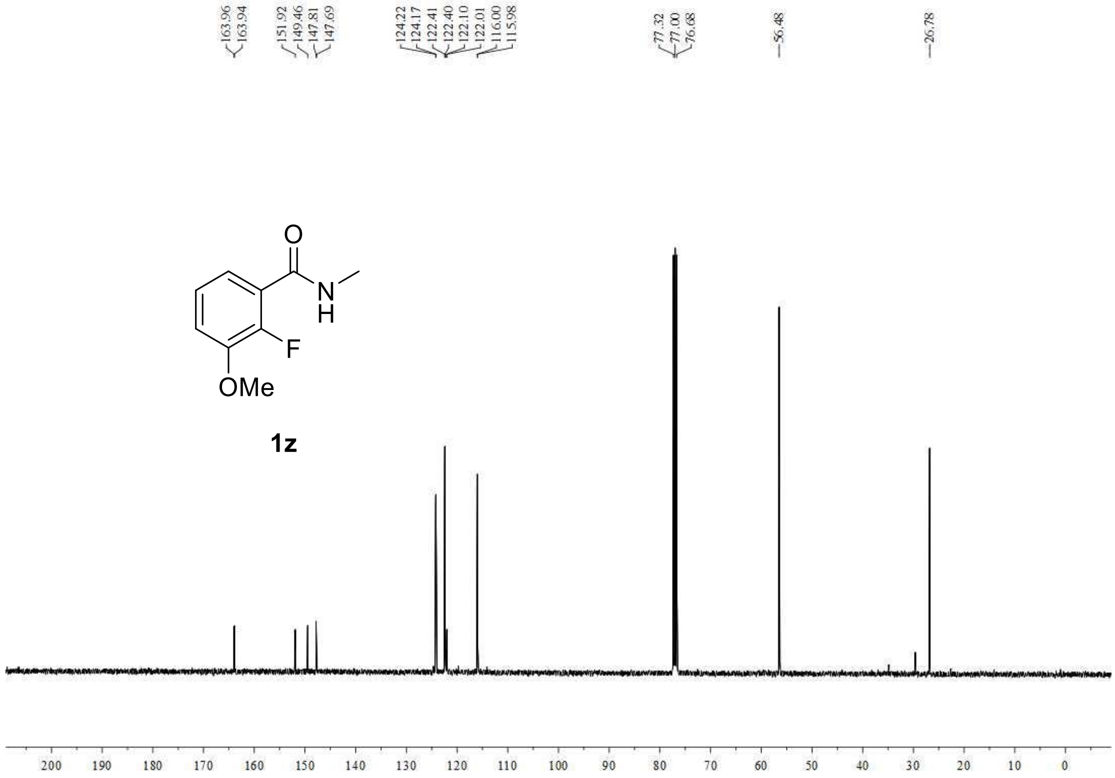

Supplementary Figure 64. ^{13}C NMR Spectrum of substrate 1z

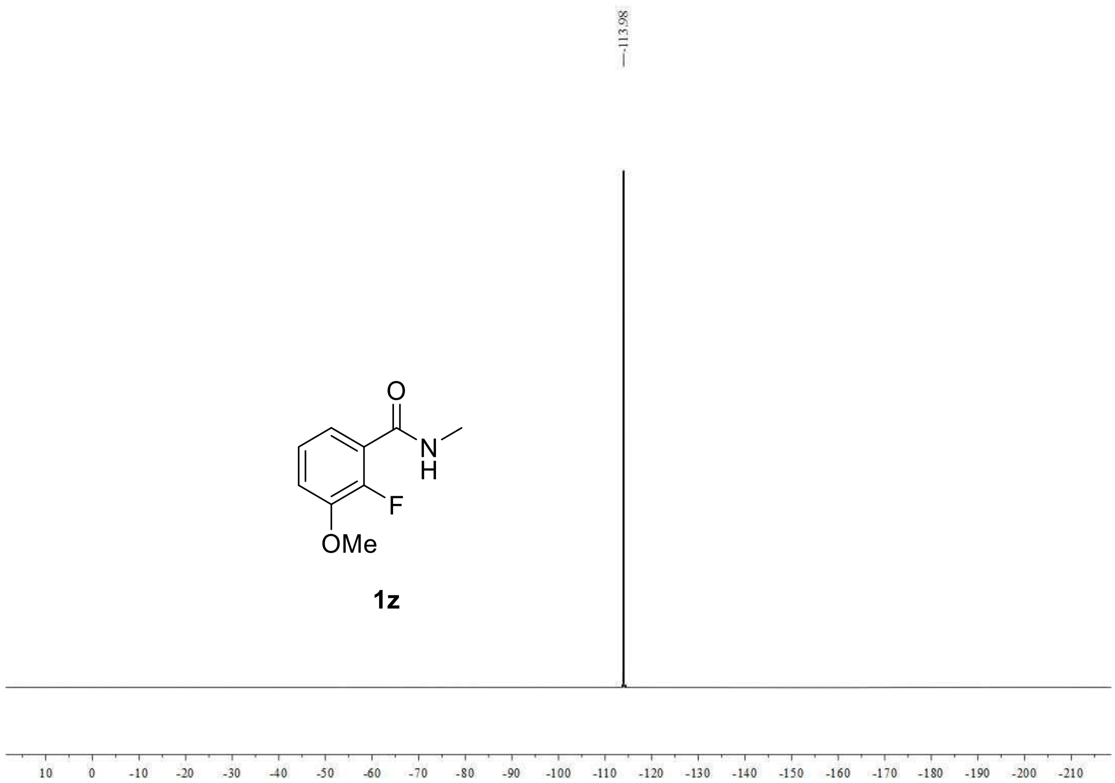

Supplementary Figure 65. ^{19}F NMR Spectrum of substrate 1z

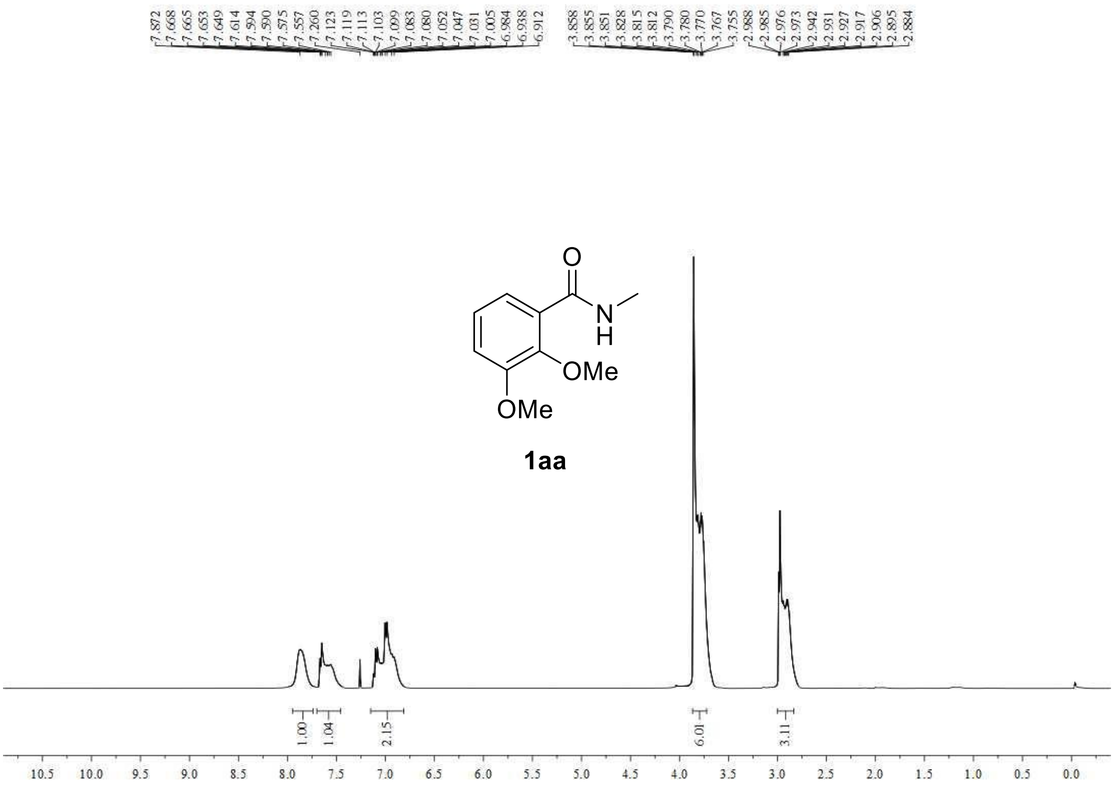

Supplementary Figure 66. ^{1}H NMR Spectrum of substrate 1aa

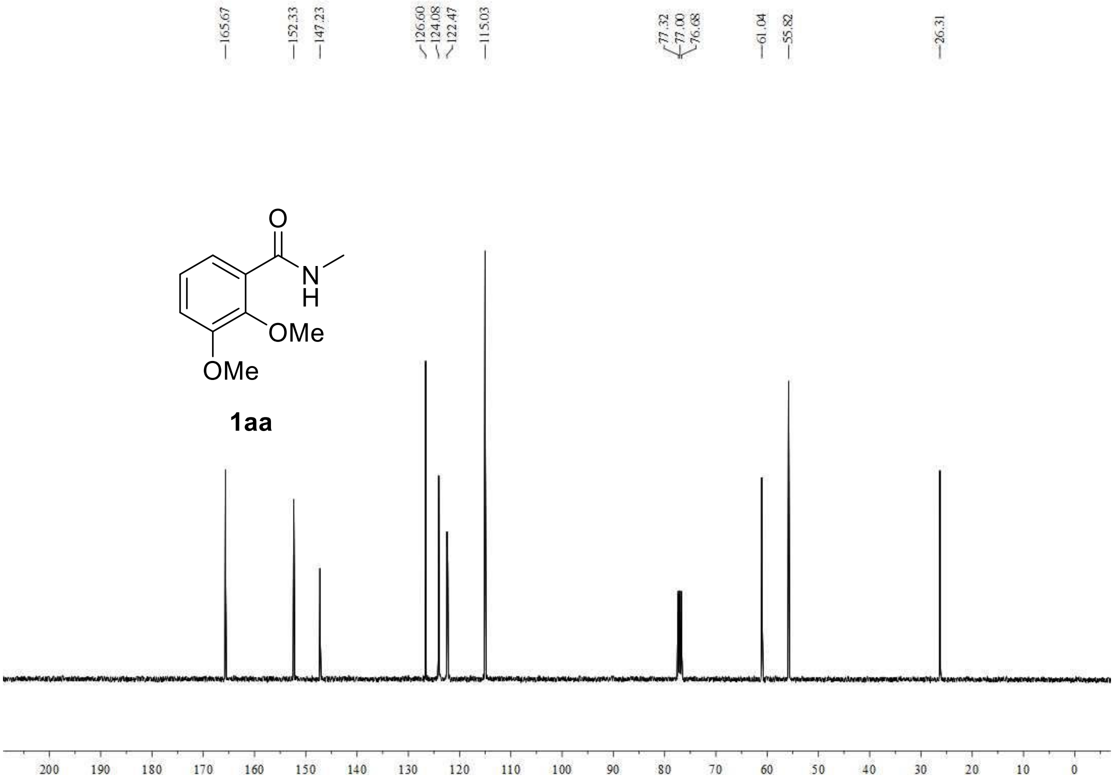

Supplementary Figure 67. ^{13}C NMR Spectrum of substrate 1aa

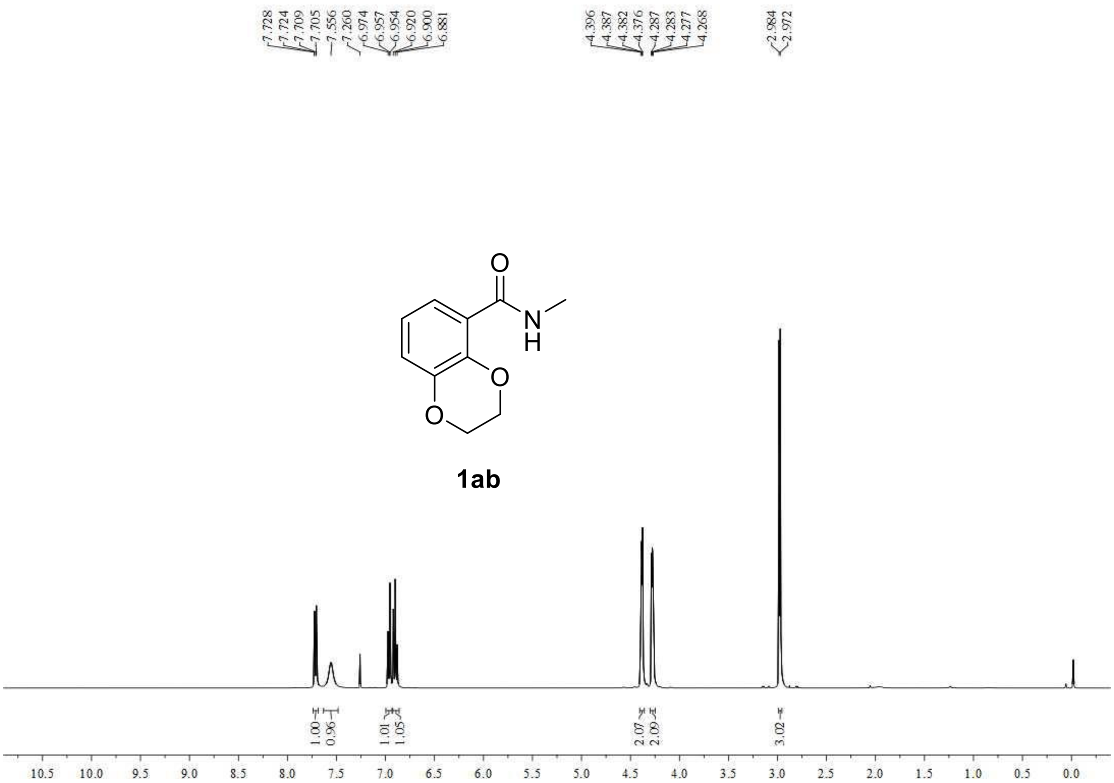

Supplementary Figure 68. ^{1}H NMR Spectrum of substrate 1ab

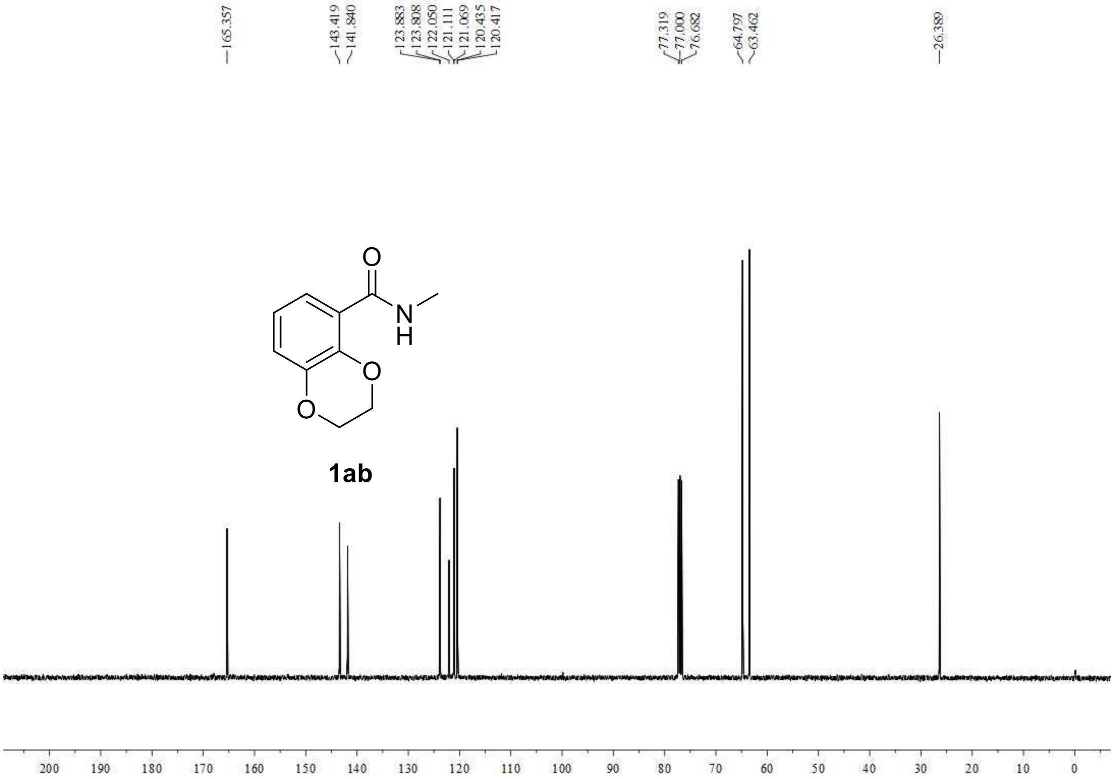

Supplementary Figure 69. ^{13}C NMR Spectrum of substrate 1ab

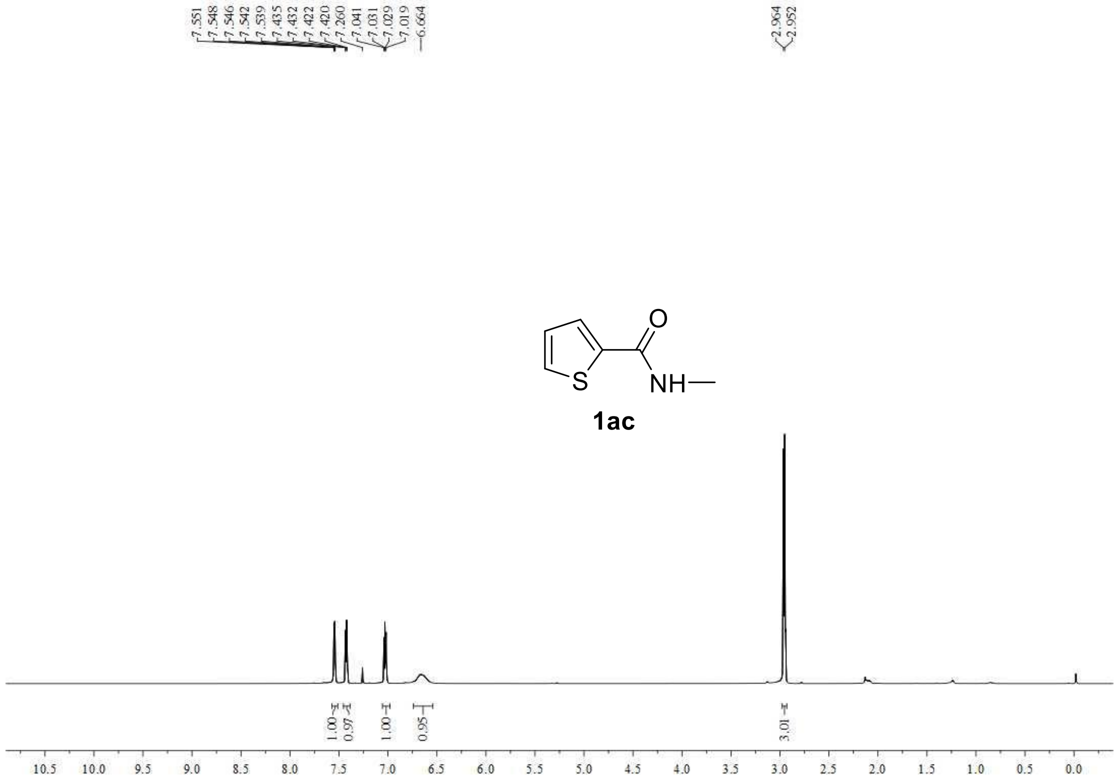

Supplementary Figure 70. ^{1}H NMR Spectrum of substrate 1ac

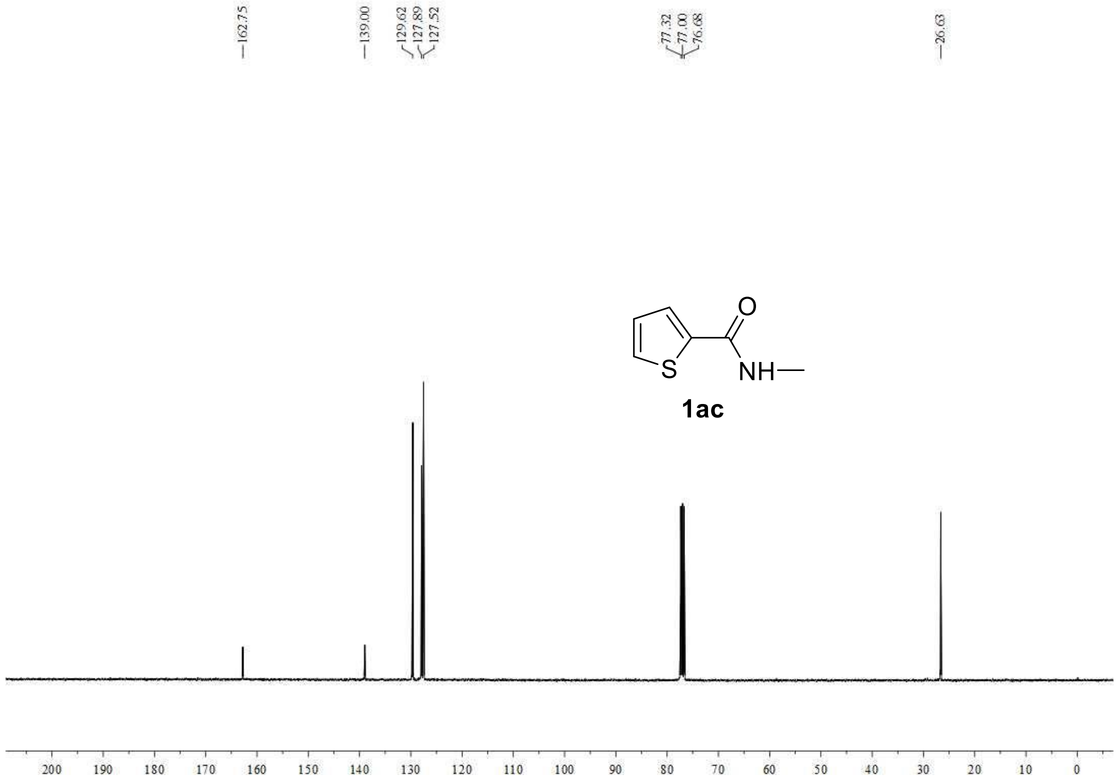

Supplementary Figure 71. ^{13}C NMR Spectrum of substrate 1ac

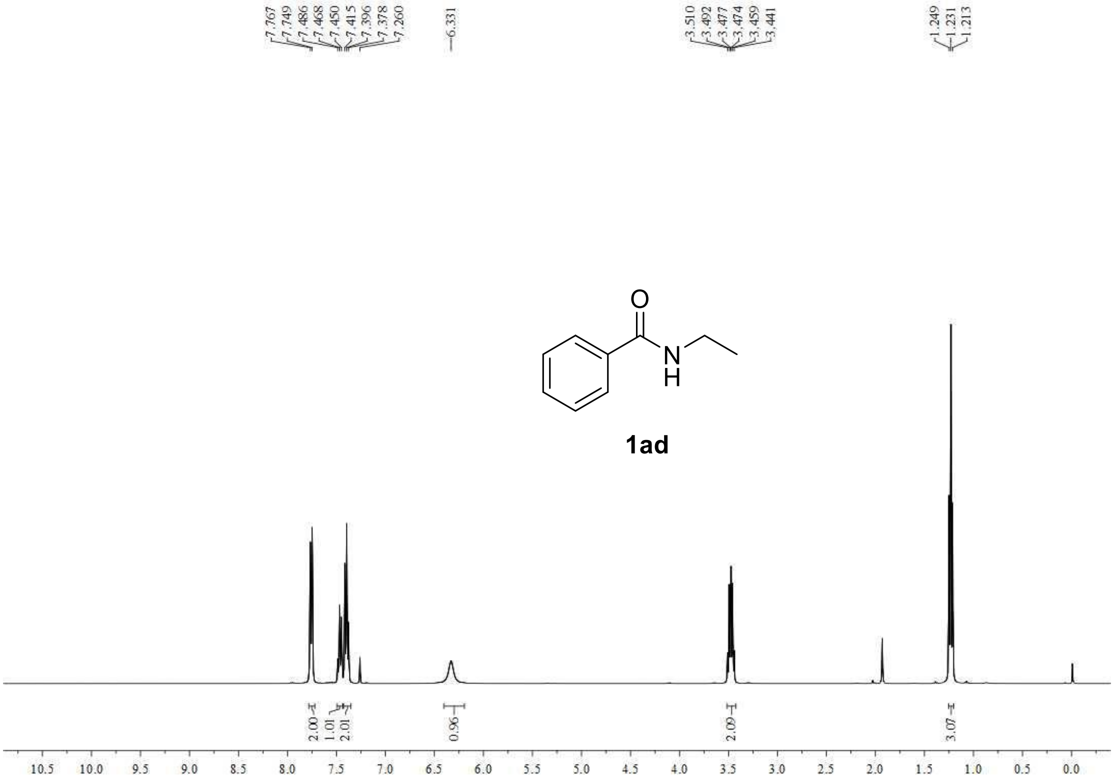

Supplementary Figure 72. ^{1}H NMR Spectrum of substrate 1ad

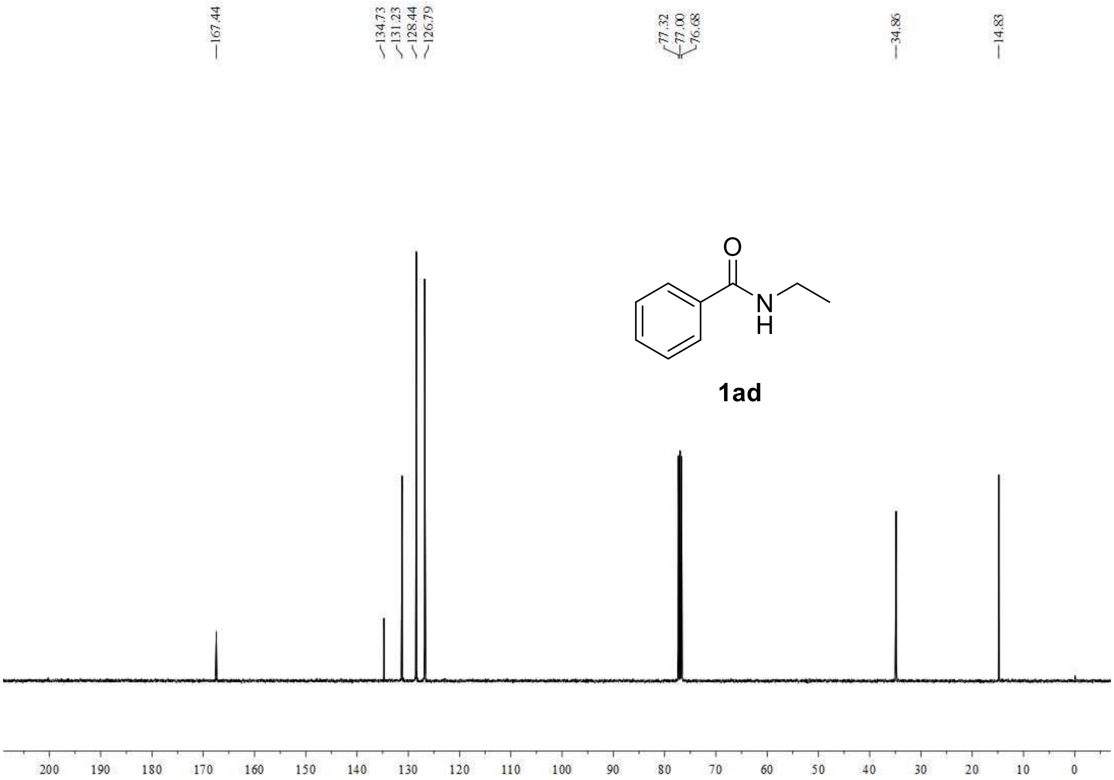

Supplementary Figure 73. ^{13}C NMR Spectrum of substrate 1ad

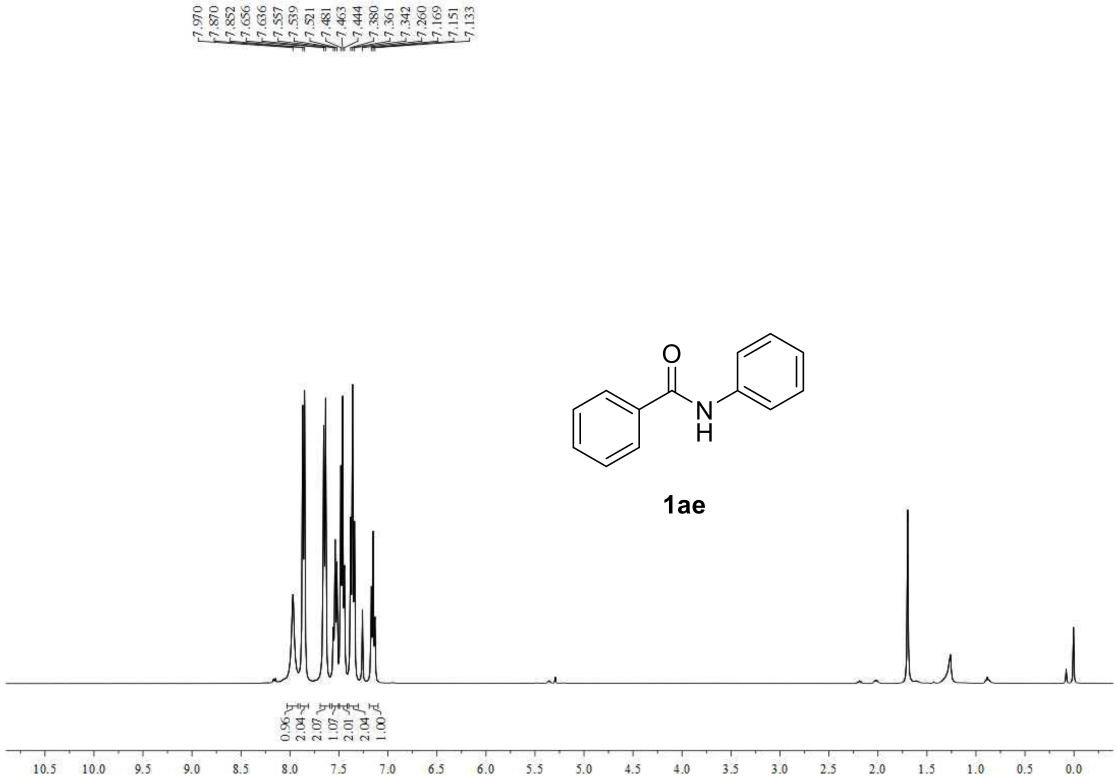

Supplementary Figure 74. ^{1}H NMR Spectrum of substrate 1ae

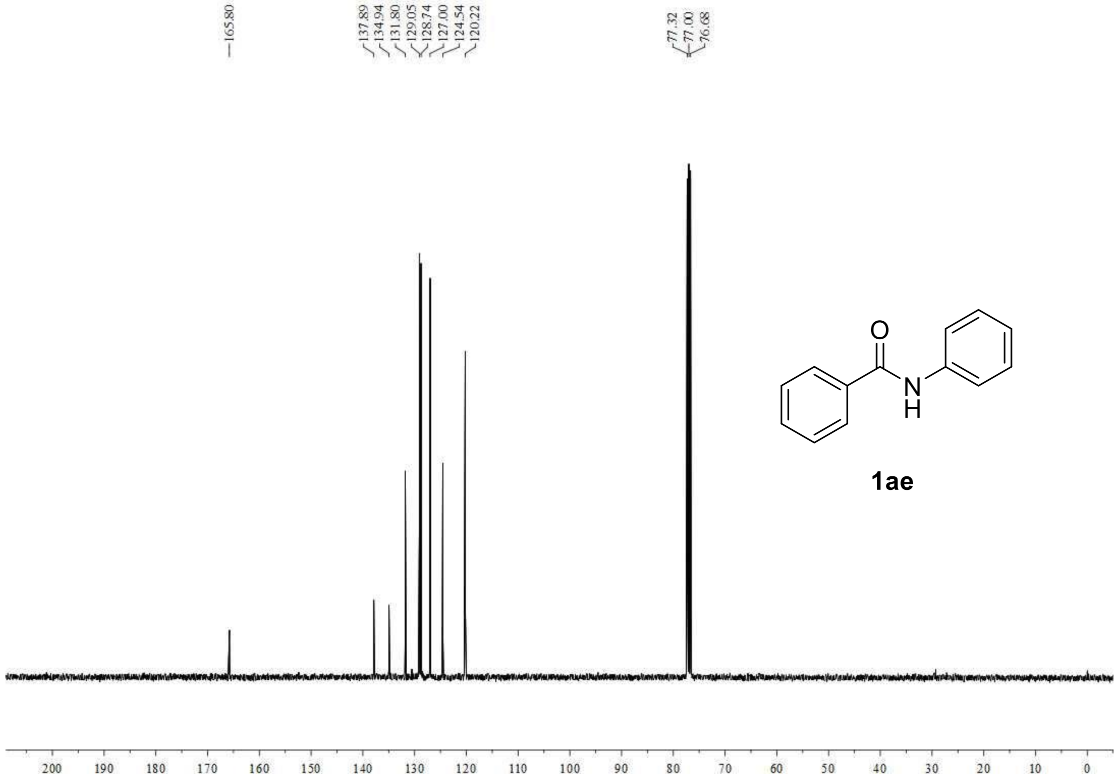

Supplementary Figure 75. ^{13}C NMR Spectrum of substrate 1ae

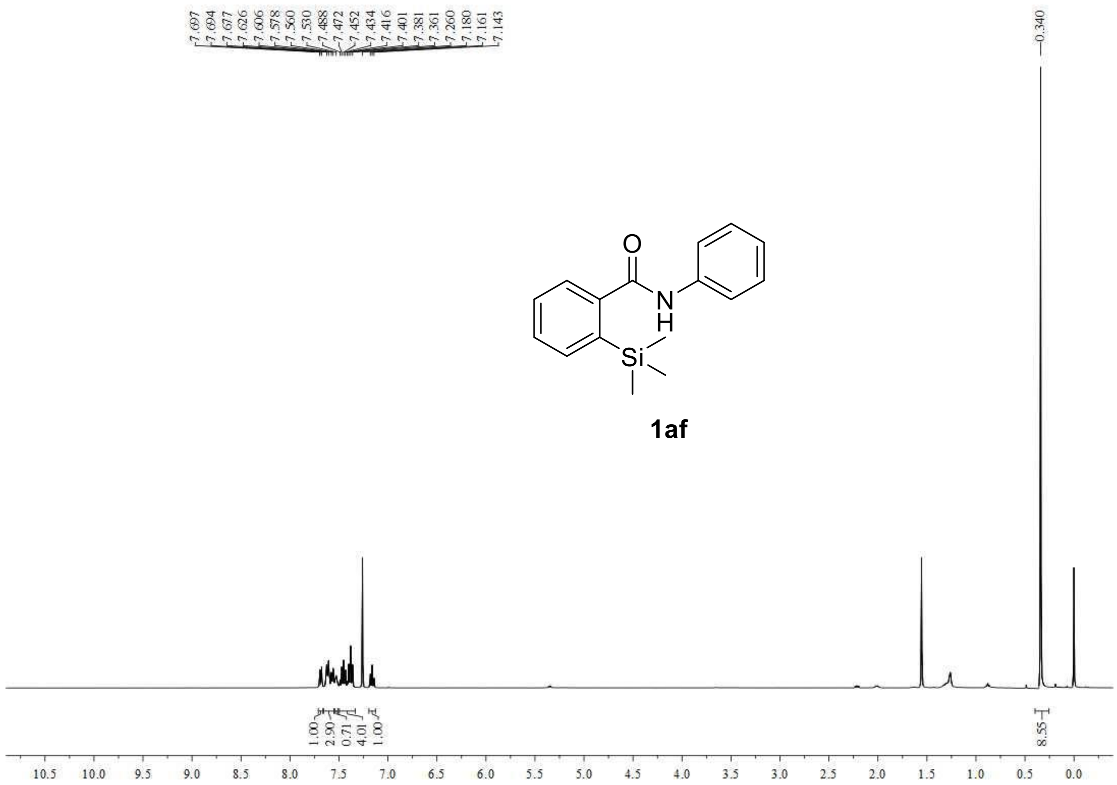

Supplementary Figure 76. ^{1}H NMR Spectrum of substrate 1af

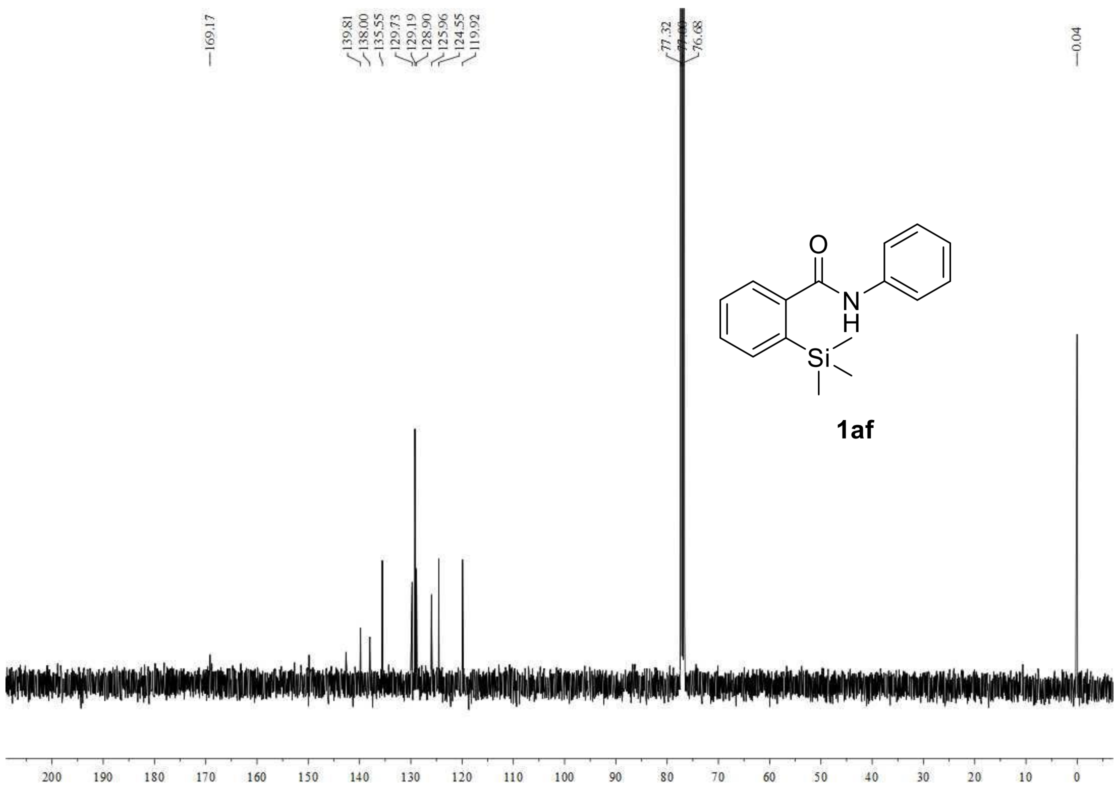

Supplementary Figure 77. ^{13}C NMR Spectrum of substrate 1af

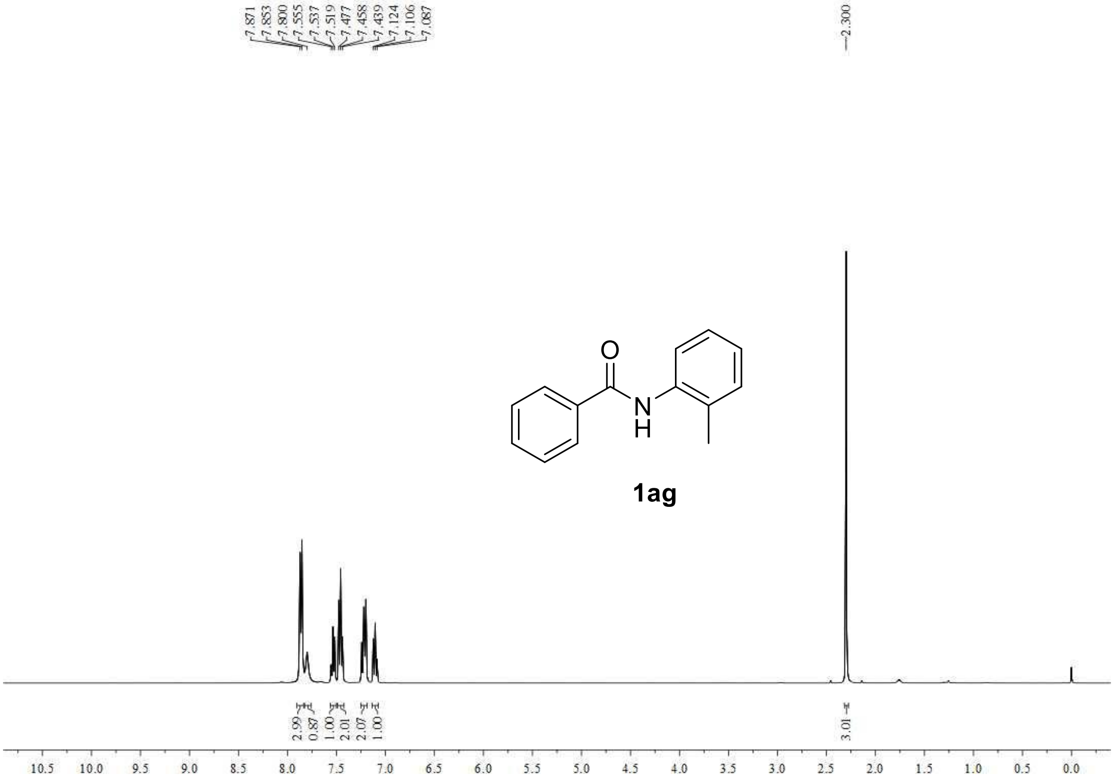

Supplementary Figure 78. ^{1}H NMR Spectrum of substrate 1ag

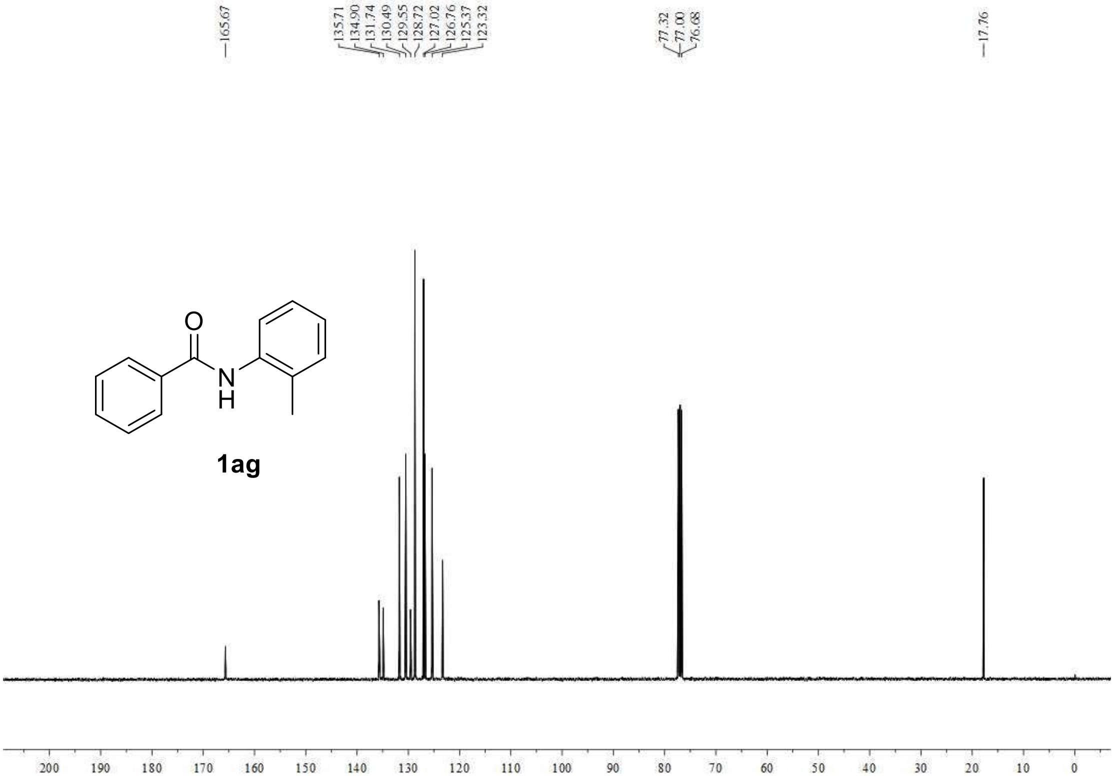

Supplementary Figure 79. ^{13}C NMR Spectrum of substrate 1ag

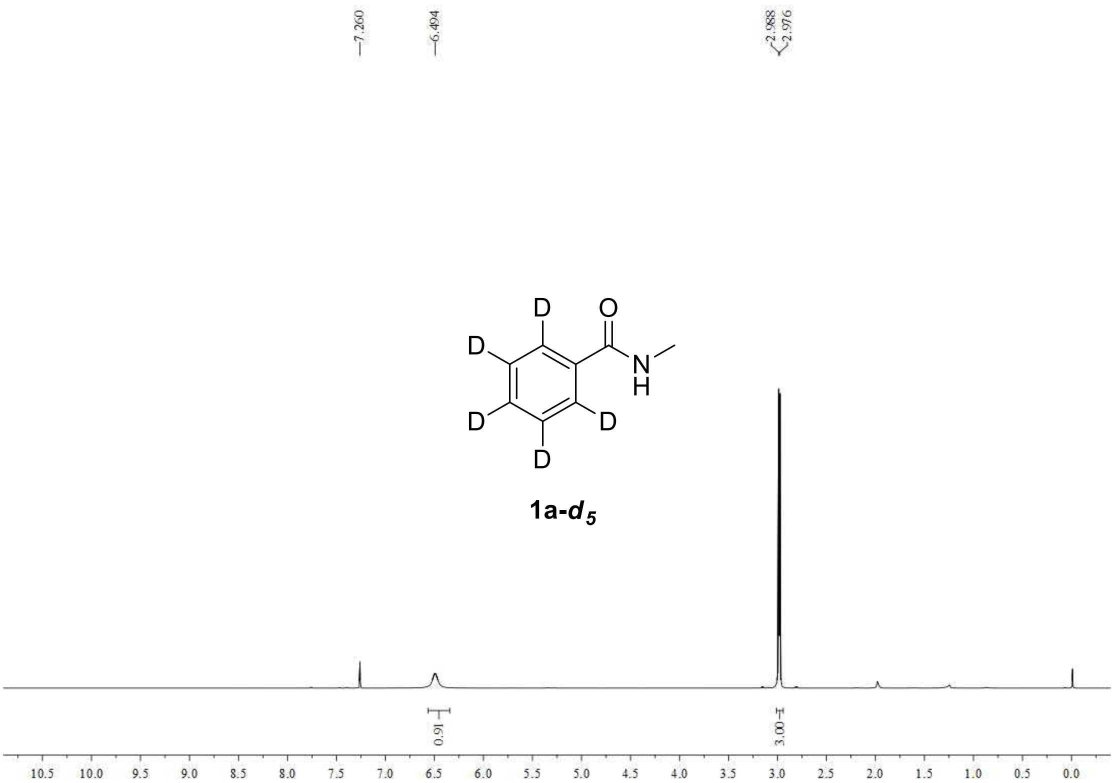

Supplementary Figure 80. ^{1}H NMR Spectrum of substrate 1a-d_5

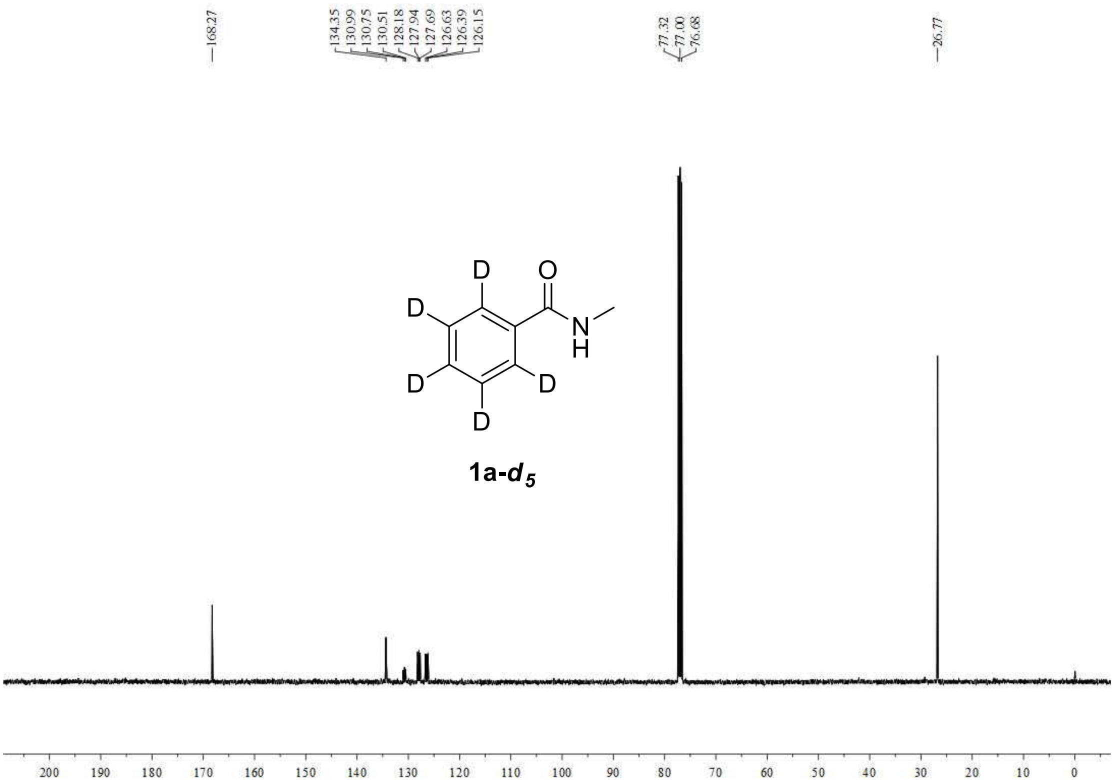

Supplementary Figure 81. ^{13}C NMR Spectrum of substrate 1a-d_5

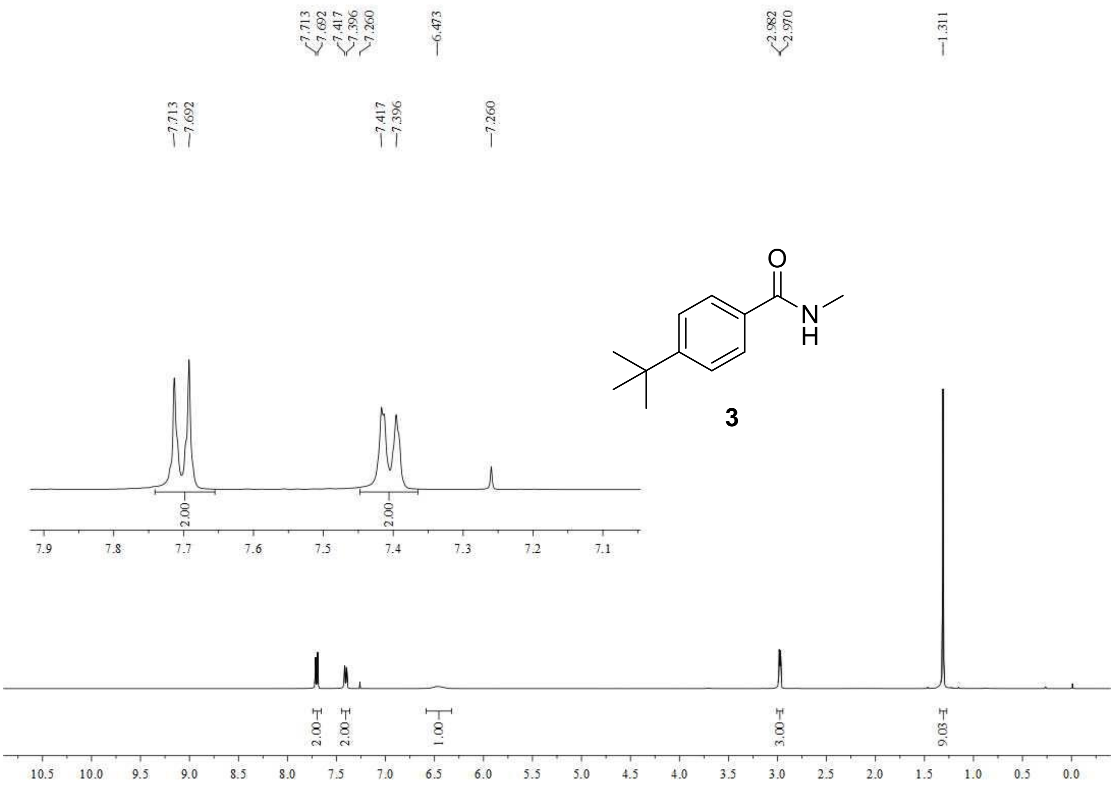

Supplementary Figure 82. ^{1}H NMR Spectrum of substrate 3

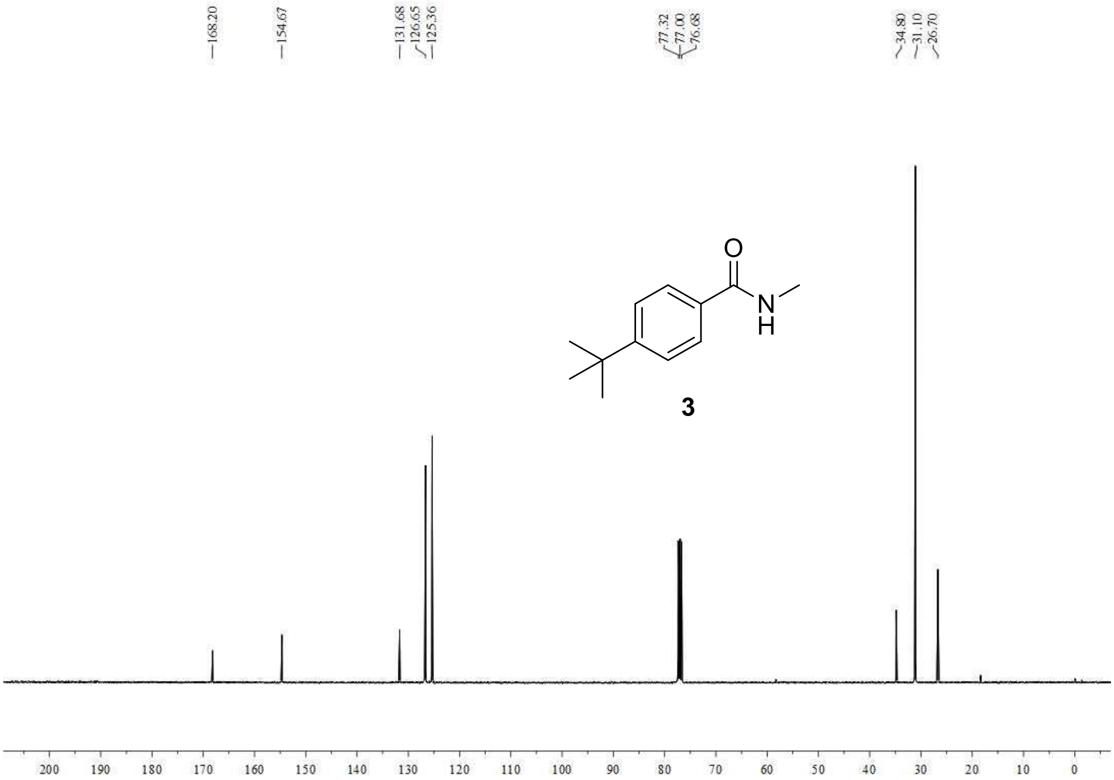

Supplementary Figure 83. ^{13}C NMR Spectrum of substrate 3

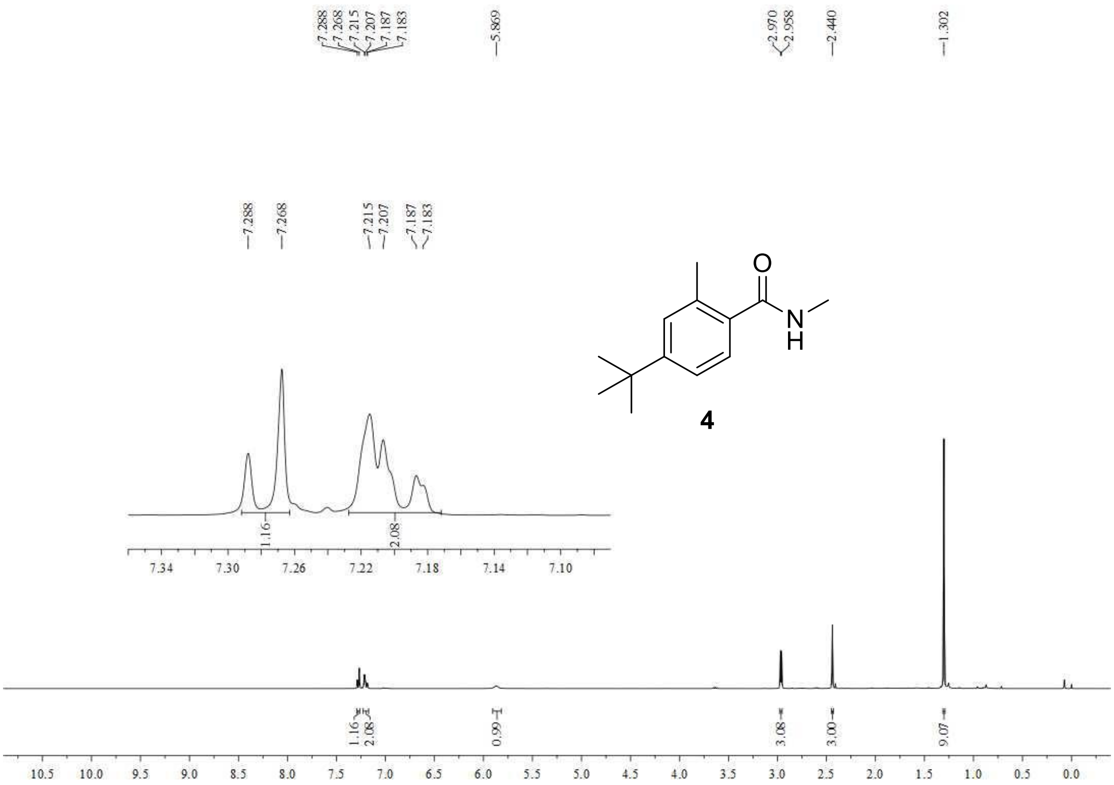

Supplementary Figure 84. ^{1}H NMR Spectrum of substrate 4

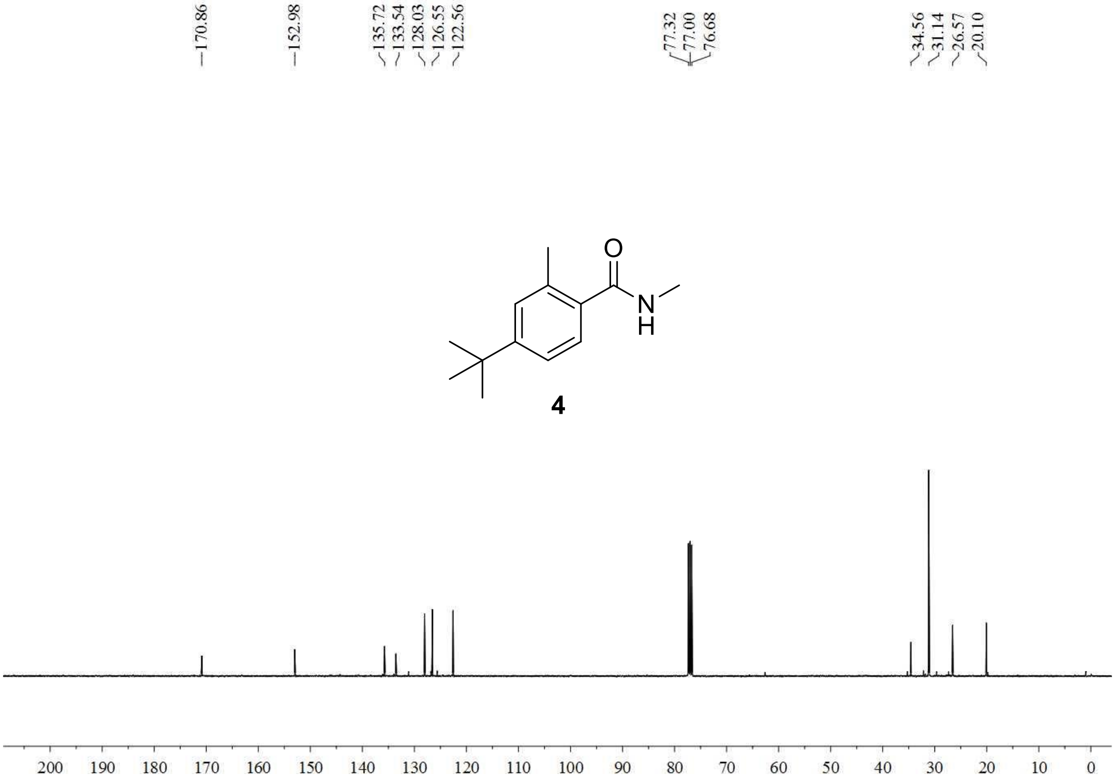

Supplementary Figure 85. ^{13}C NMR Spectrum of substrate 4

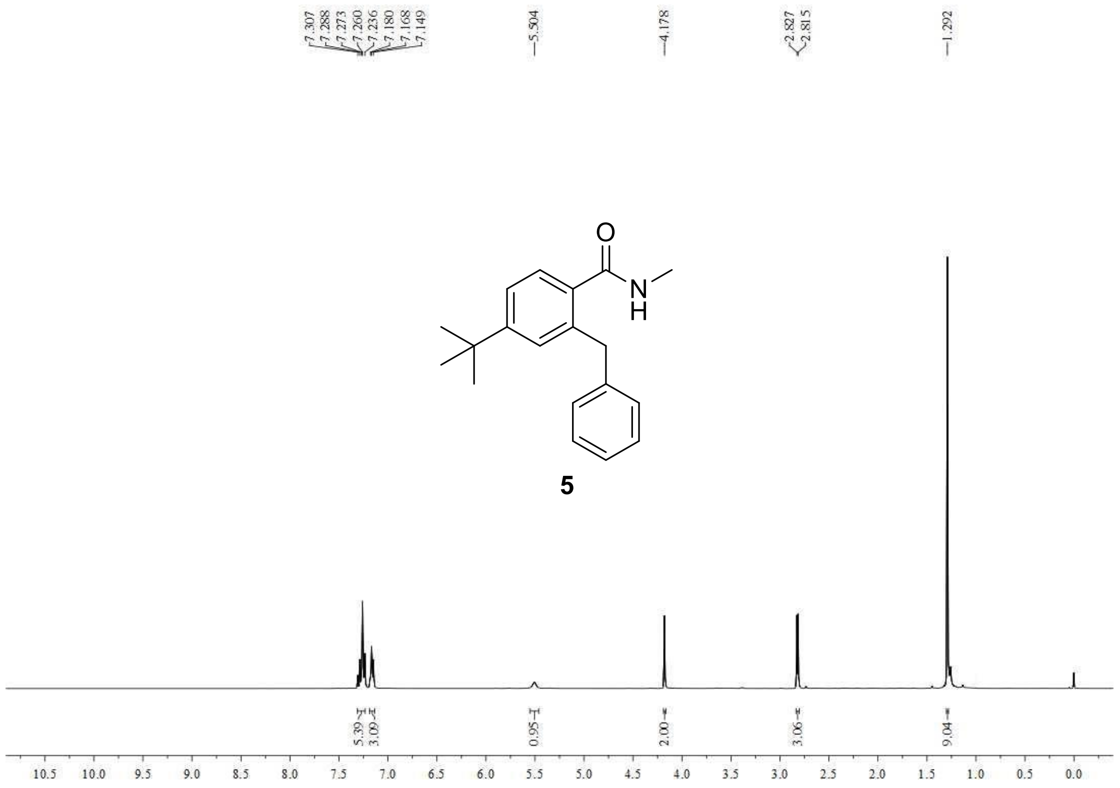

Supplementary Figure 86. ^{1}H NMR Spectrum of substrate 5

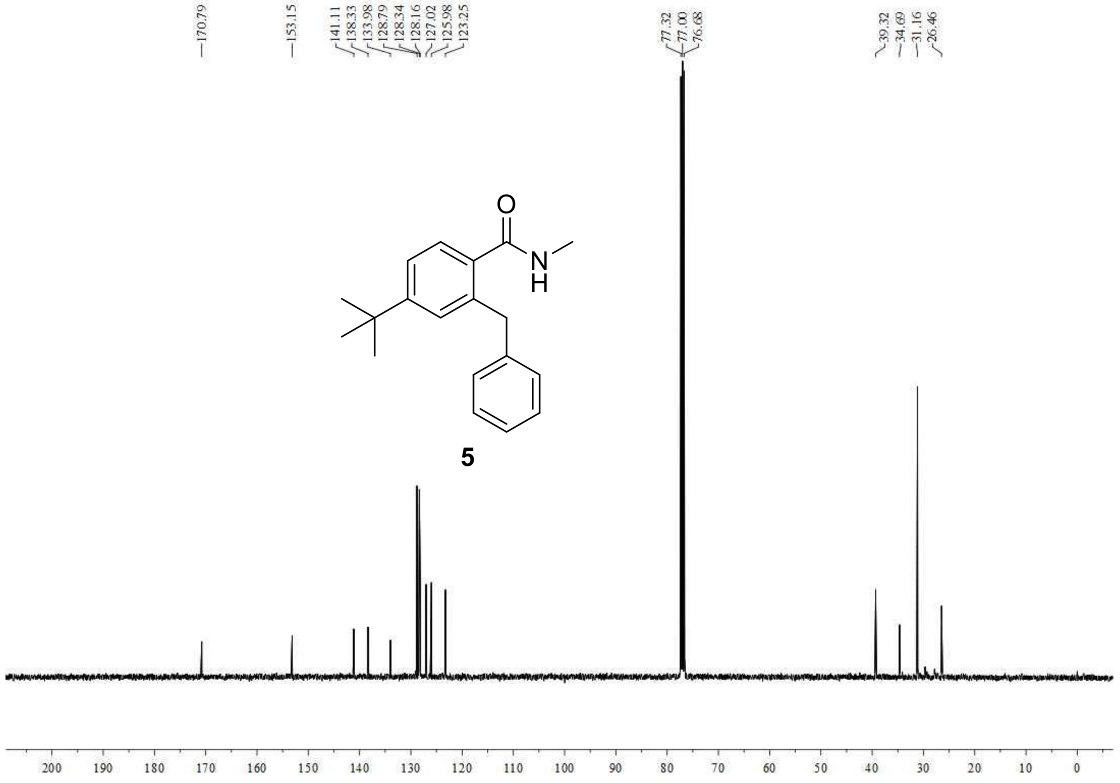

Supplementary Figure 87. ^{13}C NMR Spectrum of substrate 5

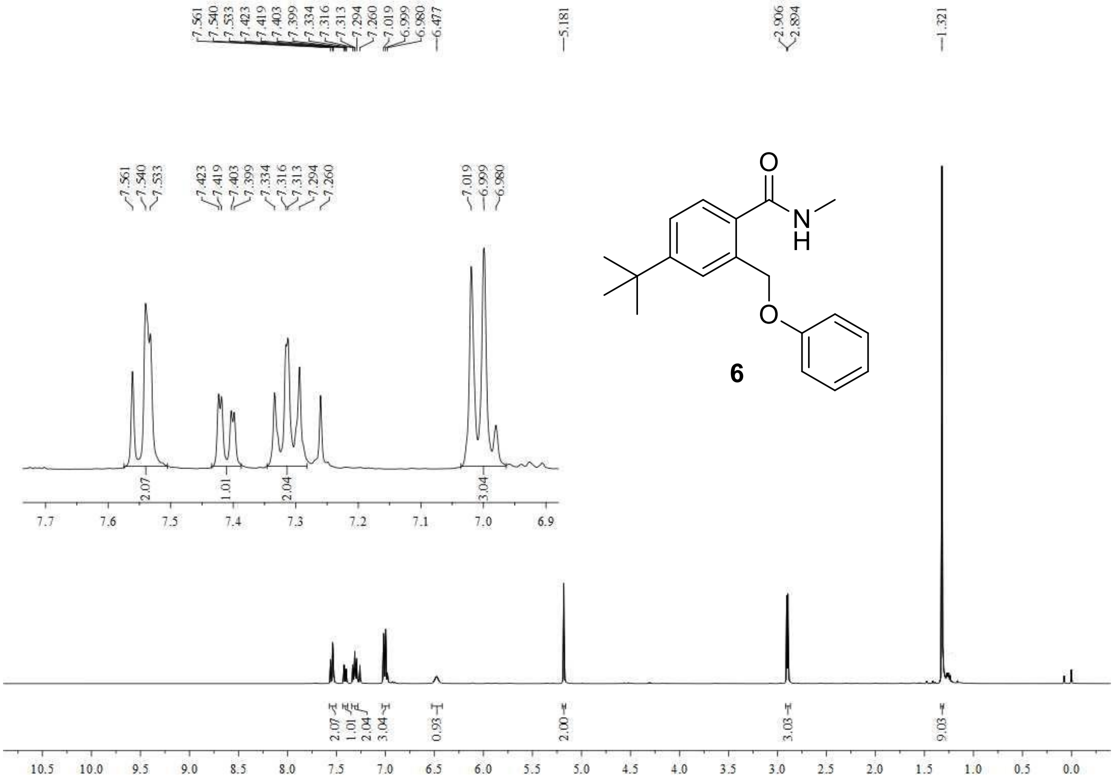

Supplementary Figure 88. ^{1}H NMR Spectrum of substrate 6

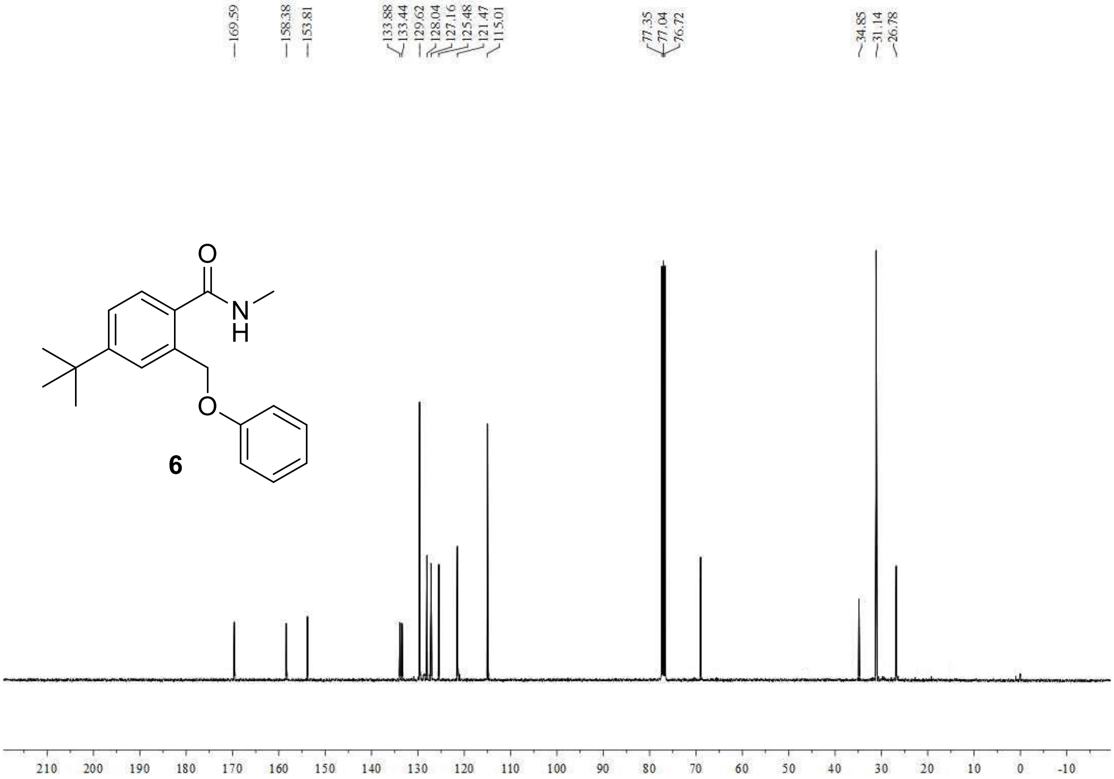

Supplementary Figure 89. ^{13}C NMR Spectrum of substrate 6

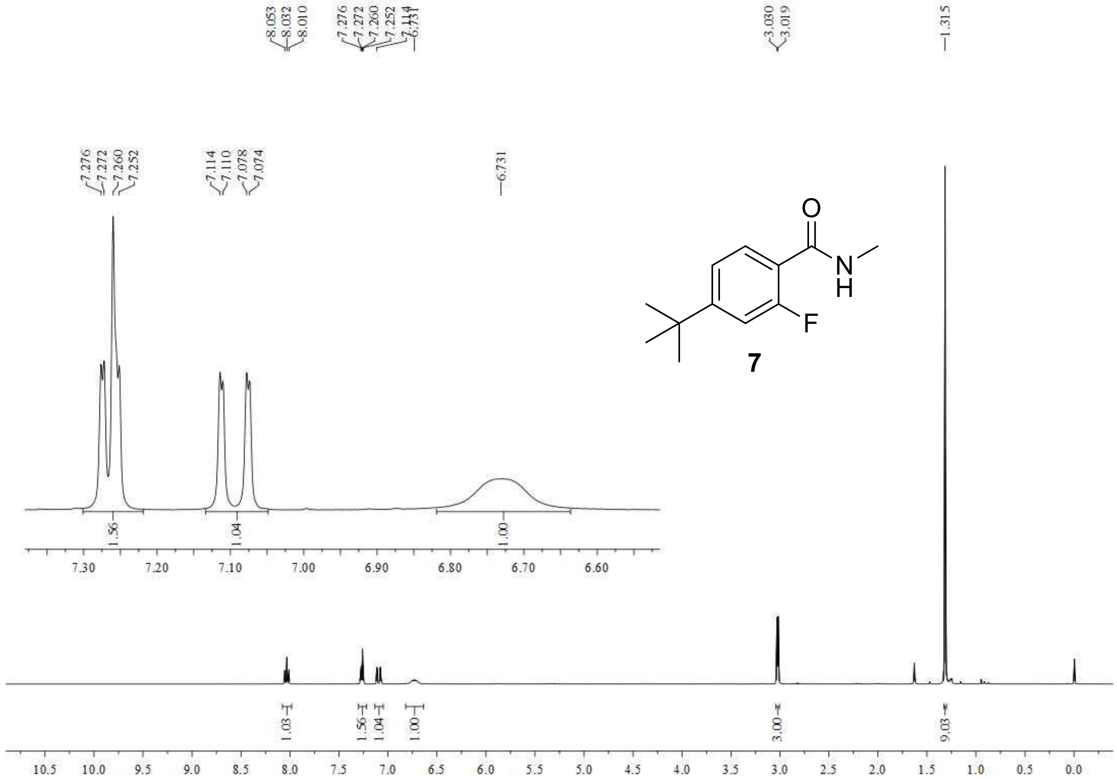

Supplementary Figure 90. ^{1}H NMR Spectrum of substrate 7

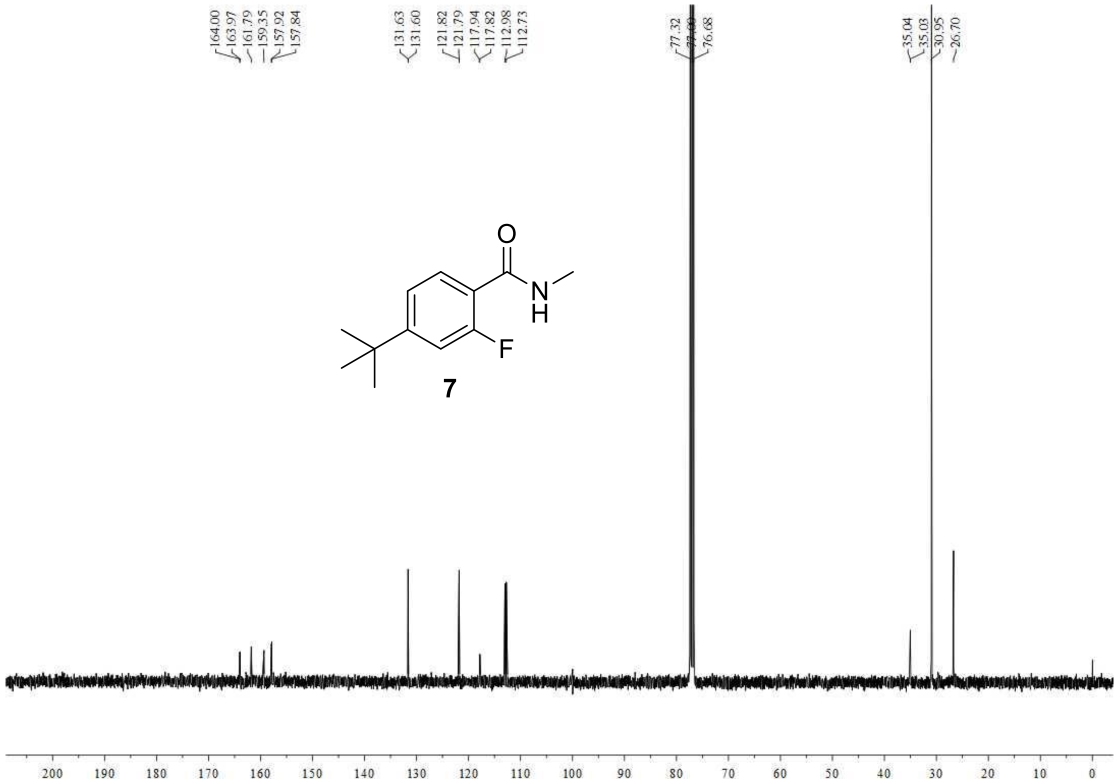

Supplementary Figure 91. ^{13}C NMR Spectrum of substrate 7

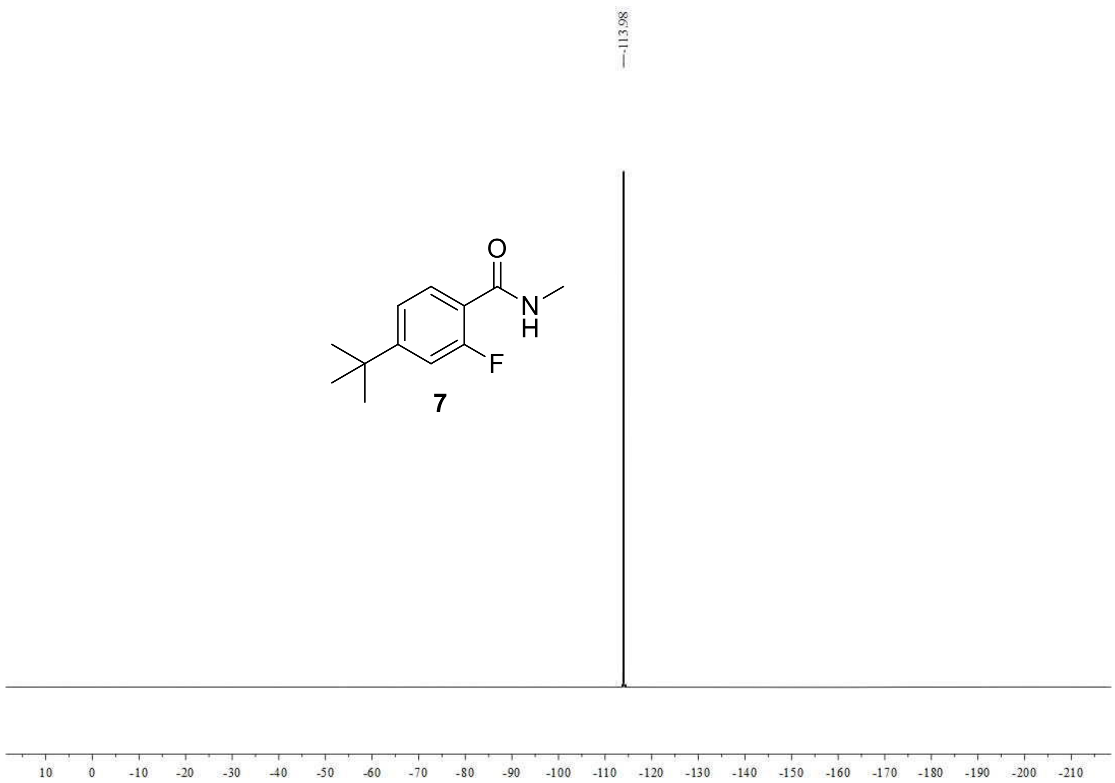

Supplementary Figure 92. ^{19}F NMR Spectrum of substrate 7

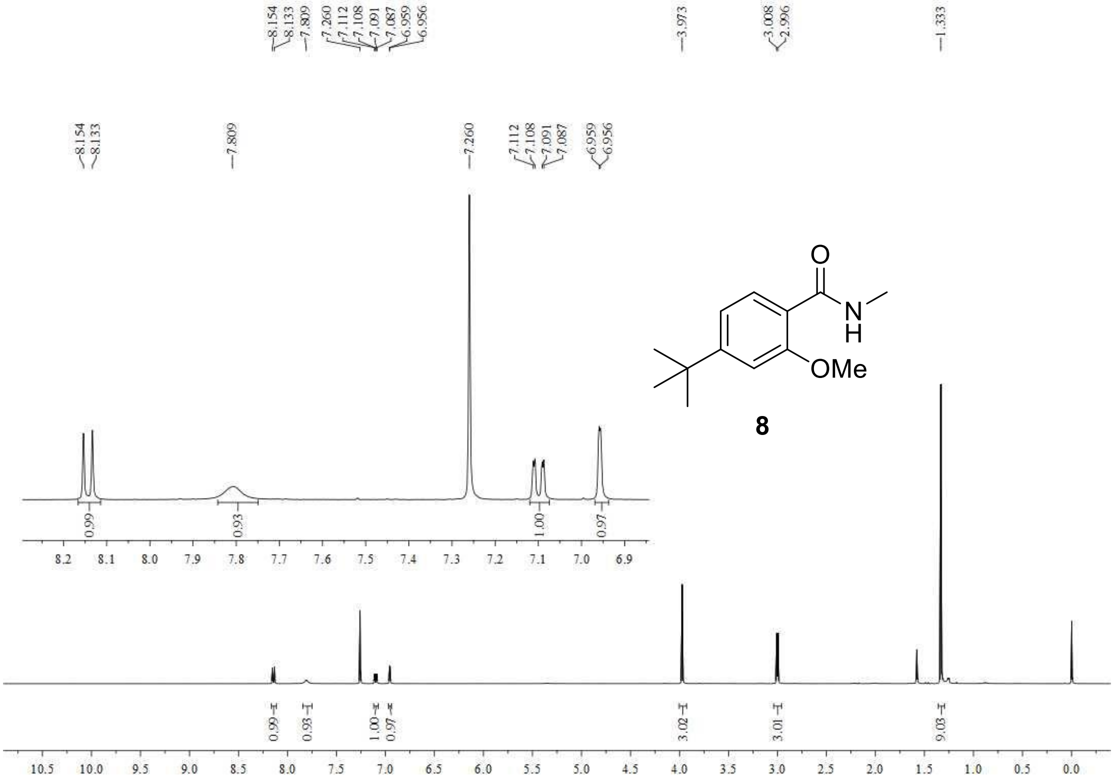

Supplementary Figure 93. ^{1}H NMR Spectrum of substrate 8

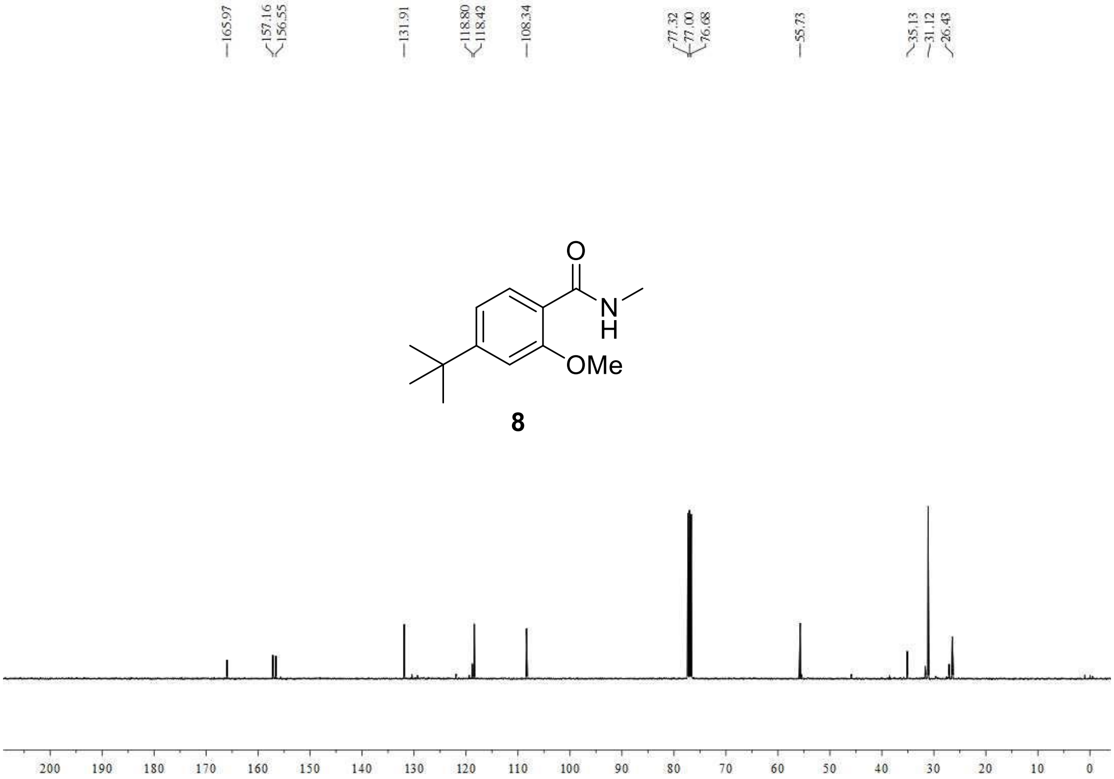

Supplementary Figure 94. ^{13}C NMR Spectrum of substrate 8

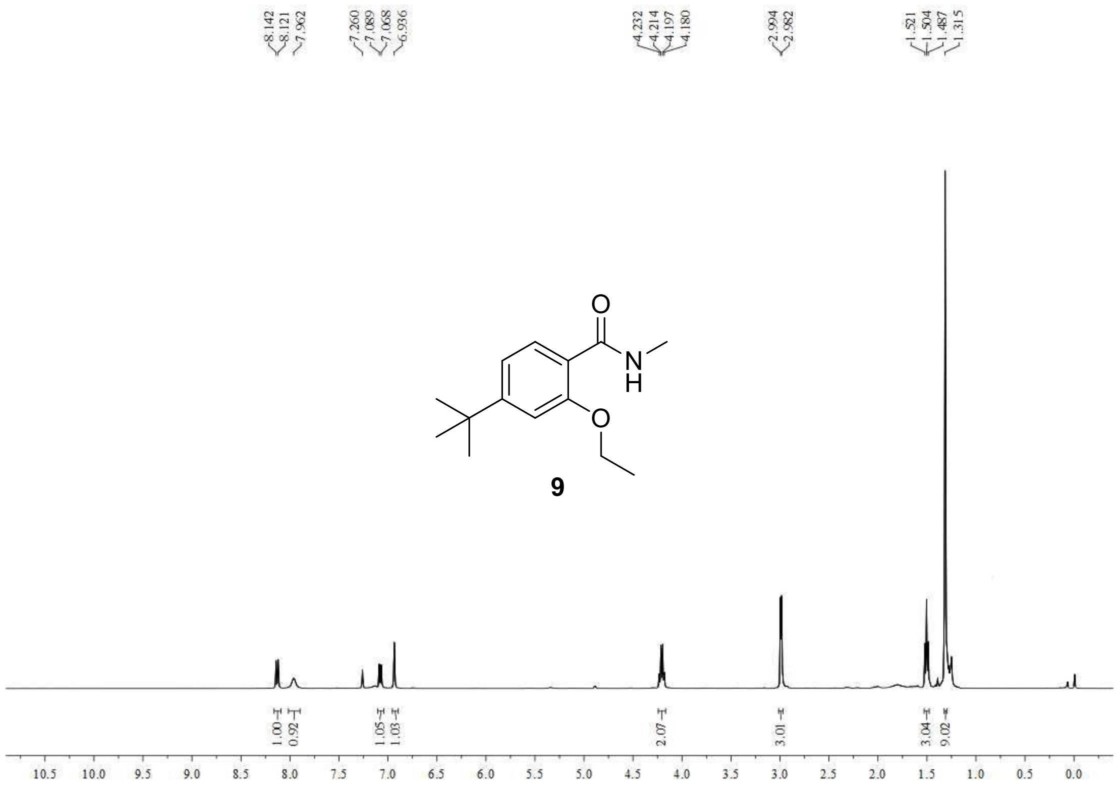

Supplementary Figure 95. ^{1}H NMR Spectrum of substrate 9

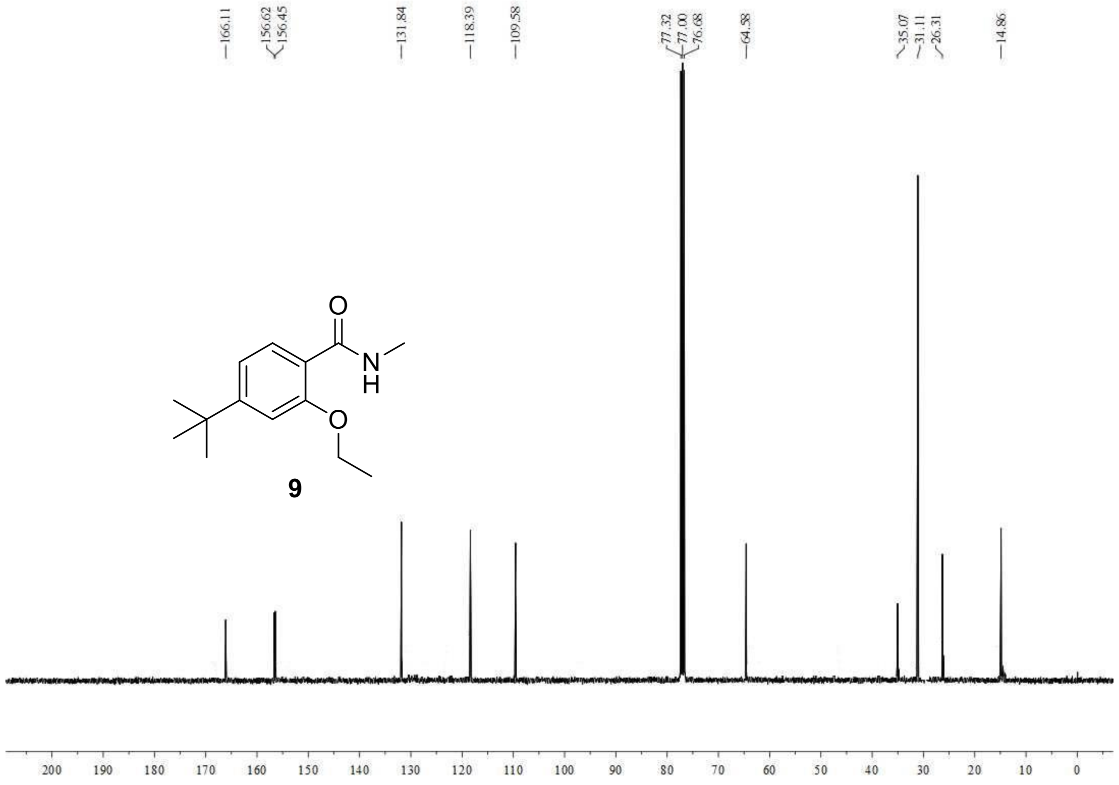

Supplementary Figure 96. ^{13}C NMR Spectrum of substrate 9

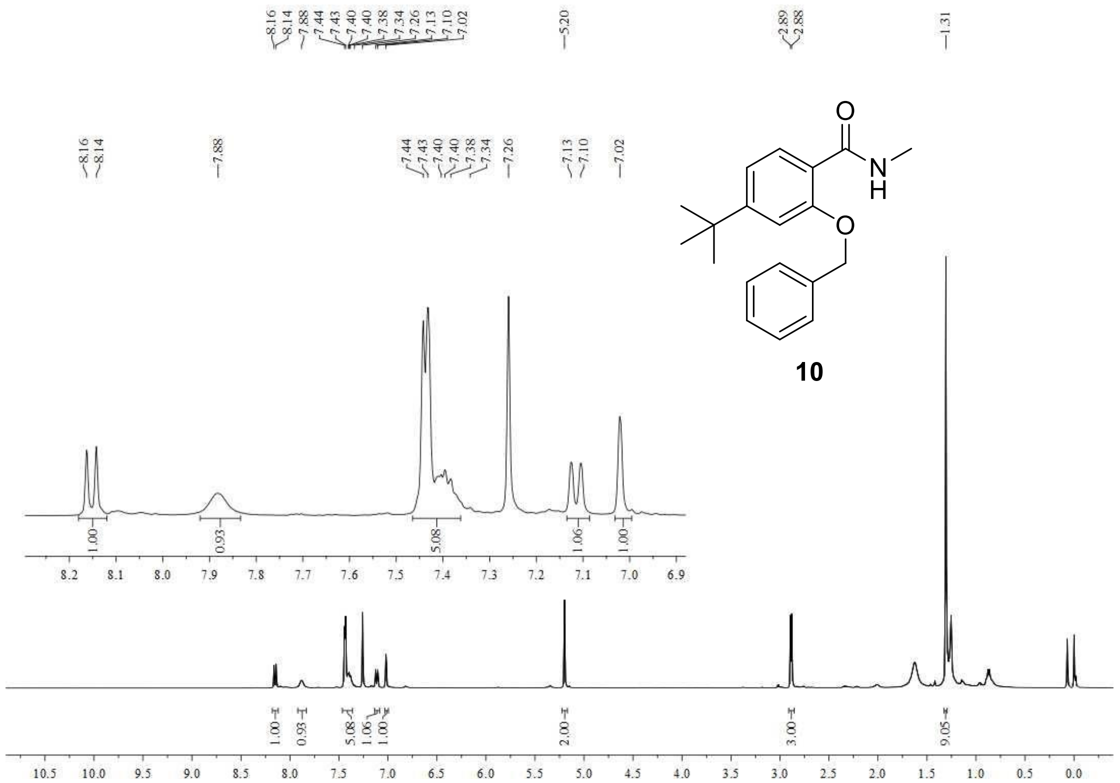

Supplementary Figure 97. ^{1}H NMR Spectrum of substrate 10

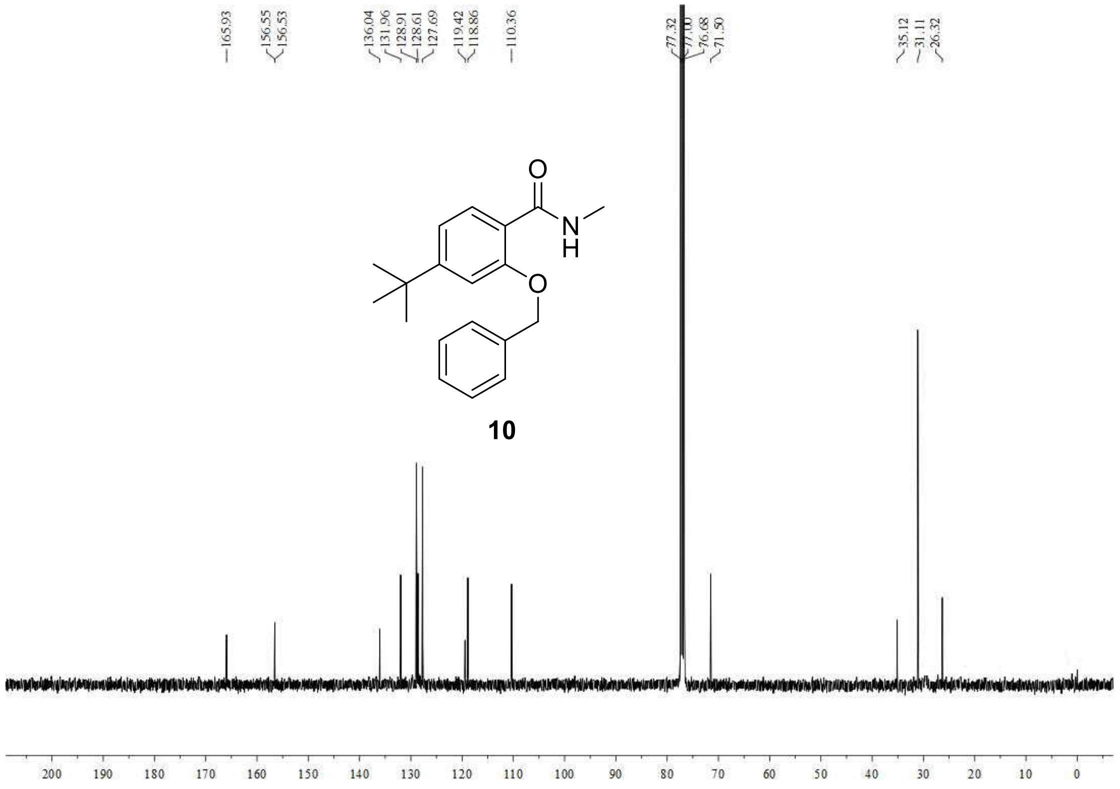

Supplementary Figure 98. ^{13}C NMR Spectrum of substrate 10

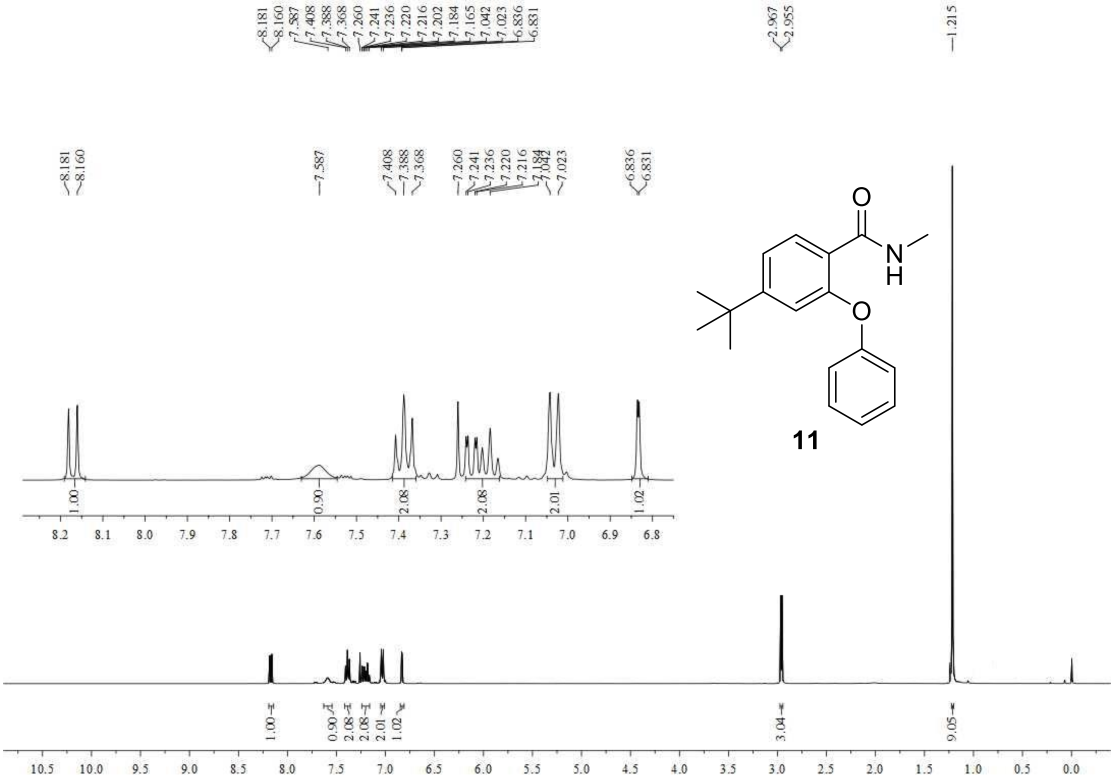

Supplementary Figure 99. ^{1}H NMR Spectrum of substrate 11

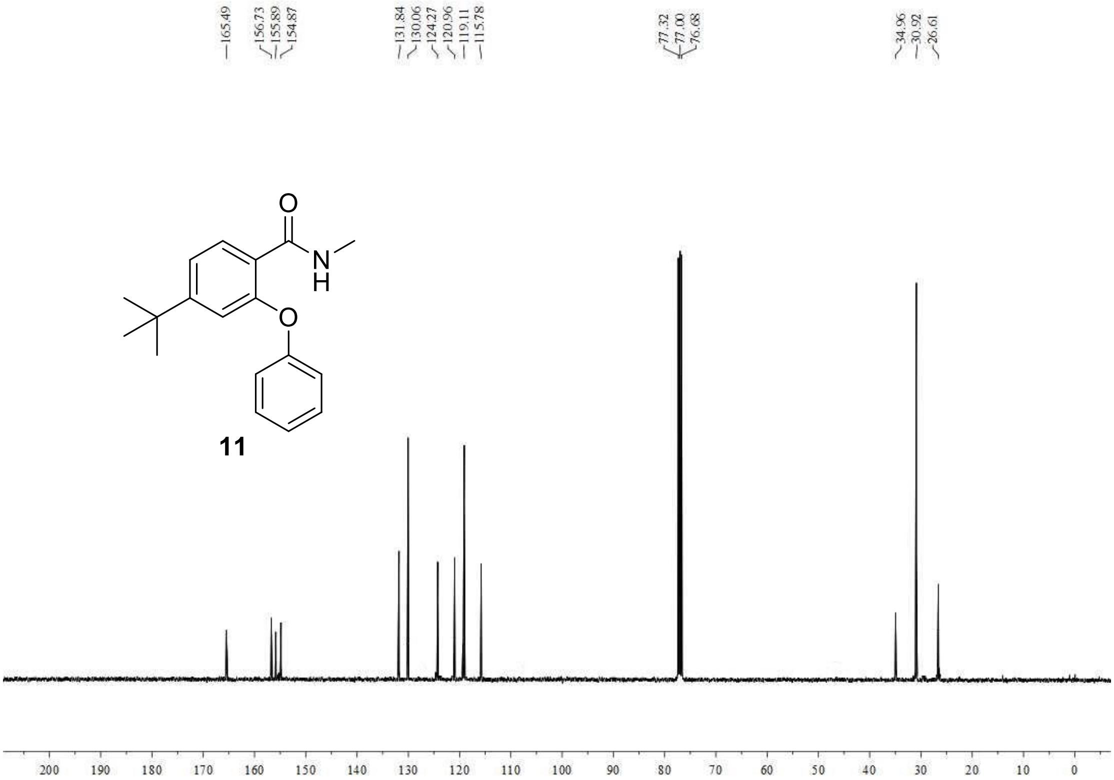

Supplementary Figure 100. ^{13}C NMR Spectrum of substrate 11

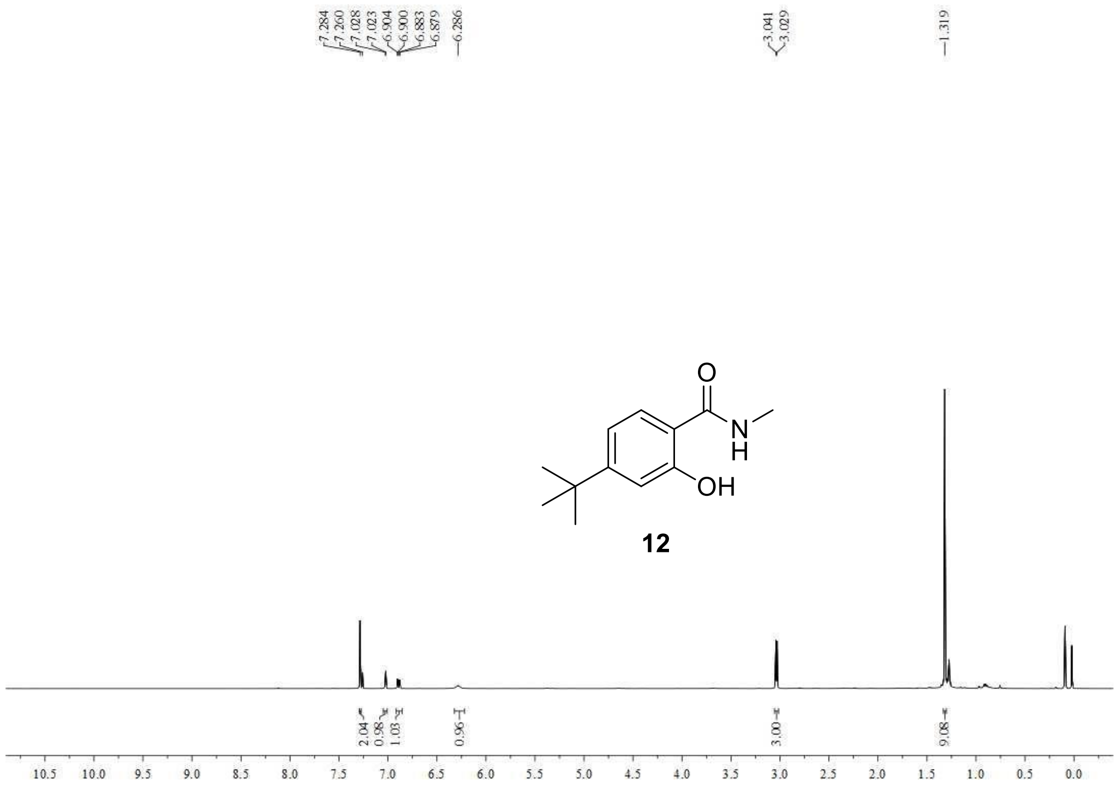

Supplementary Figure 101. ^{1}H NMR Spectrum of substrate 12

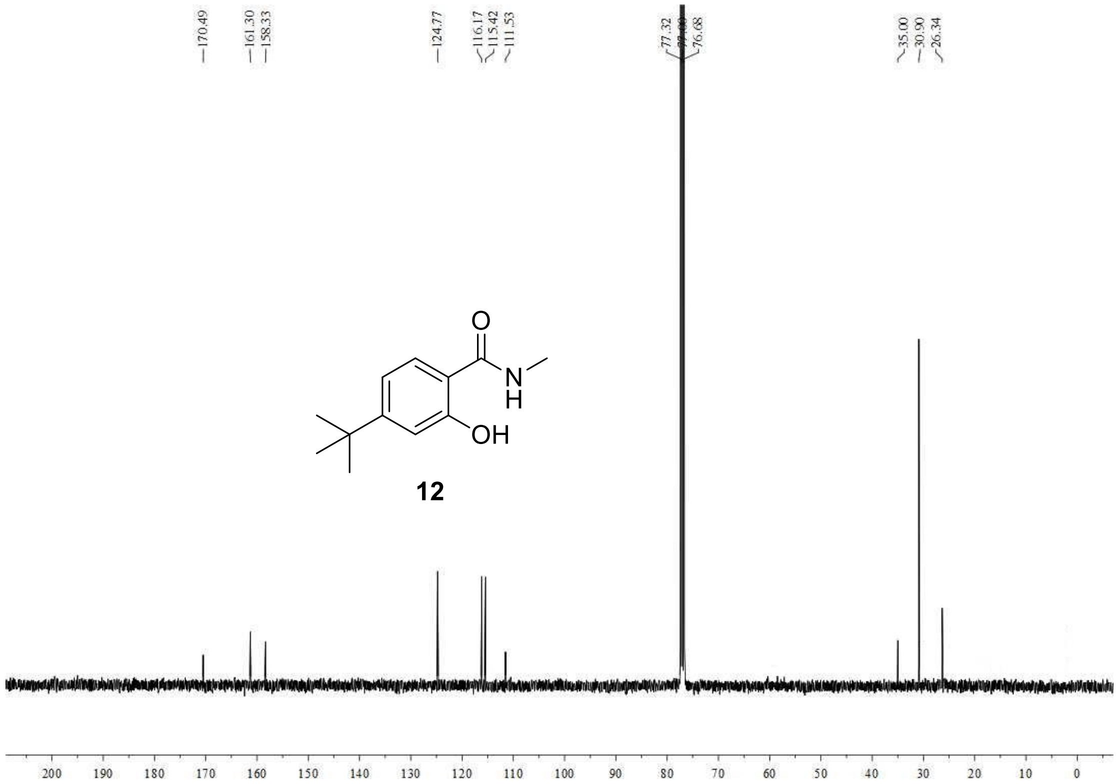

Supplementary Figure 102. ^{13}C NMR Spectrum of substrate 12

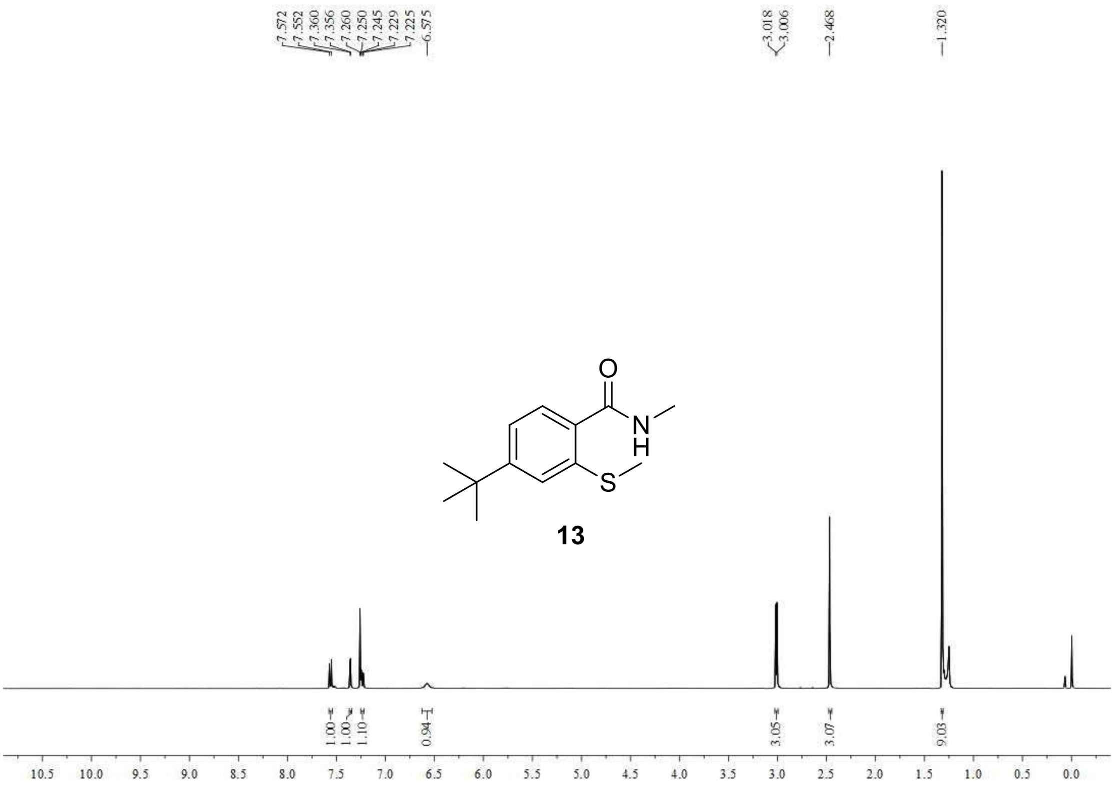

Supplementary Figure 103. ^{1}H NMR Spectrum of substrate 13

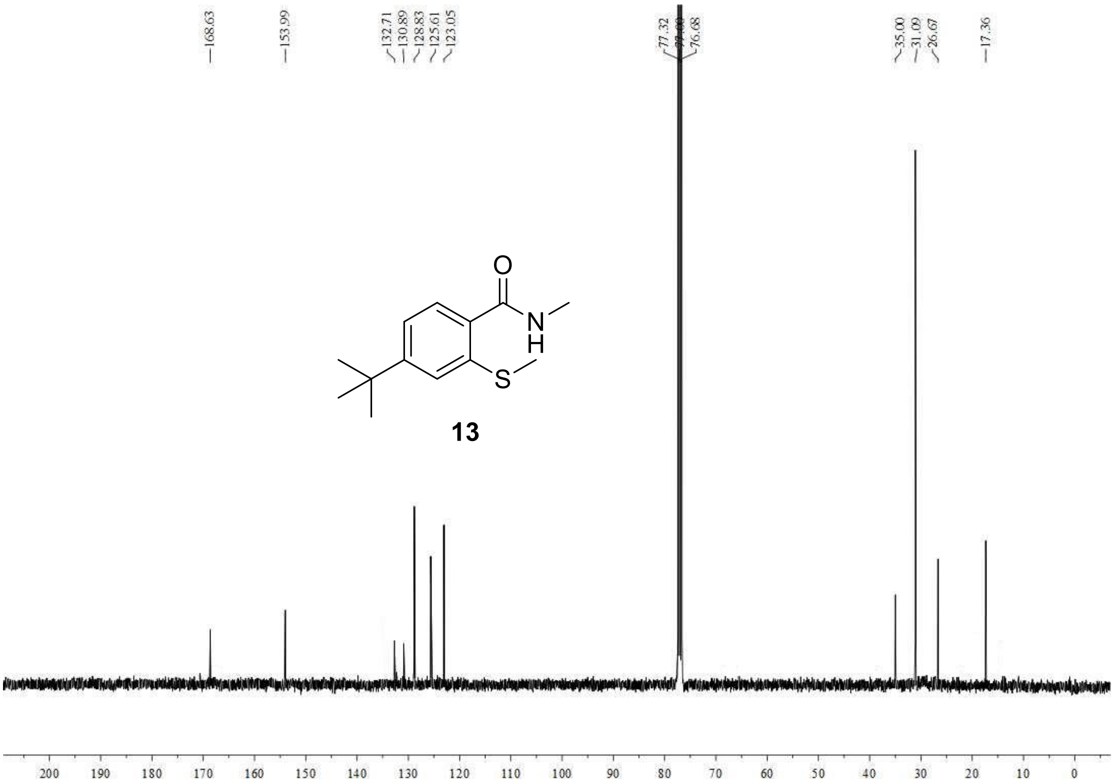

Supplementary Figure 104. ^{13}C NMR Spectrum of substrate 13

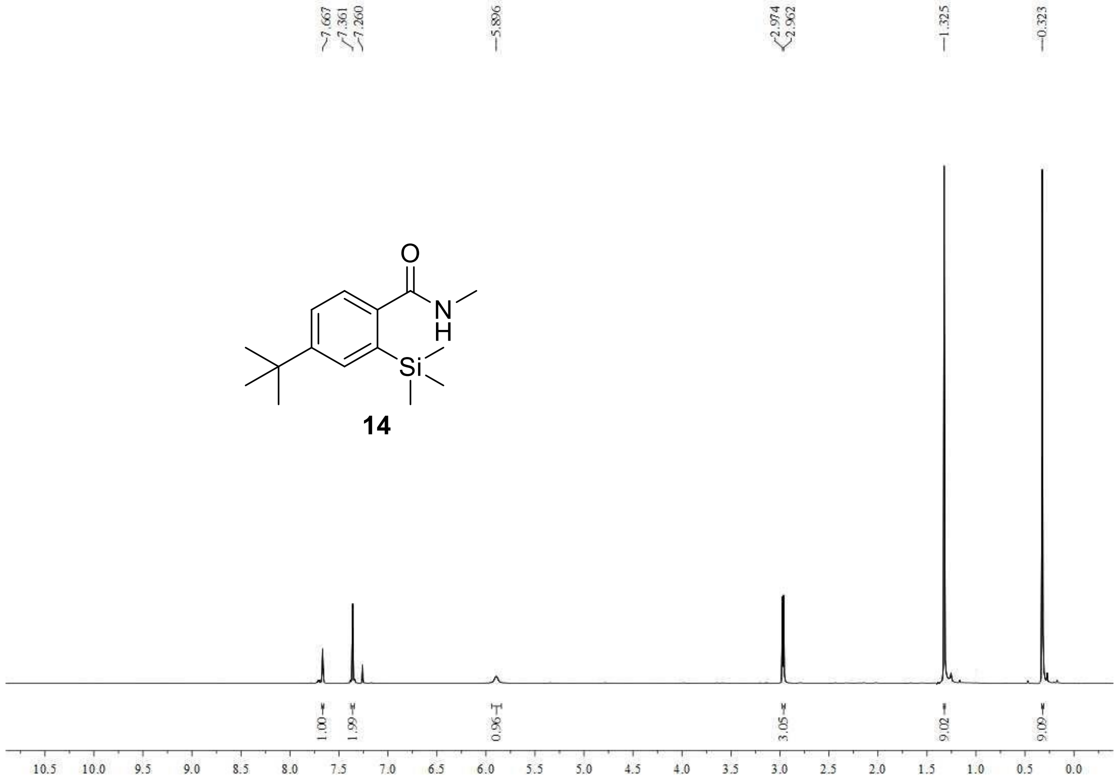

Supplementary Figure 105. ^{1}H NMR Spectrum of substrate 14

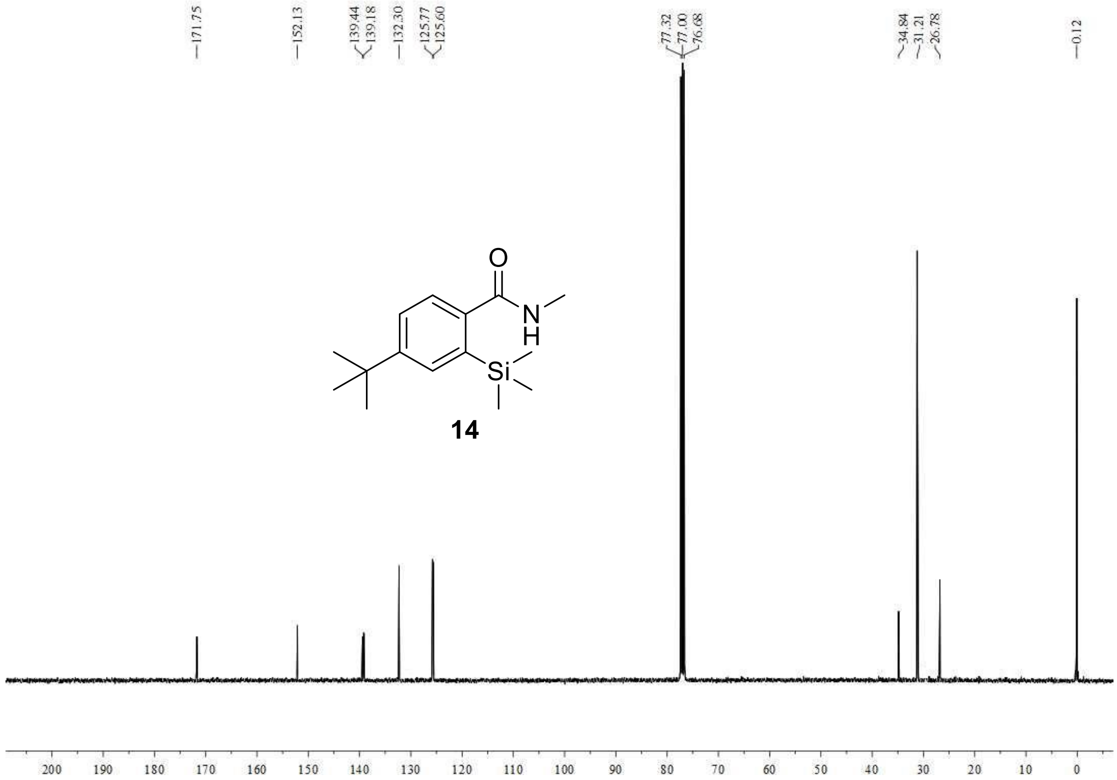

Supplementary Figure 106. ^{13}C NMR Spectrum of substrate 14

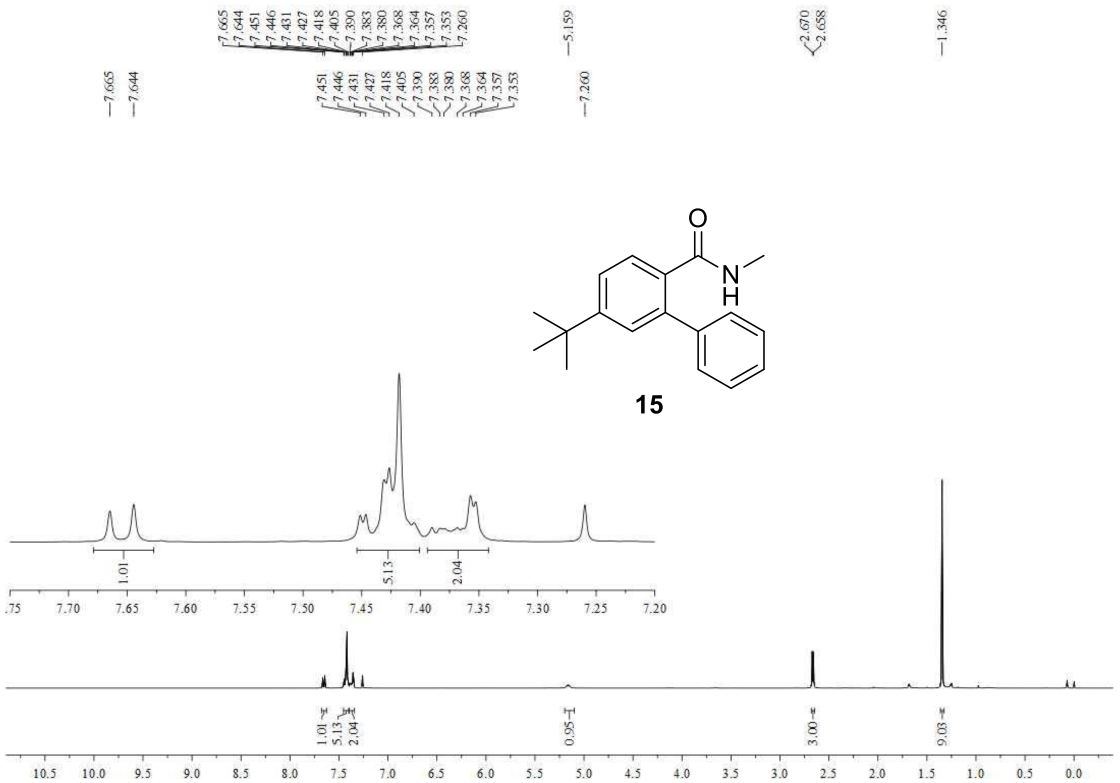

Supplementary Figure 107. ^{1}H NMR Spectrum of substrate 15

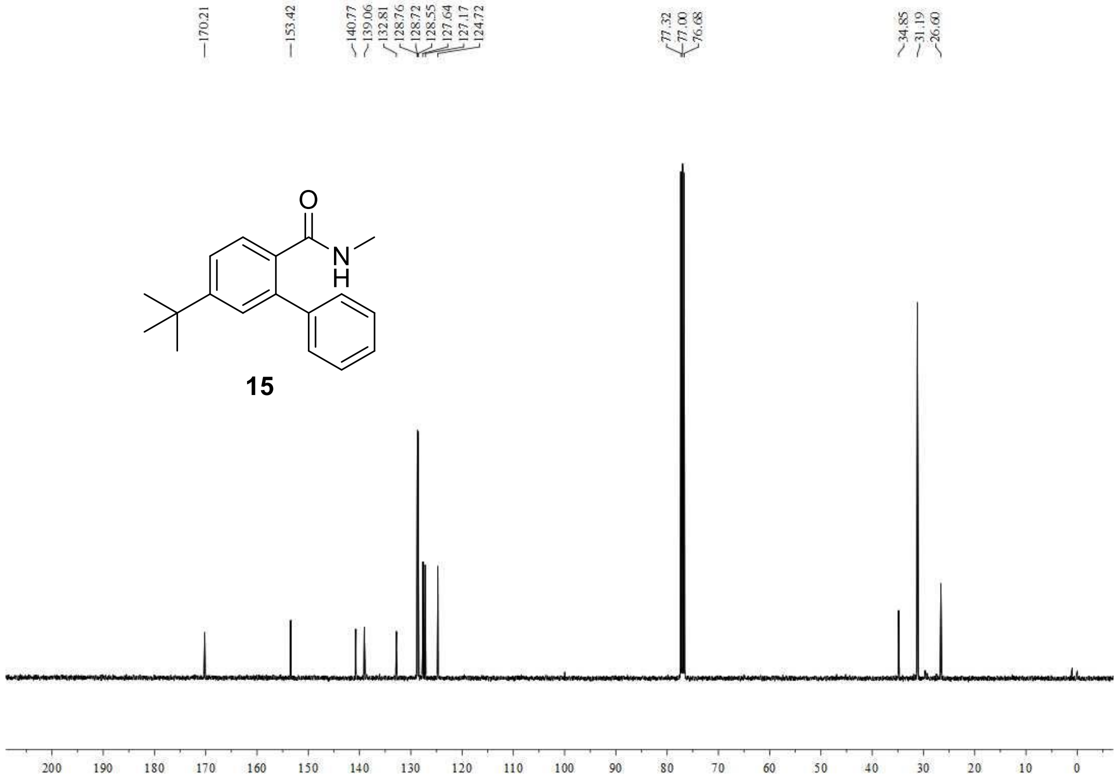

Supplementary Figure 108. ^{13}C NMR Spectrum of substrate 15

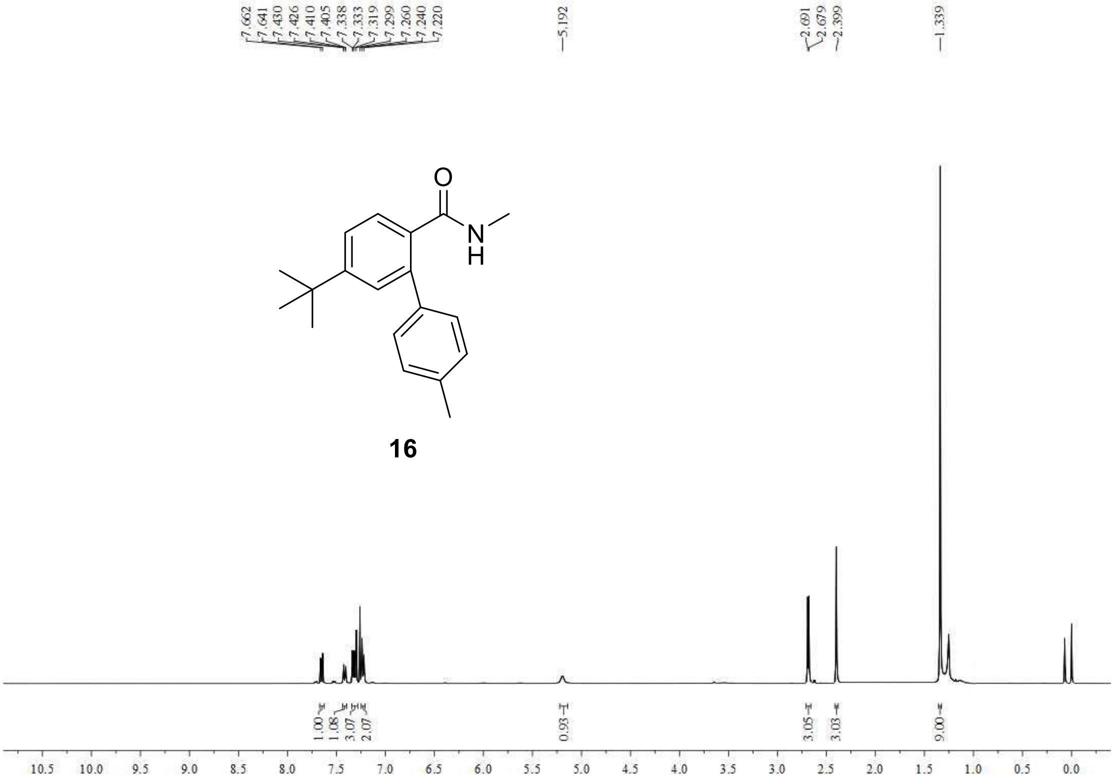

Supplementary Figure 109. ^{1}H NMR Spectrum of substrate 16

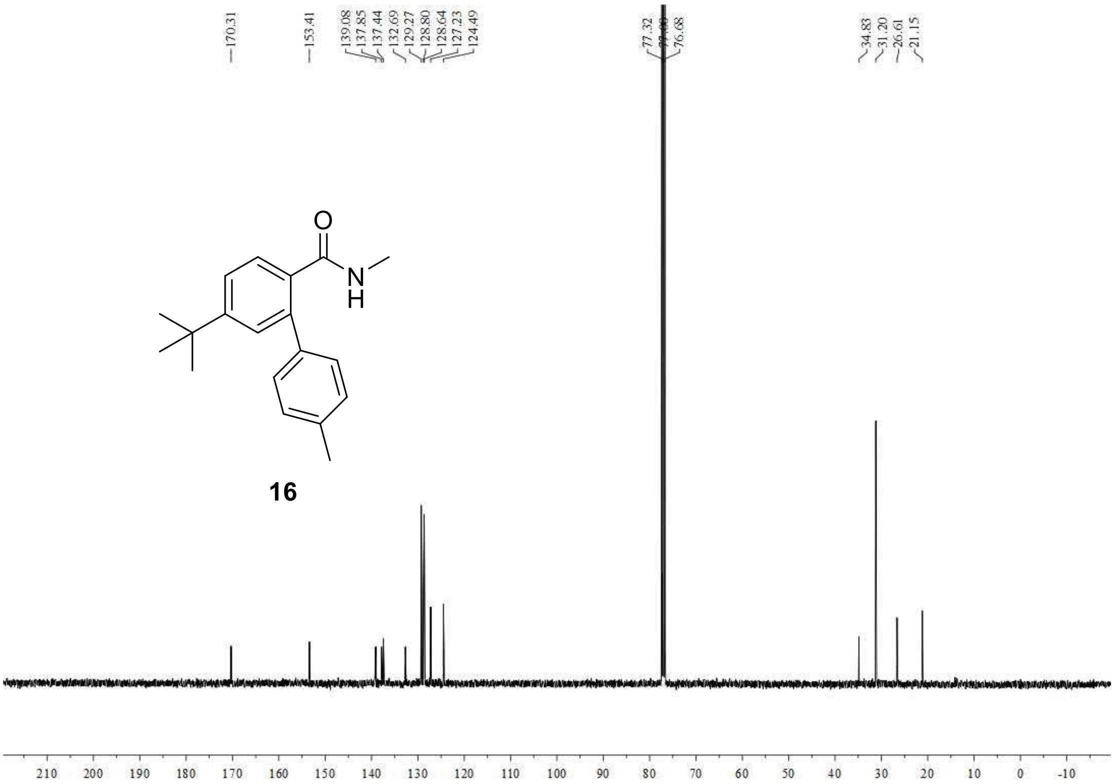

Supplementary Figure 110. ^{13}C NMR Spectrum of substrate 16

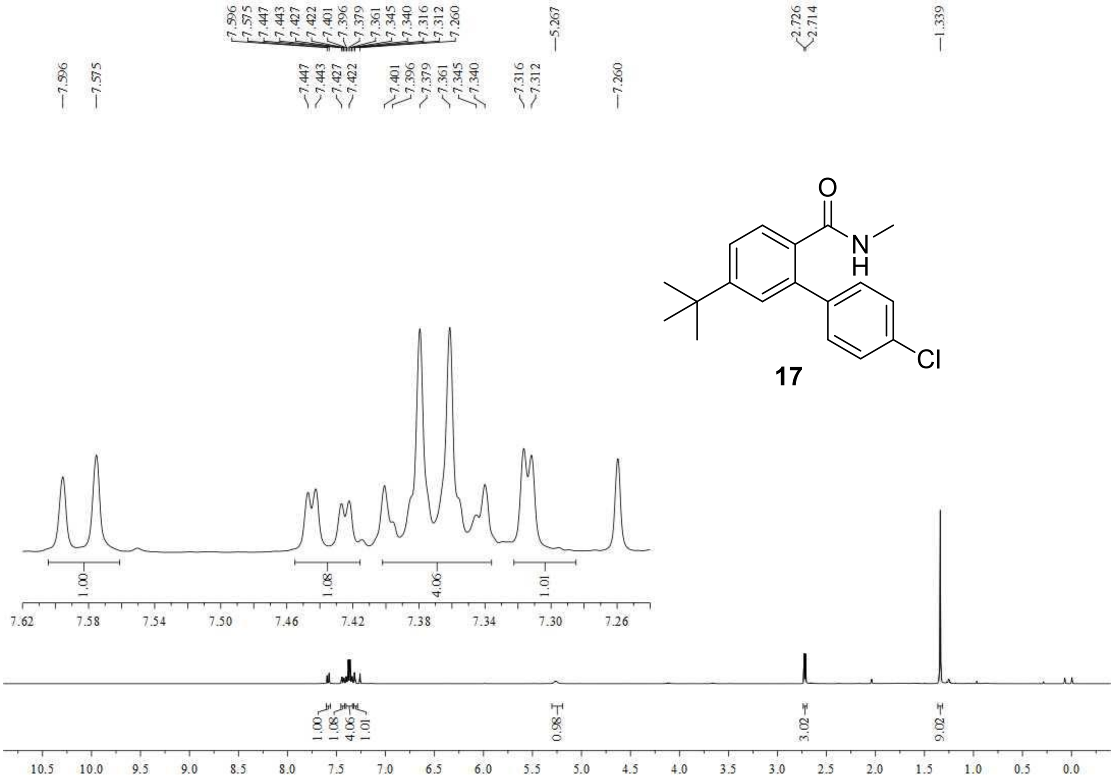

Supplementary Figure 111. ^{1}H NMR Spectrum of substrate 17

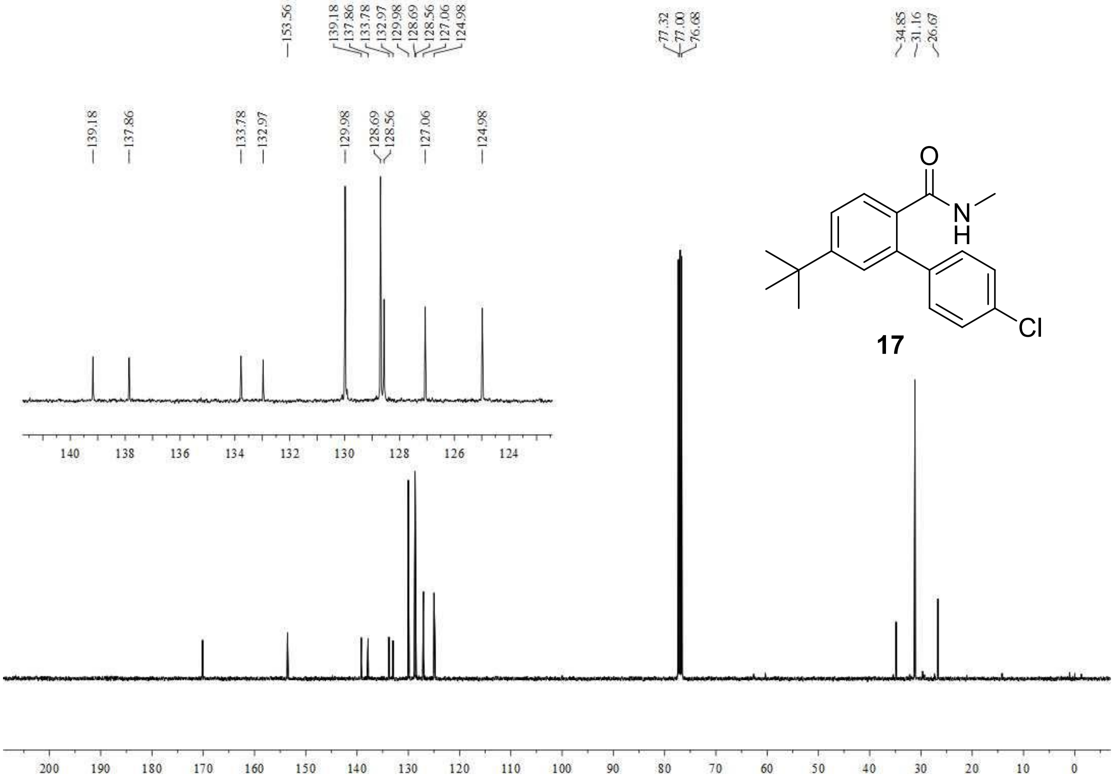

Supplementary Figure 112. ^{13}C NMR Spectrum of substrate 17

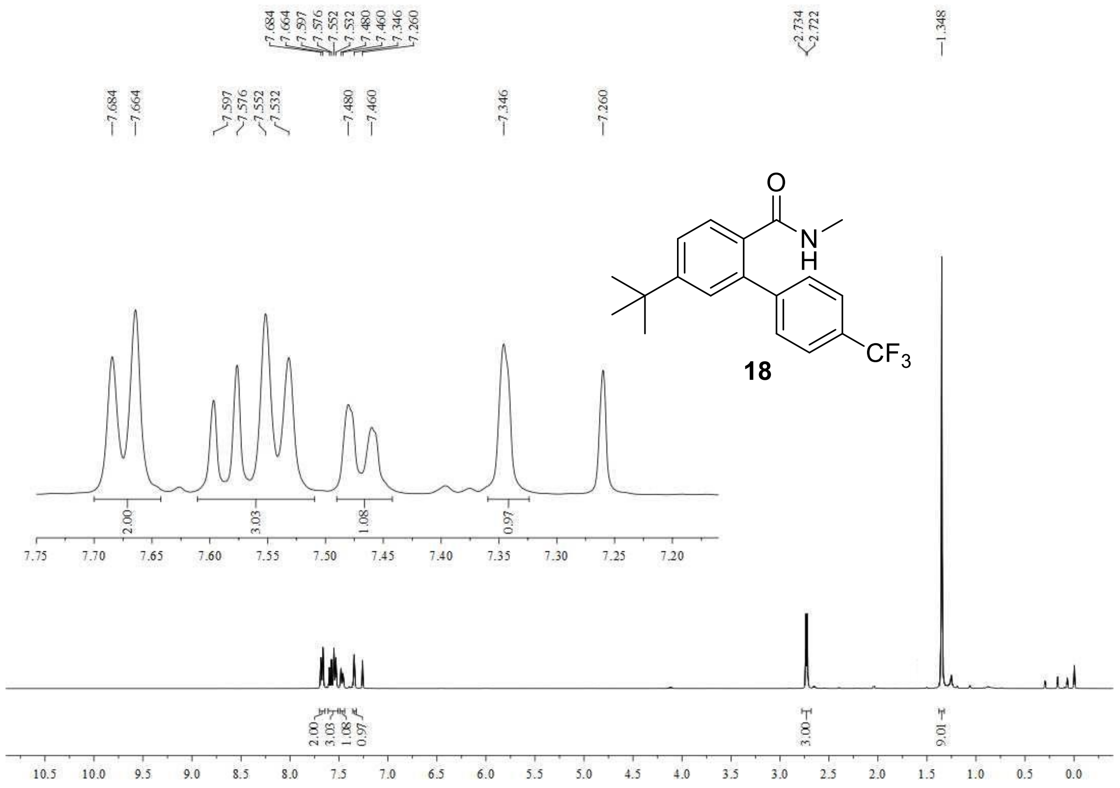

Supplementary Figure 113. ^{1}H NMR Spectrum of substrate 18

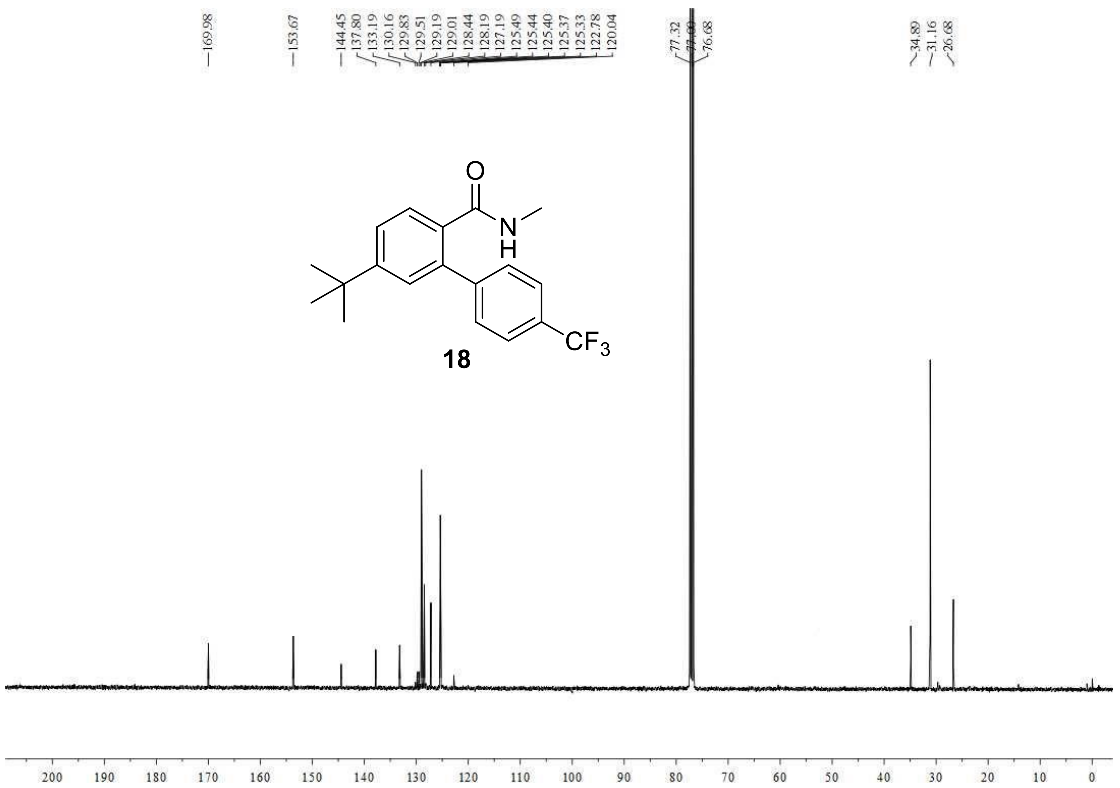

Supplementary Figure 114. ^{13}C NMR Spectrum of substrate 18

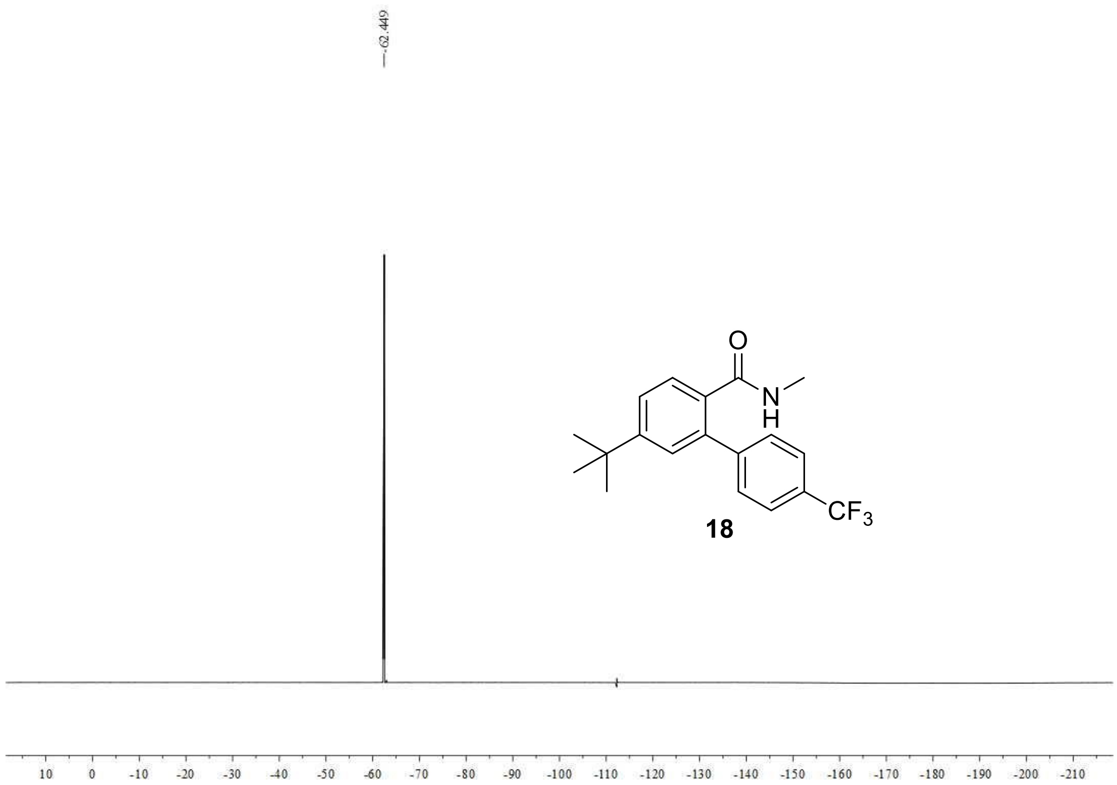

Supplementary Figure 115. ^{19}F NMR Spectrum of substrate 18

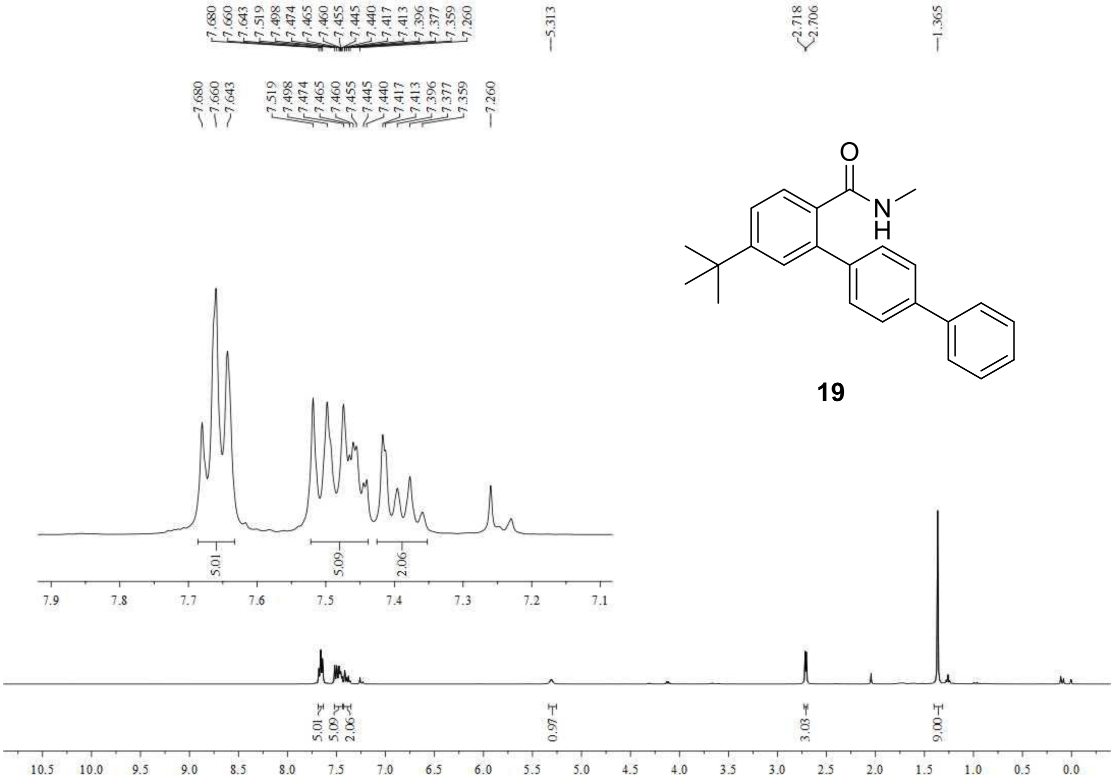

Supplementary Figure 116. ^{1}H NMR Spectrum of substrate 19

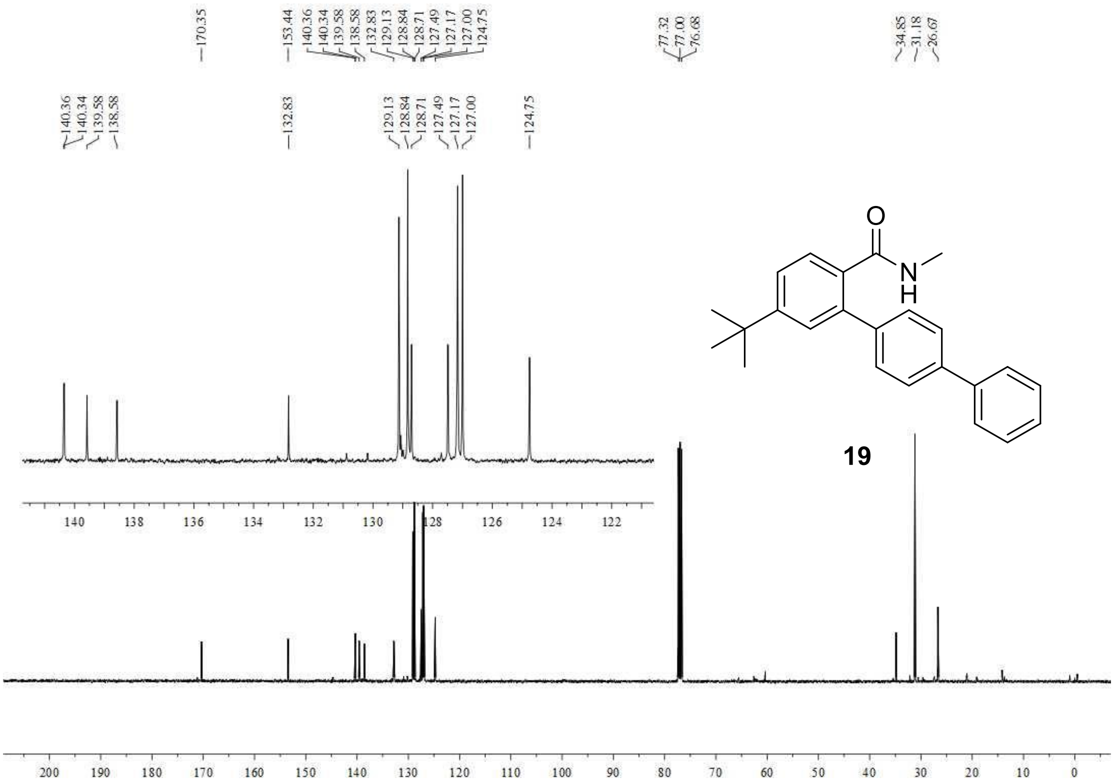

Supplementary Figure 117. ^{13}C NMR Spectrum of substrate 19

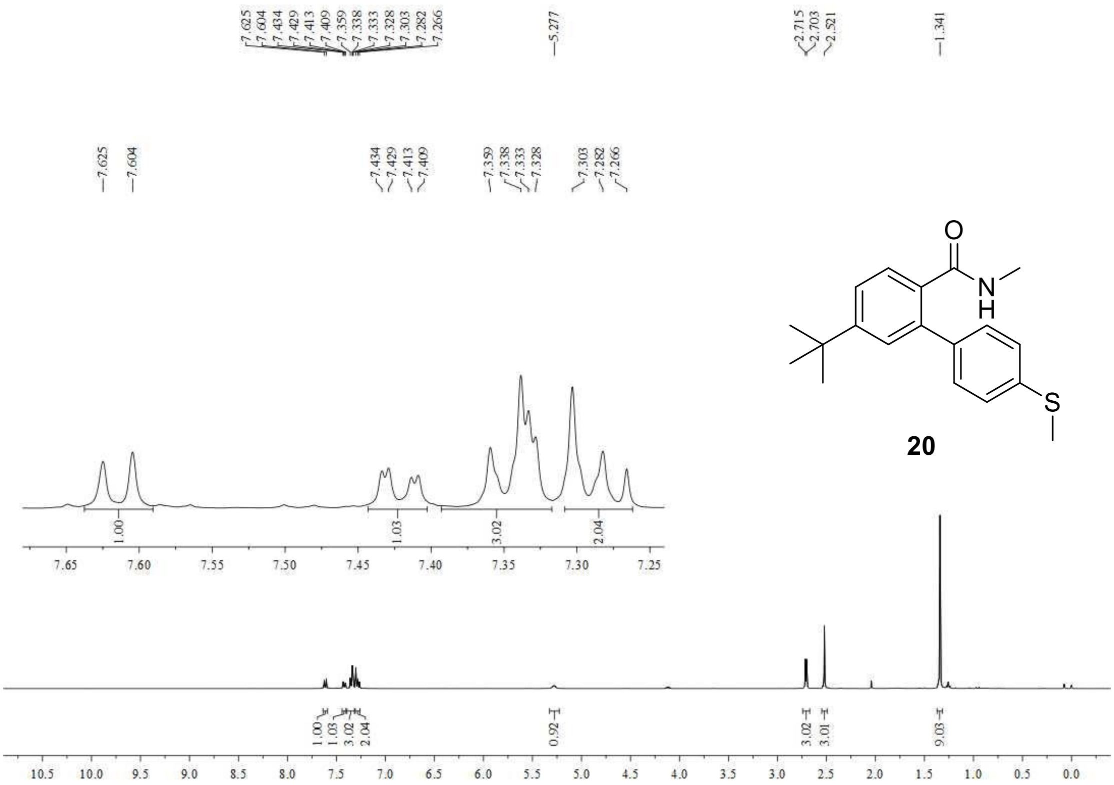

Supplementary Figure 118. ^{1}H NMR Spectrum of substrate 20

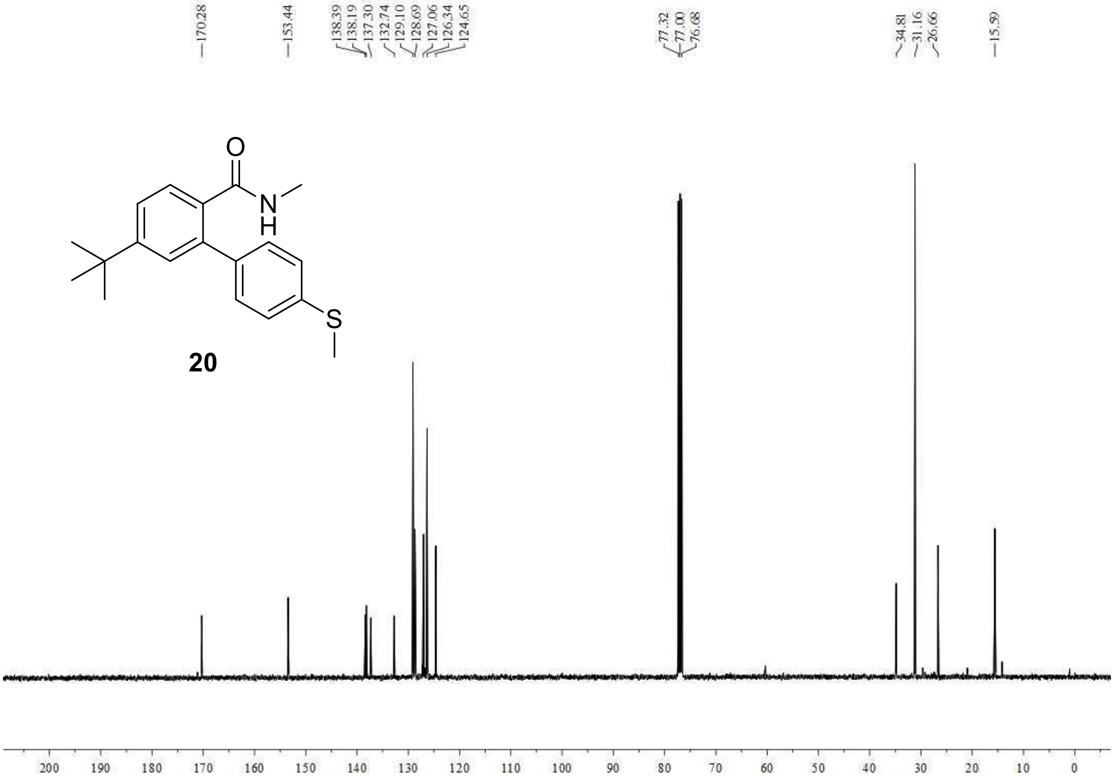

Supplementary Figure 119. ^{13}C NMR Spectrum of substrate 20

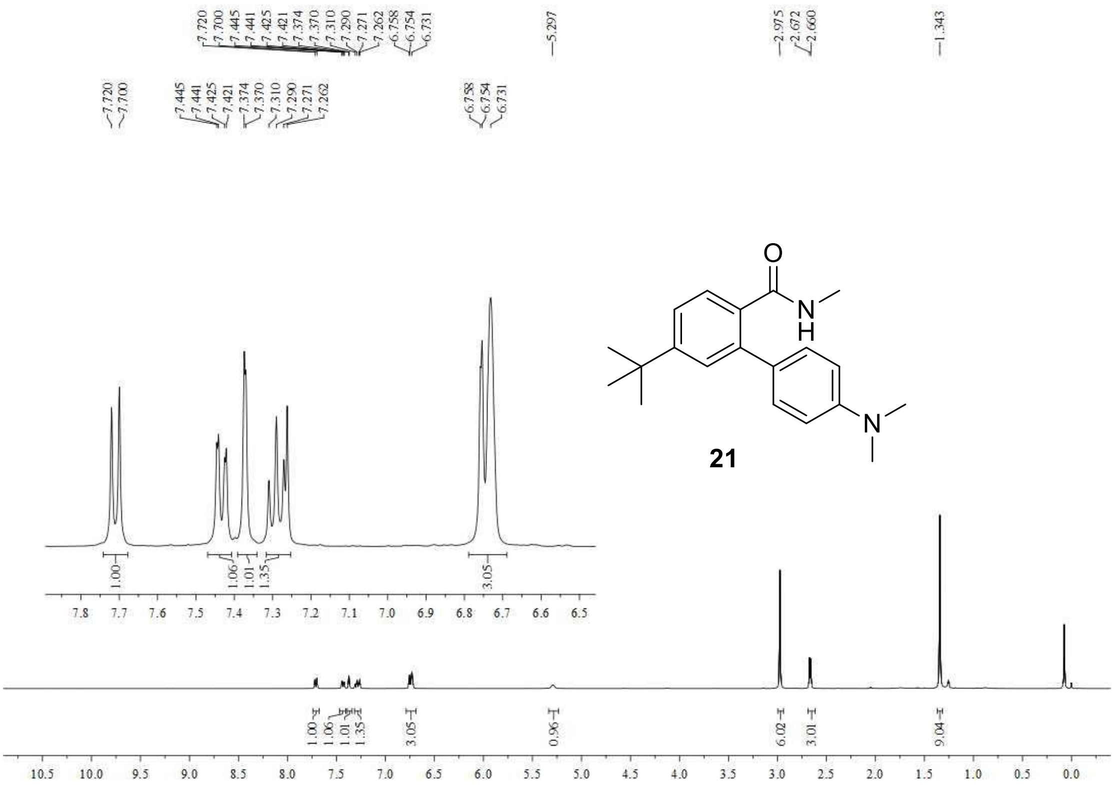

Supplementary Figure 120. ^{1}H NMR Spectrum of substrate 21

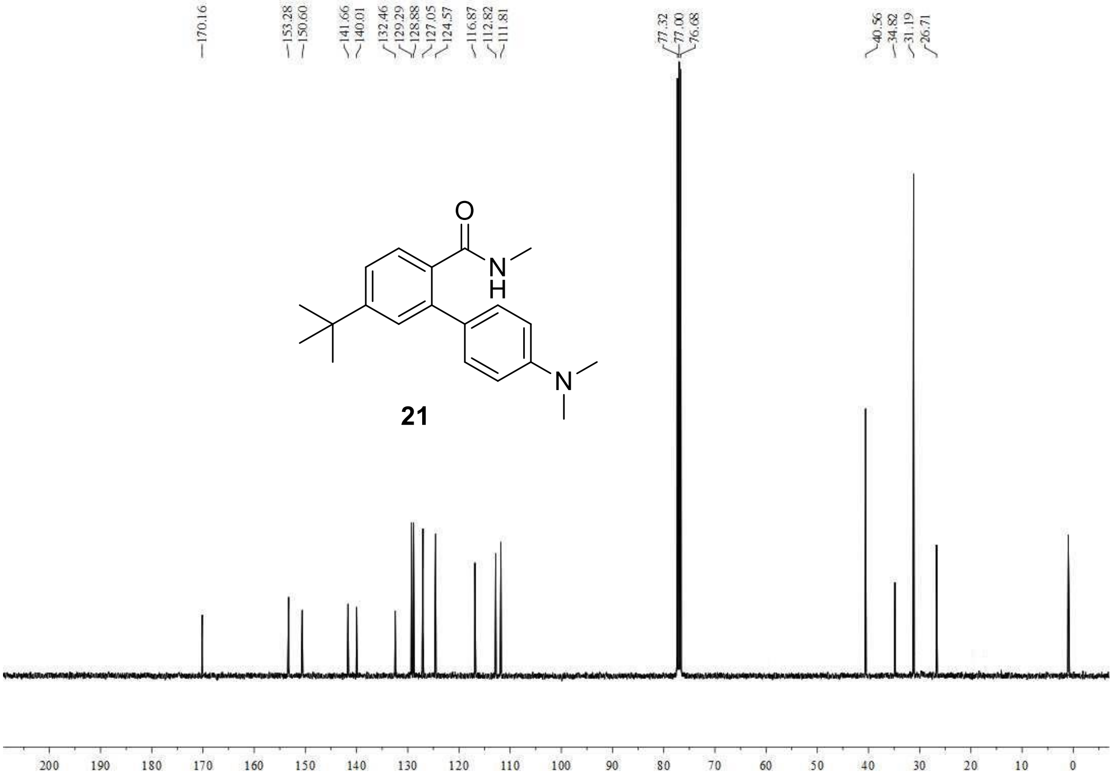

Supplementary Figure 121. ^{13}C NMR Spectrum of substrate 21

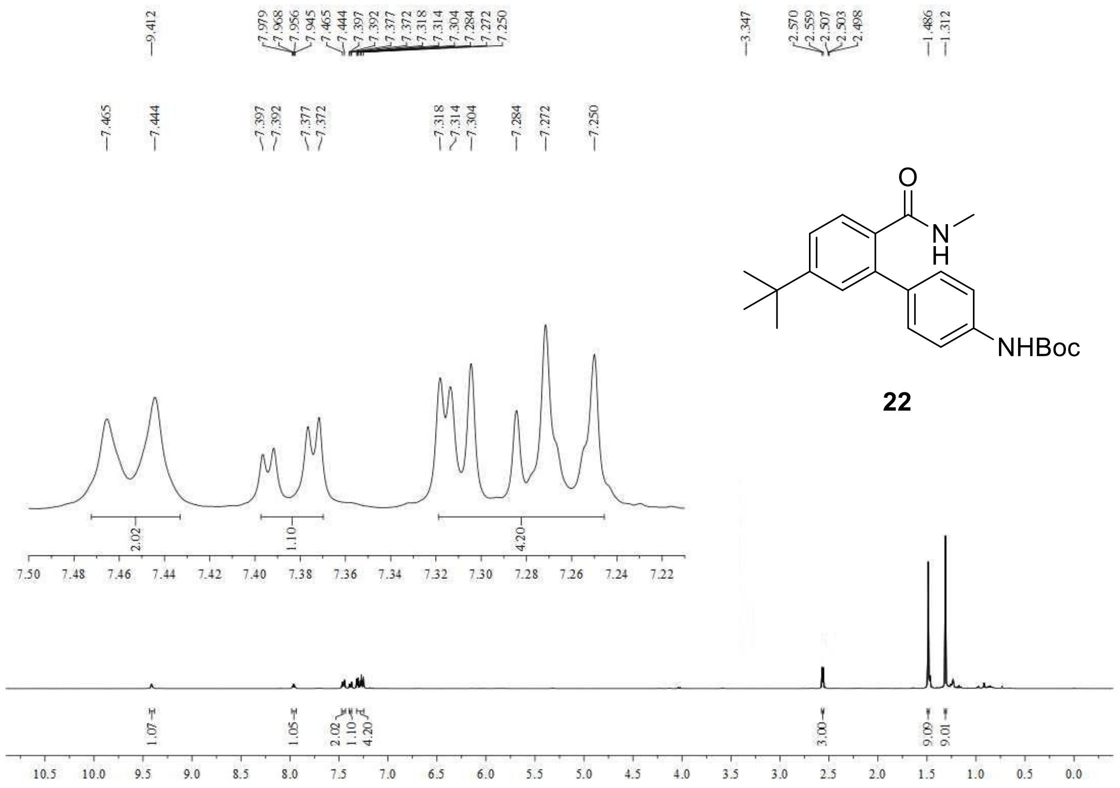

Supplementary Figure 122. ^{1}H NMR Spectrum of substrate 22

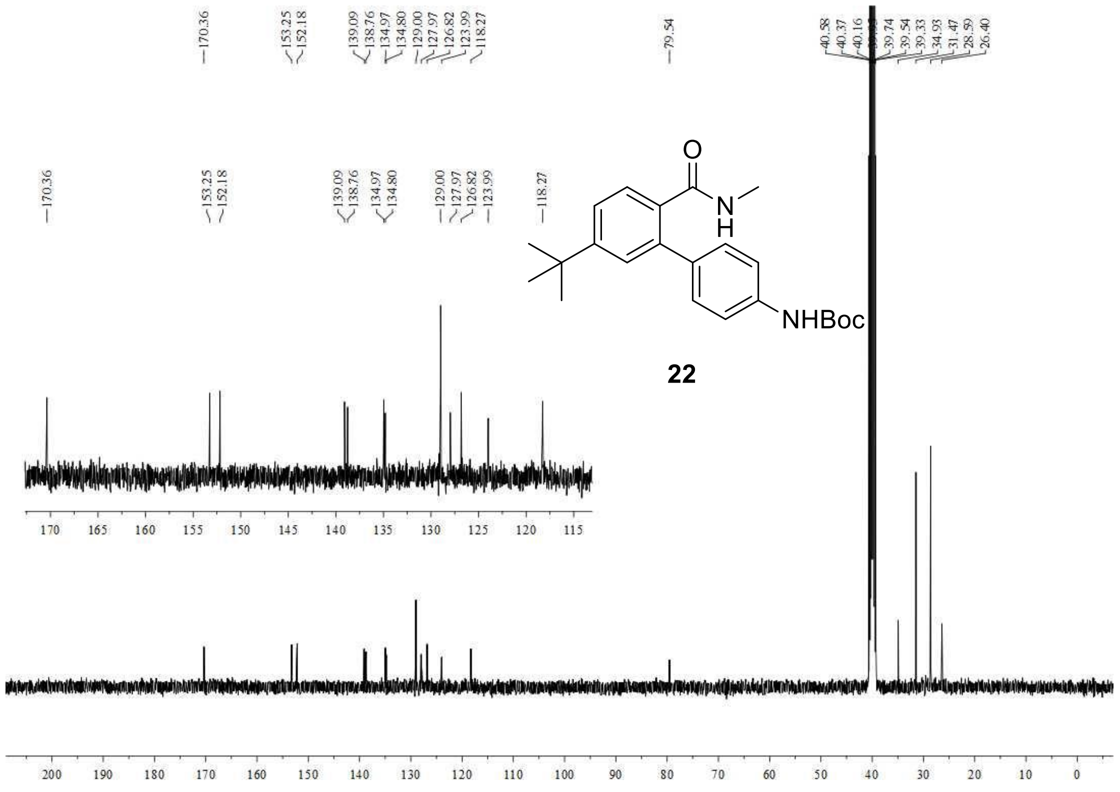

Supplementary Figure 123. ^{13}C NMR Spectrum of substrate 22

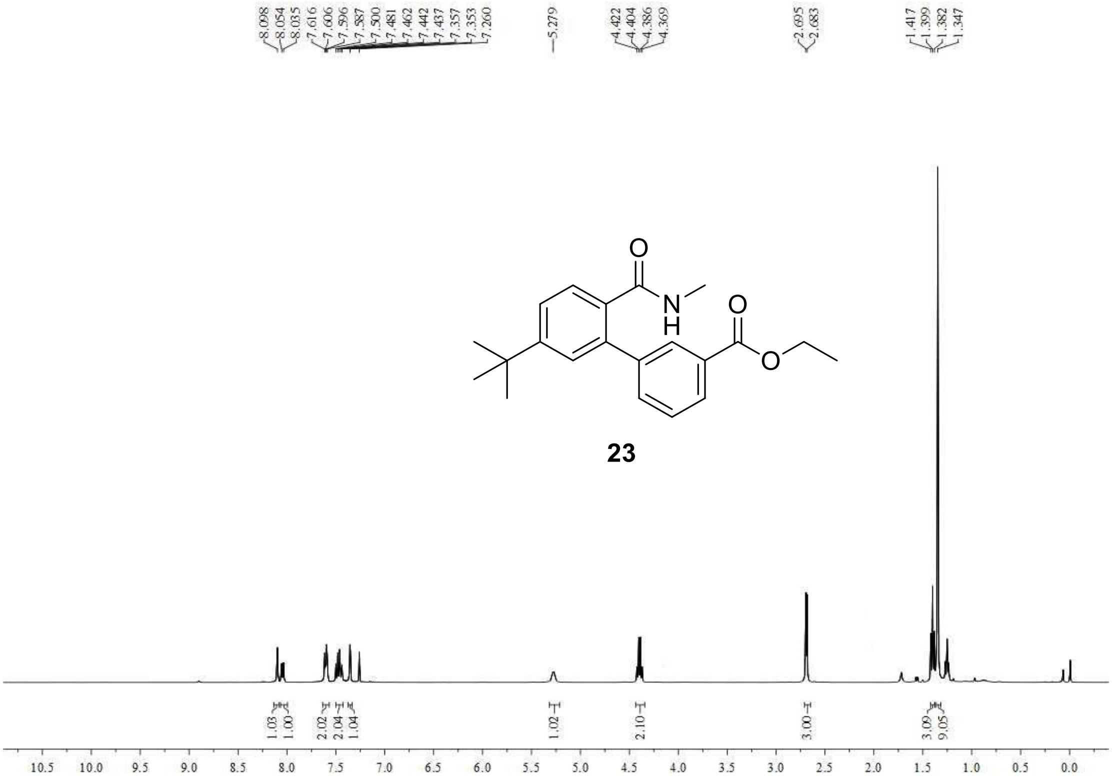

Supplementary Figure 124. ^{1}H NMR Spectrum of substrate 23

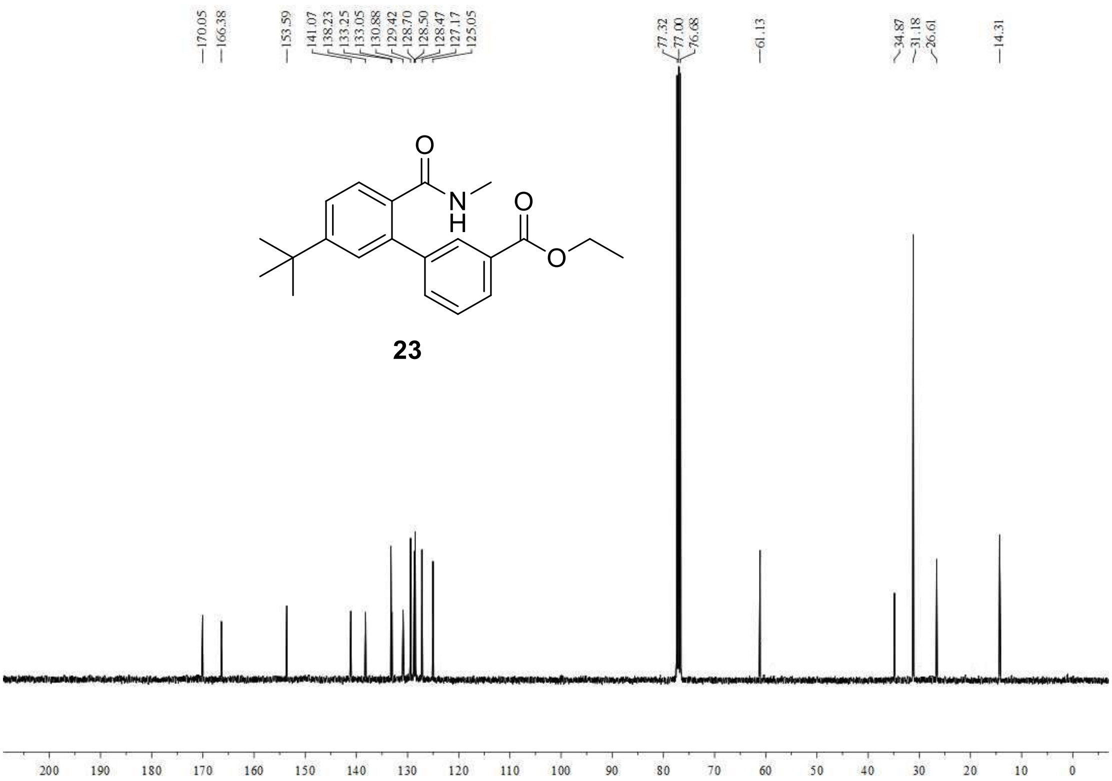

Supplementary Figure 125. ^{13}C NMR Spectrum of substrate 23

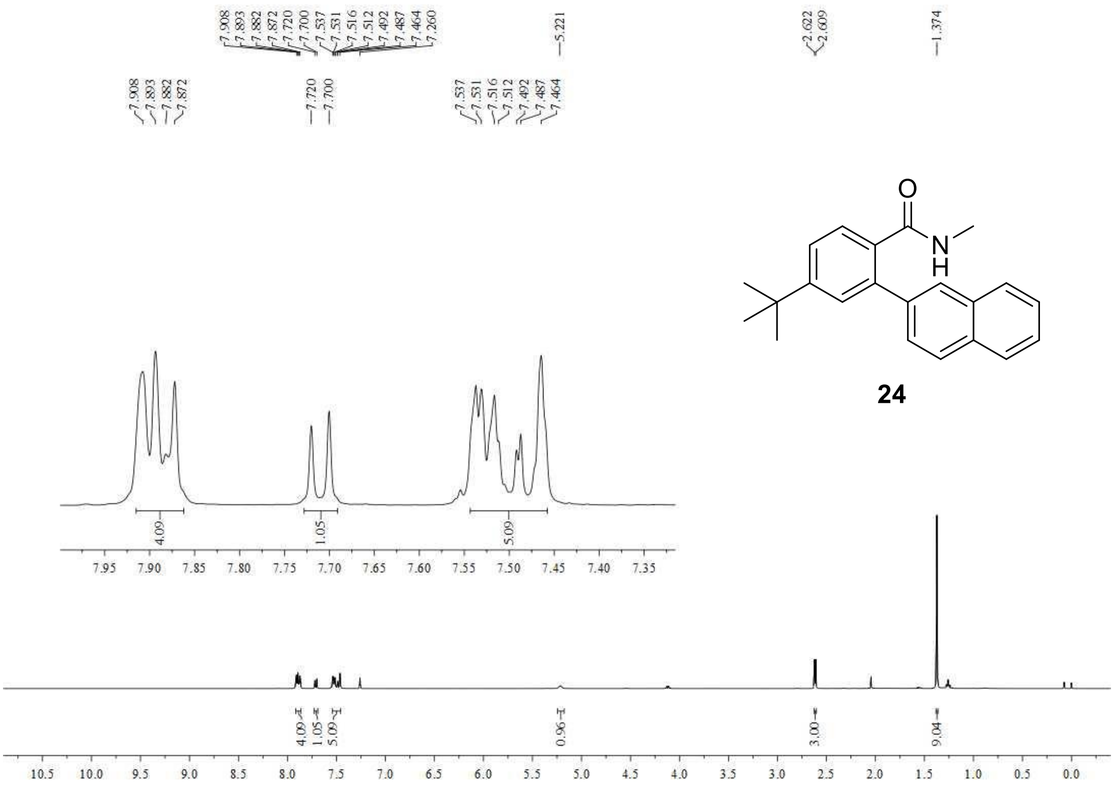

Supplementary Figure 126. ^{1}H NMR Spectrum of substrate 24

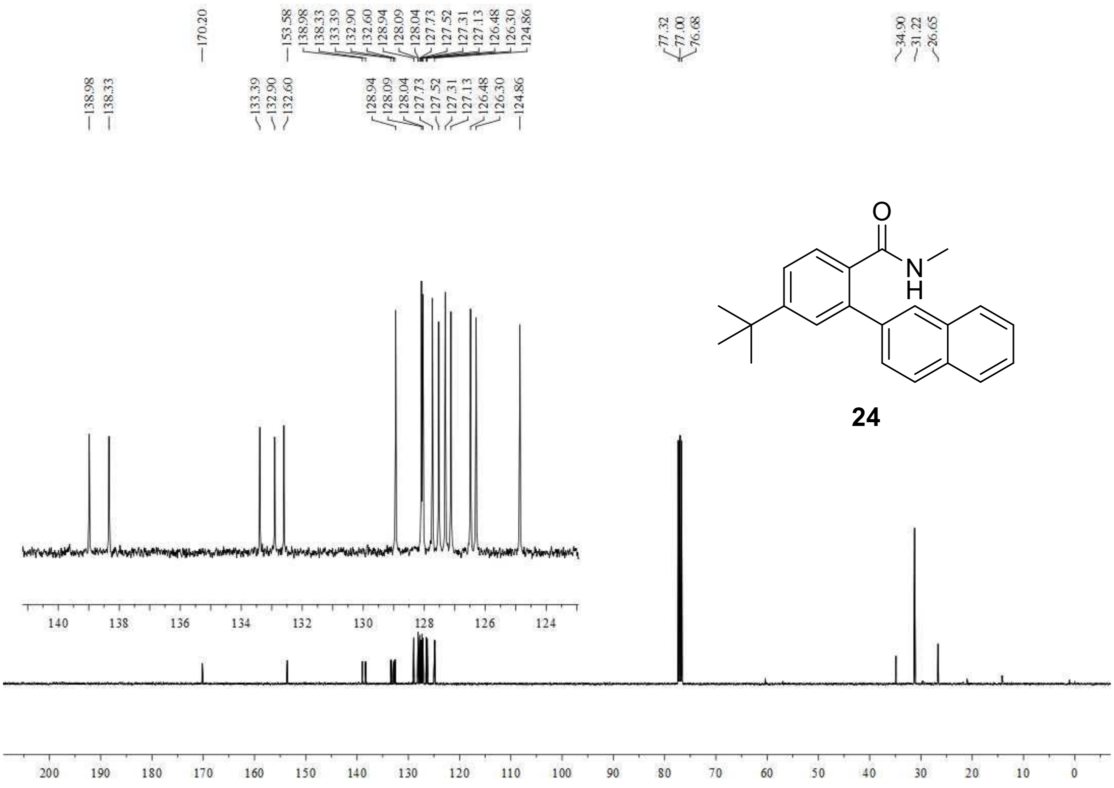

Supplementary Figure 127. ^{13}C NMR Spectrum of substrate 24

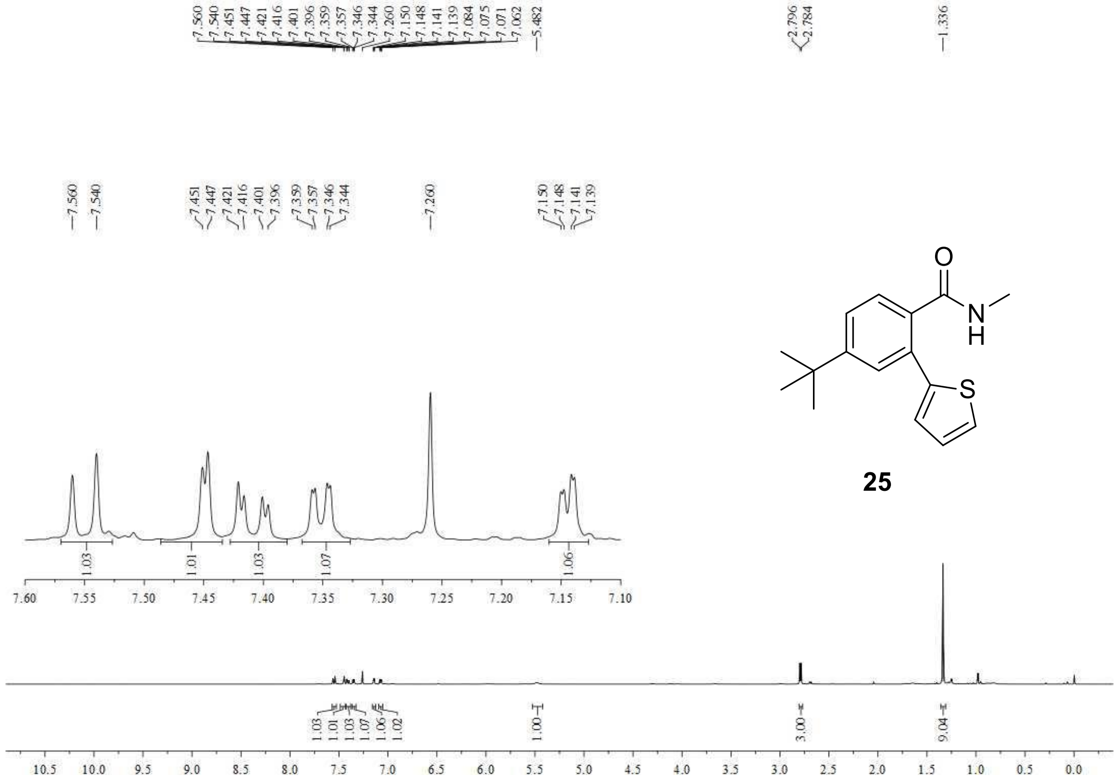

Supplementary Figure 128. ^{1}H NMR Spectrum of substrate 25

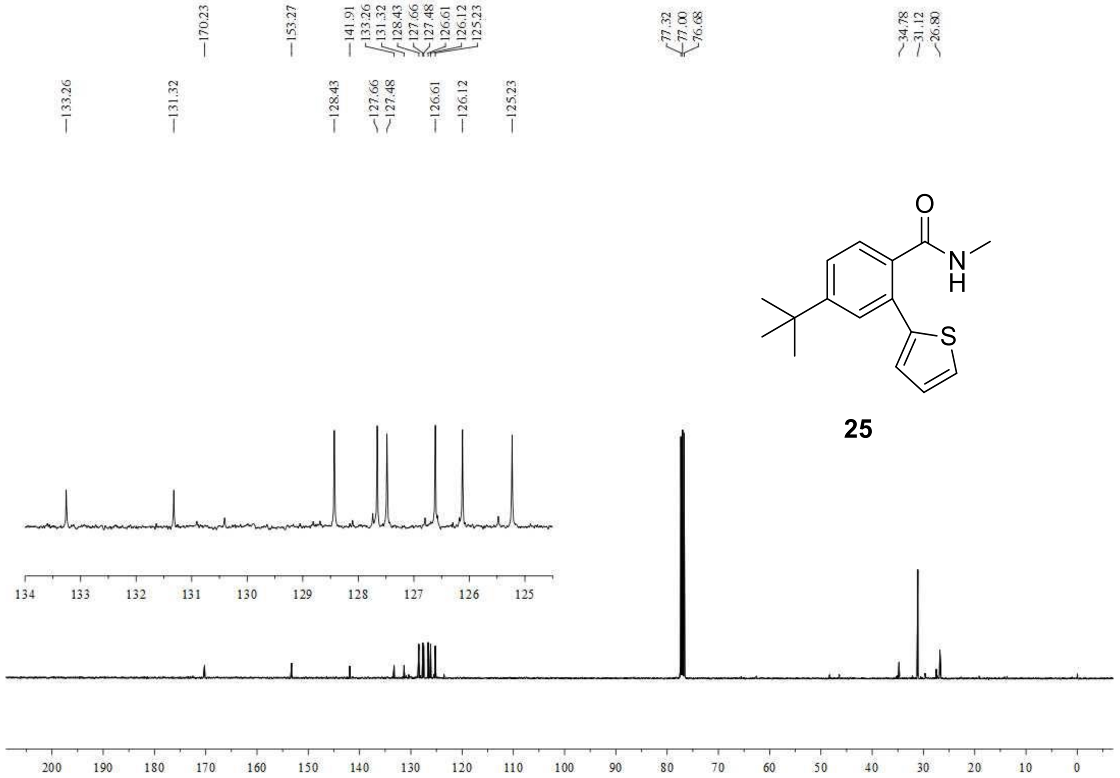

Supplementary Figure 129. ^{13}C NMR Spectrum of substrate 25

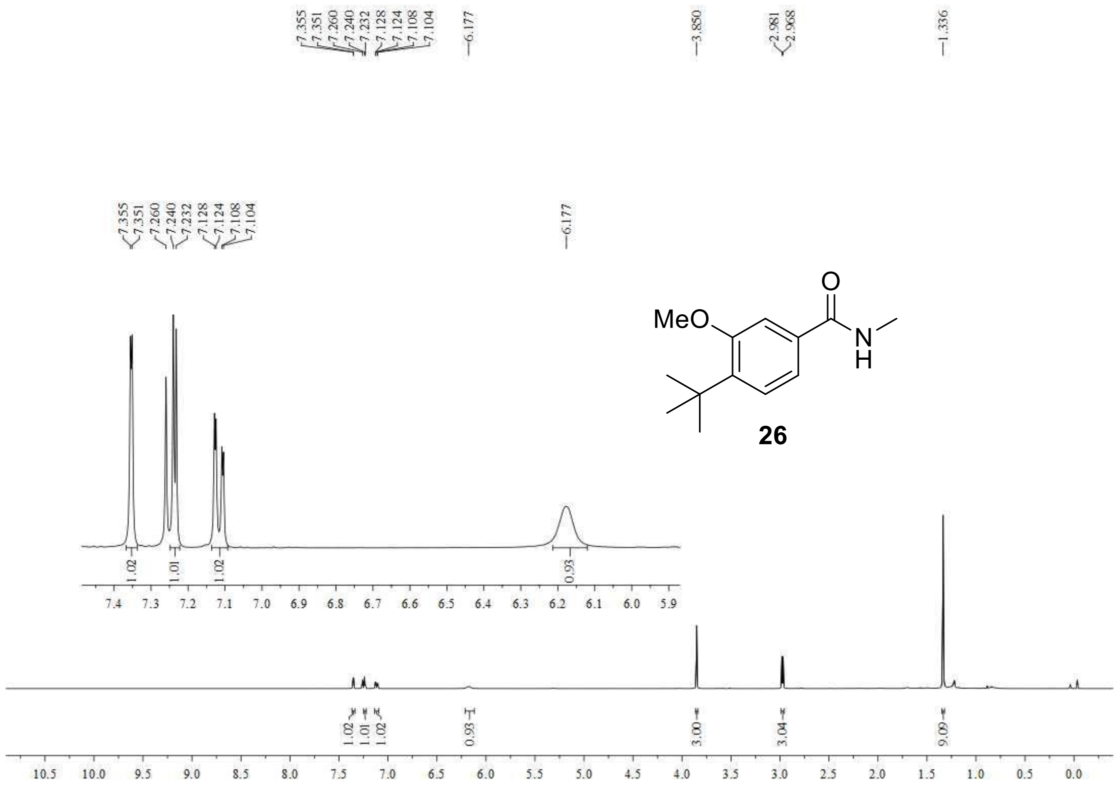

Supplementary Figure 130. ^{1}**H NMR Spectrum of substrate 26**

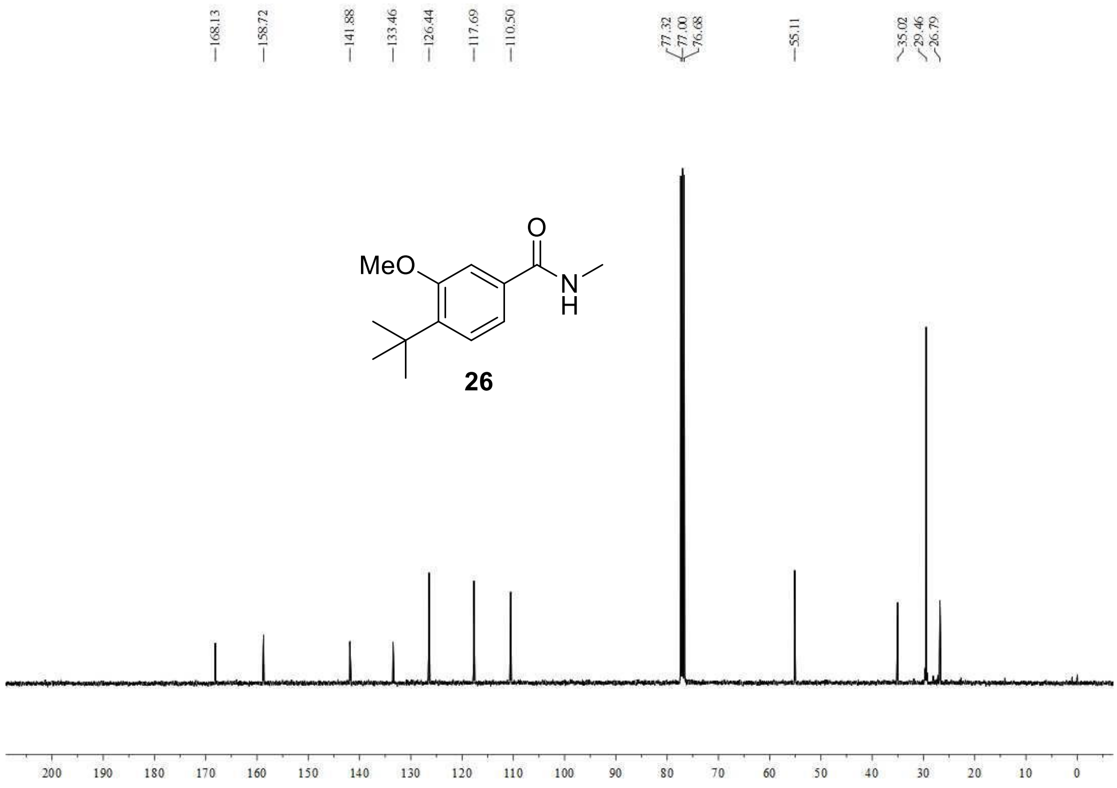

Supplementary Figure 131. ^{13}**C NMR Spectrum of substrate 26**

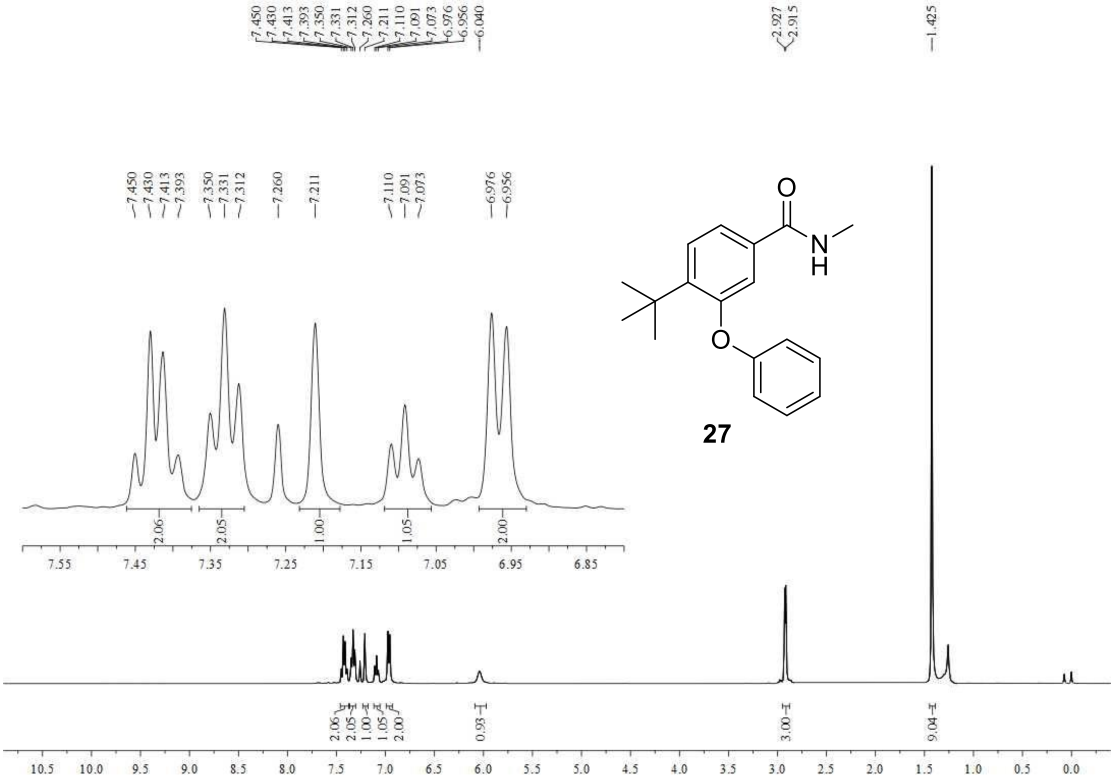

Supplementary Figure 132. ^{1}H NMR Spectrum of substrate 27

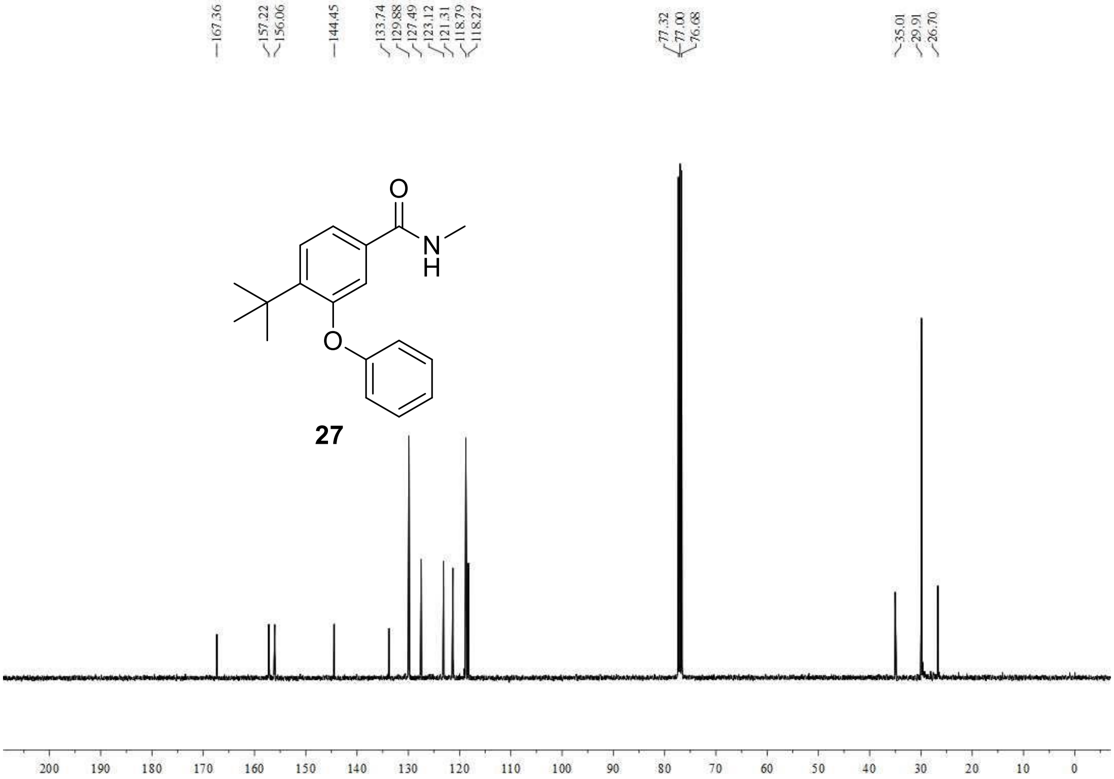

Supplementary Figure 133. ^{13}C NMR Spectrum of substrate 27

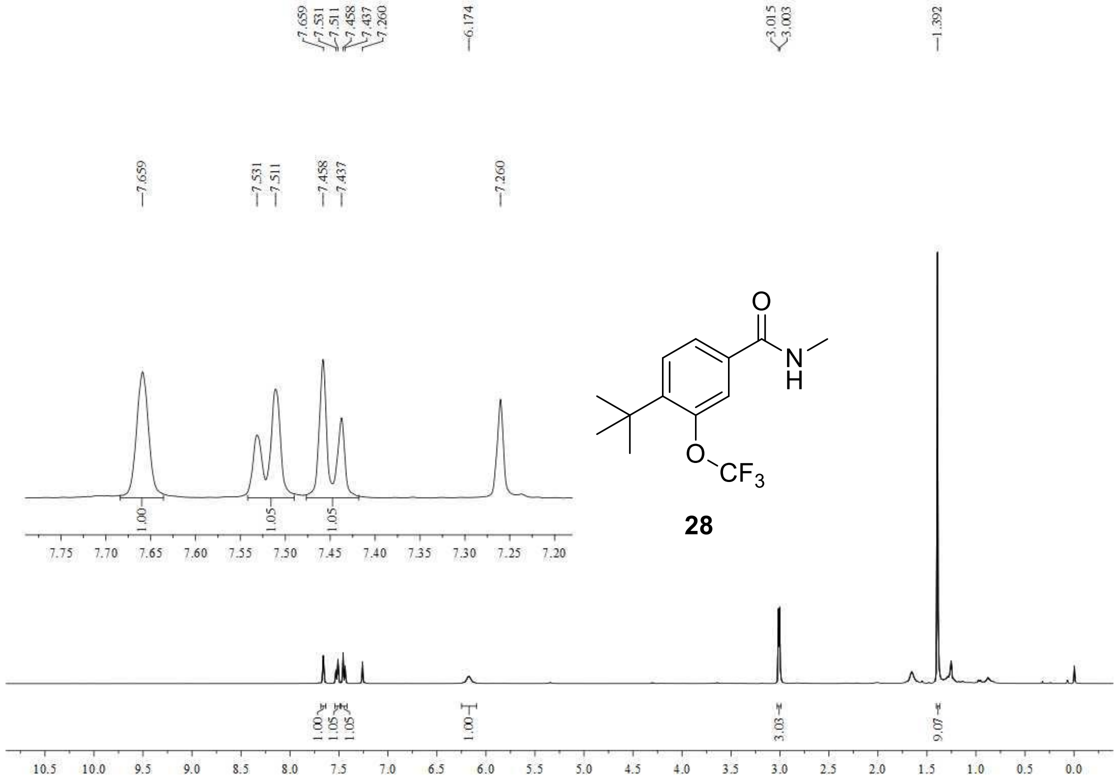

Supplementary Figure 134. ^{1}H NMR Spectrum of substrate 28

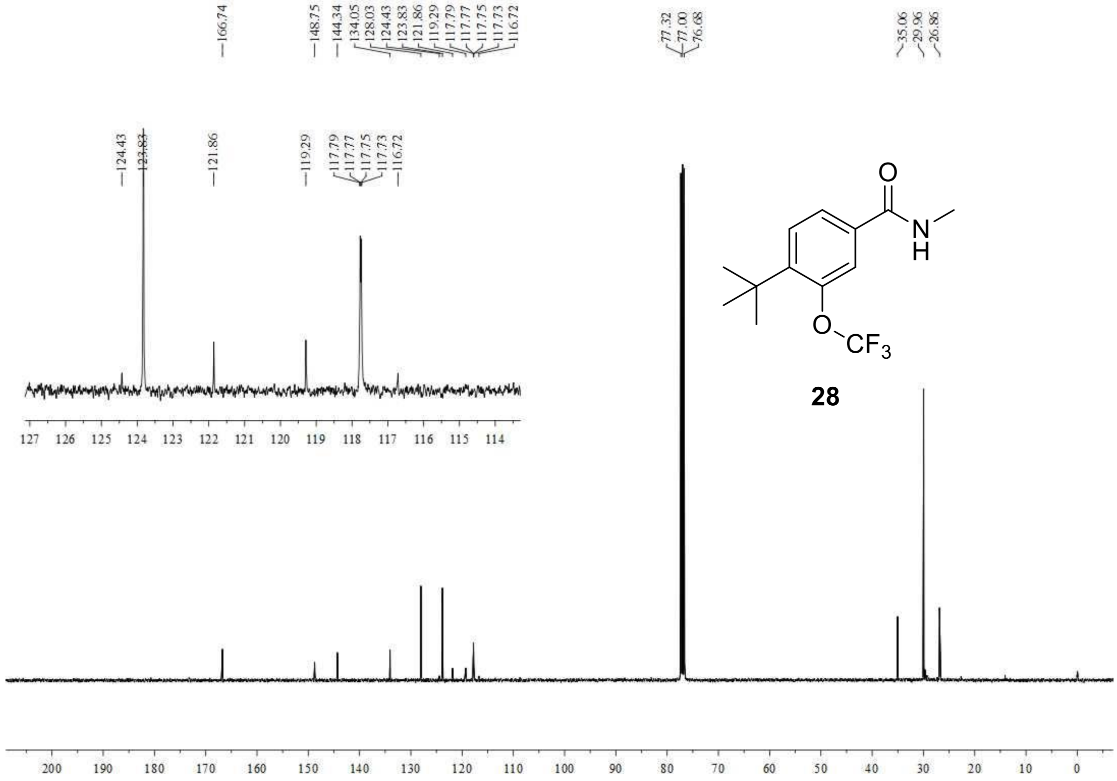

Supplementary Figure 135. ^{13}C NMR Spectrum of substrate 28

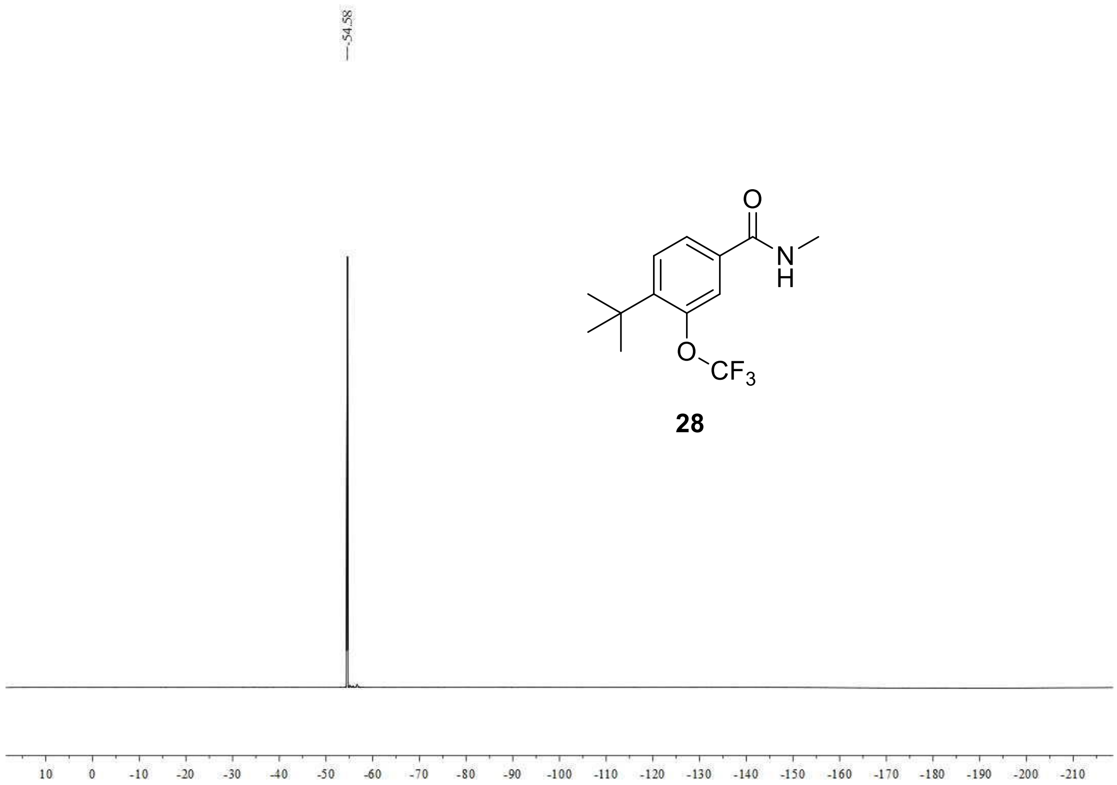

Supplementary Figure 136. ^{19}F NMR Spectrum of substrate 28

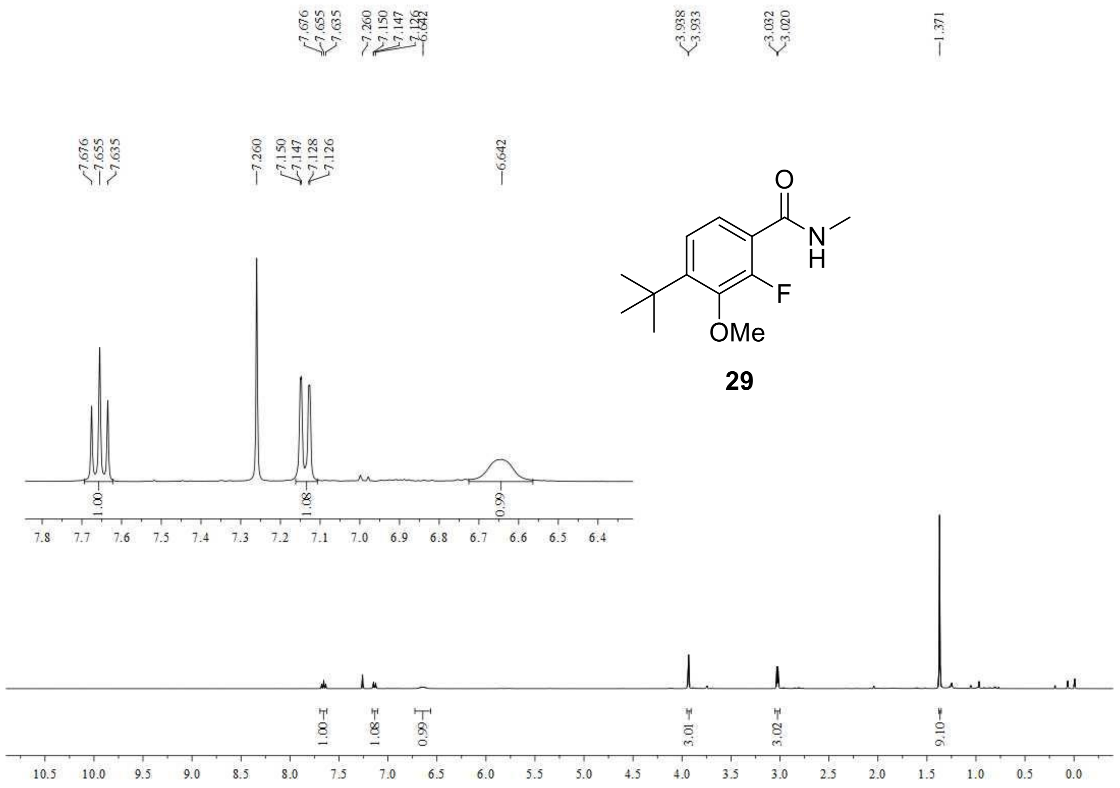

Supplementary Figure 137. ^{1}H NMR Spectrum of substrate 29

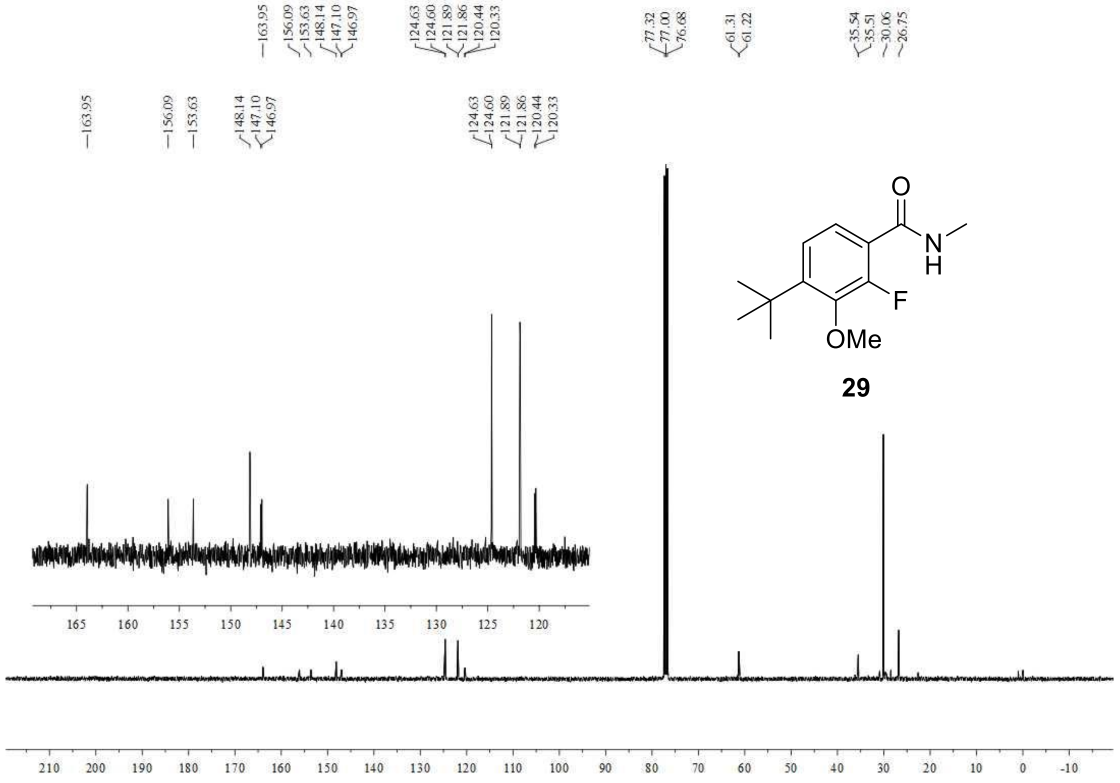

Supplementary Figure 138. ^{13}C NMR Spectrum of substrate 29

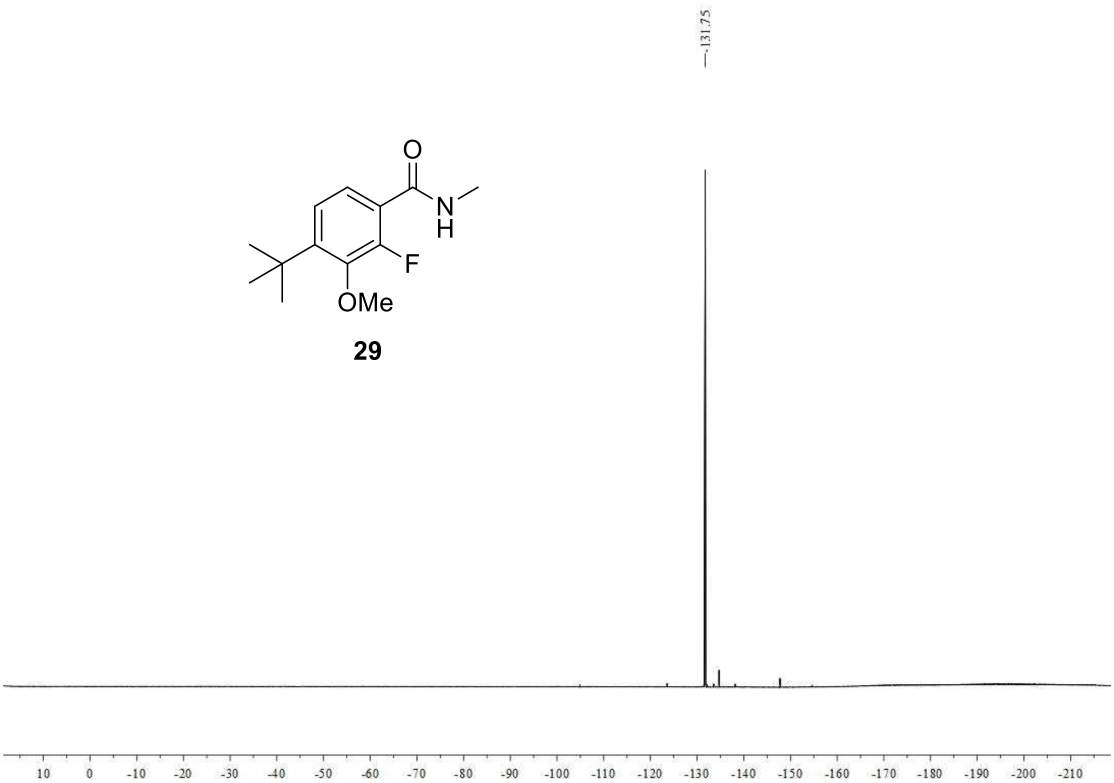

Supplementary Figure 139. ^{19}F NMR Spectrum of substrate 29

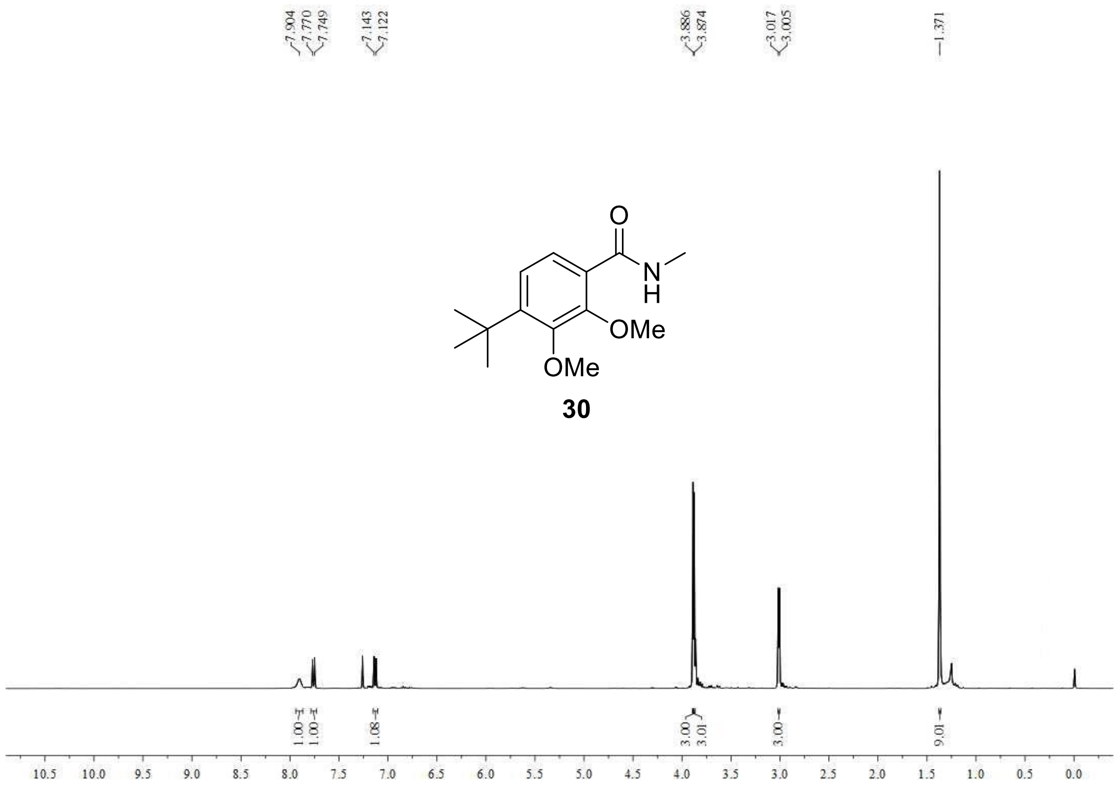

Supplementary Figure 140. ^{1}H NMR Spectrum of substrate 30

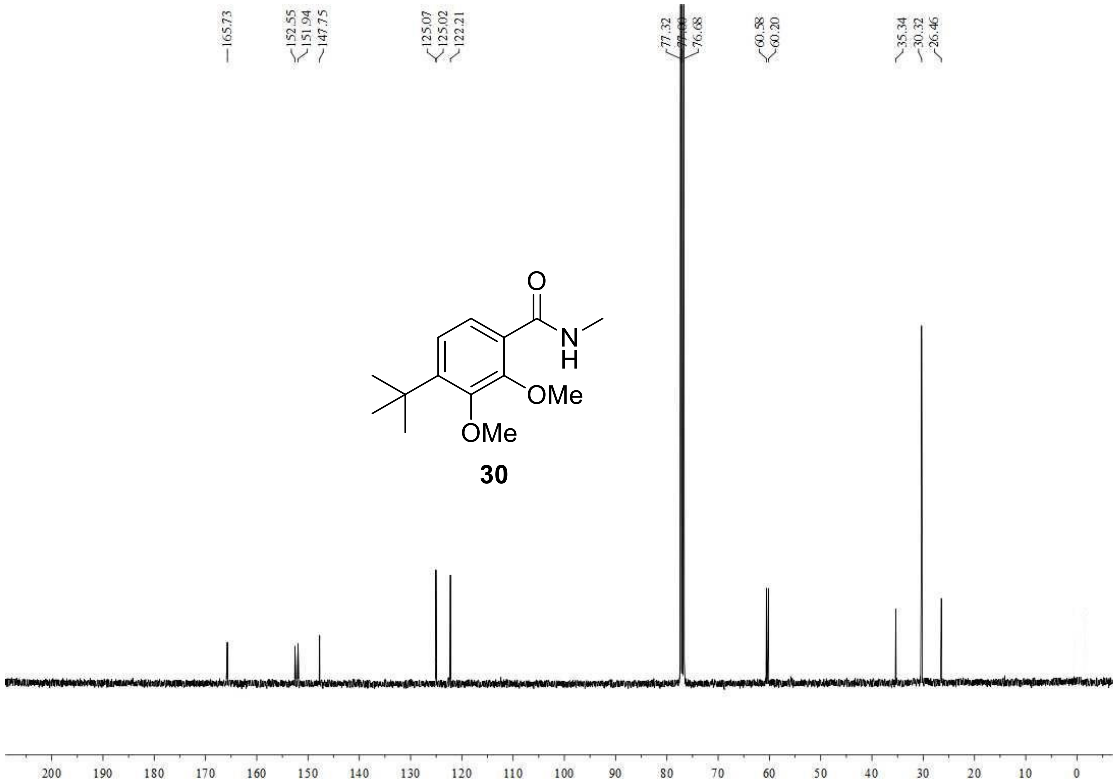

Supplementary Figure 141. ^{13}C NMR Spectrum of substrate 30

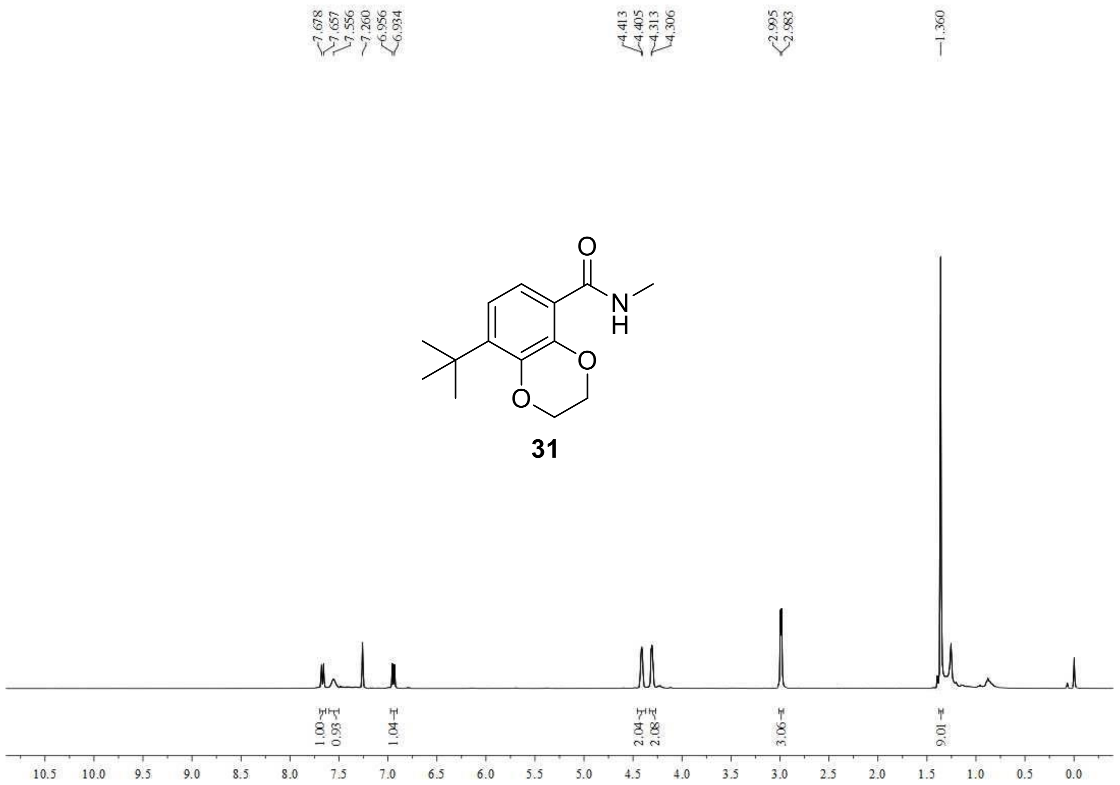

Supplementary Figure 142. ^{1}H NMR Spectrum of substrate 31

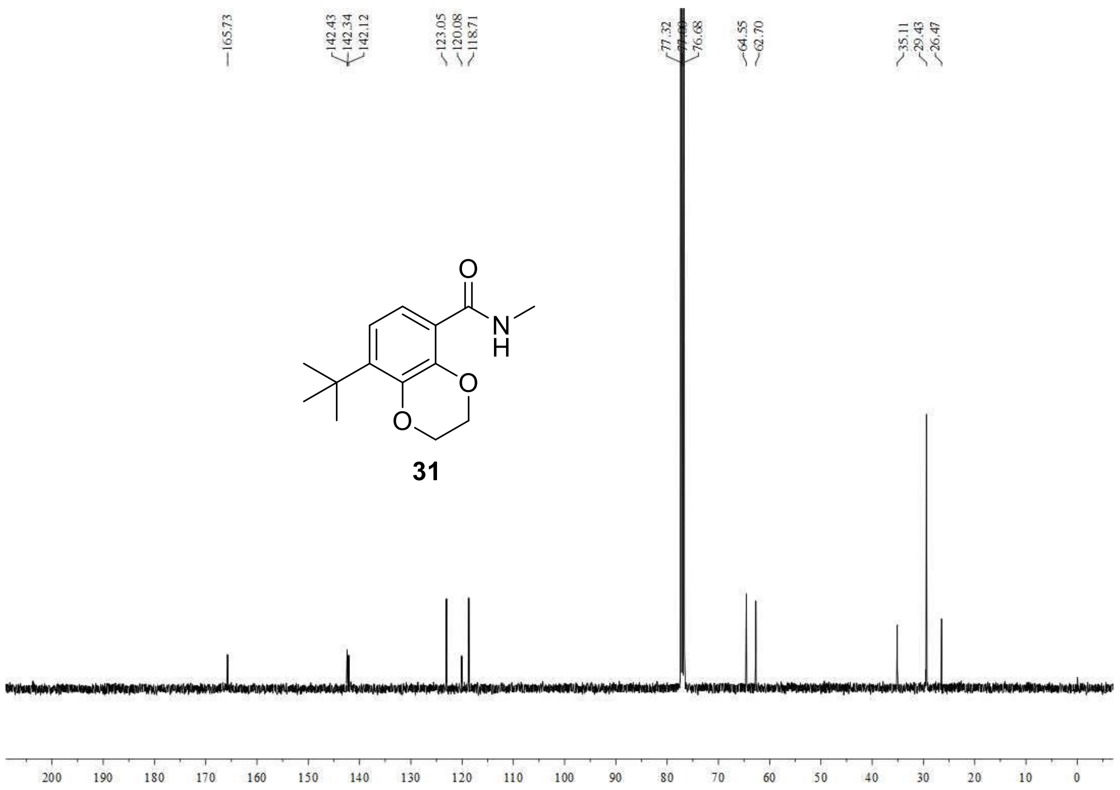

Supplementary Figure 143. ^{13}C NMR Spectrum of substrate 31

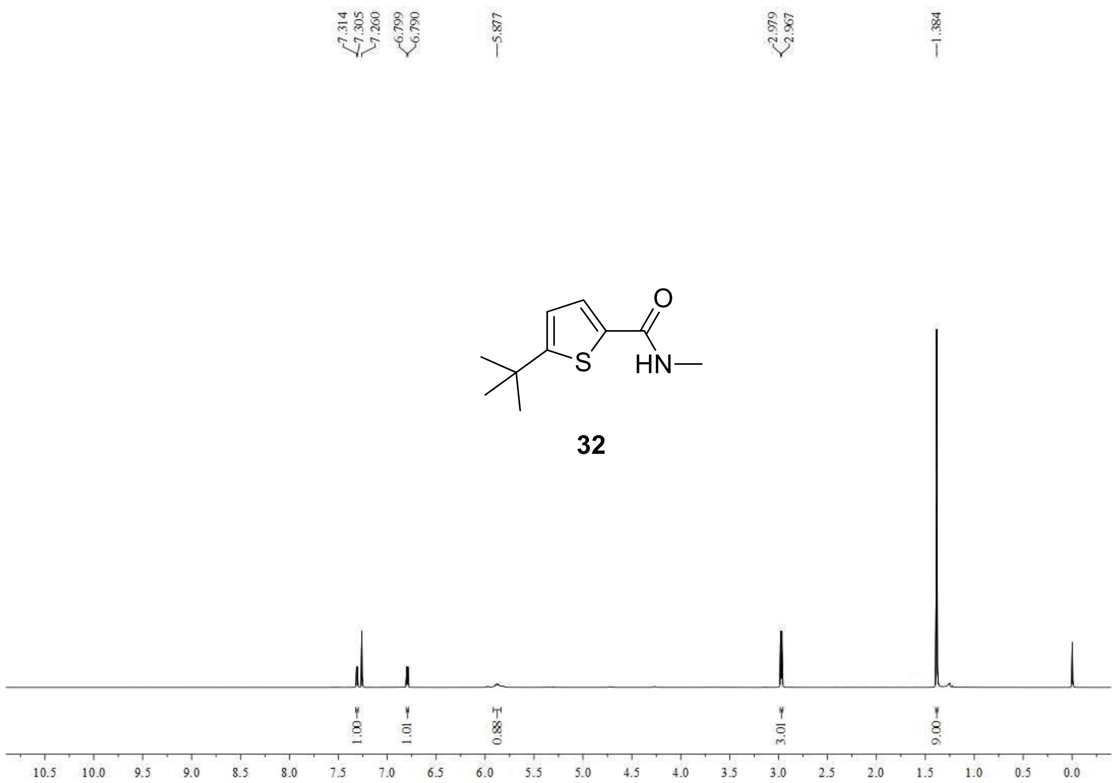

Supplementary Figure 144. ^{1}H NMR Spectrum of substrate 32

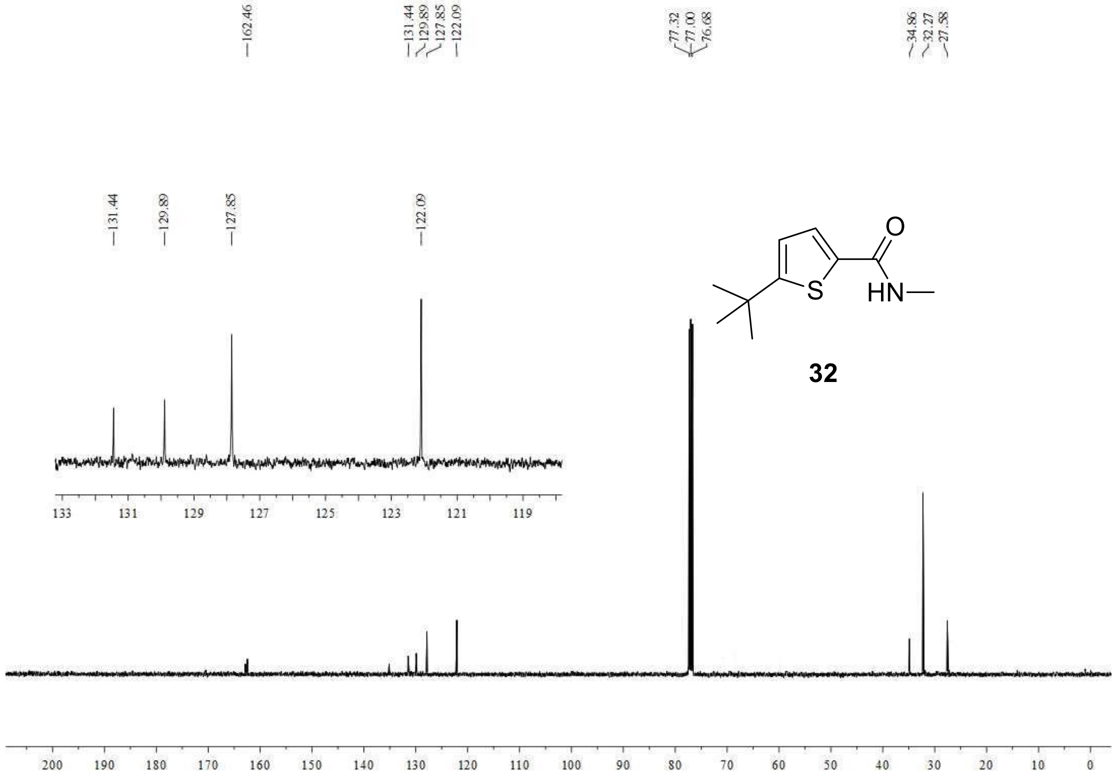

Supplementary Figure 145. ^{13}C NMR Spectrum of substrate 32

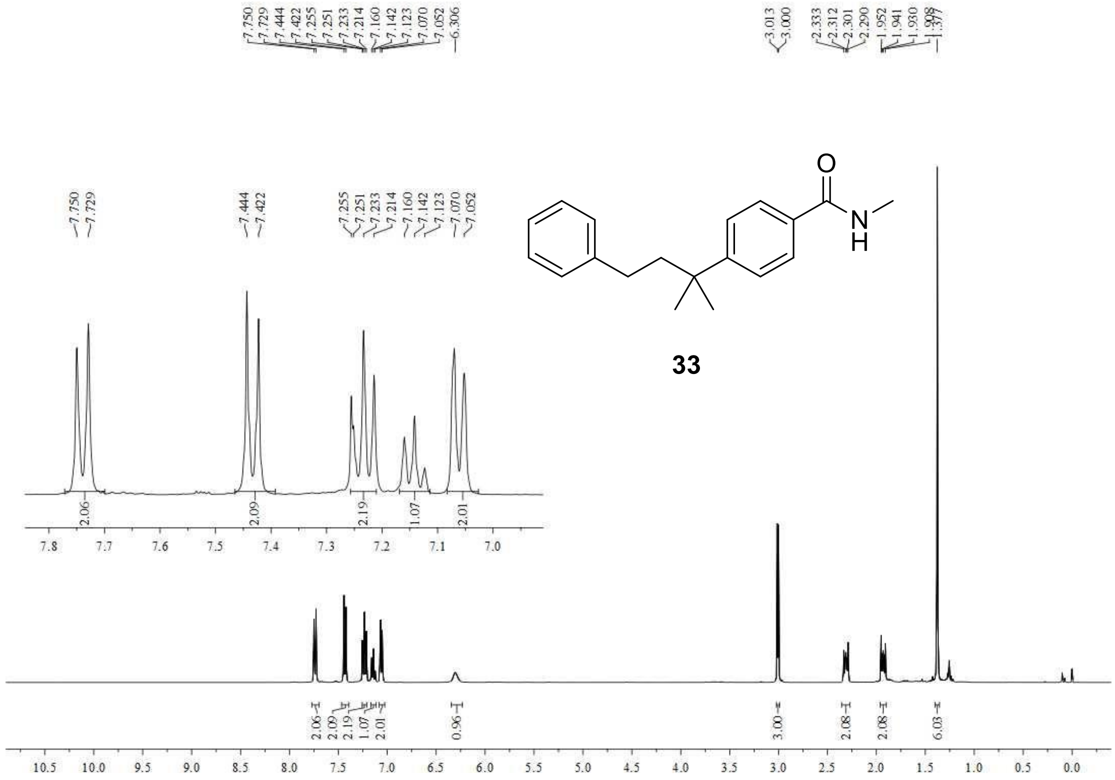

Supplementary Figure 146. ^{1}H NMR Spectrum of substrate 33

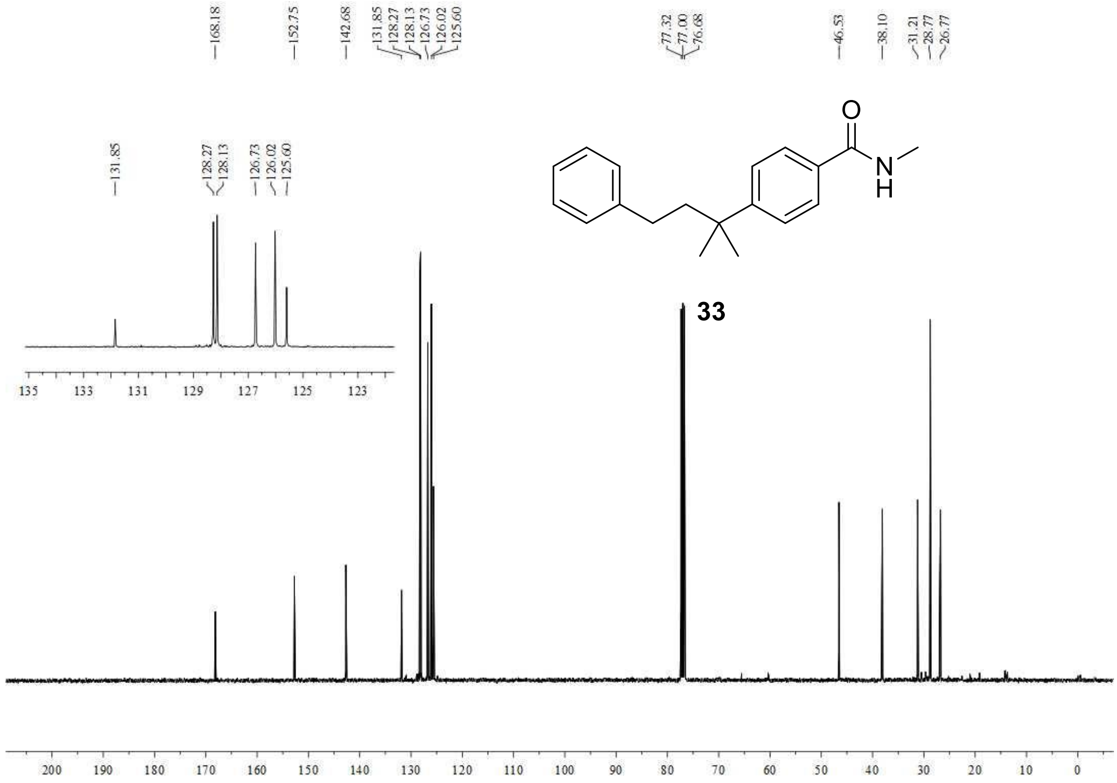

Supplementary Figure 147. ^{13}C NMR Spectrum of substrate 33

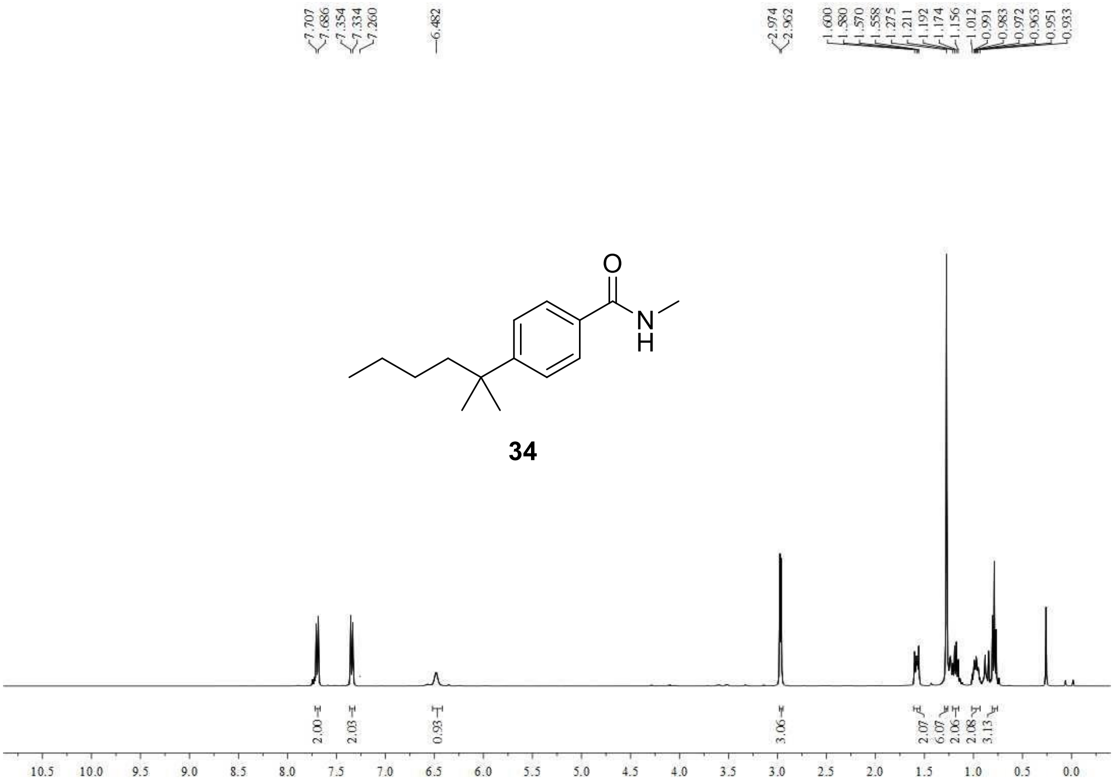

Supplementary Figure 148. ^{1}H NMR Spectrum of substrate 34

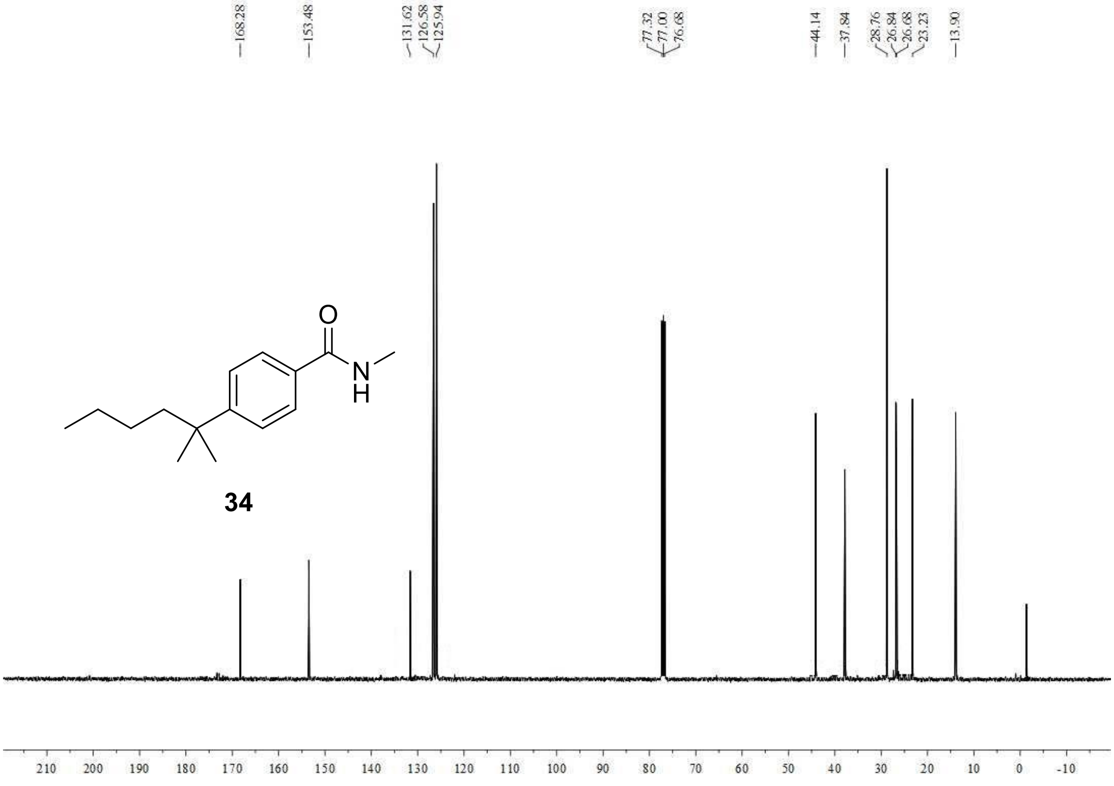

Supplementary Figure 149. ^{13}C NMR Spectrum of substrate 34

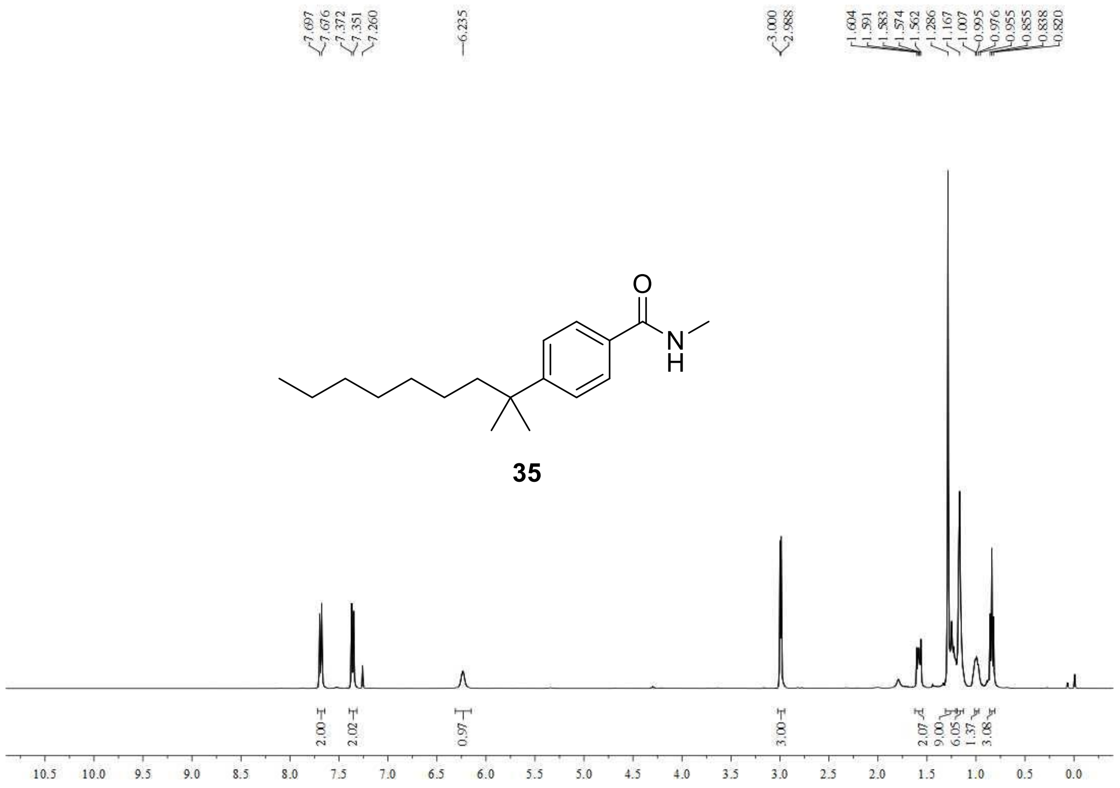

Supplementary Figure 150. ^{1}H NMR Spectrum of substrate 35

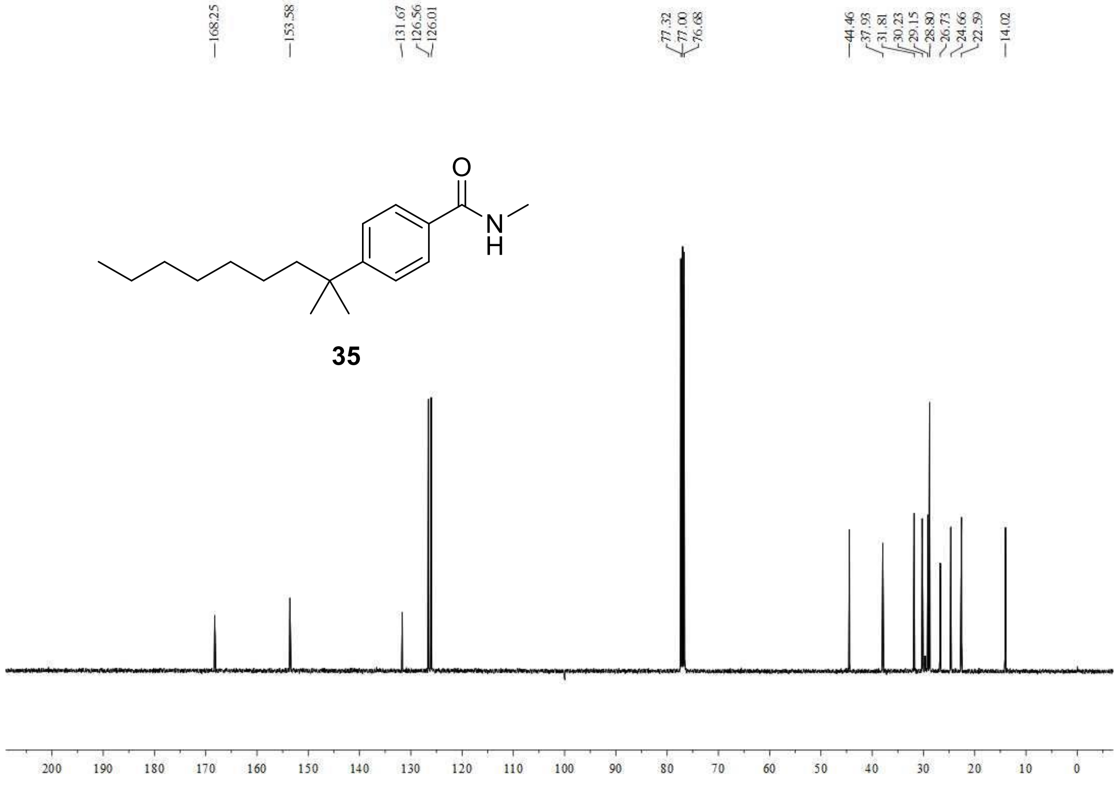

Supplementary Figure 151. ^{13}C NMR Spectrum of substrate 35

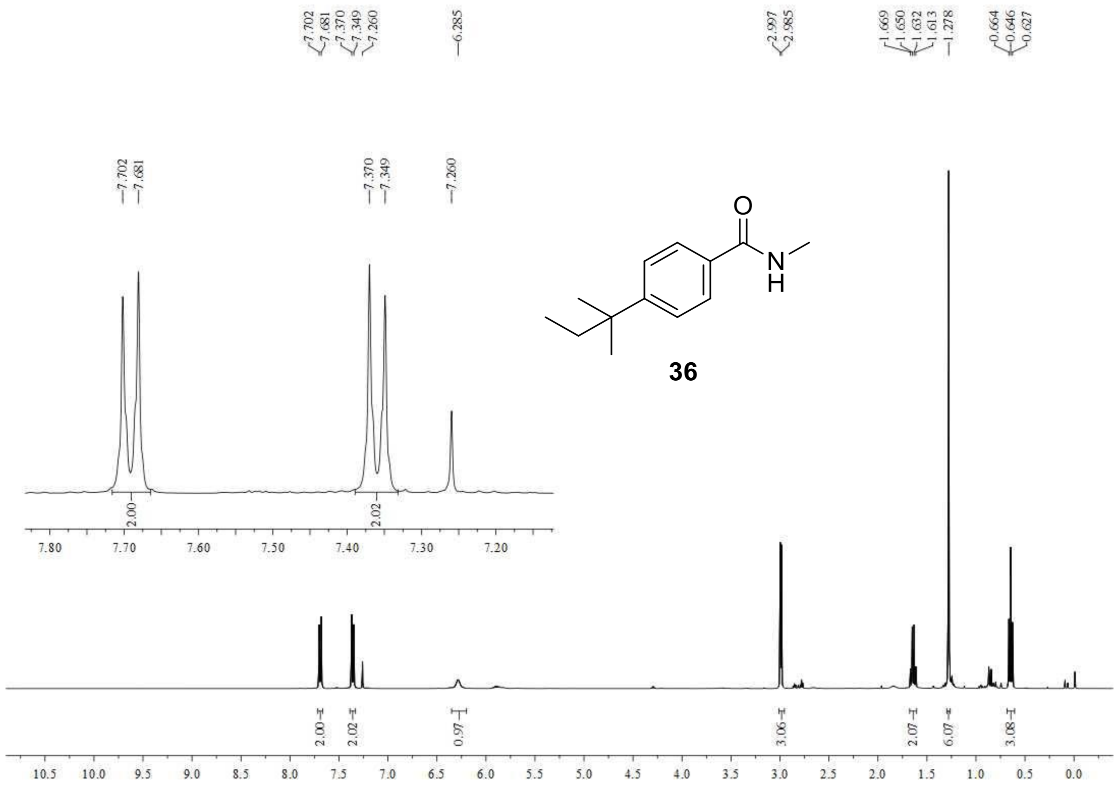

Supplementary Figure 152. ^{1}H NMR Spectrum of substrate 36

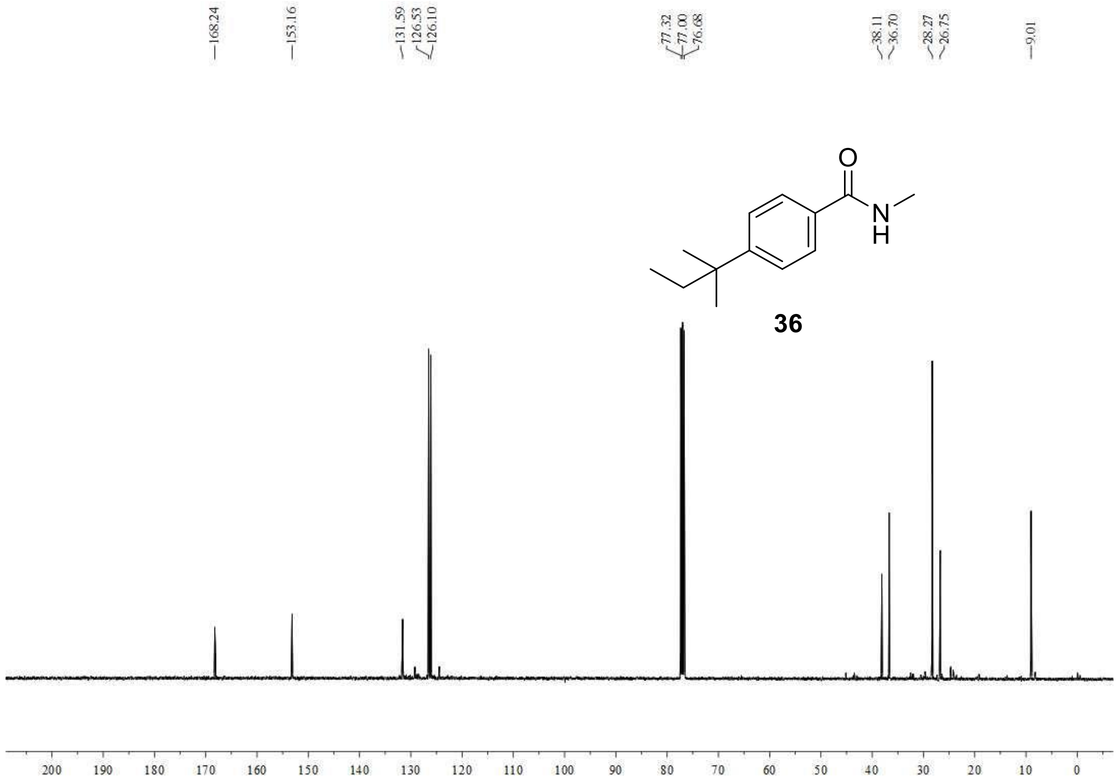

Supplementary Figure 153. ^{13}C NMR Spectrum of substrate 36

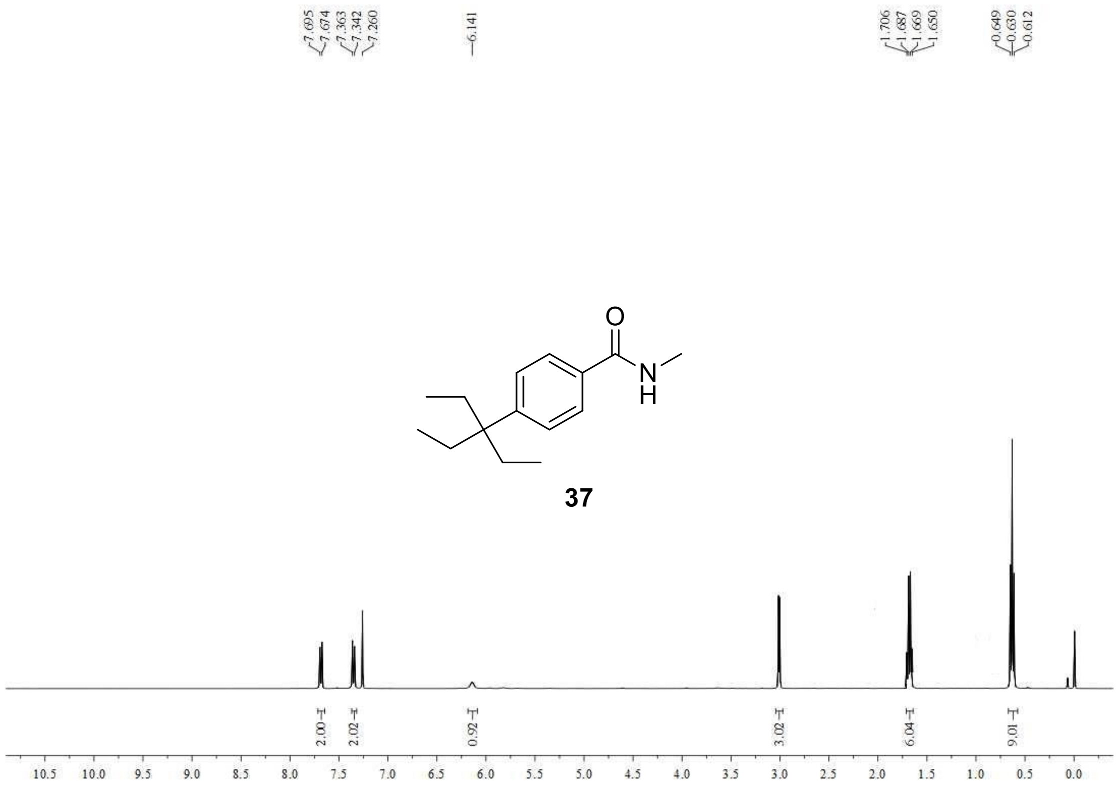

Supplementary Figure 154. ^{1}H NMR Spectrum of substrate 37

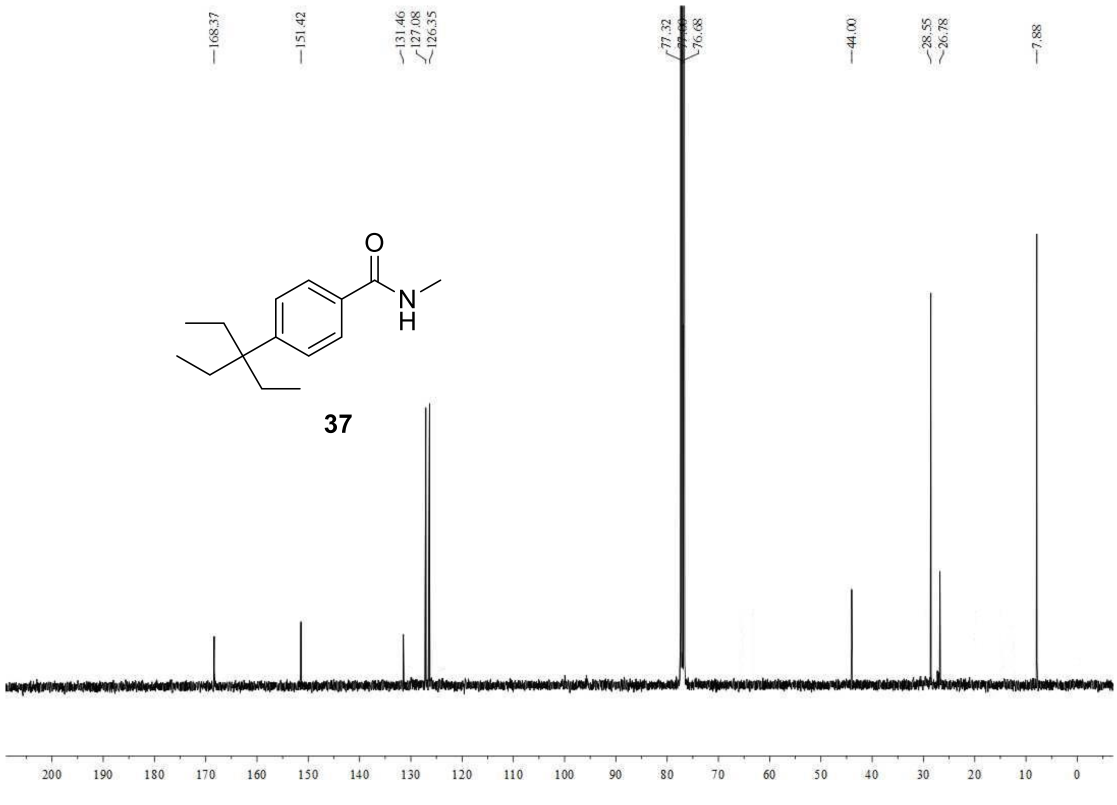

Supplementary Figure 155. ^{13}C NMR Spectrum of substrate 37

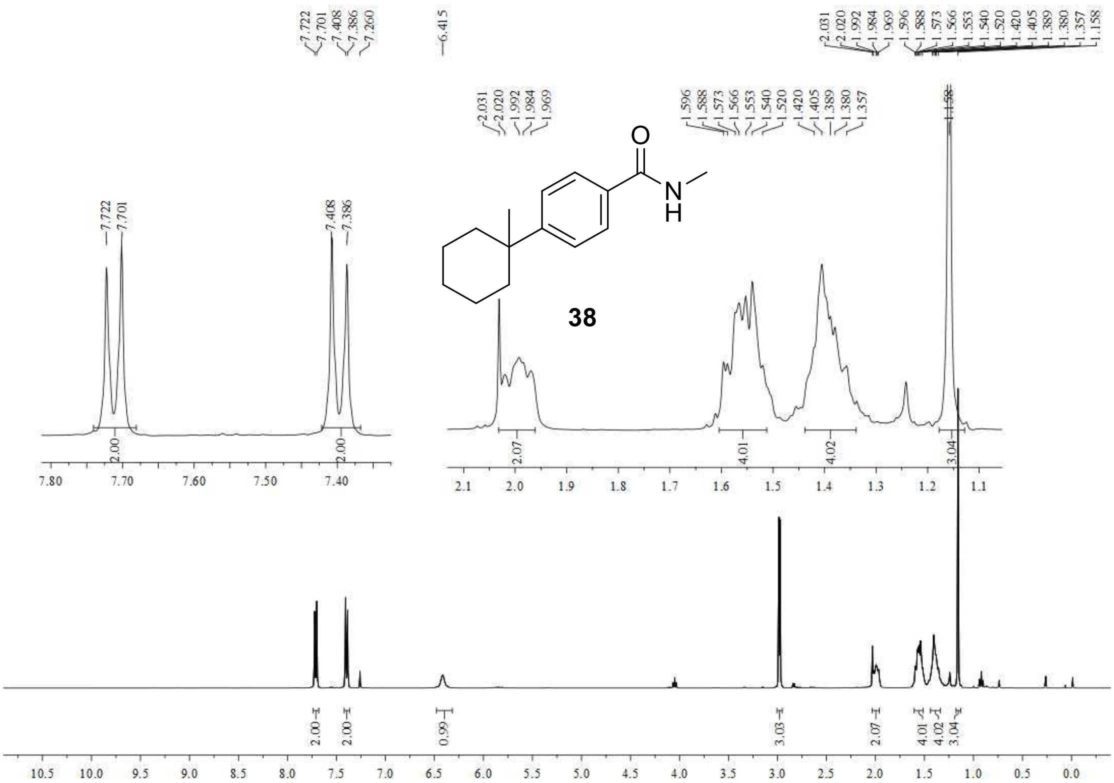

Supplementary Figure 156. ^{1}H NMR Spectrum of substrate 38

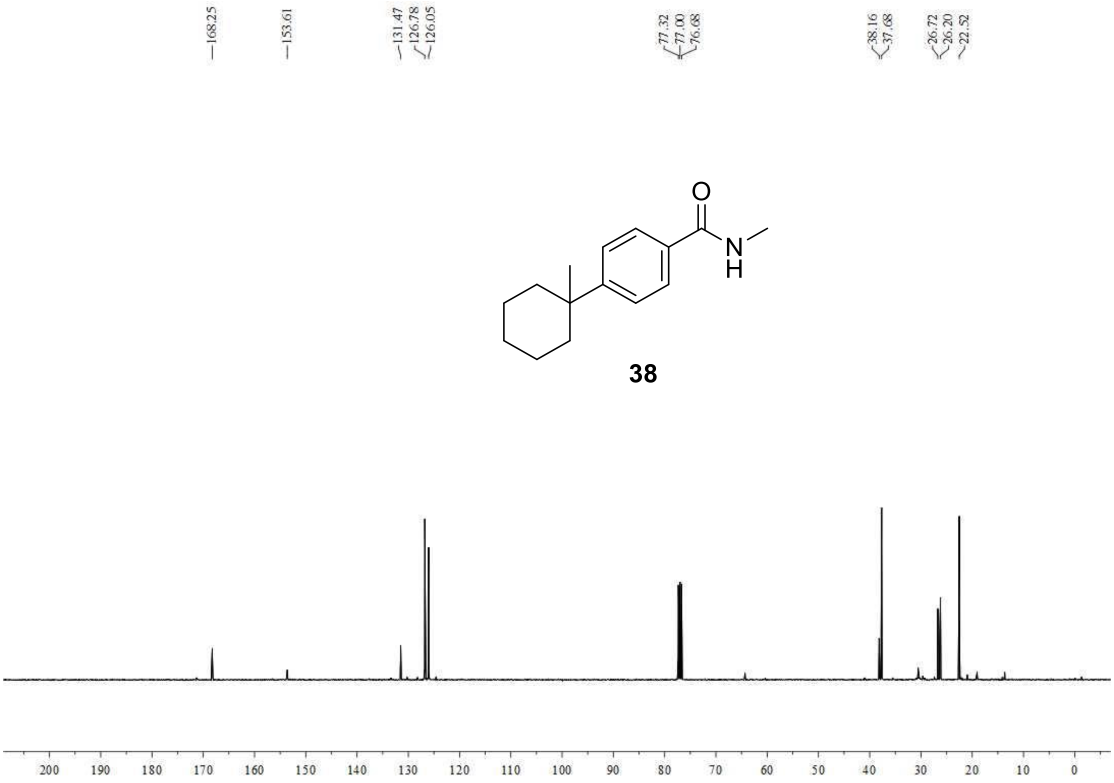

Supplementary Figure 157. ^{13}C NMR Spectrum of substrate 38

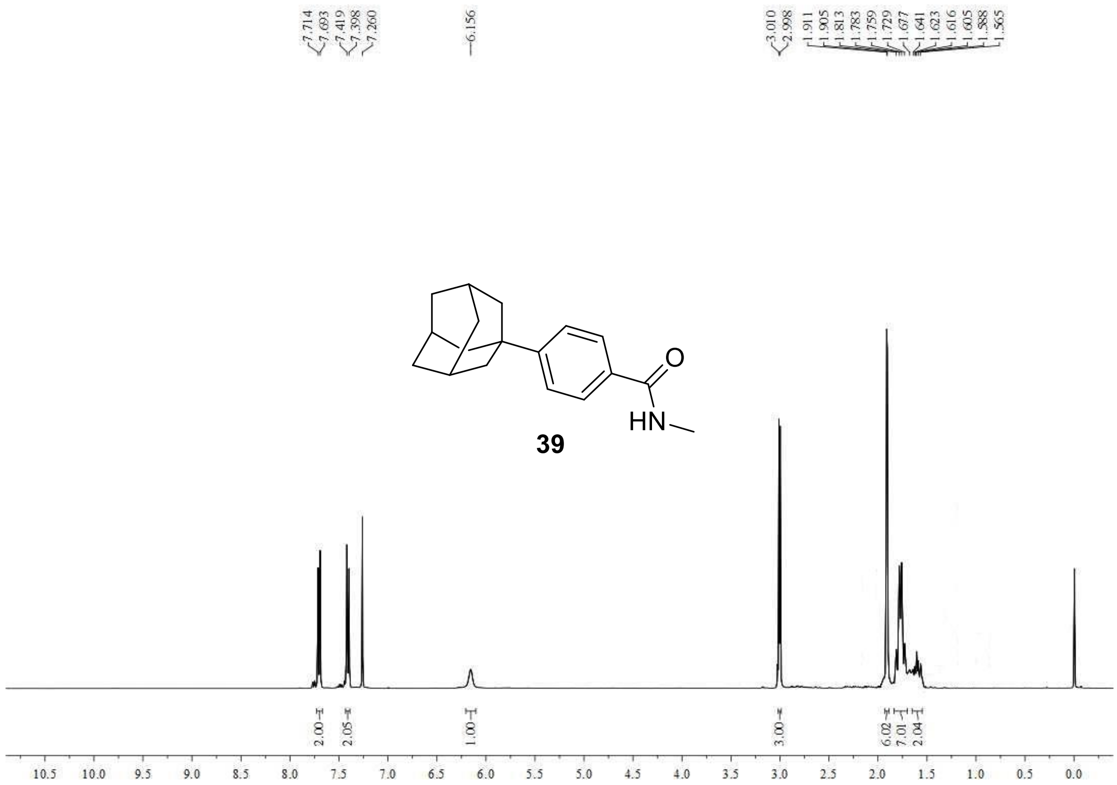

Supplementary Figure 158. ^{1}H NMR Spectrum of substrate 39

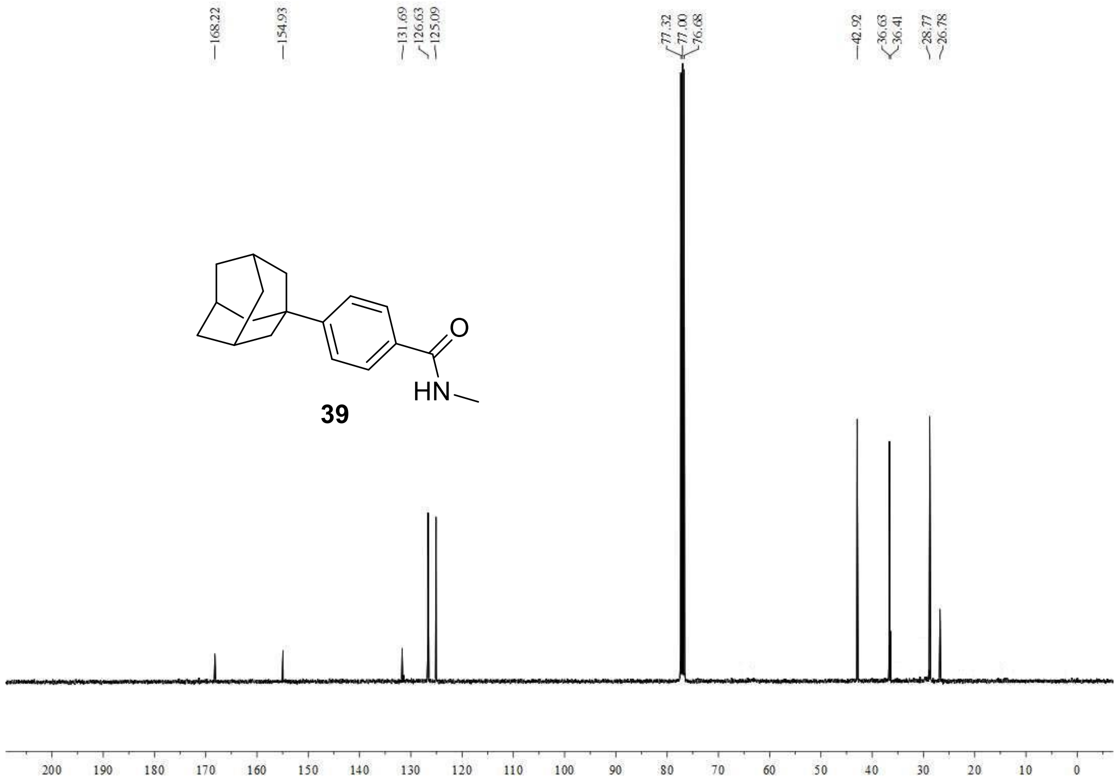

Supplementary Figure 159. ^{13}C NMR Spectrum of substrate 39

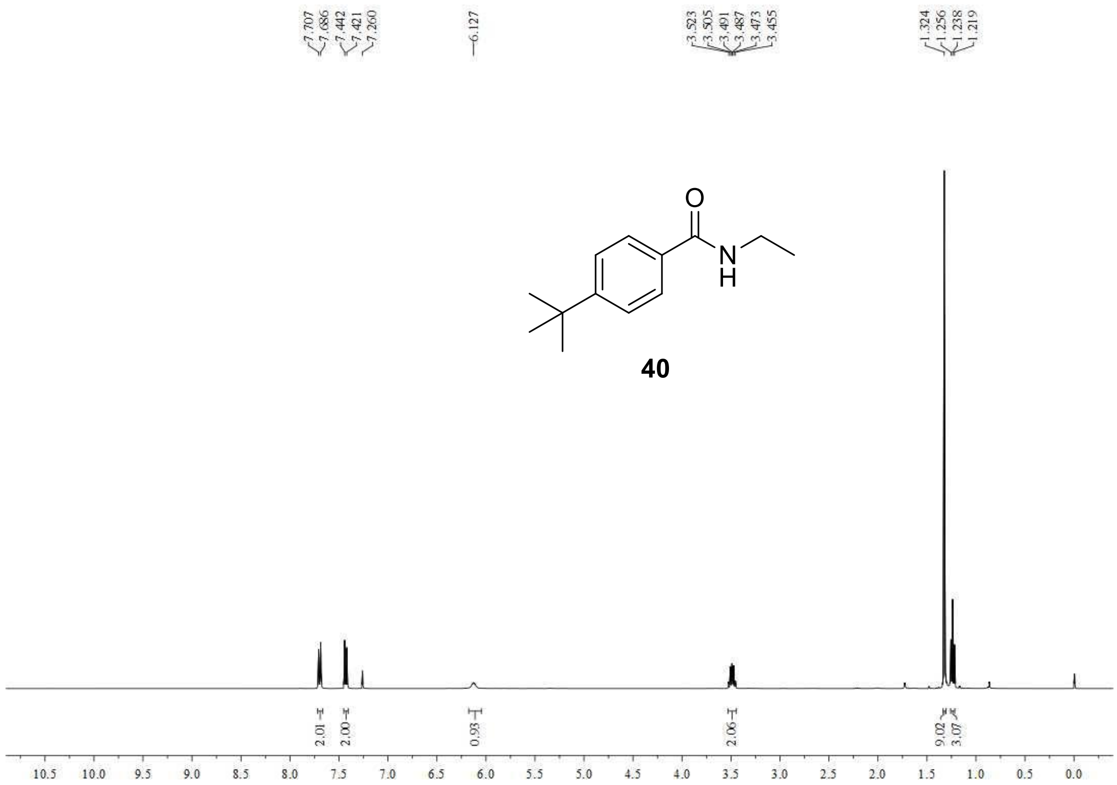

Supplementary Figure 160. ^{1}H NMR Spectrum of substrate 40

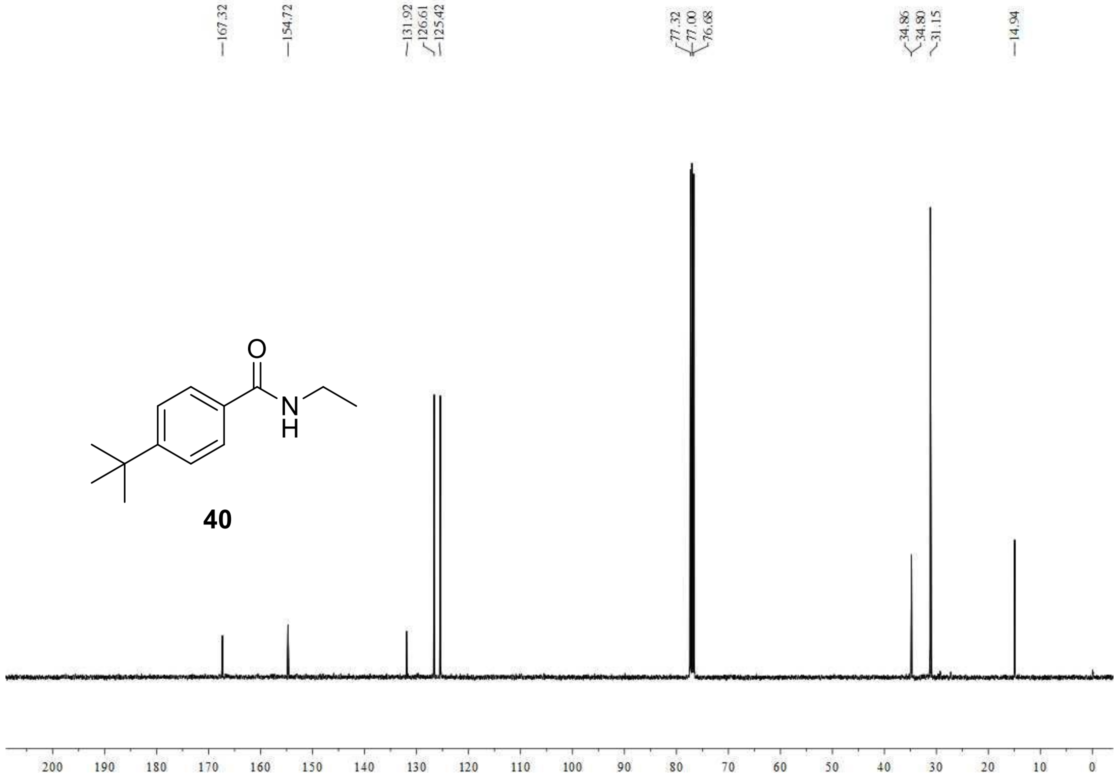

Supplementary Figure 161. ^{13}C NMR Spectrum of substrate 40

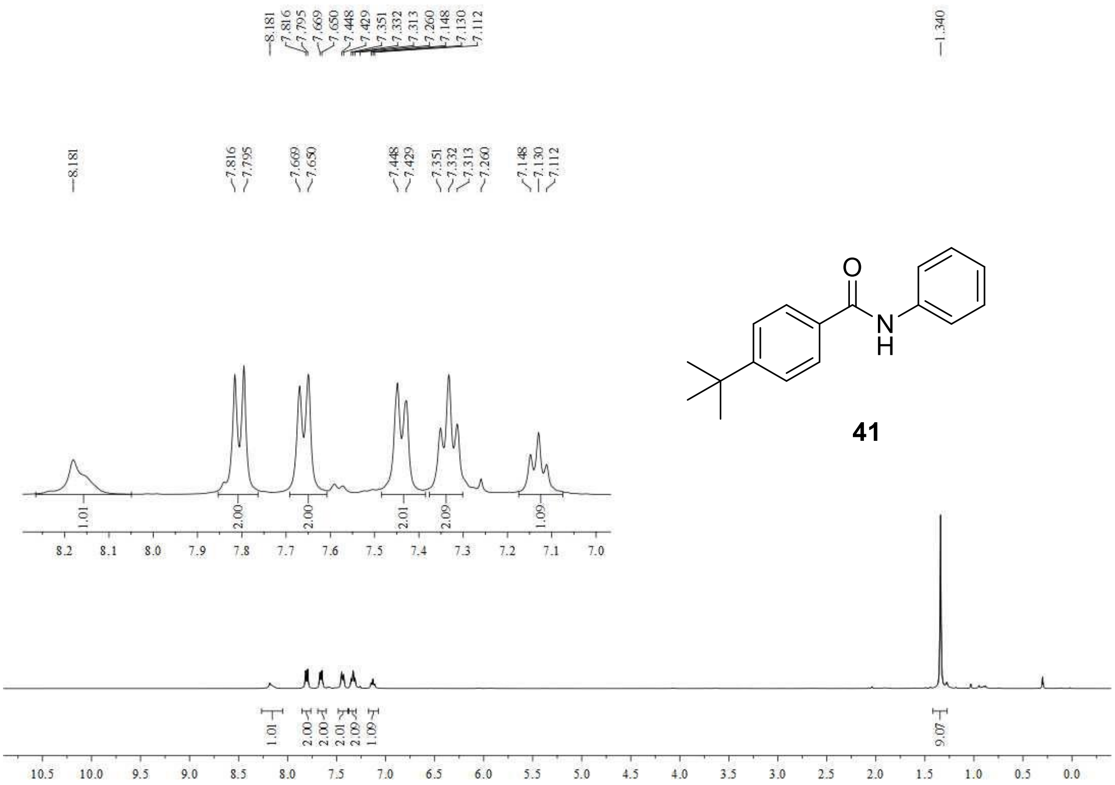

Supplementary Figure 162. ^{1}H NMR Spectrum of substrate 41

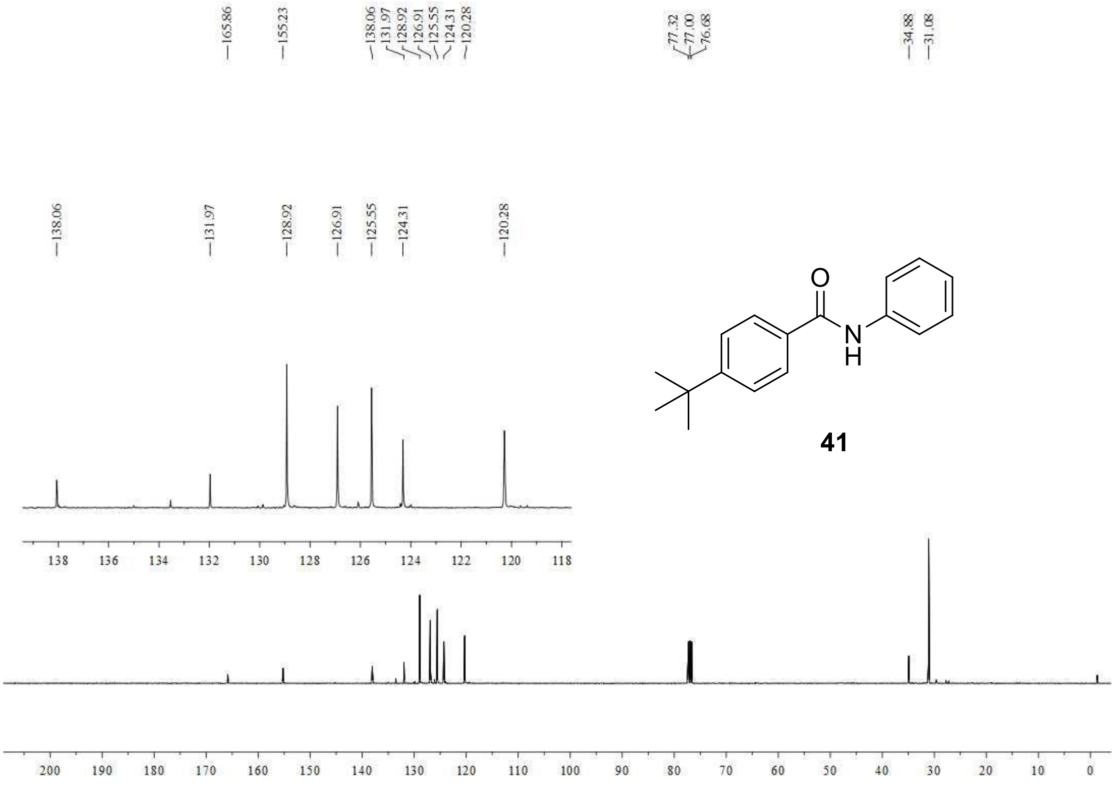

Supplementary Figure 163. ^{13}C NMR Spectrum of substrate 41

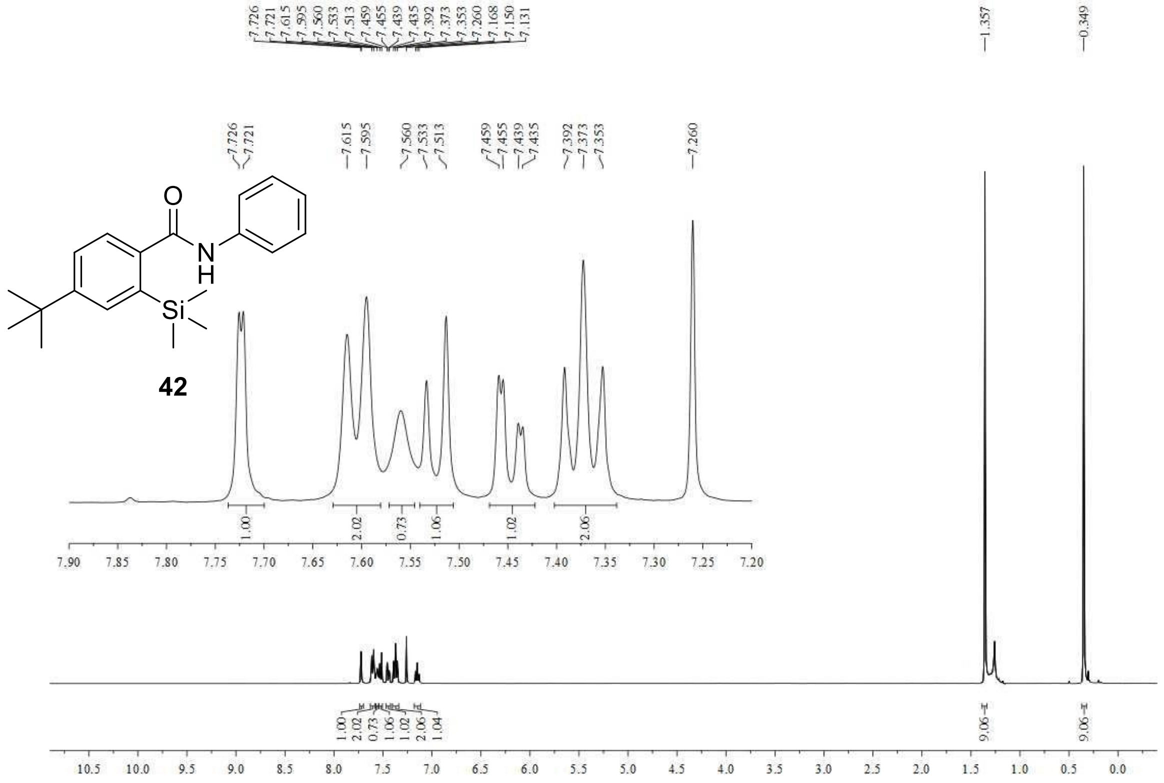

Supplementary Figure 164. ^{1}H NMR Spectrum of substrate 42

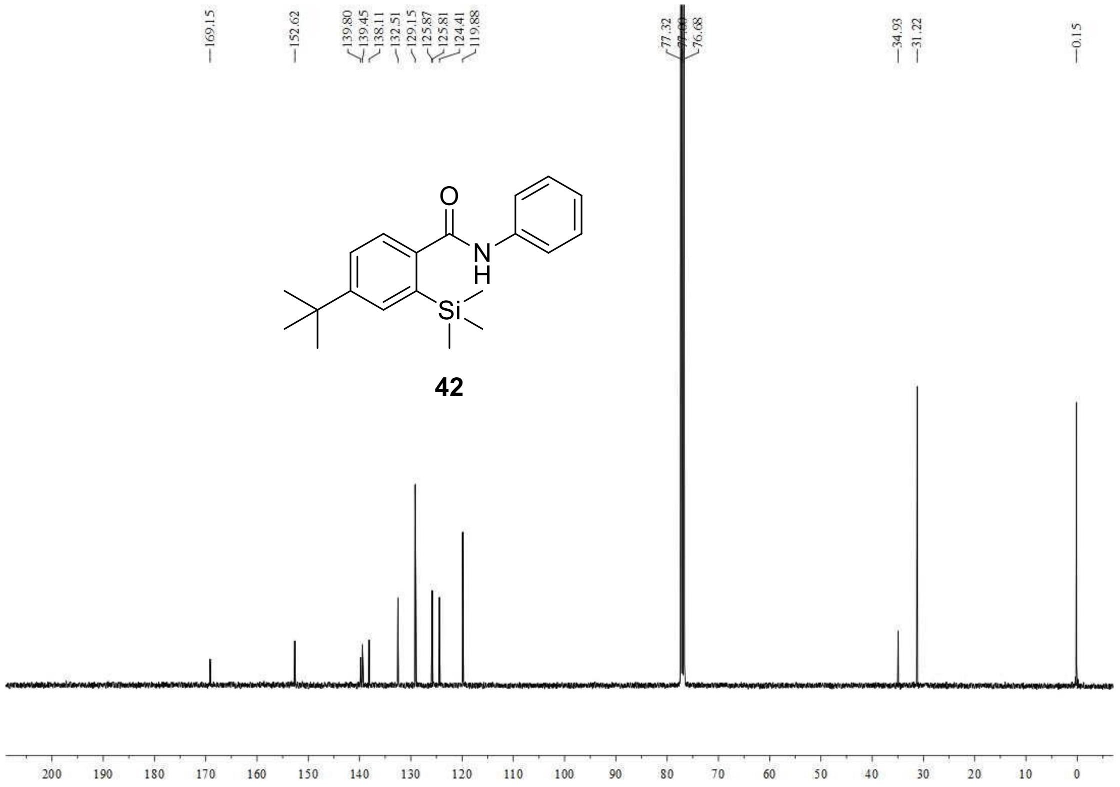

Supplementary Figure 165. ^{13}C NMR Spectrum of substrate 42

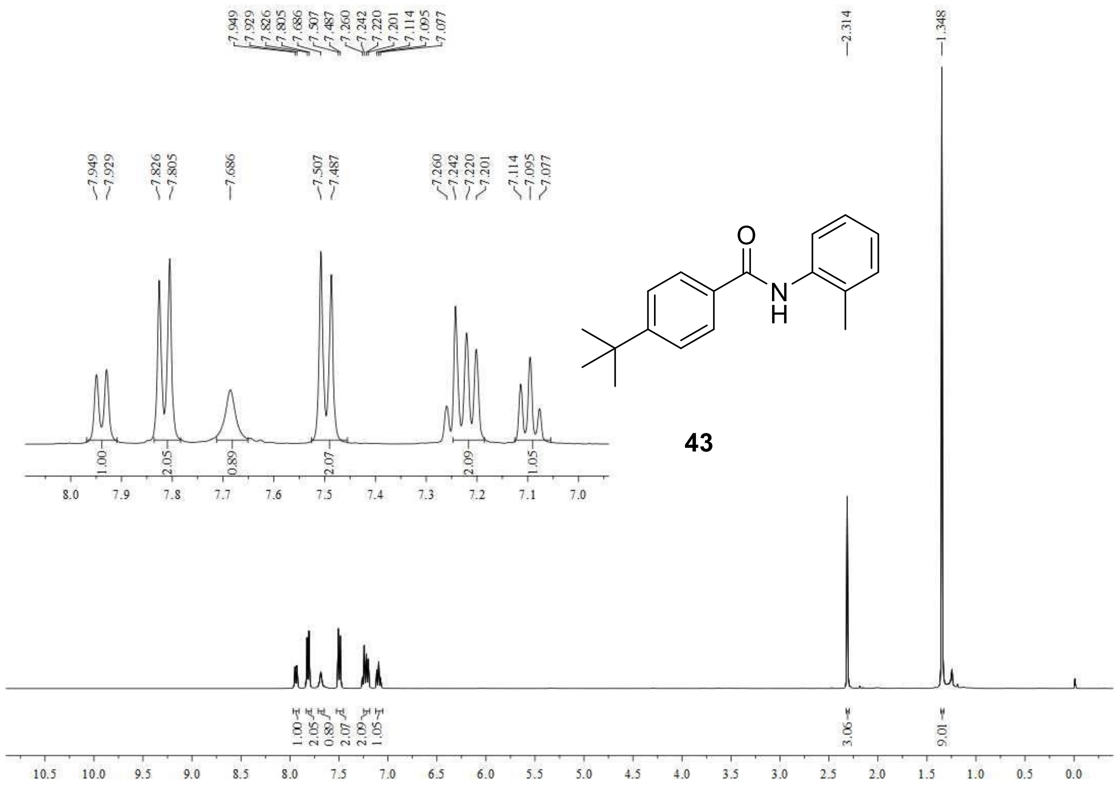

Supplementary Figure 166. ^{1}H NMR Spectrum of substrate 43

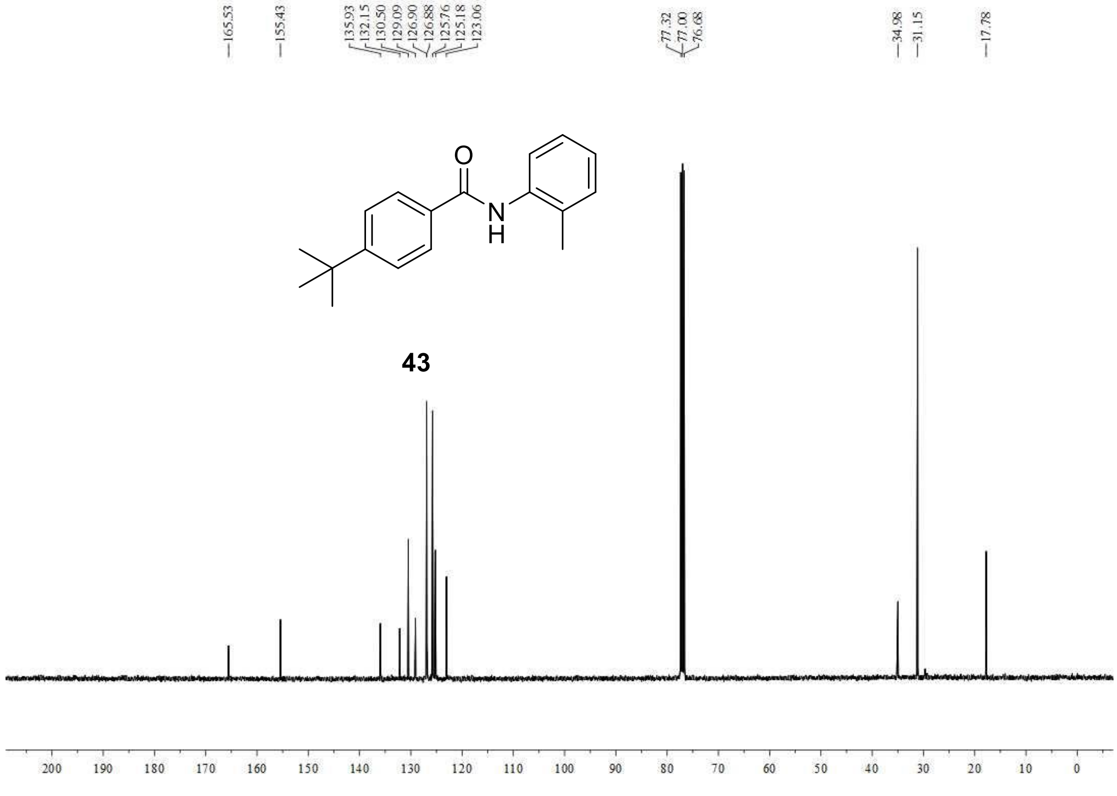

Supplementary Figure 167. ^{13}C NMR Spectrum of substrate 43

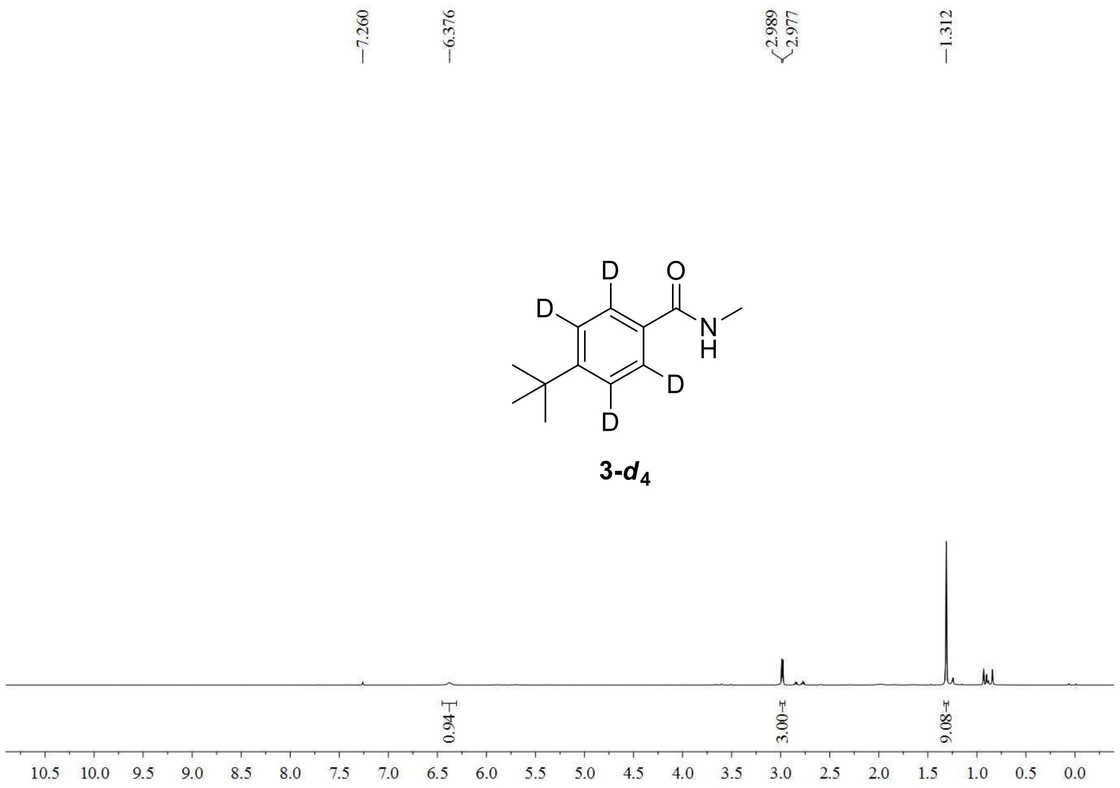

Supplementary Figure 168. ^{1}H NMR Spectrum of substrate 3-*d*$_4$

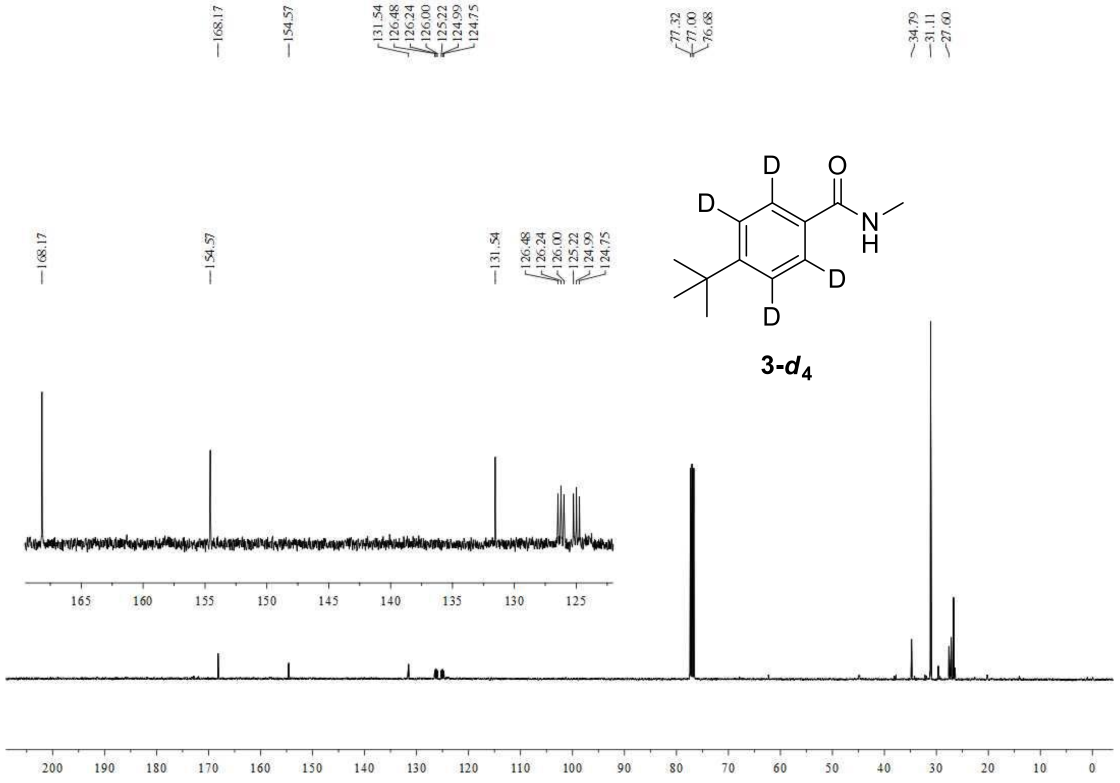

Supplementary Figure 169. ^{13}C NMR Spectrum of substrate 3-*d*$_4$

Supplementary References

1. Still, W. C., Kahn, M. & Mitra, A. Rapid chromatographic technique for preparative separations with moderate resolution. *J. Org. Chem*. **43**, 2923–2925 (1978).
2. Jo, Y., Ju, J. H., Choe, J., Song, K. H. & Lee, S. The scope and limitation of nickel-catalyzed aminocarbonylation of aryl bromides from formamide derivatives. *J. Org. Chem.* **74**, 6358–6361 (2009).
3. Hyster, T. K. & Rovis, T. Rhodium-catalyzed oxidative cycloaddition of benzamides and alkynes *via* C–H/N–H activation. *J. Am. Chem. Soc.* **132**, 10565–10569 (2010).
4. Galan, J. F., Brown, J., Wildin, J. L., Liu, Z., Liu, D., Moyna, G. & Pophristic, V. Intramolecular hydrogen bonding in *ortho*–substituted arylamide oligomers: A computational and experimental study of *ortho*–fluoro–and *ortho*–chloro–*N*–methylbenzamides. *J. Phys. Chem. B.* **113**, 12809–12815 (2009).
5. Liu, P., Tang, J. & Zeng, X. Site-selective silylation of aliphatic C−H bonds mediated by [1,5]−hydrogen transfer: Synthesis of *α*-sila benzamides. *Org. Lett.* **18**, 5536–5539 (2016).
6. Ujjainwalla, F., Da Mata, M., Pennell, A., Escolano, C., Motherwell, W. B. & Vazquez, S. Synthesis of biaryls via intramolecular free radical *ipso*−substitution reactions. *Tetrahedron* **71**, 6701–6719 (2015).
7. Jiang, J., Zhang, W.-M., Dai, J.-J., Xu, J. & Xu, H.-J. Visible−light−promoted C−H arylation by merging palladium catalysis with organic photoredox catalysis. *J. Org. Chem.* **82**, 3622–3630 (2017).
8. Xia, Q., Liu, X., Zhang, Y., Chen, C. & Chen, W. Copper–catalyzed *N*–methylation of amides and *O*–methylation of carboxylic acids by using peroxides as the methylating reagents. *Org. Lett.* **15**, 3326–3329 (2013).
